# Accelerated Synthesis and Discovery of Covalent Organic Framework Photocatalysts for Hydrogen Peroxide Production

**DOI:** 10.1021/jacs.2c02666

**Published:** 2022-05-30

**Authors:** Wei Zhao, Peiyao Yan, Boyu Li, Mounib Bahri, Lunjie Liu, Xiang Zhou, Rob Clowes, Nigel D. Browning, Yue Wu, John W. Ward, Andrew I. Cooper

**Affiliations:** † Materials Innovation Factory and Department of Chemistry, University of Liverpool, Liverpool L69 7ZD, United Kingdom; ‡ Leverhulme Research Centre for Functional Materials Design, Materials Innovation Factory and Department of Chemistry, University of Liverpool, Liverpool L69 7ZD, United Kingdom; § Albert Crewe Centre for Electron Microscopy, University of Liverpool, Liverpool L69 3GL, United Kingdom

## Abstract

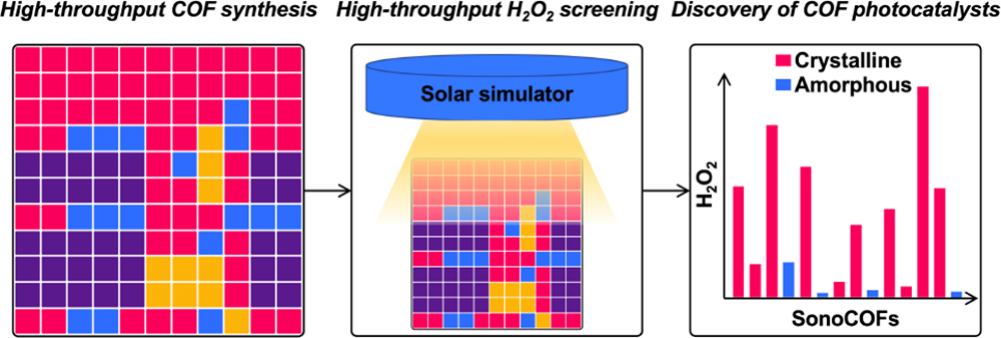

A high-throughput sonochemical synthesis and testing strategy was developed to discover covalent organic frameworks (COFs) for photocatalysis. In total, 76 conjugated polymers were synthesized, including 60 crystalline COFs of which 18 were previously unreported. These COFs were then screened for photocatalytic hydrogen peroxide (H_2_O_2_) production using water and oxygen. One of these COFs, sonoCOF-F2, was found to be an excellent photocatalyst for photocatalytic H_2_O_2_ production even in the absence of sacrificial donors. However, after long-term photocatalytic tests (96 h), the imine sonoCOF-F2 transformed into an amide-linked COF with reduced crystallinity and loss of electronic conjugation, decreasing the photocatalytic activity. When benzyl alcohol was introduced to form a two-phase catalytic system, the photostability of sonoCOF-F2 was greatly enhanced, leading to stable H_2_O_2_ production for at least 1 week.

## 1 Introduction

Hydrogen peroxide (H_2_O_2_) is an important oxidant that is used in the chemical industries, healthcare, and water treatment and as a clean fuel,^1,2^ with an annual demand of 2.2 million tons.^3^ This figure may reach 5.7 million tons per annum by 2027.^4^ Anthraquinone oxidation is the most common industrial H_2_O_2_ production method, but this consumes a lot of energy and creates harmful waste.^5^ The artificial photosynthetic production of H_2_O_2_ from water and oxygen using semiconductor photocatalysts has received attention due to its potential for low energy consumption, reduced pollution, and improved safety.^5^ To date, however, no photocatalyst exists that can realize the industrial production of H_2_O_2_ on a large scale.

Organic polymers, including graphic carbon nitride (g-C_3_N_4_),^6^ covalent triazine frameworks (CTFs),^7^ and polymer resins,^8^ have emerged as potential semiconductor photocatalysts for H_2_O_2_ production due to their tunable chemical structures, broad light absorption range, and metal-free composition. However, few organic catalysts have shown good performance for this reaction, particularly in the absence of sacrificial agents. We recently reported a linear conjugated polymer, poly(3–4-ethynylphenyl)ethynyl)pyridine (DE7), with promising photocatalytic H_2_O_2_ production, but this catalyst decomposed after a reaction period of 50 h or so, suggesting a need to focus on the photostability of organic photocatalysts.^9^

Covalent organic frameworks (COFs)^10^ are a relatively new class of porous and crystalline conjugated organic materials that have emerged as potential catalysts owing to their photocatalytic activity and (in some cases) promising stability for photocatalytic water splitting,^11^ CO_2_ reduction,^12^ and organic transformations.^13^ To date, only one study has focused on COF photocatalysts for photocatalytic H_2_O_2_ synthesis.^14^ Recently, we reported a fast and simple sonochemical method for imine COF synthesis in aqueous acetic acid.^15^ We suggested that this might be an enabling methodology for the rapid discovery of functional COF materials due to its speed, simplicity, and the lack of a requirement for anaerobic conditions, all of which lend this approach to high-throughput screening.

Here, we used this rapid and convenient sonochemical synthesis strategy to search for COF photocatalysts for photocatalytic H_2_O_2_ production from water and oxygen. We prepared 76 imine-conjugated polymers using 11 amine monomers and 11 aldehyde monomers: 60 of these materials were found to be crystalline, including 18 new, unreported COF structures with either 1D or 2D structures. High-throughput screening experiments found that a triazine-containing COF (sonoCOF-F2) showed good photocatalytic H_2_O_2_ production and improved photostability in pure water compared to our recently reported linear conjugated polymer, DE7.^9^ However, at longer reaction times (>96 h), this imine sonoCOF-F2 transformed into an amide-linked COF with reduced crystallinity and loss of electronic conjugation, decreasing the photocatalytic activity. When we added a sacrificial hole scavenger (benzyl alcohol, BA),^16^ the photocatalytic H_2_O_2_ production rate is increased, and this also protects the catalysis against transformation into the inactive amide COF. This two-phase liquid–liquid BA/water system also allows for the spontaneous separation of the reaction products.

## 2 Results and Discussion

### 2.1 High-Throughput sonoCOF Synthesis

**Figure 1:**
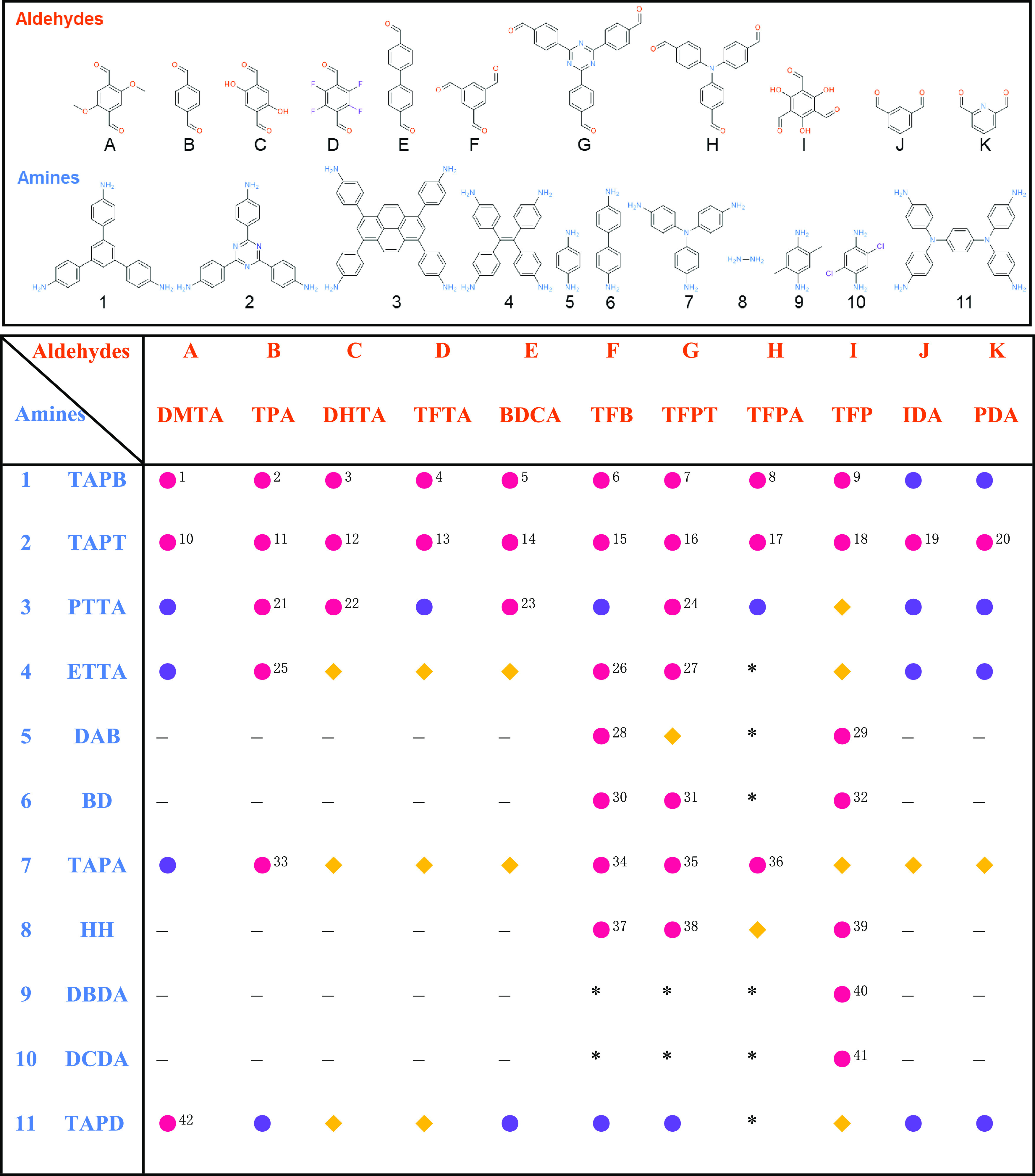
Monomer library used to synthesize the sonoCOFs and product outcomes of the 86 sonochemical reactions. Solid pink circles: reported crystalline COFs (footnotes are from Table S4); solid purple cirlces: new, unreported crystalline COFs; solid yellow diamonds: amorphous polymers; *: no polymer formed; dashes: linear polymers. The linear polymer combinations were not attempted here, giving a total of 86 sonochemical reactions.

As shown in Figure 1, 11 amine monomers and 11 aldehyde monomers were selected to synthesize a library of sonoCOFs. The monomers used in this study were selected to explore a wide range of combinations to investigate structure–function relationships for photocatalytic hydrogen peroxide production. Specifically, we chose both electron-poor monomers (e.g., aldehydes D and G and amines 2 and 10) and electron-rich monomers (e.g., aldehyde H and amines 3, 7, and 11) to generate acceptor–donor systems. The synthesis procedures for each COF were similar to our previous work.^15^ All COFs were characterized by elemental analysis (EA), powder X-ray diffraction (PXRD), Fourier-transform infrared (FT-IR) spectroscopy, scanning electron microscopy (SEM), nitrogen adsorption–desorption measurements, UV–visible absorption spectroscopy, and thermogravimetric analysis (TGA). The detailed synthesis conditions and characterization results of each COF can be found in the Supplementary Information.

**Figure 2:**
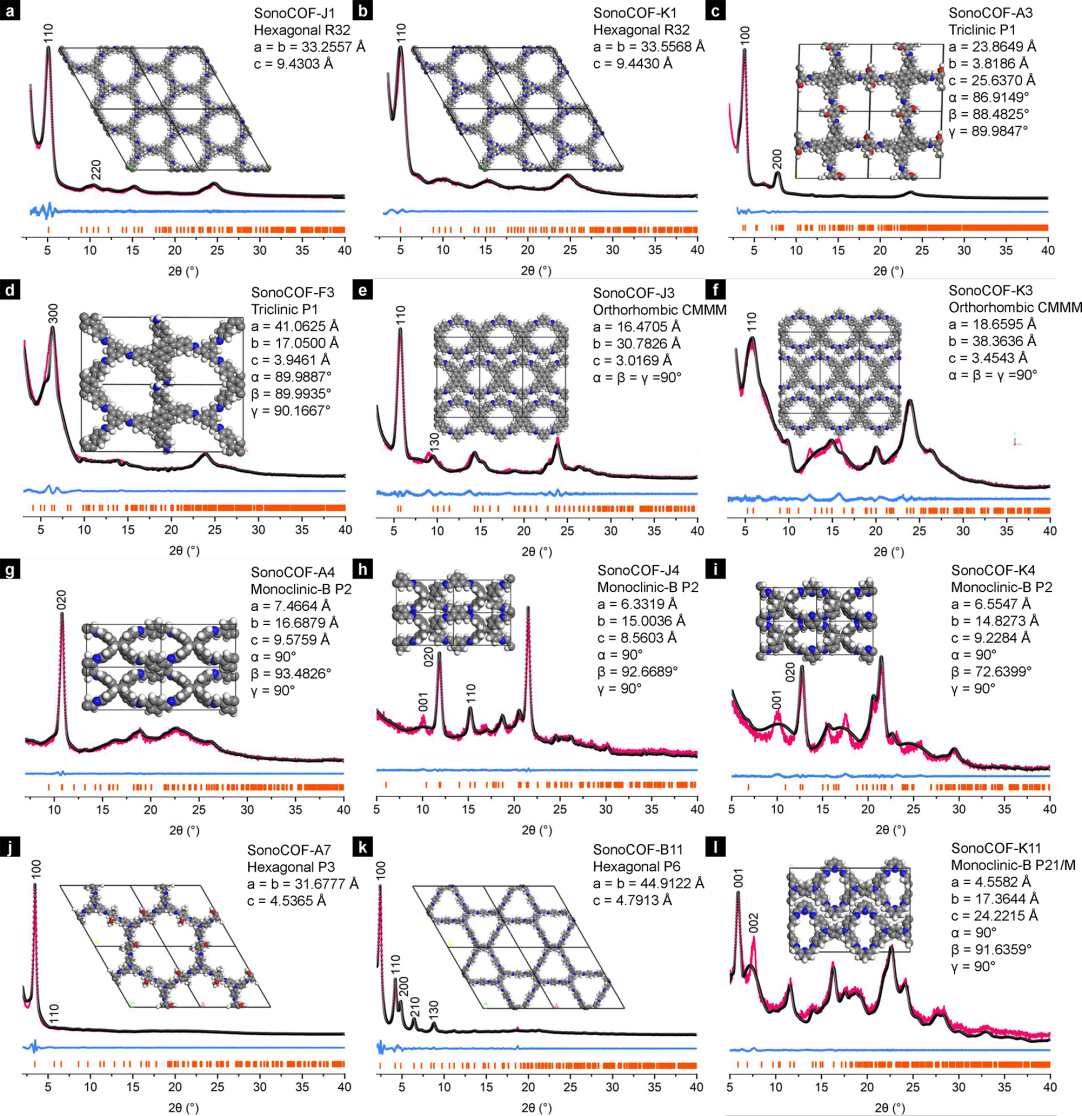
Pawley refinements against the powder X-ray diffraction patterns of (a) sonoCOF-J1, (b) K1, (c) A3, (d) F3, (e) J3, (f) K3, (g) A4, (h) J4, (i) K4, (j) A7, (k) B11, and (l) K11. Pink lines: *y*_obs_ (experimental PXRD data). Black dots: *y*_calc_ (Pawley refinement profile). Blue lines: *y*_obs_ – *y*_calc_ (residual); yellow marks, *hkl* positions calculated for that phase. Insets: modeled crystal structures. C, gray; H, white; N, blue; O, red.

In total, 76 conjugated polymers were synthesized successfully (10 combinations failed to give polymers, Figure 1), of which 60 materials showed good crystallinity. This included 18 unreported crystalline COF structures with 1D or 2D topologies (Figure 2). To obtain crystalline materials, the concentration of acetic acid (AcOH) and the activation conditions must be considered because of the different reactivities of the various monomers and the different stabilities of the resulting frameworks. As such, there is no global optimum synthesis or work-up method. For example, sonoCOF-A1 is very chemically stable and its monomers, DMTA and TAPB, show good reactivities; hence, we could prepare and isolate crystalline sonoCOF-A1 using various concentrations of AcOH (1, 3, 6, 9, and 12 M) by direct filtration using vacuum drying, albeit with different yields.

Other COFs are more fragile, such as sonoCOF-B1 and sonoCOF-C1, and these materials are more sensitive to the synthesis and activation conditions. In particular, excessive amounts of water seem to inhibit the COF formation. Moreover, the high surface tension of water may result in the formation of amorphous frameworks due to pore collapse during activation if the materials are dried directly. Several studies have shown that the activation process, including the use of low-surface-tension solvents^17^ and supercritical CO_2_ activation,^18,19^ can be important for such frameworks. As such, it is conceivable that some of the materials that were isolated here as amorphous polymers might be accessed as crystalline frameworks with alternative work-up procedures.

There were four top-level conclusions for these aqueous sonoCOF syntheses:

(i) In general, a higher concentration (12 M) of AcOH was more favorable for the formation of most COFs. In most cases, this both increases the solubility of the monomers and also catalyzes COF formation.
(ii) Specific activations, such as the use of low-surface-tension solvents (hexane) or supercritical CO_2_ activations, are crucial for the isolation of fragile COFs but unnecessary for other more robust frameworks (e.g., sonoCOF-A1). The most generalizable conditions for COF formation were 12 M AcOH with low-surface-tension solvent (hexane) activation or supercritical CO_2_ activation.
(iii) For some monomers with low reactivity, such as TFPA, it was difficult to form a solid polymer product under sonochemical conditions.
(iv) In general, keto-enamine COFs showed lower crystallinity due to the reduced reversibility in the condensation reaction.^20^

The experimental powder X-ray diffraction (PXRD) patterns for 12 of the 18 unreported COFs are shown in Figure 2. All of these COFs have either 1D or 2D structures. The PXRD measurements showed diffraction peaks that are consistent with the simulated structures (Figures S6–S9). The experimental PXRD patterns for sonoCOF-J1 and K1 matched well with a simulated ABC-stacking arrangement. SonoCOF-A3, A7, and B11, in particular, showed good crystallinity with intense and sharp low-angle reflections, which matched well with a simulated AA-stacking arrangement. The diffraction pattern of sonoCOF-F3 was very similar to that of the isostructural PT-COF^21^ with a *bex* topology. The experimental PXRD patterns of sonoCOF-J3, K3, A4, J4, K4, and K11 matched well with 1D simulated structures. We believe that these 1D COFs have lower crystallinity because they cannot form noncovalent π–π-stacked 2D layers, which are known to enhance crystallinity in COFs. The unit cell parameters of the sonoCOFs were refined using the Pawley method.

### 2.2 High-Throughput Screening for Photocatalytic H_2_O_2_ Production

High-throughput screening measurements have been used previously to identify photocatalysts for water splitting.^22^ Here, we used an analogous approach for H_2_O_2_ production. The 76 functionally diverse conjugated polymers, including 60 crystalline sonoCOFs, were screened for H_2_O_2_ production in pure water (no added sacrificial donors) in air using a high-throughput screening platform (see the Supplementary Information).

**Figure 3:**
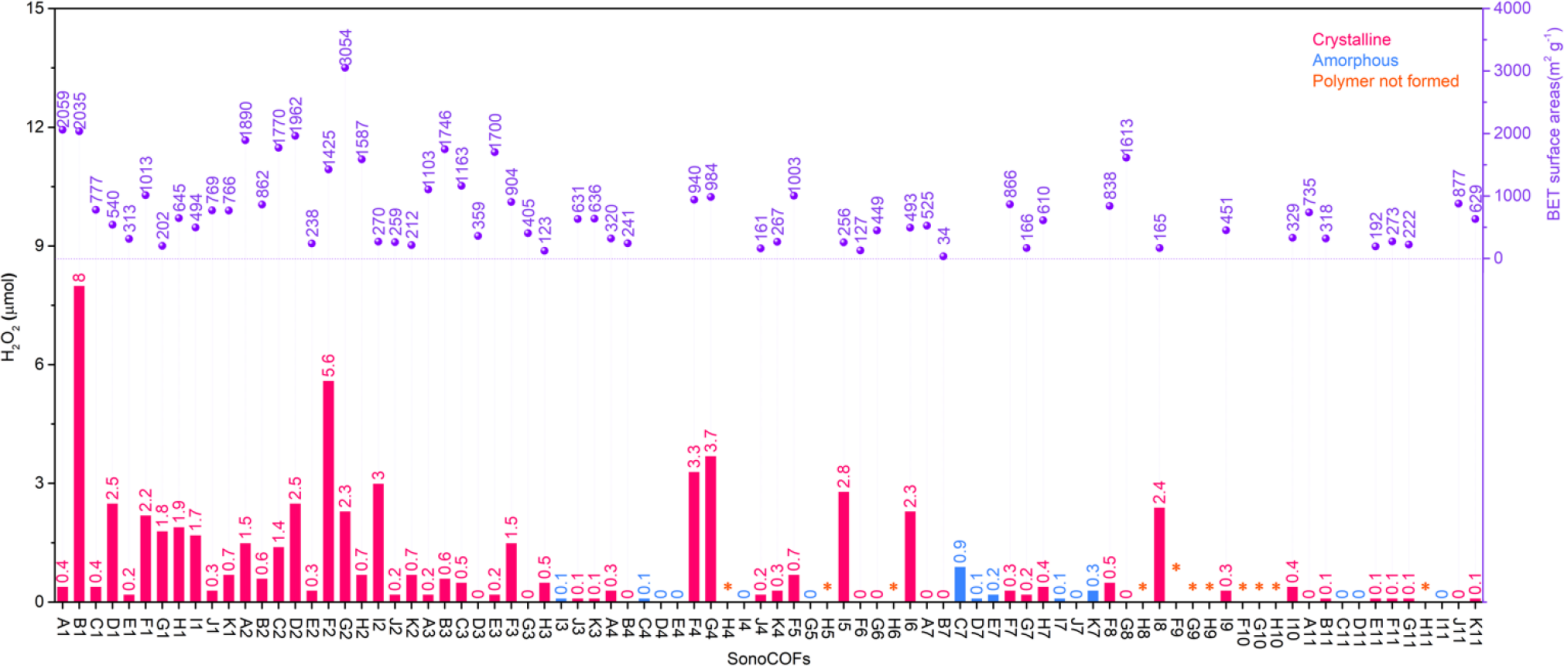
High-throughput discovery of sonoCOFs for photocatalytic H_2_O_2_ production in the absence of any added sacrificial reagents. Reaction conditions: 3 mg of polymer, 5 mL of water, air, simulated solar light for 1.5 h (Oriel Solar Simulator, 1.0 sun). Left axis: amount of H_2_O_2_ produced (bars); right axis: BET surface areas for the crystalline sonoCOFs (circle points).

High crystallinity^23^ and (arguably) porosity^24^ are thought to be favorable for photocatalytic performance. The benefits of crystallinity were strongly apparent here: none of the amorphous materials in the library showed H_2_O_2_ production levels of greater than 1 μmol (Figure 3), and all the materials that showed high H_2_O_2_ production (>2 μmol) were crystalline COFs. This shows that crystalline structures are more suitable for photocatalytic H_2_O_2_ production. Most of the best photocatalysts in the library contained triazine (points labeled as G and/or 2 in Figure 3, for example sonoCOF-G2, G4, F2, and D2); this may be because these materials promote two-electron oxygen reduction.^6,25^ Keto-enamine-based COFs (e.g., sonoCOF-I2, I5, and -I8) also tended to show good catalytic performance. The relationship between porosity and H_2_O_2_ production is shown in Figure 3. In general, there is little evidence for a correlation here: the four COFs with the highest H_2_O_2_ production (> 3.3 μmol) also have high Brunauer–Emmett–Teller (BET) surface areas (>940 m^2^ g^–1^), but then again, most of the materials in this library are porous, and many porous COFs have low catalytic activity (e.g., sonoCOF-A1, E3, and G8). In general, we found that acceptor–donor systems are beneficial for high activity. For example, sonoCOF-F2 produces twice as much hydrogen peroxide as sonoCOF-F1 under visible light irradiation. The only difference between the two structures is that the benzene at the core of sonoCOF-F1 is replaced with a triazine, thus generating electron-rich and electron-poor sites for enhanced charge separation and photocatalytic activity. All of the sonoCOFs studied absorb visible light with an experimental optical band gap of less than 2.90 eV (Figures S45–S51). The positions of the conduction band (CB) and the valence band (VB) of the COFs govern the reduction of O_2_ and the oxidation of H_2_O, respectively. As such, both the optical band gap and the band positions play an important role in photocatalytic H_2_O_2_ production. A comparison between these properties and the catalytic activity is provided in Figures S57–S59. As found for photocatalytic H_2_ production using linear conjugated polymers,^22^ no single factor governs the catalytic activity, but some broad trends can be observed. In general, larger band gaps (>2.4 eV) lead to the highest catalytic activities (Figure S57). Most of the measured sonoCOFs have VB positions that should promote H_2_O oxidation (sonoCOF-I6 and sonoCOF-I8 are exceptions and have relatively low activity). All of the measured sonoCOFs have CB positions that should allow O_2_ reduction (Figure S59), with H_2_O_2_ production broadly increasing as CB values are more negative. Again, these plots illustrate the power of high-throughput methods here since neither the band gap nor CB/VB energy levels are solely deterministic for catalytic activity. We note that sonoCOF-A11 has the same chemical structure as the COF TAPD-(OMe)_2_ that was reported previously to catalyze H_2_O_2_ production in the presence of sacrificial donors.^14^ For comparison, we also prepared this COF solvothermally (named here as solvoCOF-A11). However, neither sonoCOF-A11 nor solvoCOF-A11 showed measurable photocatalytic activity in pure water.

SonoCOF-B1 showed the highest H_2_O_2_ production in this library over a short irradiation period of 1.5 h using simulated solar light (Figure 3). However, we found that the crystallinity of sonoCOF-B1 was lost rapidly during the reaction (Figure S61a). Also, long-term tests showed that the photocatalytic performance decreased substantially after 24 h (Figure S73). By contrast, sonoCOF-F2 showed good performance for photocatalytic H_2_O_2_ production and promising photostability over short reaction times according to powder X-ray diffraction (PXRD) analysis. Considering that both performance and photostability are important, we chose sonoCOF-F2 for more detailed study.

### 2.3 Investigation of the Photocatalytic H_2_O_2_ Production Mechanism

**Figure 4:**
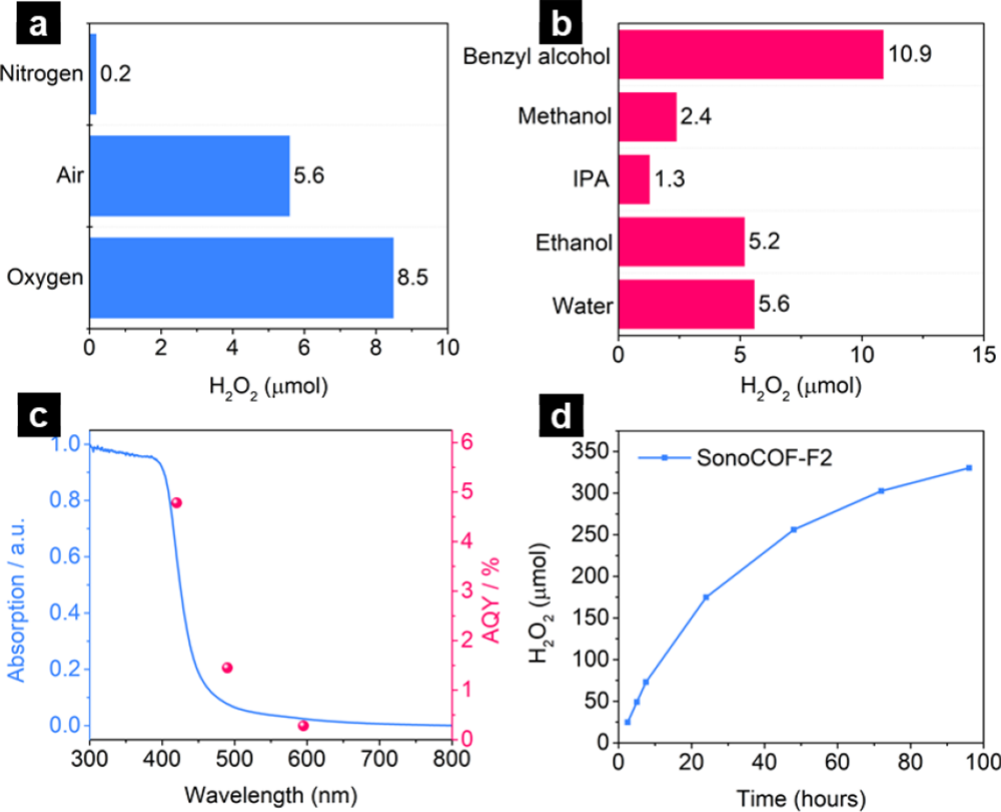
(a) Reactions using sonoCOF-F2 under different gas atmospheres: 3 mg of COF in 5 mL of water, 1.5 h illumination (Oriel Solar Simulator, 1.0 sun). (b) Photocatalytic H_2_O_2_ production for sonoCOF-F2 in neat water, with ethanol, isopropanol (IPA), methanol, and benzyl alcohol (3 mg of polymer, 4.5 mL of water, and 0.5 mL of solvents), all with 1.5 h illumination (Oriel Solar Simulator, 1.0 sun). (c) Wavelength-dependent AQE values (measured in the first 1 h) and solid-state UV–visible spectrum of sonoCOF-F2. (d) Longer-term photocatalytic H_2_O_2_ production of sonoCOF-F2: 60 mL of water and 50 mg of sonoCOF-F2; 300 W Xe lamp; λ > 420 nm.

To gain insight into the reaction mechanism, a series of experiments were conducted using a high-throughput screening platform (Supplementary Information). As shown in Figure S74, visible light irradiation is critical for the production of H_2_O_2_: in the absence of light, no H_2_O_2_ was produced over 48 h. An oxygen-rich atmosphere also favors H_2_O_2_ production; very little H_2_O_2_ (0.2 μmol) was produced under a nitrogen atmosphere, whereas 8.5 μmol was produced under pure O_2_ (99%), which is 1.5 times greater than the amount produced in air (Figure 4a). This was further confirmed by isotopic labeling experiments using ^18^O_2_ (Figure S75): the percentage of ^18^O_2_ detected by mass spectrometry in the H_2_O_2_ produced increased from 0 to 63.2% after 22 h.

Active species-trapping experiments were performed in air by using AgNO_3_, *tert*-butyl alcohol (TBA), and benzoquinone (BQ) as electron (e^–^), hydroxyl radical (·OH), and superoxide radical (·O_2_^–^) scavengers. Due to the interference of BQ with the potassium iodide titrimetric assay, the relative peroxide levels were estimated using peroxide test sticks. As shown in Figure S63, the production of H_2_O_2_ decreases sharply when AgNO_3_ is added, indicating that photogenerated electrons play a vital role in the photocatalytic oxygen reduction reaction (ORR). Almost no H_2_O_2_ was detected when BQ was added, suggesting that ·O_2_^–^ is involved. By contrast, the addition of TBA has almost no influence on the H_2_O_2_ production, suggesting that ·OH did not participate in the photocatalytic process. Based on these combined results, we suggest that the photoinduced H_2_O_2_ production of sonoCOF-F2 involves the stepwise reduction of O_2_ (O_2_ → ·O_2_^–^ → H_2_O_2_).

The apparent quantum yield (AQY) was measured at different wavelengths to evaluate the photocatalytic H_2_O_2_ production performance. The AQY was determined to be 4.8% at 420 nm, which followed the absorption spectrum, supporting a photoinduced H_2_O_2_ generation process (Figure 4c).

Hole scavengers, including ethanol, isopropanol (IPA), methanol, and benzyl alcohol (BA), were added to gain further insight into the mechanism (Figure 4b). A decrease was observed in the photocatalytic efficiency in the presence of ethanol, IPA, and methanol (all single-phase systems), but a marked increase in activity was observed in the presence of BA (a two-phase, liquid–liquid system). In the two-phase system (water/BA), the sonoCOF-F2 was selectively dispersed in the BA phase and H_2_O_2_ was produced in the aqueous phase, which may perhaps avoid the photocatalytic H_2_O_2_ decomposition (i.e., the back reaction), thus increasing the overall peroxide production rate.

### 2.4 Long-Term Photocatalytic H_2_O_2_ Production

To be practically useful, the long-term photostability of catalysts is essential. We therefore tested the photostability of sonoCOF-F2 using a continuous experiment (96 h) in pure water (Figure 4d). As shown in Table S3, the photocatalytic H_2_O_2_ production rate of sonoCOF-F2 is higher than most organic materials reported under similar conditions but lower than a linear conjugated polymer, DE7.^9^ After about 72 h, the rate of H_2_O_2_ generation for sonoCOF-F2 decreased. Similar photocatalytic H_2_O_2_ production profiles were observed in previous studies that involved composite photocatalysts (procyanidin–methoxybenzaldehyde (PM) dipolymers with carbon dots)^26^ and linear polymer photocatalyst (DE7).^9^

**Figure 5:**
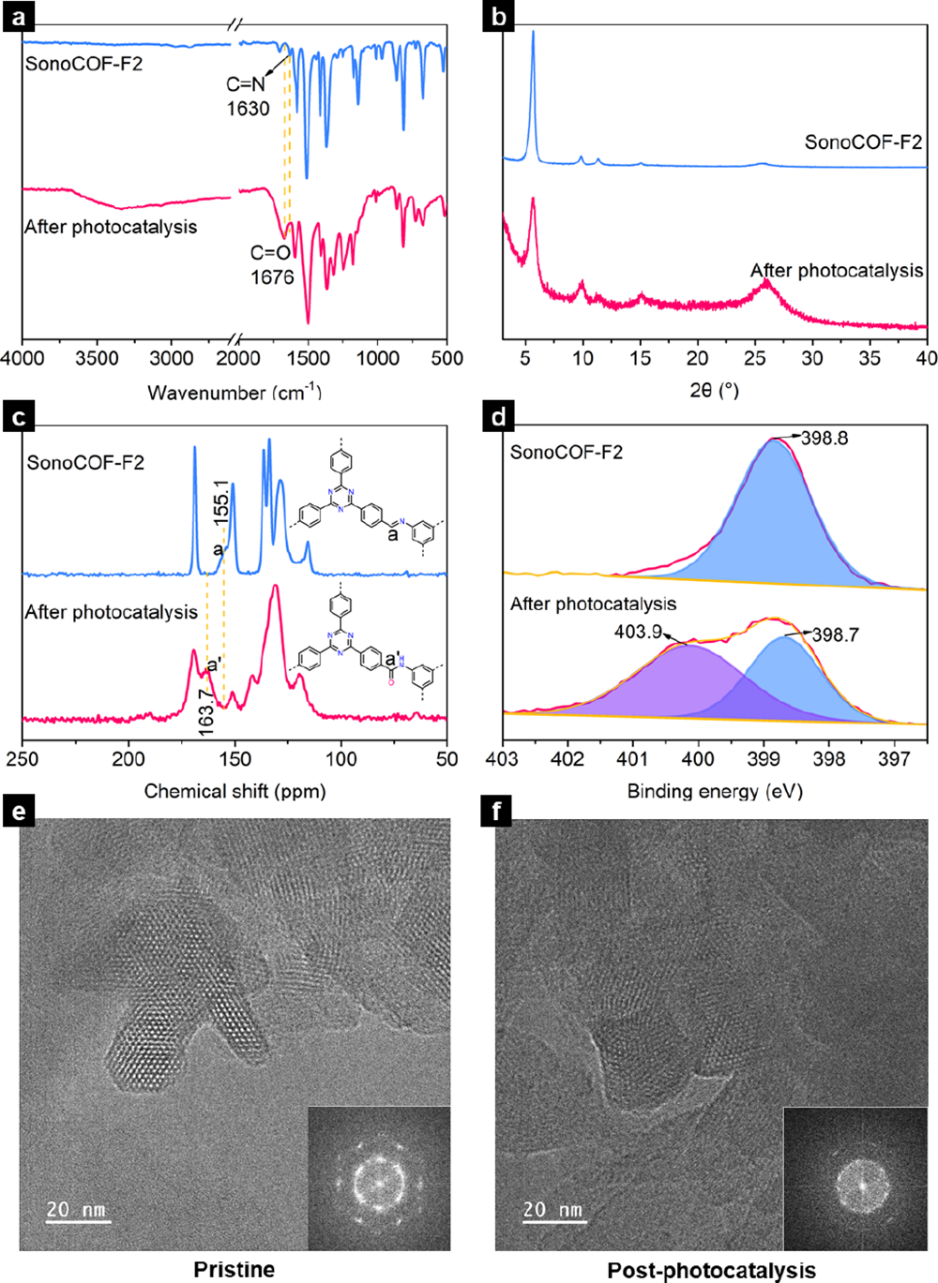
(a) FT-IR, (b) PXRD, (c) CP-MAS NMR, and (d) N 1s XPS spectra and (insets in (e) and (f): Fourier transform (FFT) images) HRTEM images of sonoCOF-F2 before and after a long-term photocatalytic testing (96 h). Reaction conditions: 50 mg of sonoCOF-F2, 60 mL of water, O_2_, 300 W Xe lamp (λ > 420 nm) for 96 h.

To understand why the catalytic efficiency of sonoCOF-F2 decreased over longer periods of photolysis, we used FT-IR, PXRD, CP-MAS ^13^C NMR, X-ray photoelectron spectroscopy (XPS), and transmission electron microscopy (TEM) to characterize the structure of sonoCOF-F2 before and after photocatalysis. FT-IR measurements showed that the imine bond (C=N) at 1630 cm^–1^ disappeared and a new peak from amide bond (C=O) at 1676 cm^–1^ emerged after photocatalysis (Figure 5a), indicating that the imine linkage was oxidized to an amide linkage by photogenerated holes or radicals such as ·O_2_^–^. CP-MAS ^13^C NMR and XPS spectra further confirmed this transformation. After photocatalysis, the resonance at 155.1 pm associated with the imine functionality disappeared in the CP-MAS ^13^C NMR spectrum, and a new signal appeared (163.7 ppm), which was assigned to the amide bond carbon (Figure 5c). The characteristic N 1s signal of the sp^2^-bonded nitrogen in the imine bonds and triazine rings was observed at 398.8 eV for sonoCOF-F2. However, the N 1s spectrum showed two separate resolved peaks after photocatalysis. The main peak at 398.7 eV corresponds to the nitrogen in the triazine rings, and the other signal at 403.9 eV is ascribed to the nitrogen in the amide group (Figure 5d). This transformation from the imine linkage into an amide bond is one reason for the decrease in catalytic efficiency because of the loss of extended conjugation. To confirm that this transformation was not caused by light itself or by H_2_O_2_, sonoCOF-F2 was illuminated in water and stirred in 20 mM H_2_O_2_ solution under a nitrogen atmosphere for 24 h. There was no change in FT-IR and PXRD patterns in either case (Figure S64), indicating that this transformation was caused by photogenerated holes or radicals rather than light or hydrogen peroxide. The conduction band (CB) and valence band (VB) (Figure S60) for sonoCOF-F2 are estimated to be −2.0 V (vs NHE) and 0.86 V (vs NHE), respectively, which suggests that reduction of oxygen is thermodynamically possible but water oxidation to O_2_ (1.23 V vs NHE)^27^ and two-electron water oxidation (1.76 V vs NHE)^28^ directly to H_2_O_2_ are not. We propose that photogenerated holes that do not participate in water oxidation could react with the COF itself and contribute to the degradation of catalytic performance. In addition, both PXRD (Figure 5b) and HR-TEM images (Figure 5e,f) showed that sonoCOF-F2 had reduced crystallinity levels after photocatalysis compared to the pristine COF, which could be a secondary reason for the decrease in catalytic efficiency.

**Figure 6:**
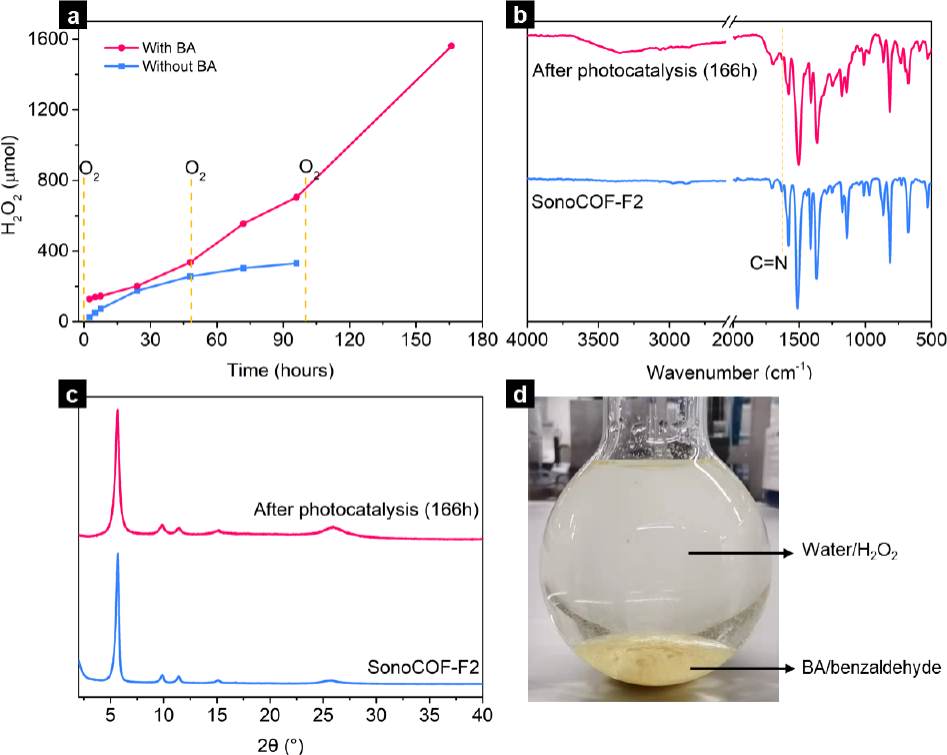
(a) Comparison of long-term photocatalytic H_2_O_2_ production using sonoCOF-F2 with and without benzyl alcohol. (b) FT-IR and (c) PXRD spectra of sonoCOF-F2 before and after long-term photocatalytic testing using benzyl alcohol (166 h). Reaction conditions: 50 mg of sonoCOF-F2, 60 mL of water or water/benzyl alcohol (9/1), O_2_, Xe lamp (λ > 420 nm) for 166 h. (d) Image of sonoCOF-F2 dispersed in a mixture of water/benzyl alcohol (9/1).

While sonoCOF-F2 seems to have improved photostability compared to DE7^9^ and procyanidin–methoxybenzaldehyde (PM) dipolymers,^26^ the stability is still far too low for practical applications. We therefore sought to improve the photostability and photocatalytic H_2_O_2_ production performance of sonoCOF-F2 by system design. Benzyl alcohol (BA) is a hole scavenger for photocatalytic H_2_O_2_ production, and it was found to be effective in this sonoCOF-F2 system. Importantly, sonoCOF-F2 was found to be selectively dispersed in the BA phase in this two-phase system of water/BA mixture (Figure 6d), which realizes spontaneous separation of the benzaldehyde that is formed (in the BA phase) and of the photoproduced H_2_O_2_ (in the aqueous phase). Photocatalytic reduction of H_2_O_2_ to OH^–^ and ·OH can be avoided in this two-phase system, which will increase the overall activity.

A continuous photocatalytic experiment (166 h) was performed in a water/BA (9/1, volume) mixture. This long-term photocatalytic test showed that there was no decrease in rate even after 166 h (Figure 6a), with approximately linear kinetics throughout. There was no change in FT-IR spectra or PXRD patterns for sonoCOF-F2 before and after 166 h of photocatalysis, suggesting excellent photostability under these conditions (Figure 6b,c). To compare the photocatalytic activity with TAPD-(OMe)_2_ COF^14^ when using hole scavengers, we performed a long-term photocatalytic H_2_O_2_ production test using exactly the same photocatalysis set-up (Supplementary Information, Figure S70). The only difference with the reported procedure was that ethanol was used in place of BA. Over a reaction period of 96 h, sonoCOF-F2/BA produced 275.2 μmol of H_2_O_2_, which is almost two times the amount produced by TAPD-(OMe)_2_ COF/ethanol (142.3 μmol). Also, longer-term photocatalytic tests showed that sono-COF-F2 could produce H_2_O_2_ continuously over 1 week using a two-phase mixture of water/BA (1/9) (Figure S72) and 3482.8 μmol of H_2_O_2_ was obtained over 168 h (final concentration of H_2_O_2_ = 116 mM and an average sustained H_2_O_2_ production rate of 414.6 mmol h^–1^ g^–1^ based on the mass of the catalyst).

## 3 Conclusions

A high-throughput sonochemical synthesis strategy of imine COFs was developed and applied for the discovery of functional COFs as photocatalysts for photocatalytic H_2_O_2_ production. Imine-based sonoCOF-F2 was found to be an active photocatalyst for photocatalytic H_2_O_2_ production in the absence of any sacrificial agents, but it is unstable over prolonged reaction times, transforming into an amide COF. Benzyl alcohol was introduced to form a two-phase catalytic system, which both improves the photocatalytic H_2_O_2_ production performance and also protects the COF structure to enhance its photostability. Moreover, the two-phase system separates the reaction products. While the transformation of benzyl alcohol to benzaldehyde may not provide a practical solution for H_2_O_2_ generation, the basic concept could be extended to other “sacrificial” agents. For example, it might be possible to selectively oxidize waste materials, such as biomass, to produce value-added chemicals in parallel with H_2_O_2_ production.

## Supporting Information Available

Experimental details; elemental analysis data; UV/Vis and FT-IR spectra; PXRD patterns; TGA curves; gas sorption data; CV data; SEM and TEM images; hydrogen peroxide production data (PDF)
