# Supplementary material for: Accelerated Synthesis and Discovery of Covalent Organic Framework Photocatalysts for Hydrogen Peroxide Production
Source: J Am Chem Soc. 2022 May 30;144(22):9902–9. doi: 10.1021/jacs.2c02666 (PMC9185744; doi:10.1021/jacs.2c02666)
Supplement: Supplementary file 1 — ja2c02666_si_001.pdf [file ja2c02666_si_001.pdf]

Supporting Information

Accelerated Synthesis and Discovery of Covalent Organic Framework Photocatalysts for Hydrogen Peroxide Production

Wei Zhao[1, 2], Peiyao Yan[1], Boyu Li[1], Mounib Bahri[3], Lunjie Liu[1], Xiang Zhou[1], Rob Clowes[1], Nigel D. Browning[3], Yue Wu[1], John W. Ward*[1, 2] and Andrew I. Cooper*[1, 2]

[1] Department of Chemistry and Materials Innovation Factory, University of Liverpool, Crown Street, Liverpool L69 7ZD, UK.

[2] Leverhulme Research Centre for Functional Materials Design, Materials Innovation Factory and Department of Chemistry, University of Liverpool, Liverpool, UK.

[3] Albert Crewe Centre for Electron Microscopy, University of Liverpool, Liverpool, L69 3GL, UK.

Table of contents

1. **Instrumentation and materials** S2
2. **Synthetic procedures** S6
3. **Characterization of COFs** S8
4. **Photocatalytic hydrogen peroxide production** S68
5. **Structural refinements against PXRD data** S80
6. **^{1}H NMR and ^{13}C NMR spectra** S121
7. **References** S122

1. Instrumentation and materials

Reagents were obtained from Sigma-Aldrich, TCI Europe or Fluorochem Ltd. Anhydrous solvents were purchased from Acros Organics or Fisher Scientific. All chemicals were used as received without further purification.

Solution nuclear magnetic resonance. ^{1}H and ^{13}C NMR spectra were recorded in solution on a Bruker Avance 400 NMR spectrometer at 400 MHz and 100 MHz, respectively.

^{13}C cross polarization magic angle spinning nuclear magnetic resonance. Carbon-13 magic-angle spinning measurements were carried out at 100.63 MHz using a Bruker Avance III HD spectrometer and 4 mm (rotor o.d.) probe. Spectra were acquired at a spin rate of 10 kHz. Cross-polarisation (CP) spectra were recorded with TOSS spinning sideband suppression, 1 ms contact time, and with a recycle delay of 1 s. Carbon spectral referencing is relative to neat tetramethylsilane, carried out by setting the high frequency signal from an external sample of adamantane to 38.5 ppm.

Fourier-transform infrared spectroscopy (FT-IR). IR spectra were recorded on a Bruker Tensor 27 FT-IR spectrometer using KBr pellets.

Powder X-ray diffraction (PXRD). PXRD measurements were performed on a PANalytical X'Pert PRO MPD, using in high throughput transmission mode with Kα focusing mirror and PIXCEL 1D detector with a Cu X-ray source.

Structural modelling of COFs. Structural atomistic simulations of the possible framework structures were carried out using *Material Studio* software. The simulated PXRD patterns were determined by the Reflex module. The space group for simulated structures was selected as P1.

Thermogravimetric analysis (TGA). TGA analysis was performed on an EXSTAR6000 under a flow of nitrogen by heating from room temperature to 800 °C at a rate of 10 °C min^{-1}.

Gas sorption analysis. The surface areas and nitrogen adsorption isotherms of samples (at 77.3 K) were obtained using a Micromeritics ASAP 2020 volumetric adsorption analyzer. Before analysis, the samples were degassed at 120 °C for 12 h under vacuum (10^{-5} bar). Pore size distributions of COFs were obtained by fitting the density functional theory (DFT) model to the adsorption data.

Elemental analysis (EA). EA was determined on Thermo EA1112 Flash CHNS-O analyzer with standard microanalytical procedures.

Scanning electron microscopy (SEM). SEM images were collected on a Hitachi S-4800 cold field emission scanning electron microscope. Samples were prepared by depositing the dry powders on 15

mm Hitachi M4 aluminum stubs using an adhesive high-purity carbon tape before coating with a 2 nm layer of gold using an Emitech K550X automated sputter coater. Imaging was conducted at a working voltage of 3 kV and a working distance of 8 mm using a combination of upper and lower secondary electron detectors.

Transmission electron microscopy (TEM). TEM images were obtained on a JEOL 2100FCs microscopy at an accelerating voltage of 200 kV. The samples were prepared by drop-casting sonicated ethanol suspensions of the materials onto a copper grid.

UV-visible absorption spectra. UV-visible absorption spectra of the polymers were measured on a Shimadzu UV-2550 UV-vis spectrometer by measuring the reflectance of powders in the solid state.

XPS spectra. XPS Analysis was performed using a Thermo K-Alpha^{+} XPS system fitted with a microfocused monochromatic Al kα X-ray source (1486.7 eV) operating at 6mA × 12 kV (72W). All data was recorded with a X-ray beam size of 600 x 400 μm. Survey scans were recorded at a pass energy of 200 eV, and high-resolution scans recorded at a pass energy of 50 eV. Electronic charge neutralization, where required, was achieved using a dual-beam low-energy electron/ion source (Thermo Scientific FG-03). Ion gun current = 100 μA. Ion gun voltage = 40 V with a chamber pressure from the Ar gas of 10^{-7} mbar.

Cyclic voltammetry (CV). CV curves were carried out by a Bio-logic SP200 electrochemical workstation in a normal three electrode cell (glassy carbon as the working electrode, Pt wire as counter electrode and Ag/AgCl electrode as the reference electrode). The experiments were carried out in acetonitrile solutions with 0.1 M tetra-n-butylammonium hexafluorophosphate ($TBAPF_6$) as the supporting electrolyte at a scan rate of 100 mV s^{-1}. The ferrocenium/ferrocene (Fc/Fc^{+}) redox couple was used as an external potential reference.

$E_{HOMO} = -(E_{ox,onset} - E^{(1/2)}Fc/Fc^{+} + 4.8)$ eV   vs. vacuum

$E_{LUMO} = E_{HOMO} + E_g$ vs. vacuum

Reference: $E^{(1/2)}Fc/Fc^{+} = (0.10+0.03)/2=0.455$

High-throughput photocatalytic H_2O_2 production experiment. A sample vial was charged with a sonoCOF (3 mg) and either water (5 mL) or water (4.5 mL) plus a sacrificial reagent (0.5 mL), and then ultrasonicated for 10 min (to disperse the COF materials) after being capped under air. The photocatalytic H_2O_2 evolution experiments were performed on an Oriel Solar Simulator 94123A with an output of 1.0 sun (Class AAA, 1440 W xenon, 12 × 12 in.). After 1.5 h, 1 mL solution was sampled with an injection

syringe after shook evenly and then filtered with a 0.2 μm Millipore filter to remove the photocatalyst. The amount of H_2O_2 produced was analyzed with *Quantofix* peroxide test sticks (for semi-quantitative initial screening) or a KI titrimetric method (for quantification).

Kinetic H_2O_2 production experiment. A flask was charged with 50 mg of COF powders and either water (60 mL) or water (54 mL) plus benzyl alcohol (6 mL) and sealed with a rubber septum. The suspension was ultrasonicated for 30 min in dark to disperse well before degassing by O_2 bubbling for 30 min. The reaction solution was illuminated by a 300 W Xe lamp with a filter (λ > 420 nm) and was kept at room temperature by air cooling. The concentration of H_2O_2 was determined using a KI titrimetric method.

AQY measurement. The apparent quantum yield (AQY) was determined under monochromatic LED light irradiation at a certain wavelength (λ = 420 nm, 490 nm or 595 nm), and the light intensity was measured by a ThorLabs PM100D Power with a photodiode sensor.

The AQY was calculated using the following equation:

$$AQY\ \% = \frac{[\mathrm{H_2O_2\ produced\ (mol)}] \times 2}{\mathrm{photon\ number\ entered\ into\ the\ reactor\ (mol)}} \times 100$$

$$= \frac{[N_\mathrm{a} \times h \times c)][\mathrm{H_2O_2\ produced\ (mol)}] \times 2}{\mathrm{I \times S \times t \times \lambda}} \times 100$$

Where, N_a is Avogadro's constant (6.022 × 1023 mol^{-1}), h is the Planck constant (6.626 × 10^{-34} J s), c is the speed of light (3 × 108 m s^{-1}), S is the irradiation area (cm^2), I is the intensity of irradiation light (W cm^{-2}), t is the photoreaction time (s), λ is the wavelength of the monochromatic light (m).

Gas chromatography (Quantification of produced benzaldehyde)

The amount of produced benzaldehyde was analyzed by gas chromatography (Agilent, 7890A). Column: DB-WAXetr, 60 m * 0.25 mm * 0.25 μm.

Ultrasound experiments. All sonoCOFs were prepared using a Branson Sonifier SFX550 cell disrupter with O.D. 3 mm microtip probe.

Isotopic exchange experiments. A vial was charged with sonoCOF-F2 (50 mg) and $H_2{}^{16}O$ (50 mL) and sealed with a rubber septum. Helium gas was then bubbled through the solution for 20 min and $^{18}O_2$ gas (purity : 99%) for 5 min. The vial was illuminated using a Xe lamp with a filter (λ >420 nm) for 22 hours, then He gas was bubbled again to remove the $^{18}O_2$ gas. The reaction mixture was then injected into a vial containing MnO_2 and He gas, and the gas produced from the decomposition phase was detected by an

Agilent 7890B GC-MS system. For the 0 h experiment, exactly the same procedures were used, except with no irradiation.

2. Synthetic procedures

1.1. Synthesis of COFs using ultrasonication

The amine and aldehyde monomers and solvents were added into a vial and sonicated (550 W Branson Sonifier SFX550 cell disrupter with O.D. 3 mm microtip) in continuous mode for 60 minutes. The details can be found in Table S1. The resulting solid was washed in sequence with acetone and methanol, followed by a 24 h Soxhlet extraction with THF. The sample was then dried directly under high vacuum at 80 °C for 24 h, or washed and immersed in hexane (12 h) to replace all other solvents and then dried under high vacuum at 80 °C for 24 h, or activated using supercritical CO_2 drying.

Table S1. Summary of sonochemical synthetic details.

COFs	Amines	Aldehydes	Concentration of aqueous AcOH	Volume of aqueous AcOH (ml)	Activation	Sonication power (%)	Yield (%)
SonoCOF-A1	TAPB 28.2 mg	DMTA 23.2 mg	6 M	2	Direct drying	50	93
SonoCOF-B1	TAPB 105.3 mg	TPA 60.3 mg	12 M	6	Hexane	50	87
SonoCOF-C1	TAPB 3.2 mg	DHTA 25 mg	12	2	$scCO_2$ drying	50	80
SonoCOF-D1	TAPB 35.1 mg	TFTA 31 mg	12	4	Hexane	25	77
SonoCOF-E1	TAPB 105.5 mg	BDCA 94.5 mg	12	10	Hexane	25	73
SonoCOF-F1	TAPB 35.1 mg	TFB 16.2 mg	6	2	Direct drying	50	84
SonoCOF-G1	TAPB 17.6 mg	TFPT 19.7 mg	6	2	Hexane	50	58
SonoCOF-H1	TAPB 35.1 mg	TFPA 32.9 mg	12	2	Direct drying	50	50
SonoCOF-I1	TAPB 35.2 mg	TFP 21 mg	9	4	Direct drying	50	72
SonoCOF-J1	TAPB 35.1 g	IDA 20.2 mg	12	2	Direct drying	50	52
SonoCOF-K1	TAPB 35.2 mg	PDA 20.2 mg	6	2	Direct drying	50	94
SonoCOF-A2	TAPT 56.8 mg	DMTA 46.4 mg	6	2	Direct drying	50	87
SonoCOF-B2	TAPT 35.4 mg	TPA 20.1 mg	12	2	$scCO_2$ drying	50	86
SonoCOF-C2	TAPT 31.9 mg	DHTA 21.6 m	12	2	Direct drying	50	95
SonoCOF-D2	TAPT 17.7 mg	TFTA 15. mg	12	2	Direct drying	50	88
SonoCOF-E2	TAPT 106.5 mg	BDCA 94.5 mg	12	10	Hexane	25	70
SonoCOF-F2	TAPT 399 mg	TFB 183 mg	12	18	Direct drying	40	81
SonoCOF-G2	TAPT 53 mg	TFPT 59 mg	6	2	Direct drying	30	69
SonoCOF-H2	TAPT 49.2mg	TFPA 45.7 mg	6	2	Direct drying	50	83
SonoCOF-I2	TAPT 105 mg	TFP 177 mg	12	10	Direct drying	40	93
SonoCOF-J2	TAPT 35.4 mg	IDA 20.2 mg	6	2	Direct drying	50	83
SonoCOF-K2	TAPT 35.4 mg	PDA 202 mg	12	2	Hexane	50	81
SonoCOF-A3	PTTA 28.3 mg	DMTA 19.4 mg	6	2	Direct drying	50	63
SonoCOF-B3	PTTA 28.3 mg	TPA 13.4 mg	6	2	Direct drying	50	99
SonoCOF-C3	PTTA 22.7 mg	DHTA 13.3 mg	6	2	Direct drying	50	96
SonoCOF-D3	PTTA 67.1 mg	TFTA 49.5 mg	12	2	Hexane	25	86
SonoCOF-E3	PTTA 22.7 mg	BDCA 16.8 mg	12	2	Hexane	50	95
SonoCOF-F3	PTTA 42.6 mg	TFB 16.2 mg	6	4	Direct drying	50	90
SonoCOF-G3	PTTA 34 mg	TFPT 31. mg	12	4	Direct drying	50	83
SonoCOF-H3	PTTA 34 mg	TFPA 26.4 mg	12	2	Direct drying	50	39
SonoCOF-J3	PTTA 56.6 mg	IDA 26.8 mg	6	2	Hexane	50	88
SonoCOF-K3	PTTA 54 mg	PDA 24.3 mg	6	4	Hexane	50	87

SonoCOF-A4	ETTA 47.1 mg	DMTA 46.5 mg	12	6	Hexane	25	45
SonoCOF-B4	ETTA 58.8mg	TPA 40.2 mg	12	6	Hexane	25	37
SonoCOF-F4	ETTA35.4mg	TFB 29.1mg	12	2	Direct drying	50	86
SonoCOF-G4	ETTA 35.4mg	TFPT 47.1 mg	12	2	Direct drying	50	67
SonoCOF-J4	ETTA 39.3 mg	IDA 26.8 mg	12	2	Hexane	50	77
SonoCOF-K4	ETTA 15.7mg	PDA 10.8 mg	12	2	Hexane	50	67
SonoCOF-F5	DAB 162.1 mg	TFB 162.1 mg	6	20	Hexane	40	73
SonoCOF-I5	DAB 81 mg	TFP 105 mg	6	20	Hexane	40	67
SonoCOF-F6	BD 138 mg	TFB 81 mg	12	10	Hexane	25	93
SonoCOF-G6	BD 29.4 mg	TFPT 31.4 mg	12	4	Hexane	25	61
SonoCOF-I6	BD 27.6 mg	TFP 21 mg	12	2	Direct drying	50	66
SonoCOF-A7	TAPA 232 mg	DMTA 233 mg	12	20	Hexane	40	64
SonoCOF-B7	TAPA 29 mg	TPA 20.2 mg	12	2	scCO_2 drying	50	55
SonoCOF-F7	TAPA 290 mg	TFB 162 mg	6	20	Hexane	40	90
SonoCOF-G7	TAPA 17.4 mg	TFPT 23.6 mg	6	2	Direct drying	50	68
SonoCOF-H7	TAPA 29 mg	TFPA 33 mg	12	2	Hexane	50	32
SonoCOF-F8	HH 46.4 mg	TFB 97.5 mg	12	6	Hexane	25	70
SonoCOF-G8	HH 8.3 mg	TFPT 39.4 mg	12	4	Hexane	25	32
SonoCOF-I8	HH 41 mg	TFP 105 mg	12	10	Hexane	25	83
SonoCOF-I9	DBDA 21.5 mg	TFP 21 mg	12	2	Hexane	50	79
SonoCOF-I10	DCDA 21.7 mg	TFP 16.8 mg	6	2	Hexane	50	65
SonoCOF-A11	TAPD 47.3 mg	DMTA 38.8 mg	12	4	Direct drying	50	67
SonoCOF-B11	TAPD 28.4 mg	TPA 24.4 mg	12	2	scCO_2 drying	50	70
SonoCOF-E11	TAPD 142 mg	BDCA 126 mg	12	10	scCO_2 drying	25	63
SonoCOF-F11	TAPD 47.3 mg	TFB 21.6 mg	12	4	Direct drying	50	65
SonoCOF-G11	TAPD 14.2 mg	TFPT 15.7 mg	12	2	Hexane	50	63
SonoCOF-J11	TAPD 23.6 mg	IDA 13.4 mg	12	2	Hexane	50	53
SonoCOF-K11	TAPD 3.6 mg	PDA 13.5 mg	12	2	Hexane	50	99

3. Characterization of COFs

3.1 Elemental Analysis

Table S2. Elemental analysis of sonoCOFs.

Sample	C (wt%)		H (wt%)		N (wt%)	
	Calc.	Expt.	Calc.	Expt.	Calc.	Expt.
SonoCOF-A1	79.57	78.42	5.14	5.15	7.14	6.94
SonoCOF-B1	86.72	84.07	4.85	5.09	8.43	7.93
SonoCOF-C1	79.11	76.82	4.43	4.74	7.69	7.03
SonoCOF-D1	71.29	69.80	2.99	3.26	6.93	6.22
SonoCOF-E1	88.21	86.02	4.94	5.13	6.86	6.11
SonoCOF-F1	86.25	83.63	4.61	4.44	9.14	8.53
SonoCOF-G1	83.46	78.25	4.38	4.67	12.17	10.53
SonoCOF-H1	86.24	81.69	4.82	5.10	8.94	7.90
SonoCOF-I1	78.09	69.47	4.17	4.34	8.28	6.17
SonoCOF-J1	86.72	83.05	4.85	5.17	8.43	7.57
SonoCOF-K1	82.86	78.04	4.54	4.83	12.60	10.99
SonoCOF-A2	73.08	71.73	4.60	4.66	14.20	13.19
SonoCOF-B2	79.02	75.91	4.22	4.46	16.76	15.56
SonoCOF-C2	72.12	70.97	3.85	4.06	15.29	14.55
SonoCOF-D2	65.03	65.50	2.48	2.97	13.79	13.32
SonoCOF-E2	81.93	79.23	4.42	4.58	13.65	13.02
SonoCOF-F2	77.91	76.39	3.92	4.23	18.17	16.61
SonoCOF-G2	77.91	75.70	3.92	4.27	18.17	16.63
SonoCOF-H2	80.11	79.04	4.32	4.45	15.57	14.88
SonoCOF-I2	70.58	65.63	3.55	3.77	16.46	14.23
SonoCOF-J2	79.02	76.11	4.22	4.28	16.76	14.40
SonoCOF-K2	75.21	70.49	3.91	4.57	20.88	19.95
SonoCOF-A3	81.61	75.65	4.79	4.88	6.35	5.46
SonoCOF-B3	88.16	85.49	4.49	4.61	7.34	6.78
SonoCOF-C3	81.34	76.79	4.14	4.94	6.78	5.71
SonoCOF-D3	74.17	70.98	2.89	3.02	6.18	5.54
SonoCOF-E3	89.25	83.38	4.63	5.01	6.12	5.39
SonoCOF-F3	87.86	79.82	4.25	4.82	7.88	7.85
SonoCOF-G3	84.85	79.91	4.15	4.46	10.99	9.87
SonoCOF-H3	87.47	80.45	4.53	5.08	8.00	6.46
SonoCOF-J3	88.16	82.32	4.49	4.75	7.34	6.24
SonoCOF-K3	84.80	79.93	4.22	4.76	10.99	9.36
SonoCOF-A4	77.95	74.94	5.12	5.36	7.90	6.58

SonoCOF-B4	85.69	81.67	4.79	5.08	9.52	8.82
SonoCOF-F4	85.05	79.70	4.51	4.41	10.44	9.10
SonoCOF-G4	82.44	78.05	4.29	4.39	13.26	11.78
SonoCOF-J4	85.69	81.82	4.79	4.89	9.52	8.62
SonoCOF-K4	81.34	77.54	4.44	4.69	14.23	12.77
SonoCOF-F5	79.98	75.45	4.47	4.53	15.55	13.70
SonoCOF-I5	67.92	59.85	3.80	4.47	13.20	9.00
SonoCOF-F6	84.35	80.41	4.72	4.90	10.93	8.78
SonoCOF-I6	74.99	69.56	4.20	4.33	9.72	7.94
SonoCOF-A7	75.13	72.44	5.16	5.13	10.62	10.04
SonoCOF-B7	82.36	78.83	4.84	5.21	12.81	11.43
SonoCOF-F7	81.39	78.78	4.55	4.90	14.06	12.45
SonoCOF-G7	80.11	79.30	4.32	4.71	15.57	13.79
SonoCOF-H7	82.8	78.67	4.81	5.10	12.38	10.02
SonoCOF-F8	69.22	67.00	3.87	4.55	26.91	23.59
SonoCOF-G8	74.40	71.87	3.90	4.02	21.69	19.76
SonoCOF-I8	52.95	51.56	2.96	3.13	20.58	13.17
SonoCOF-I9	59.99	64.17	5.03	4.84	11.66	9.14
SonoCOF-I110	51.28	48.38	2.15	2.57	9.97	9.33
SonoCOF-A11	76.12	72.73	5.11	5.07	10.65	10.15
SonoCOF-B11	82.61	77.25	4.82	5.02	12.57	12.12
SonoCOF-F11	81.80	75.52	4.58	4.87	13.63	11.35
SonoCOF-G11	80.50	76.09	4.36	5.17	15.14	12.32
SonoCOF-J11	82.61	75.49	4.82	12.57	14.98	12.12
SonoCOF-K11	78.79	73.92	4.51	4.63	16.71	14.70

Calc. = calculated data, assuming idealized stoichiometry, infinite frameworks, and no physisorption of guests (*e.g.*, N_2, H_2O in the COF pores); note that none of these assumptions are satisfied in the real materials, and hence elemental analyses for these porous solids can differ significantly from the idealized, calculated values; Expt. = experimental results.

3.2 Fourier-transform infrared spectroscopy

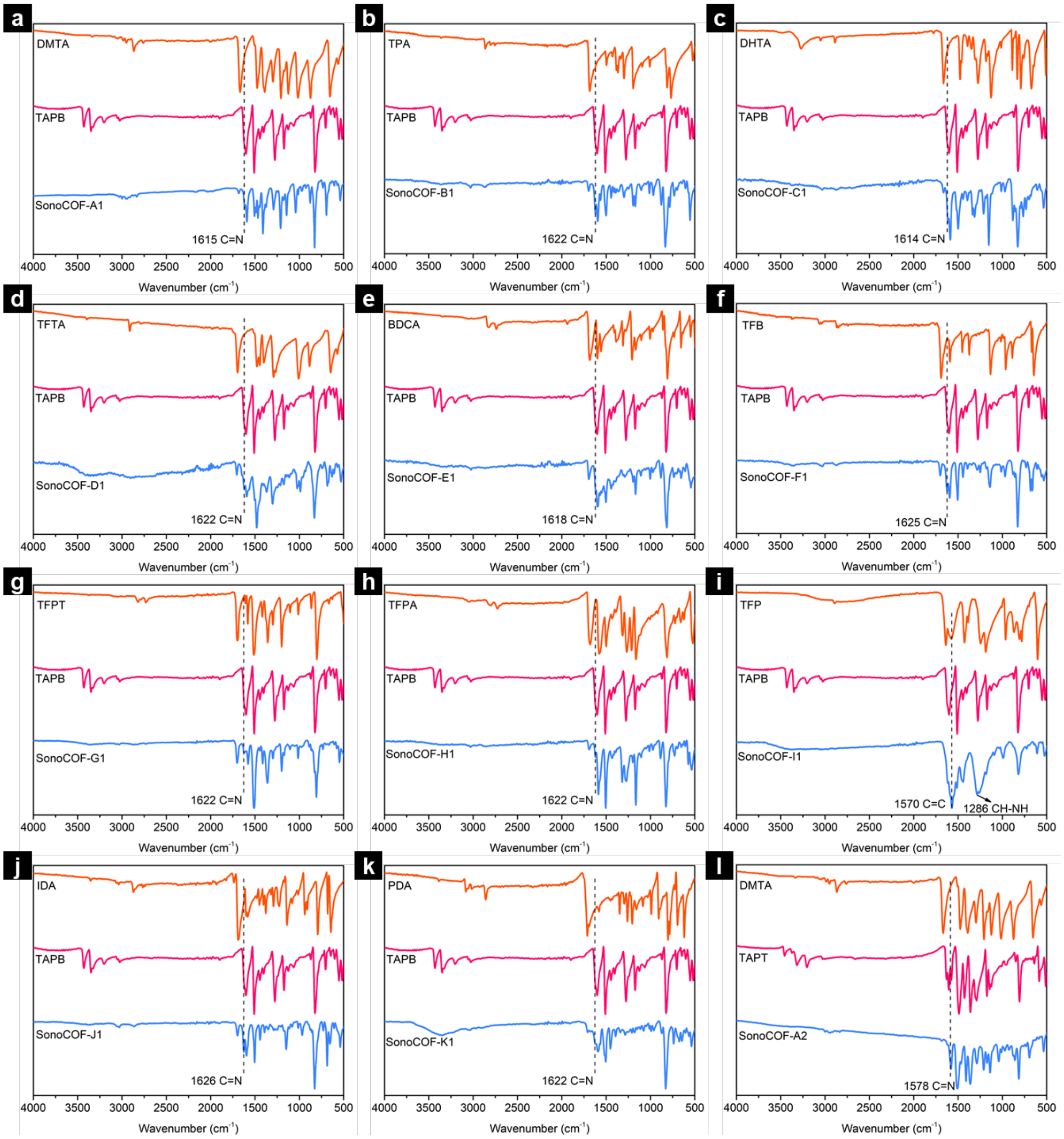

Figure S1. FT-IR spectra of sonoCOFs A1-K1 and sonoCOF-A2 compared with corresponding starting materials.

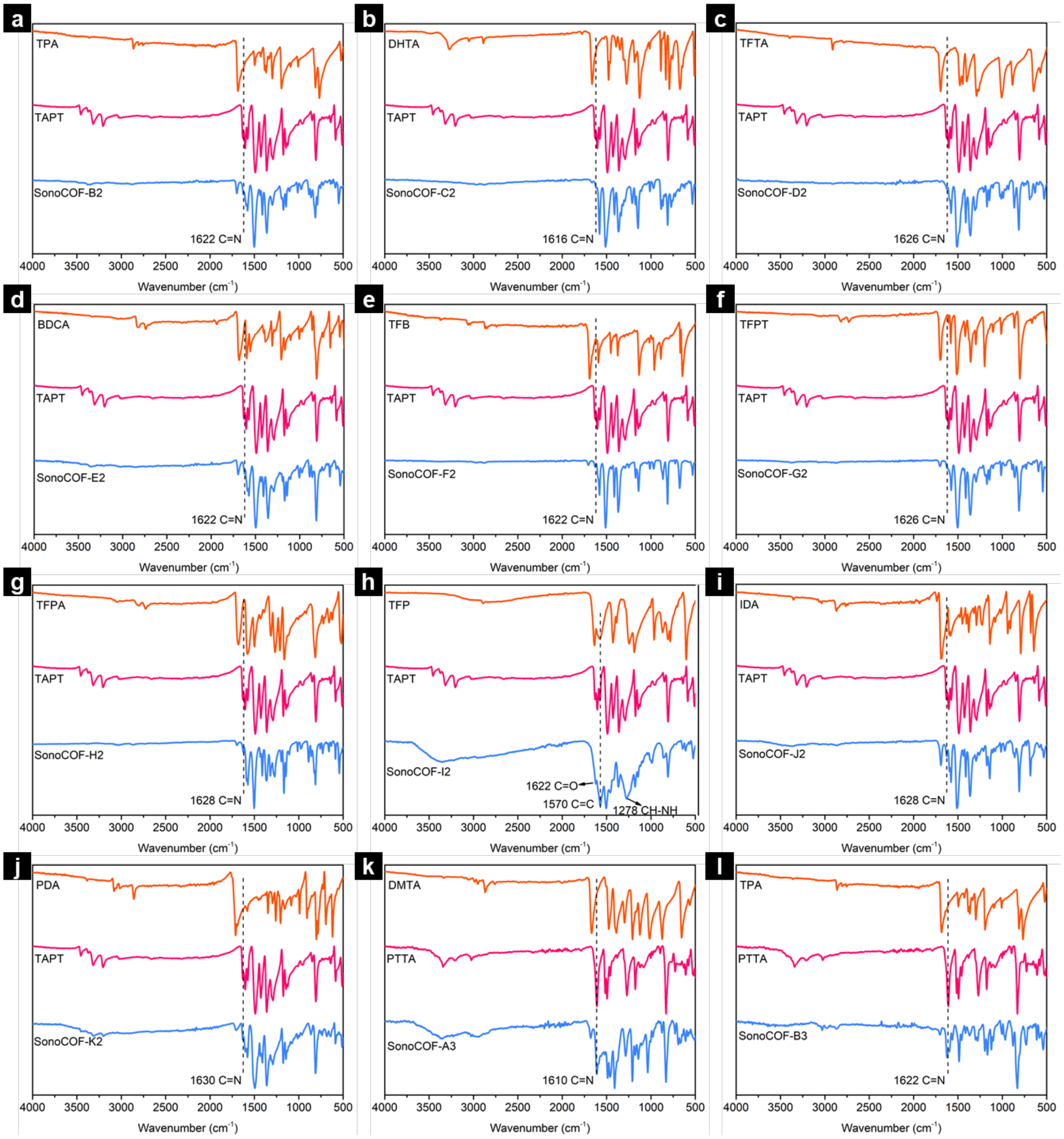

Figure S2. FT-IR spectra of sonoCOFs B2-K2 and sonoCOF-A3 and -B3 compared with corresponding starting materials.

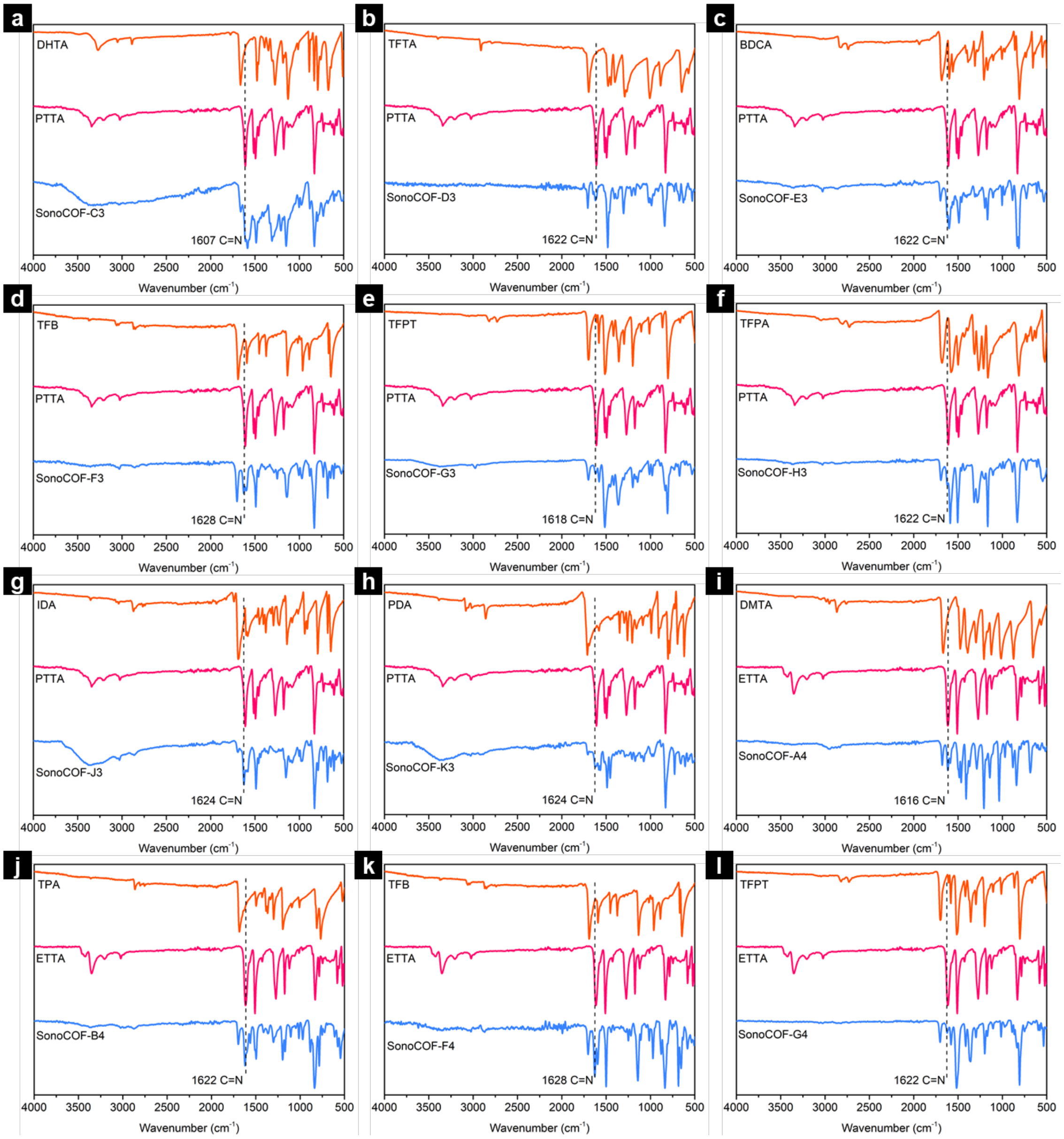

Figure S3. FT-IR spectra of sonoCOF-C3, -D3, -E3, -F3, -G3, -H3, -J3, -K3, -A4, -B4, -F4 and -G4 compared with corresponding starting materials.

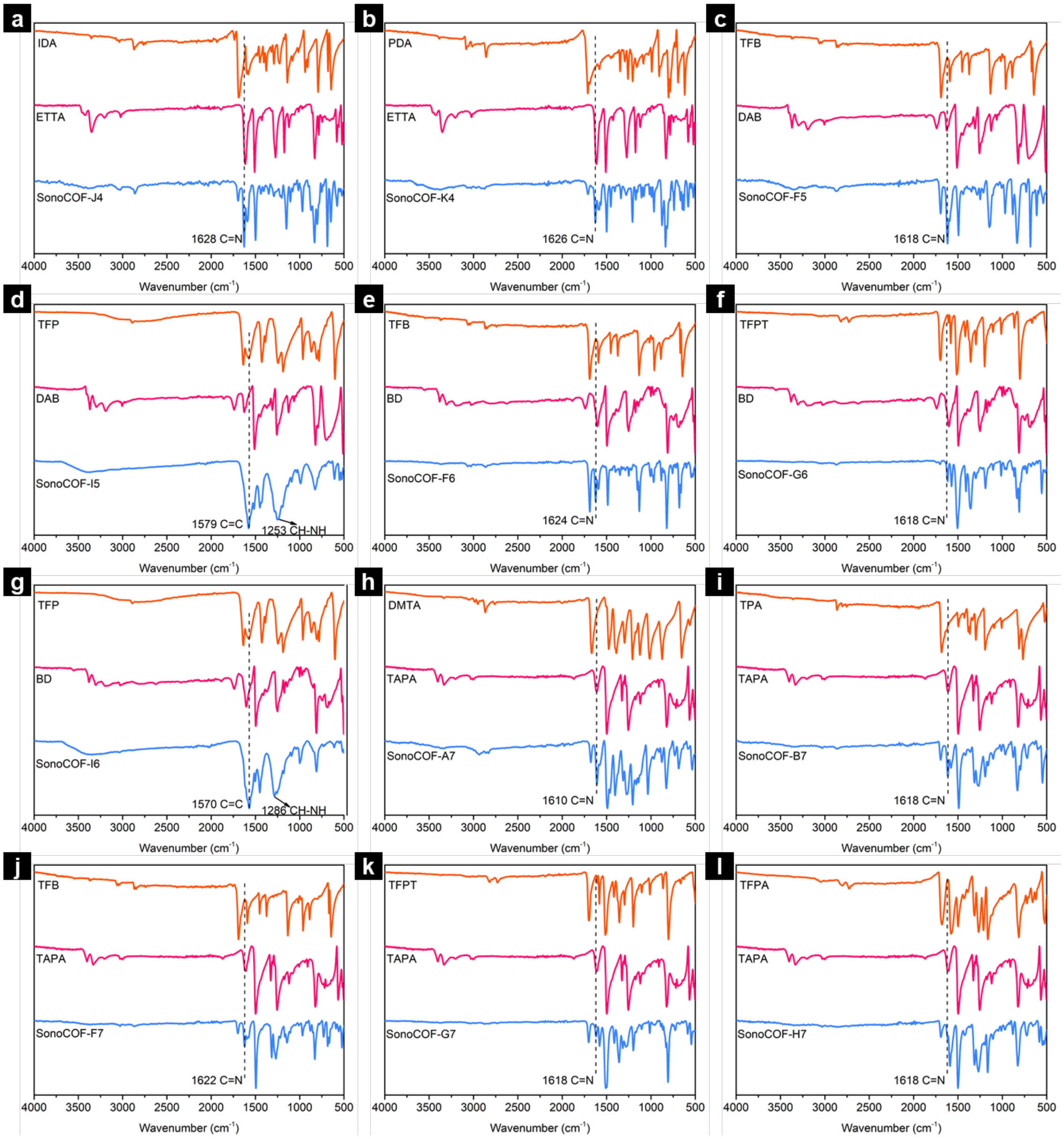

Figure S4. FT-IR spectra of sonoCOF-J4, -K4, -F5, -I5, -F6, -G6, -I6, -A7, -B7, -F7, -G7 and -H7 compared with corresponding starting materials.

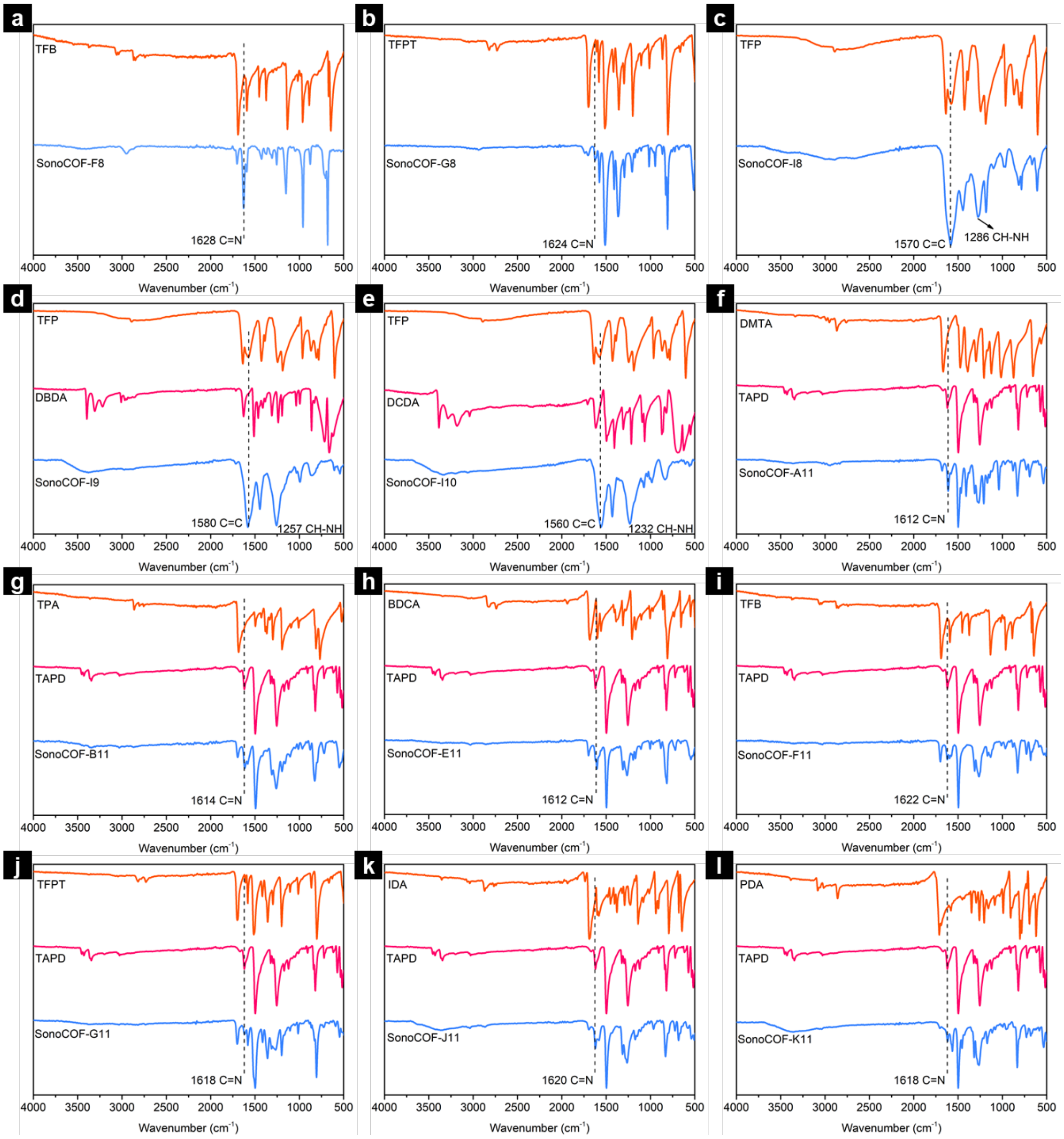

Figure S5. FT-IR spectra of sonoCOF-F8, -G8, -I8, -I9, -I10, -A11, -B11, -E11, -F11, -G11, -J11 and -K11 compared with corresponding starting materials.

3.3 Powder X-ray diffraction analysis

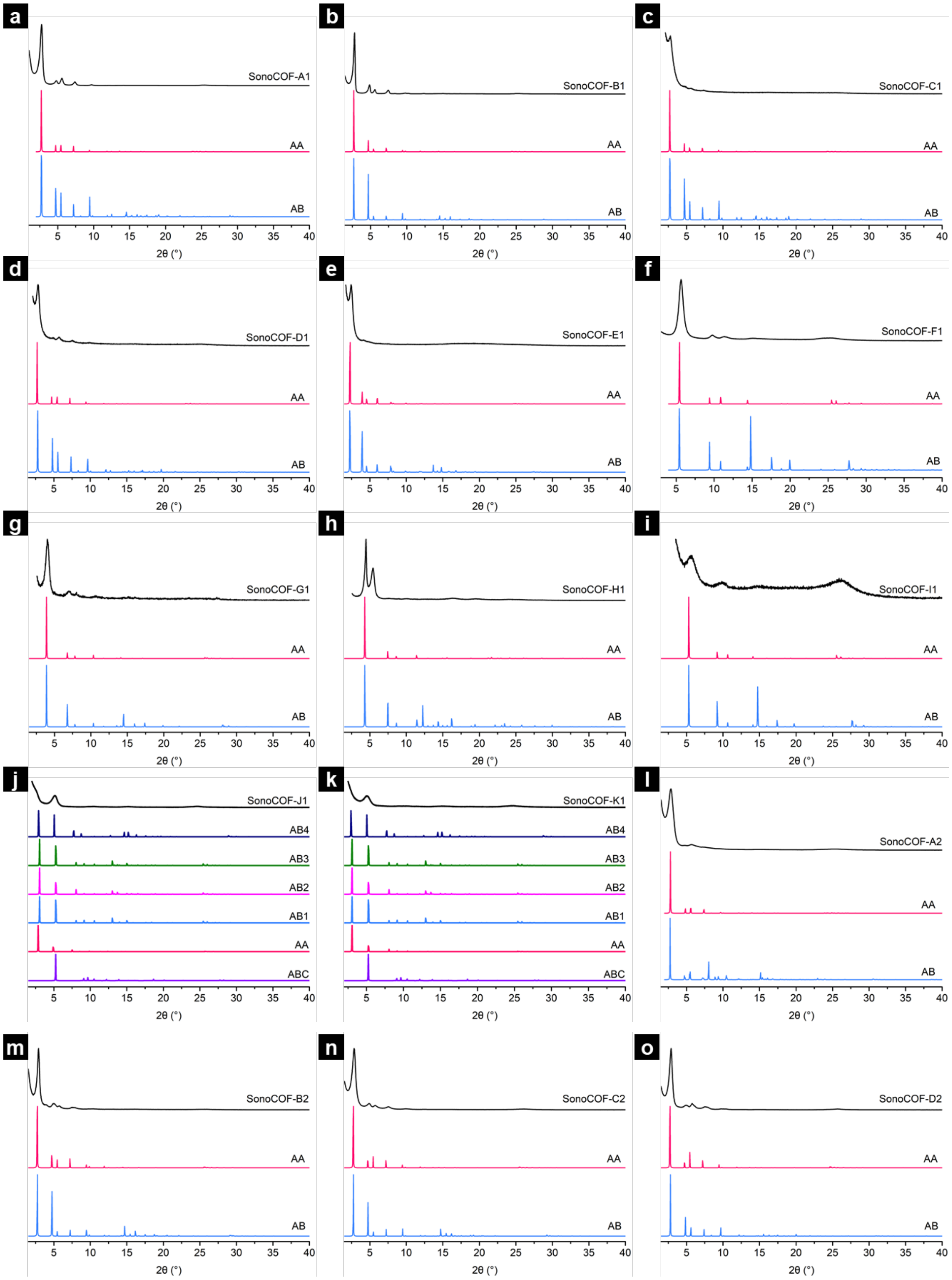

Figure S6. Observed PXRD patterns of sonoCOF-A1, -B1, -C1, -D1, -E1, -F1, -G1, -H1, -I1, -J1, -K1, -A2, -B2, -C2 and -D2 with calculated models.

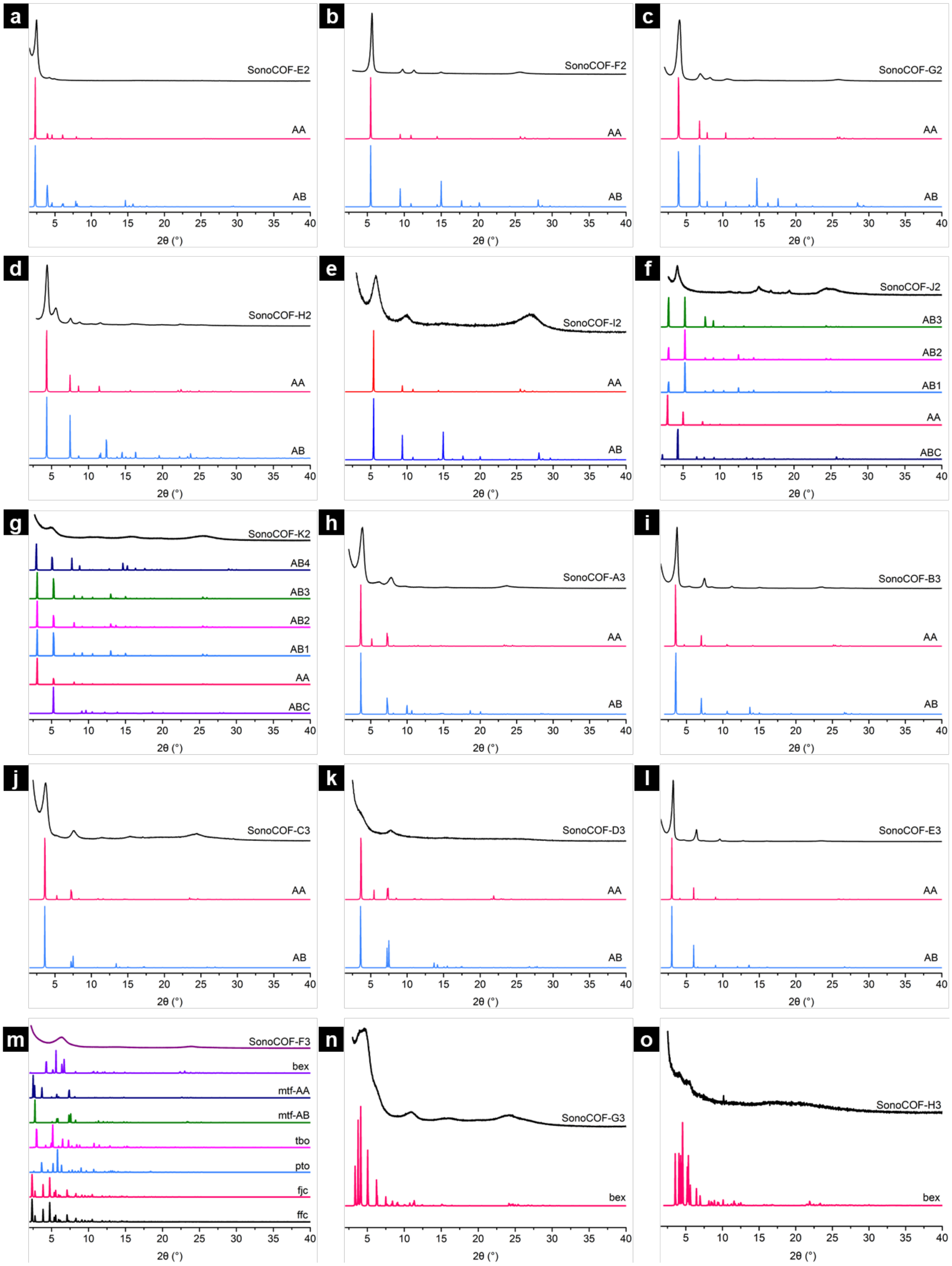

Figure S7. Observed PXRD patterns of sonoCOF-E2, -F2, -G2, -H2, -I2, -J2, -K2, -A3, -B3, -C3, -D3, -E3, -F3, -G3 and -H3 with calculated models.

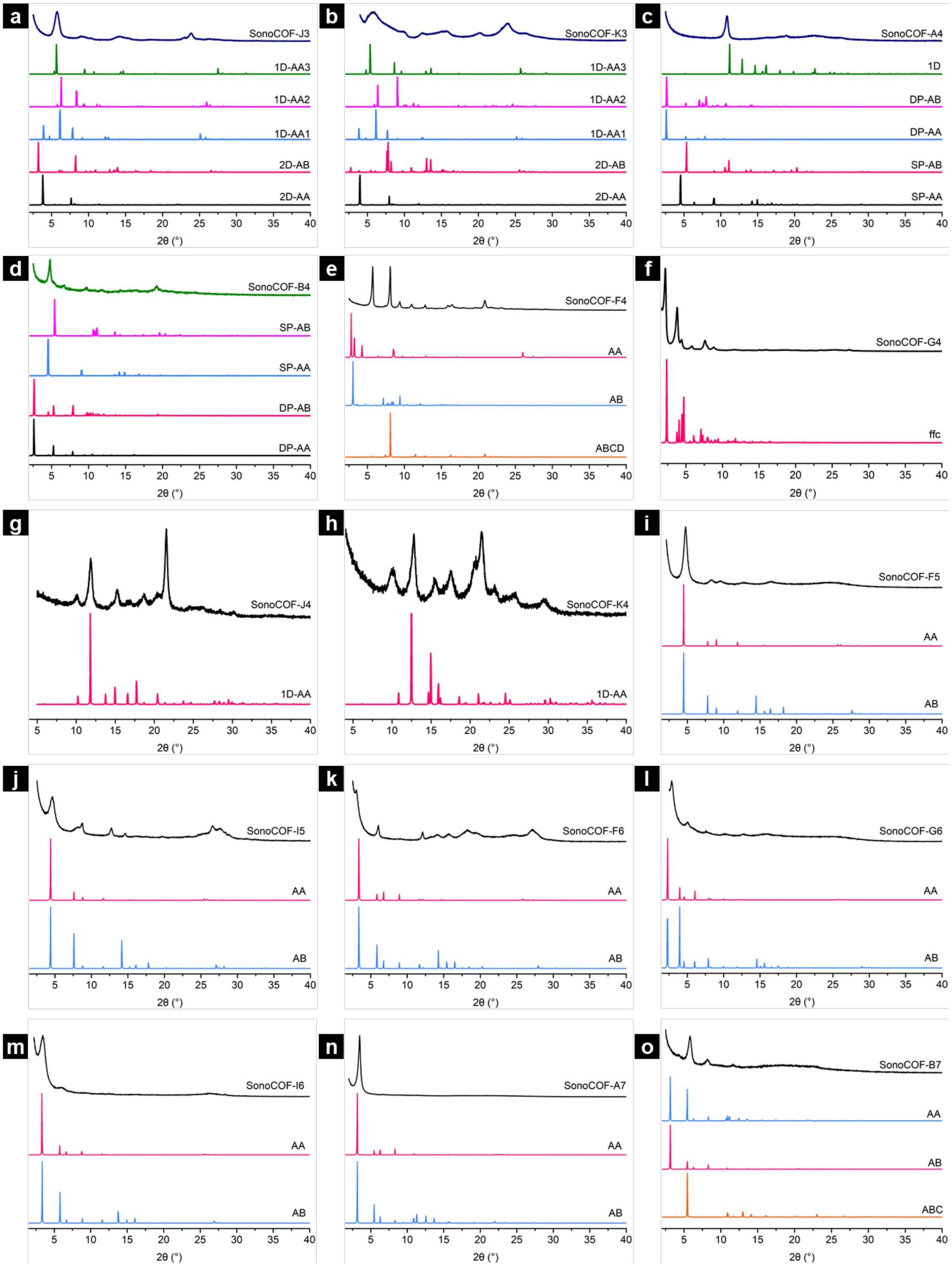

Figure S8. Observed PXRD patterns of sonoCOF-J3, -K3, -A4, -B4, -F4, -G4, -J4, -K4, -F5, -I5, -F6, -G6, -I6, -A7 and -B7 with calculated models.

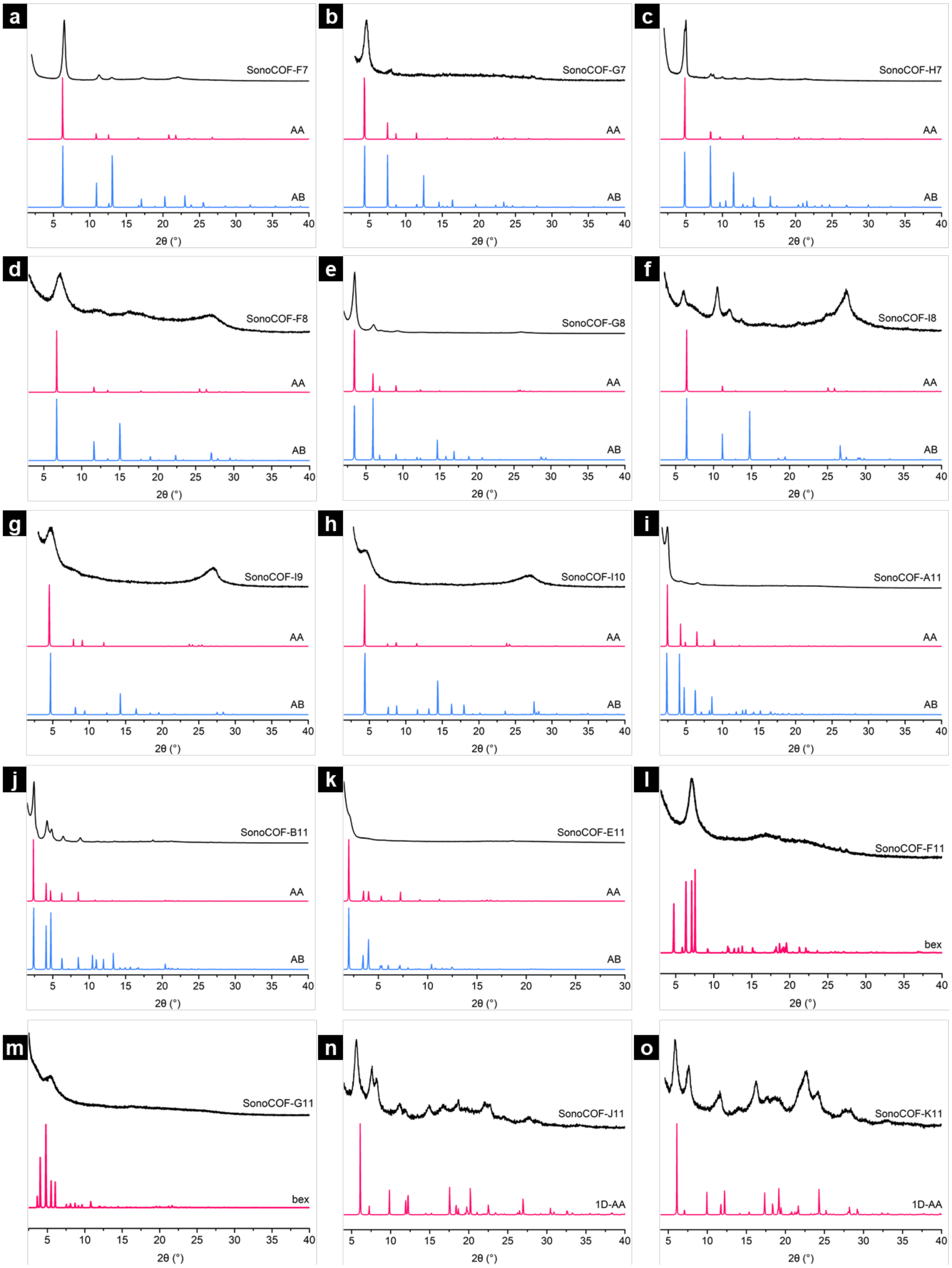

Figure S9. Observed PXRD patterns of sonoCOF-F7, -G7, -H7, -F8, -G8, -I8, -I9, -I10, -A11, -B11, -E11, -F11, -G11, -J11 and -K11 with calculated models.

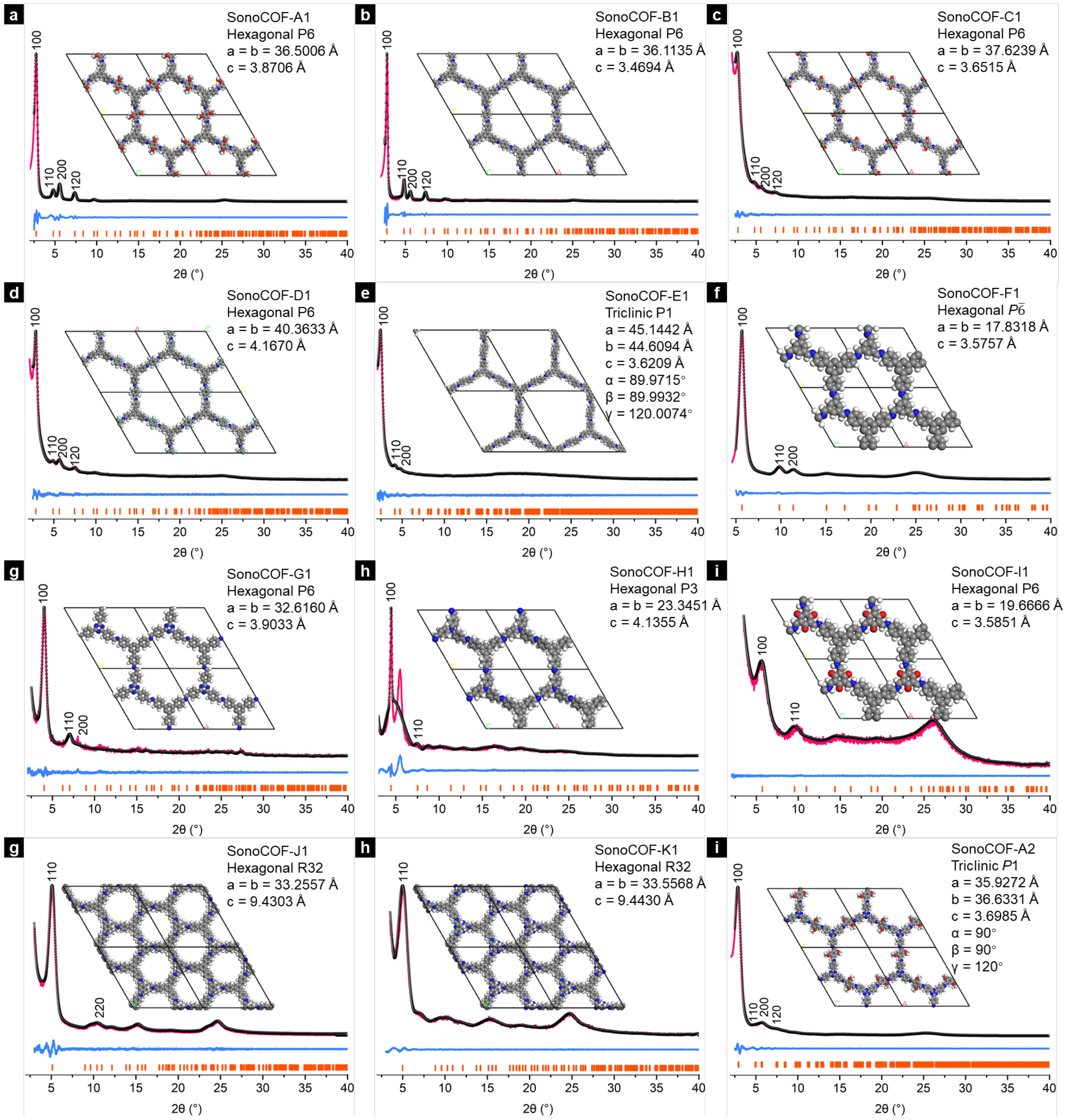

Figure S10. Pawley refinements against the PXRD patterns of sonoCOF-A1, B1, C1, D1, E1, F1, G1, H1, I1, J1, K1 and A2, respectively. Pink lines: y_{obs} (experimental PXRD data). Black dots: y_{calc} (Pawley refinement profile). Blue lines: $y_{obs} - y_{calc}$ (residual); yellow marks, *hkl* positions calculated for that phase. Insets: modelled crystal structures. C, grey; H, white; N, blue; O, red.

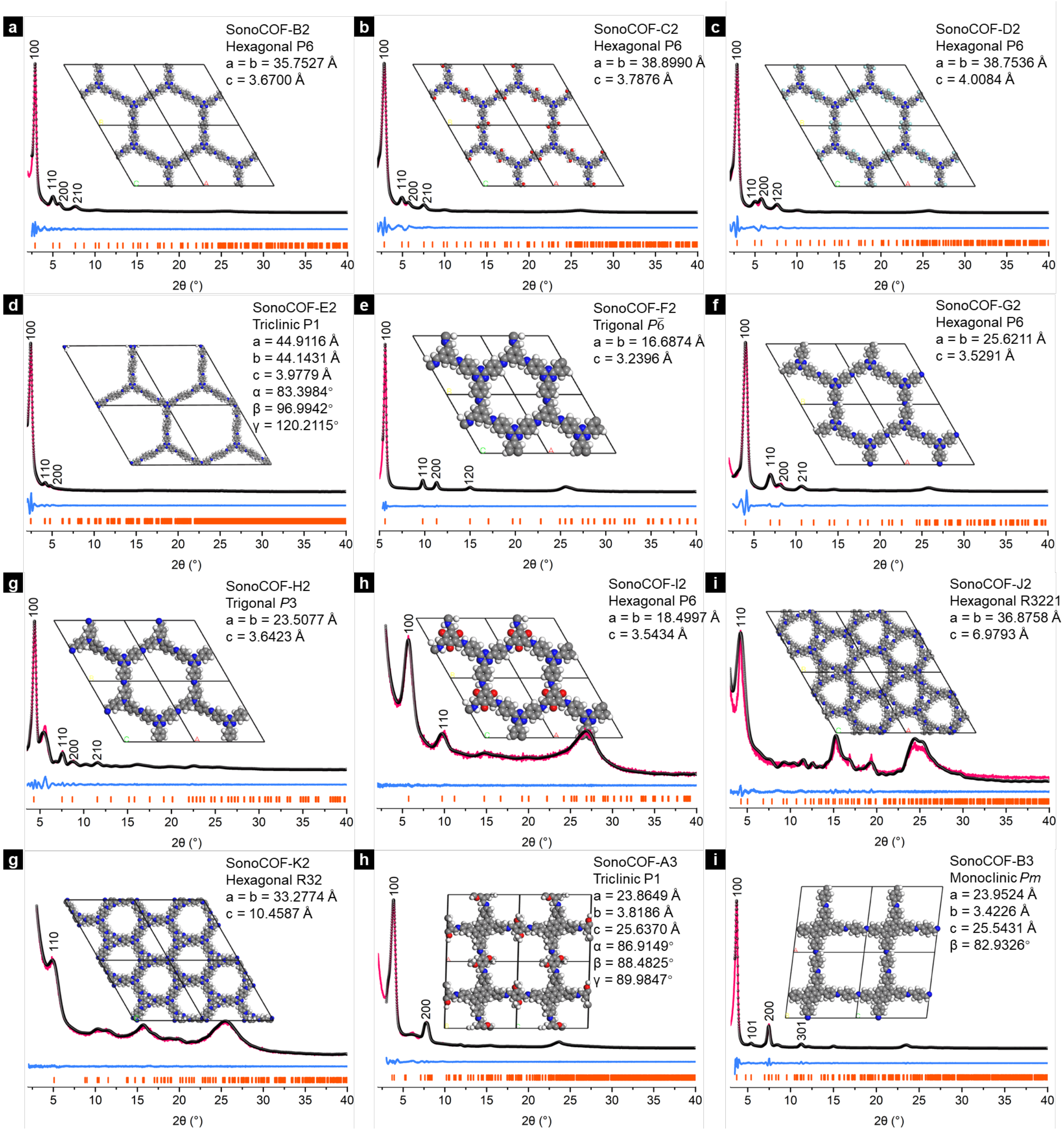

Figure S11. Pawley refinements against the PXRD patterns of sonoCOF-B2, C2, D2, E2, F2, G2, H2, I2, J2, K2, A3 and B3, respectively. Pink lines: y_{obs} (experimental PXRD data). Black dots: y_{calc} (Pawley refinement profile). Blue lines: $y_{obs} - y_{calc}$ (residual); yellow marks, *hkl* positions calculated for that phase. Insets: modelled crystal structures. C, grey; H, white; N, blue; O, red.

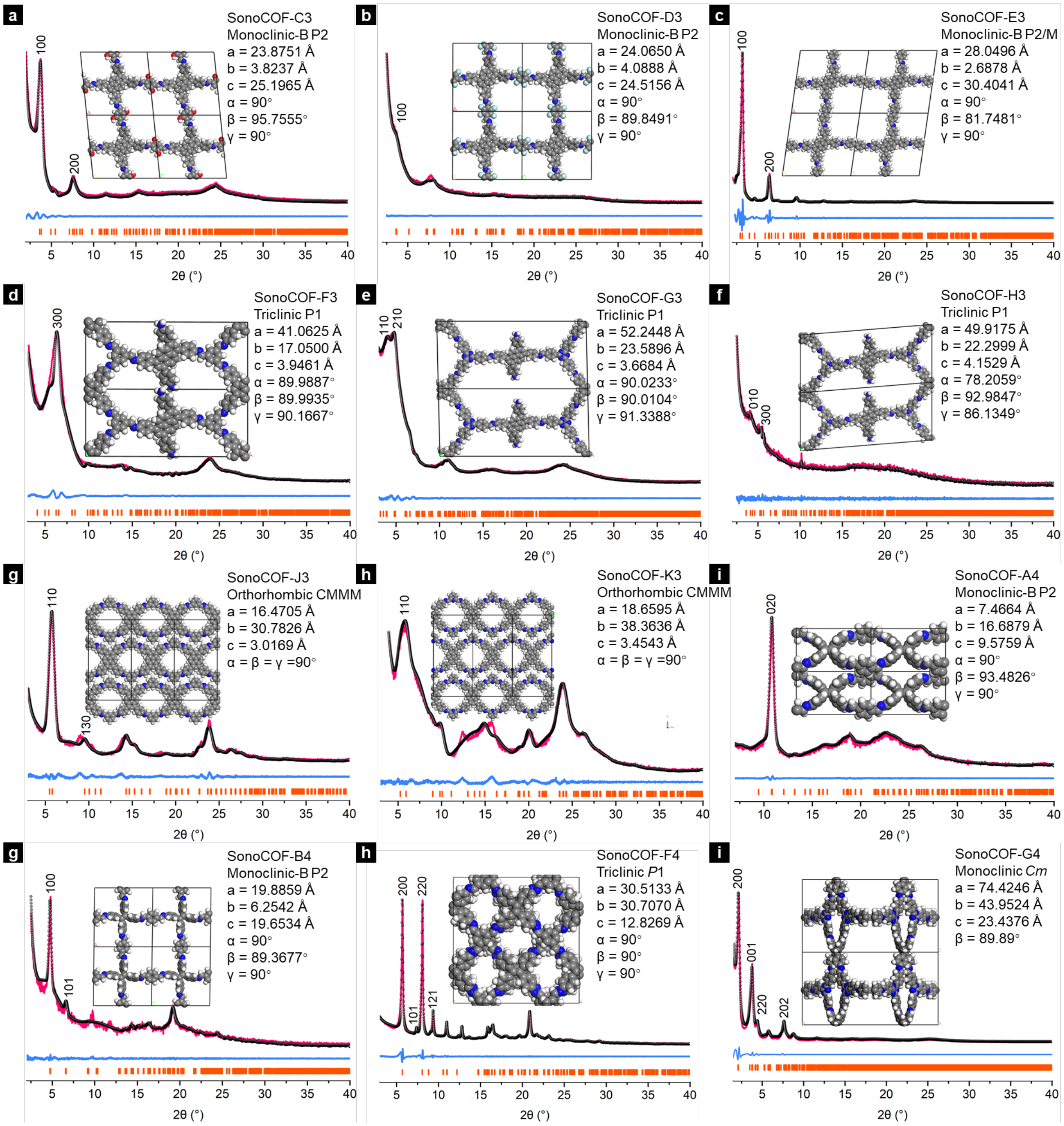

Figure S12. Pawley refinements against the PXRD patterns of sonoCOF-C3, D3, E3, F3, G3, H3, J3, K3, A4, B4, F4 and G4, respectively. Pink lines: y_{obs} (experimental PXRD data). Black dots: y_{calc} (Pawley refinement profile). Blue lines: $y_{obs} - y_{calc}$ (residual); yellow marks, *hkl* positions calculated for that phase. Insets: modelled crystal structures. C, grey; H, white; N, blue; O, red.

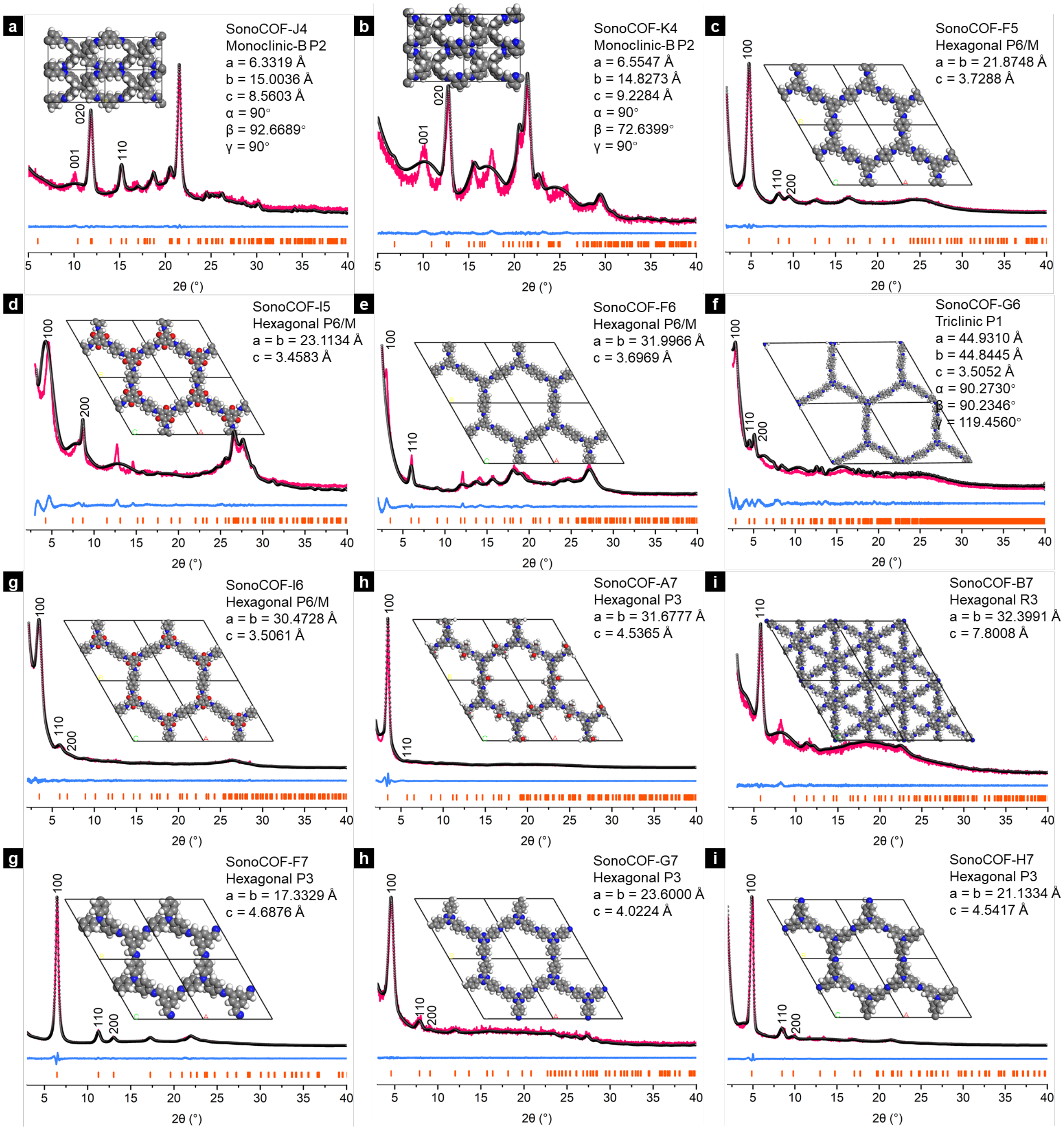

Figure S13. Pawley refinements against the PXRD patterns of sonoCOF-J4, K4, F5, I5, F6, G6, I6, A7, B7, F7, G7 and H7, respectively. Pink lines: y_{obs} (experimental PXRD data). Black dots: y_{calc} (Pawley refinement profile). Blue lines: $y_{obs} - y_{calc}$ (residual); yellow marks, *hkl* positions calculated for that phase. Insets: modelled crystal structures. C, grey; H, white; N, blue; O, red.

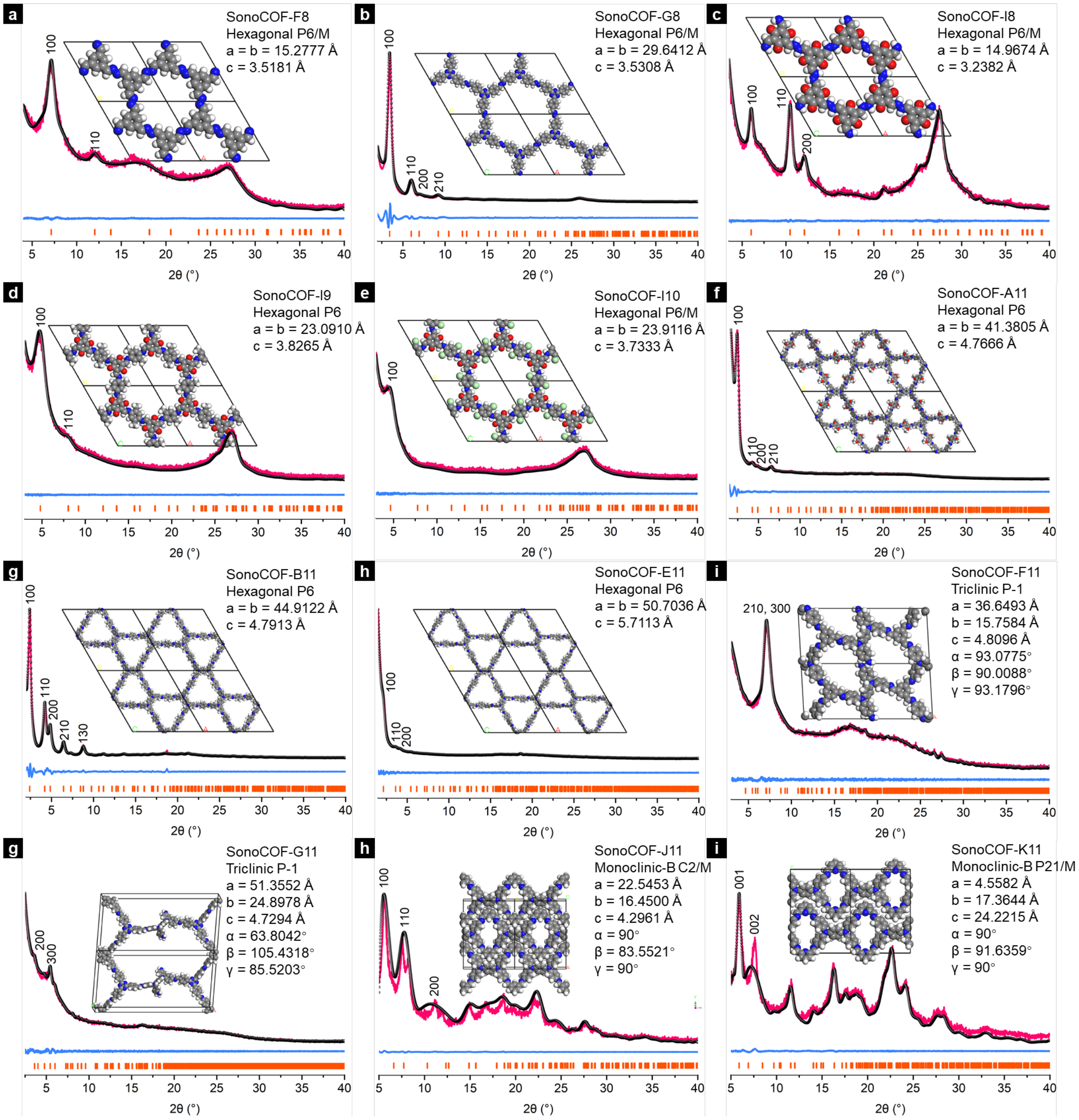

Figure S14. Pawley refinements against the PXRD patterns of sonoCOF-F8, G8, I8, I9, I10, A11, B11, E11, F11, G11, J11 and K11, respectively. Pink lines: y_{obs} (experimental PXRD data). Black dots: y_{calc} (Pawley refinement profile). Blue lines: $y_{obs} - y_{calc}$ (residual); yellow marks, *hkl* positions calculated for that phase. Insets: modelled crystal structures. C, grey; H, white; N, blue; O, red.

3.4 Thermogravimetric analysis

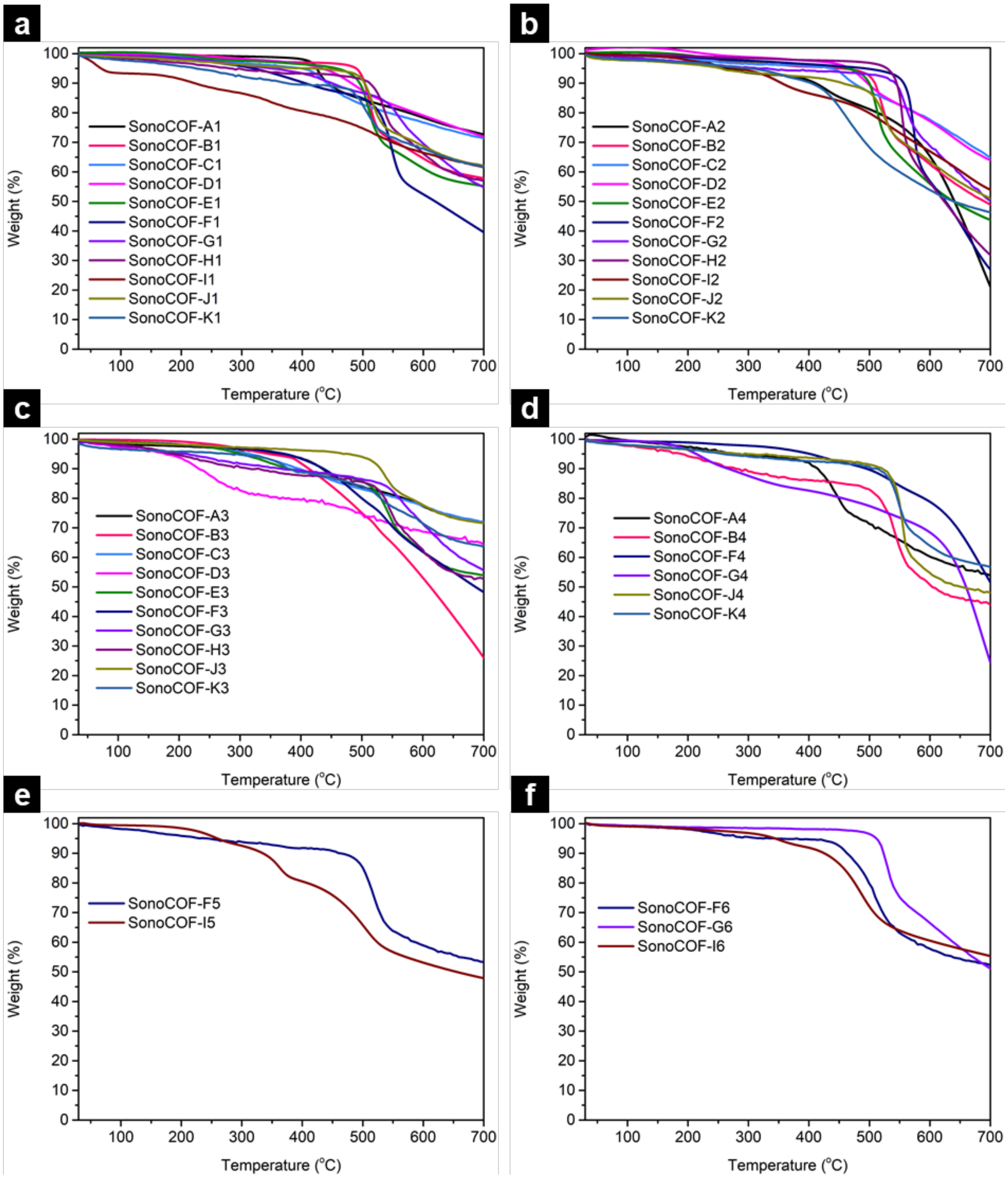

Figure S15. TGA data of (a) sonoCOF-A1, -B1, -C1, -D1, -E1, -F1, -G1, -H1, -I1, -J1 and -K1; (b) sonoCOF-A2, -B2, -C2, -D2, E2, -F2, -G2, -H2, -I2, -J2 and -K2; (c) sonoCOF-A3, -B3, -C3, -D3, -E3, -F3, -G3, -H3, -J3 and -K3; (d) sonoCOF-A4, -B4, -F4, -G4, -J4 and -K4; (e) sonoCOF-F5 and -I5; (f) sonoCOF-F6, -G6 and I6.

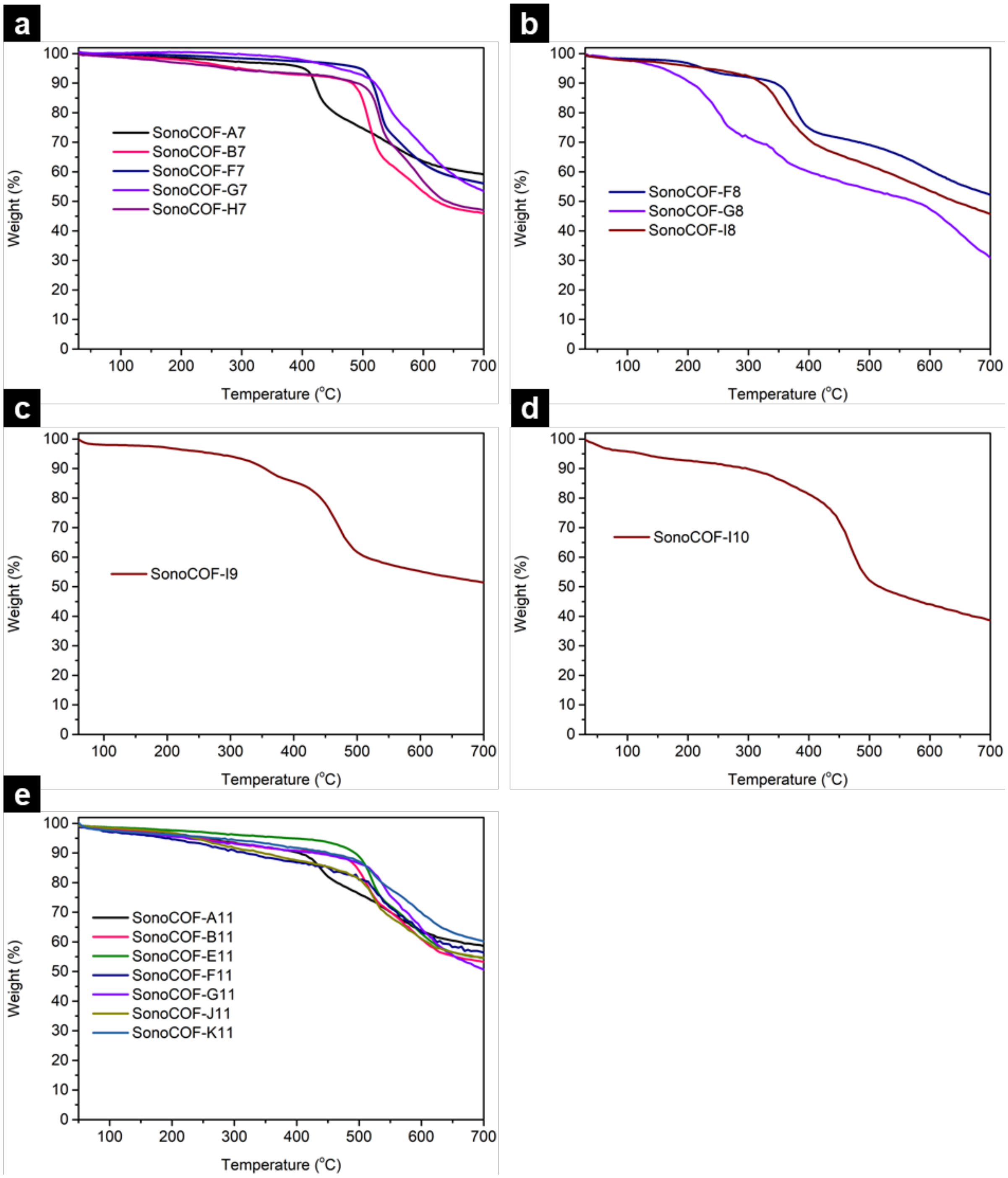

Figure S16. TGA data of (a) sonoCOF-A7, -B7, -F7, -G7 and -H7; (b) sonoCOF-F8, -G8 and -I8; (c) sonoCOF-I9; (d) sonoCOF-I10; (e) sonoCOF-A11, B11, E11, F11, G11, J11 and K11.

3.5 Scanning electron microscopy

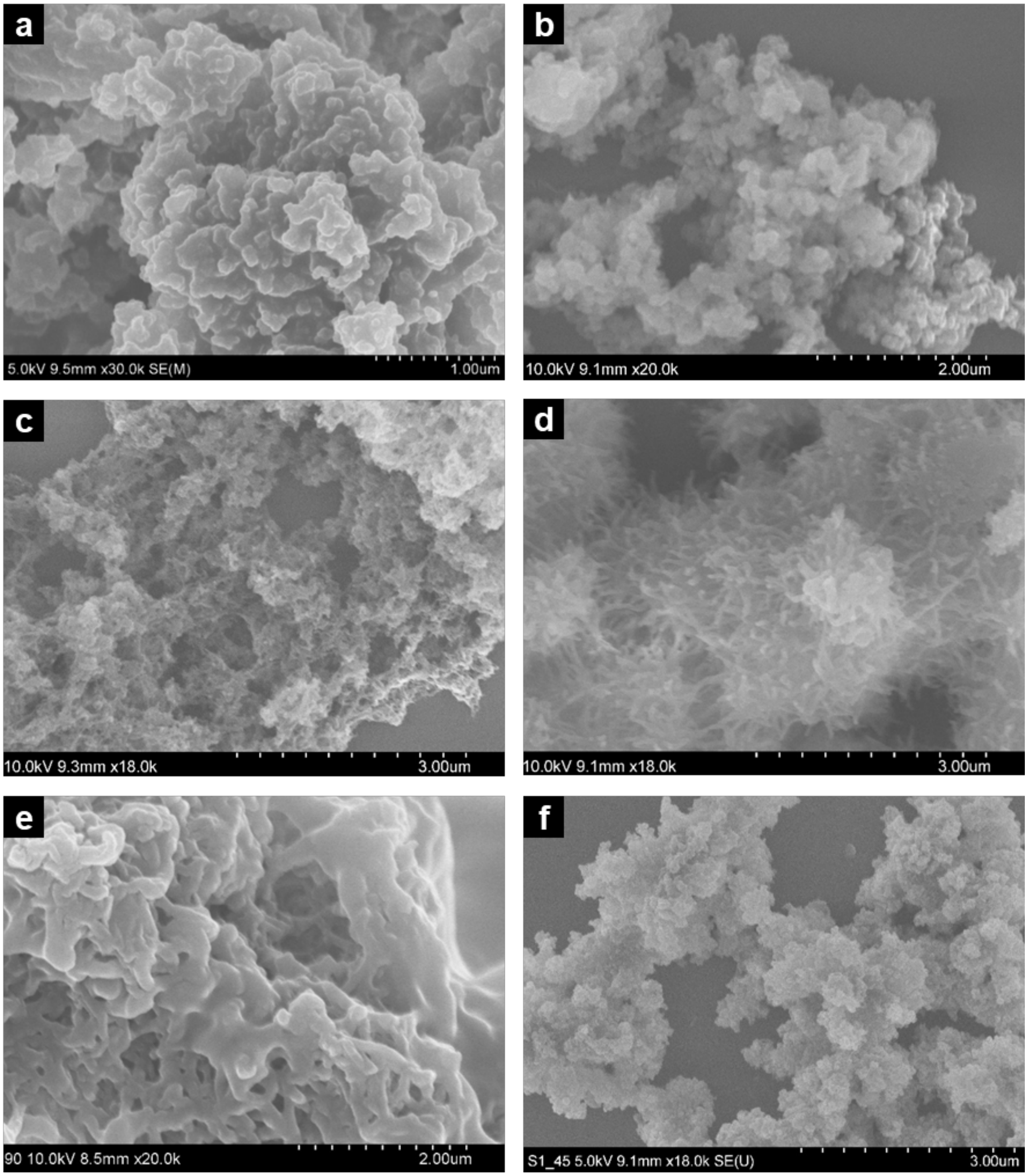

Figure S17. SEM images of (a) sonoCOF-A1, (b) sonoCOF-B1, (c) sonoCOF-C1, (d) sonoCOF-D1, (e) sonoCOF-E1 and (f) sonoCOF-F1.

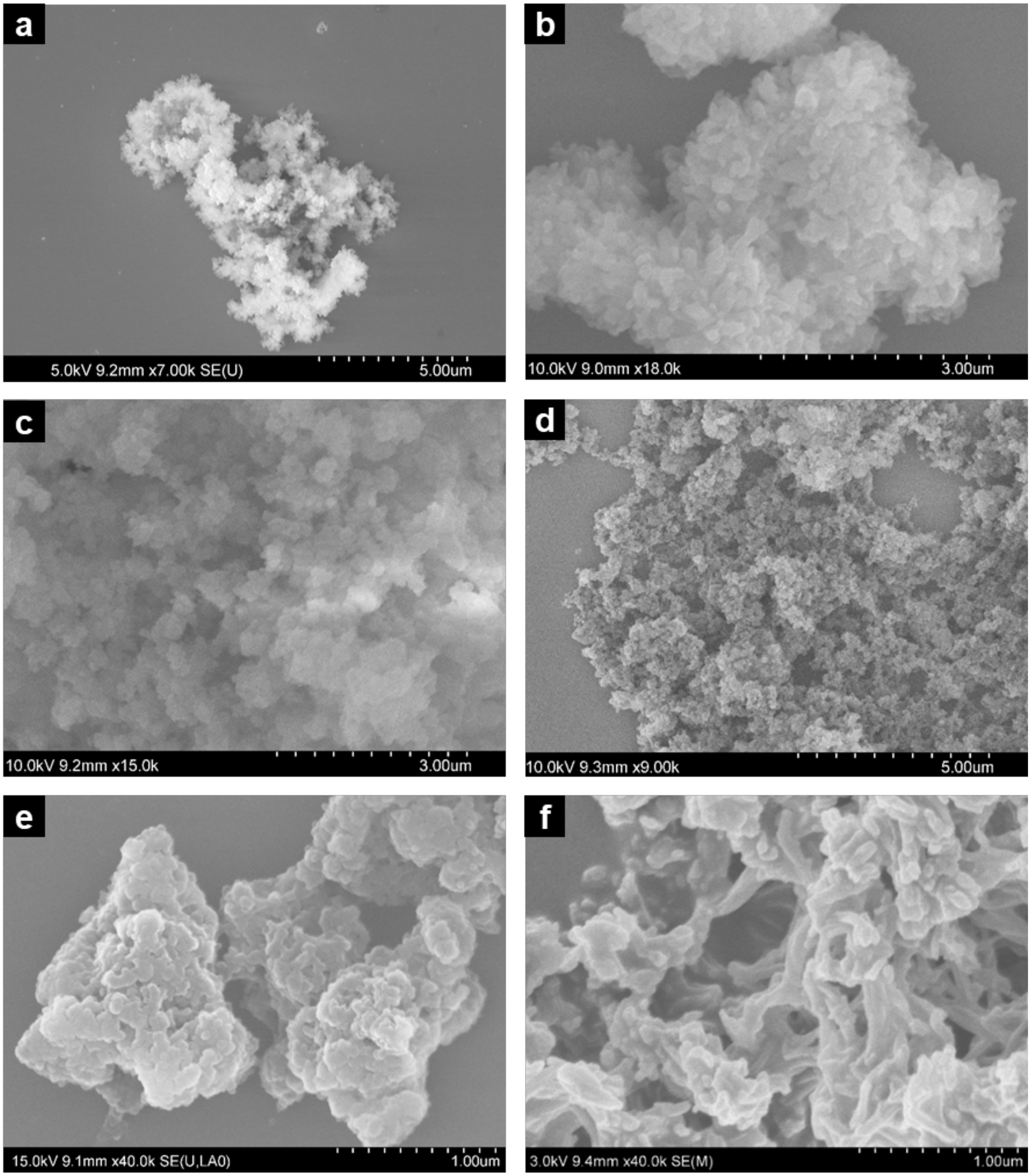

Figure S18. SEM images of (a) SonoCOF-G1, (b) sonoCOF-H1, (c) sonoCOF-I1, (d) sonoCOF-J1, (e) sonoCOF-K1 and (f) sonoCOF-A2.

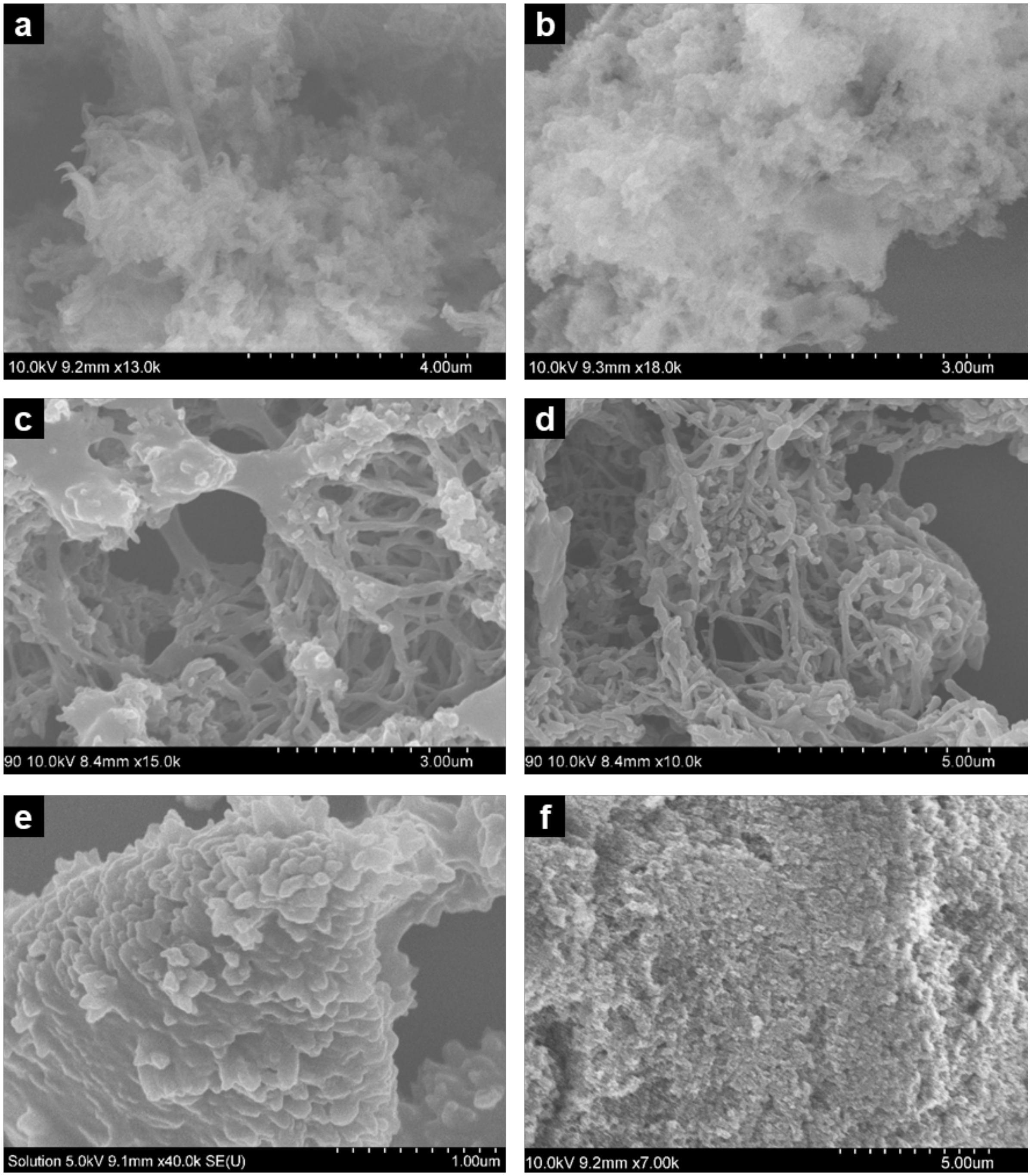

Figure S19. SEM images of (a) SonoCOF-B2, (b) sonoCOF-C2, (c) sonoCOF-D2, (d) sonoCOF-E2, (e) sonoCOF-F2 and (f) sonoCOF-G2.

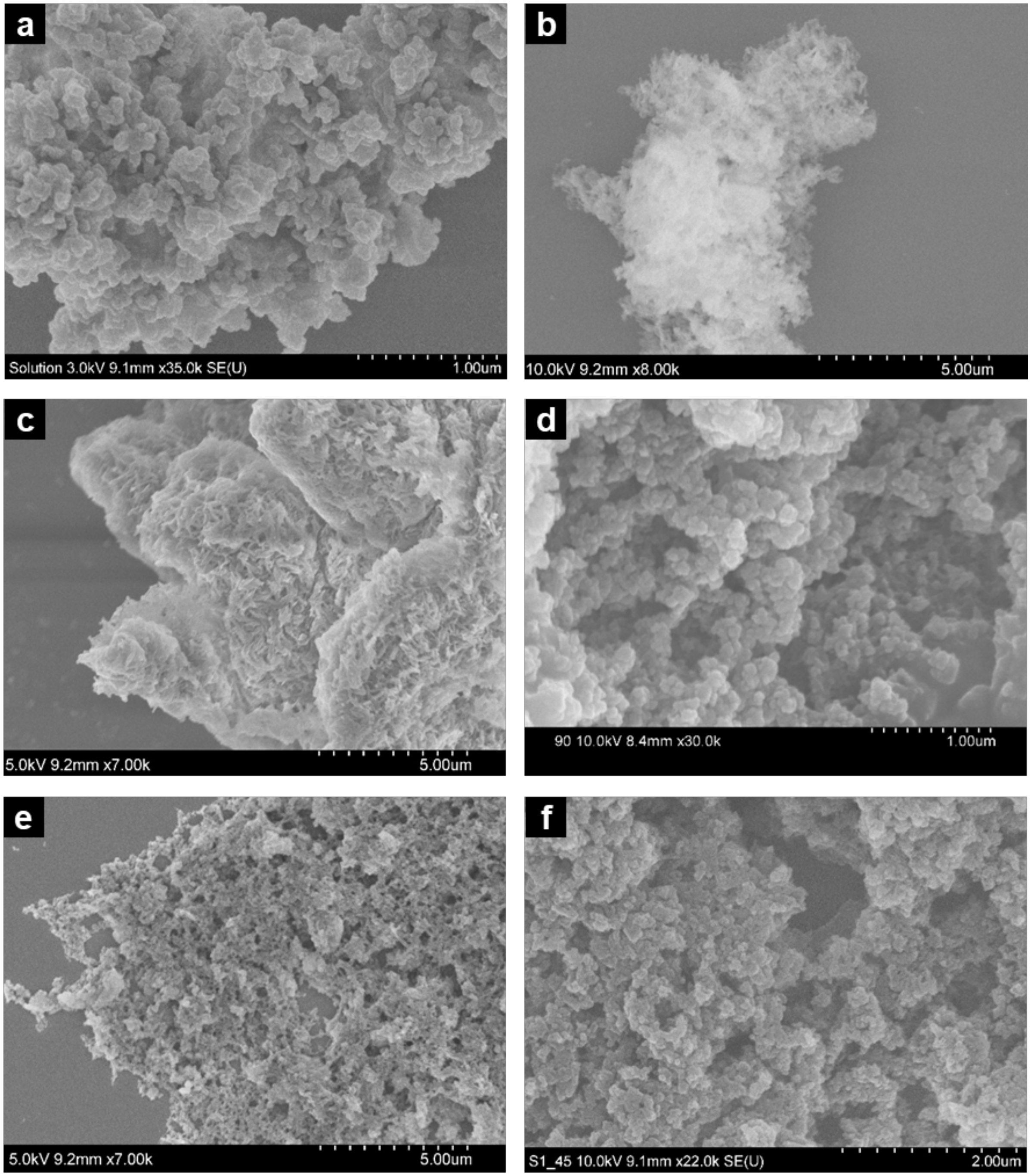

Figure S20. SEM images of (a) SonoCOF-H2, (b) sonoCOF-I2, (c) sonoCOF-J2, (d) sonoCOF-K2, (e) sonoCOF-A3 and (f) sonoCOF-B3.

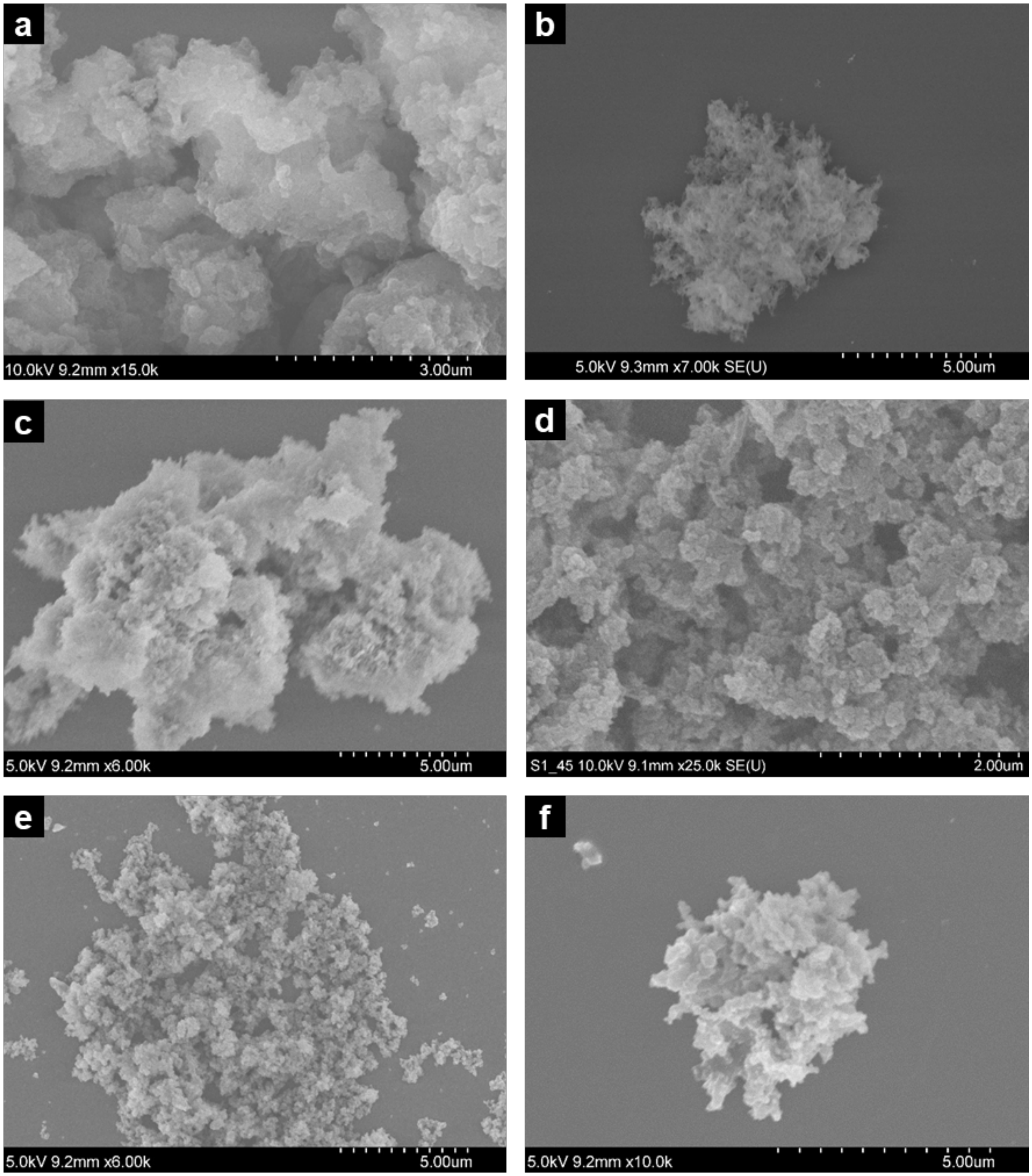

Figure S21. SEM images of (a) SonoCOF-C3, (b) sonoCOF-D3, (c) sonoCOF-E3, (d) sonoCOF-F3, (e) sonoCOF-G3 and (f) sonoCOF-H3.

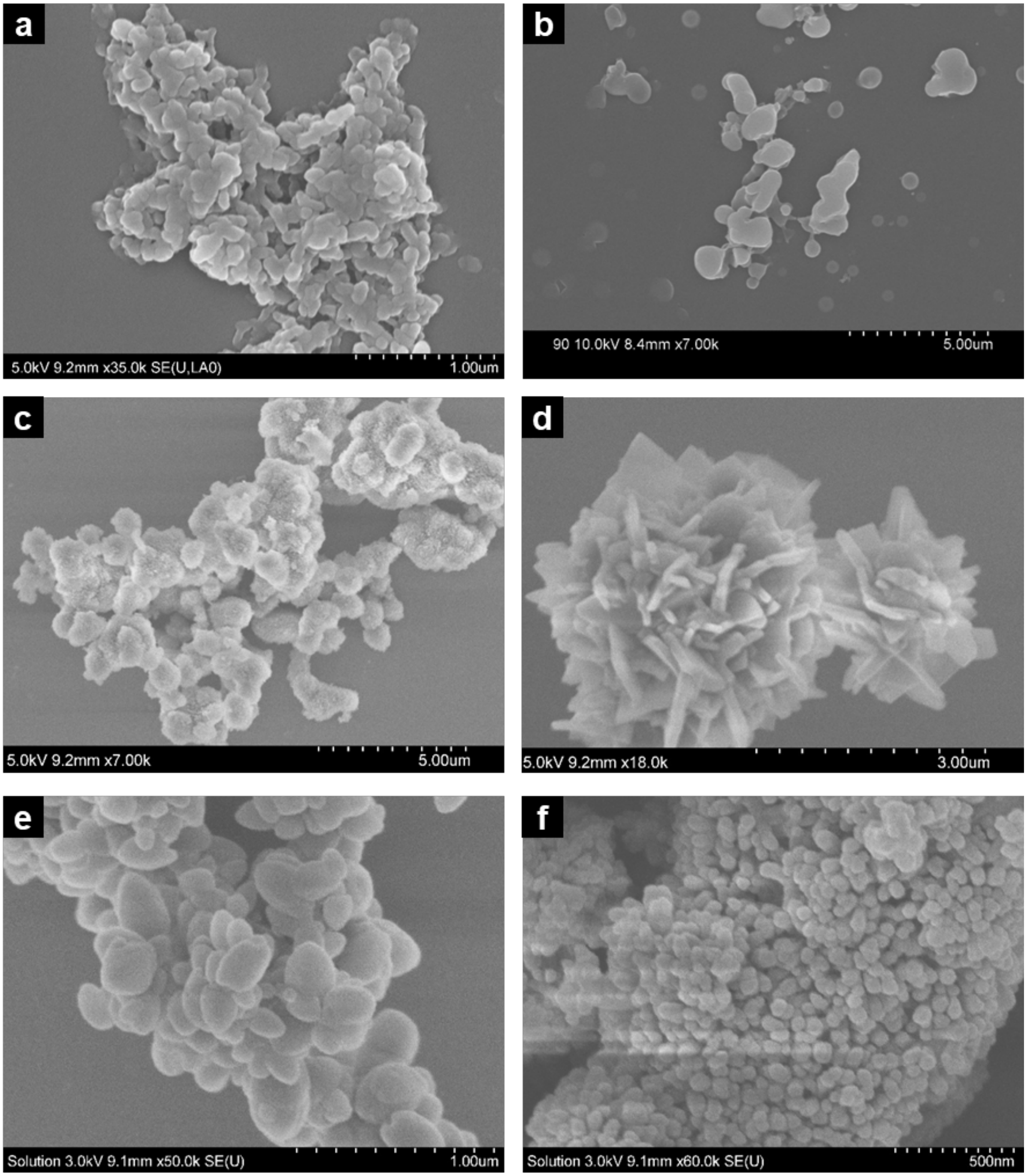

Figure S22. SEM images of (a) SonoCOF-J3, (b) sonoCOF-K3, (c) sonoCOF-A4, (d) sonoCOF-B4, (e) sonoCOF-F4 and (f) sonoCOF-G4.

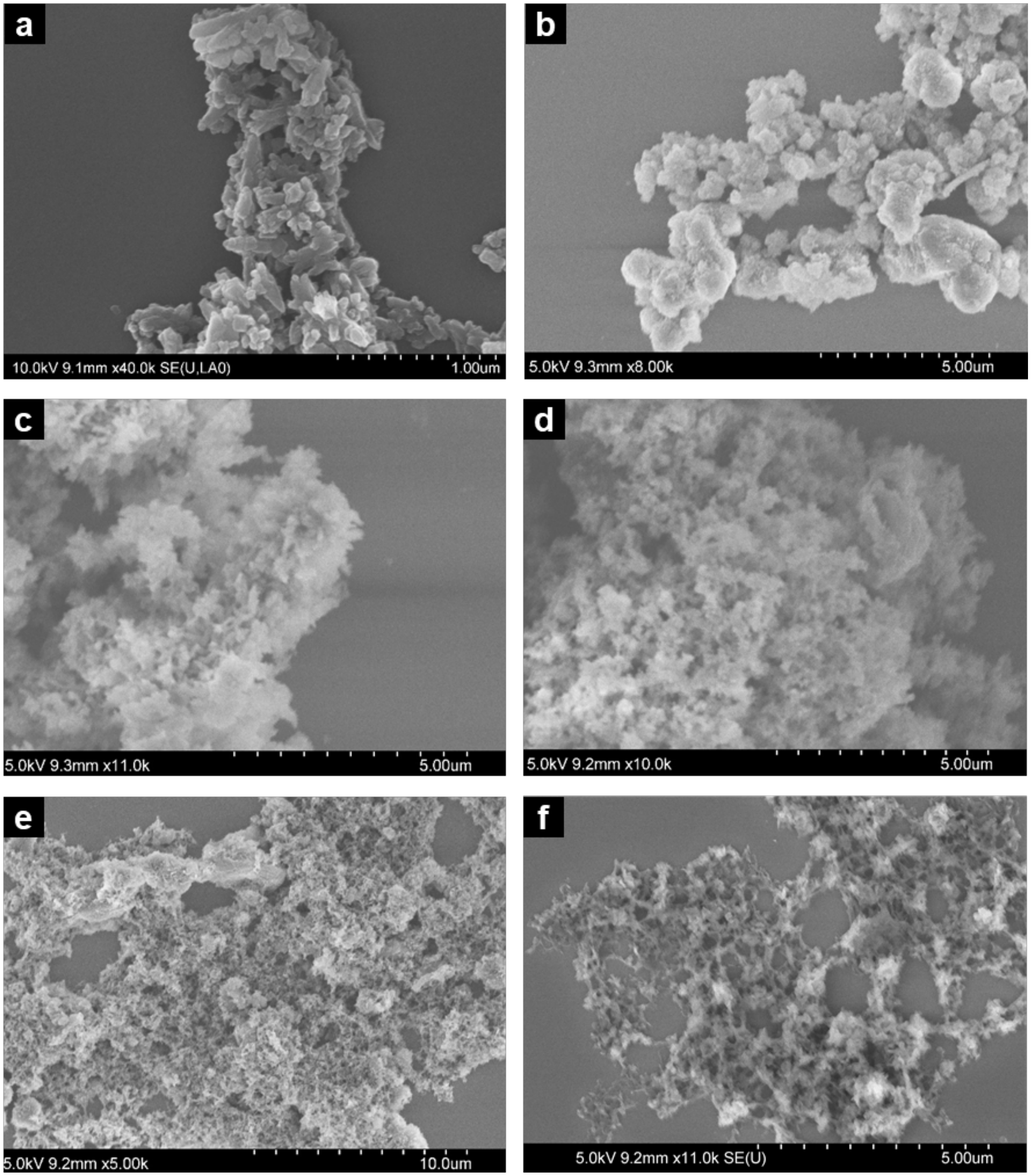

Figure S23. SEM images of (a) sonoCOF-J4, (b) sonoCOF-K4, (c) sonoCOF-F5, (d) sonoCOF-I5, (e) sonoCOF-F6 and (f) sonoCOF-G6.

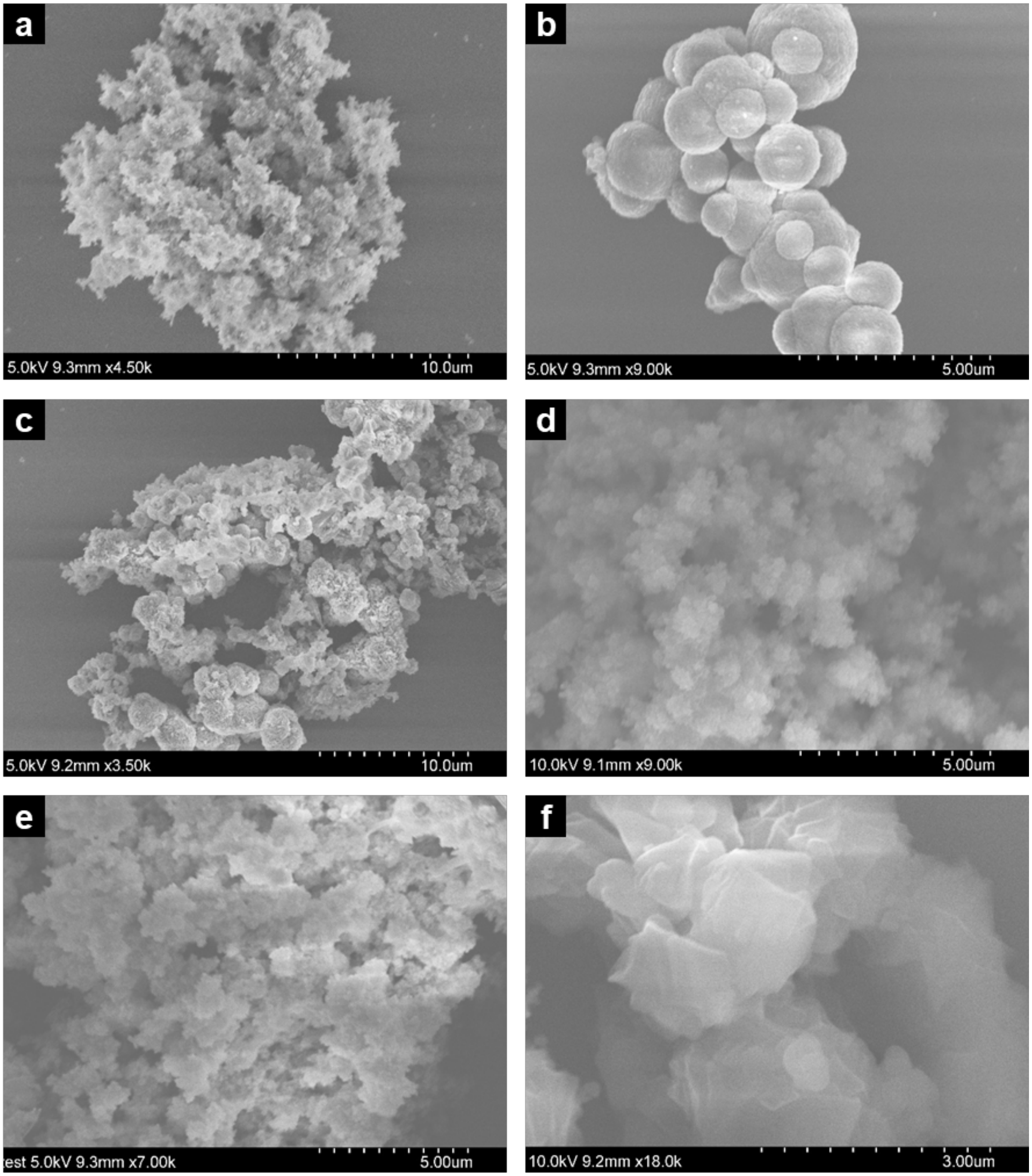

Figure S24. SEM images of (a) sonoCOF-I6, (b) sonoCOF-A7, (c) sonoCOF-B7, (d) sonoCOF-F7, (e) sonoCOF-G7 and (f) sonoCOF-H7.

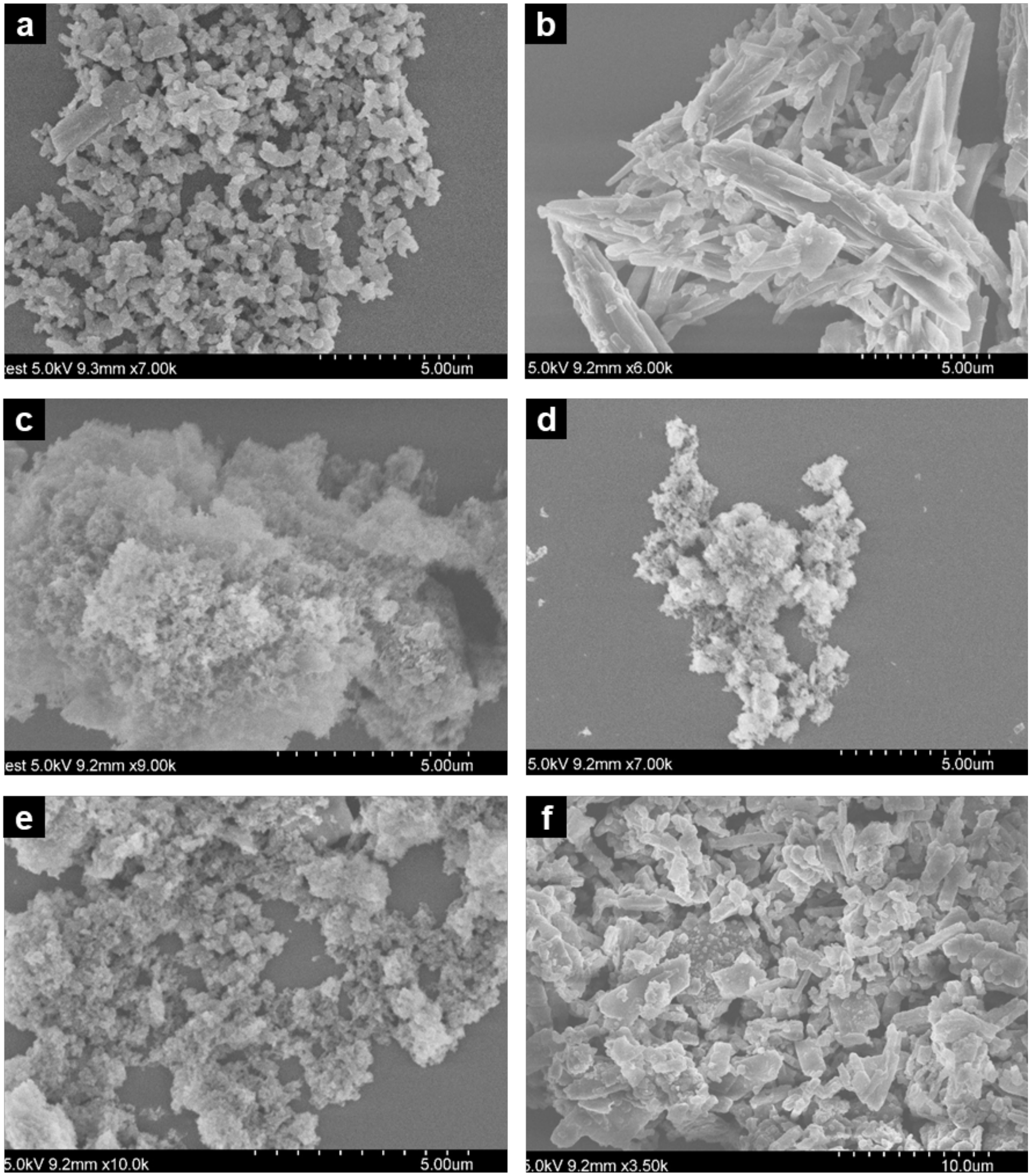

Figure S25. SEM images of (a) SonoCOF-F8, (b) sonoCOF-G8, (c) sonoCOF-I8, (d) sonoCOF-I9, (e) sonoCOF-I10 and (f) sonoCOF-A11.

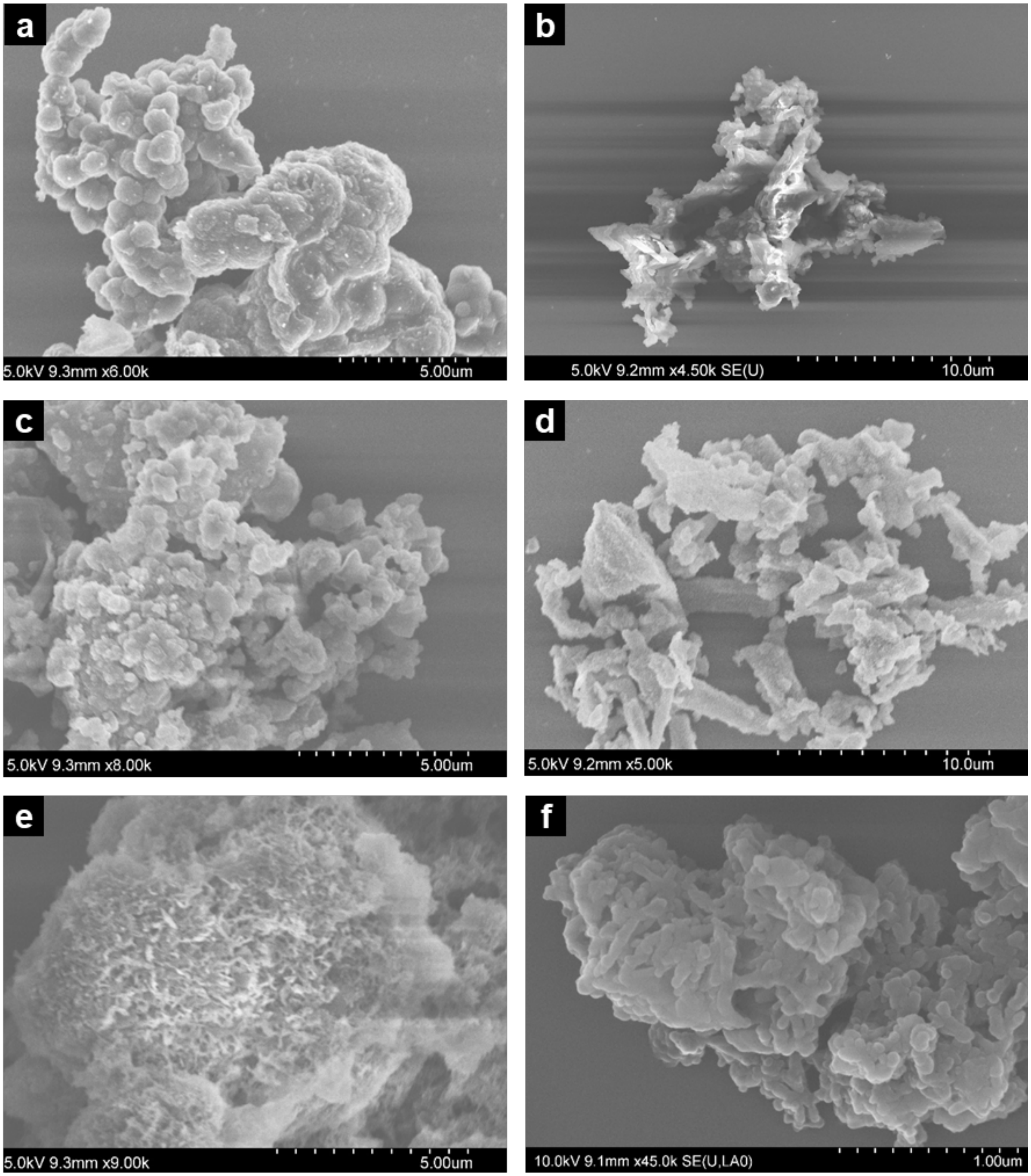

Figure S26. SEM images of (a) SonoCOF-B11, (b) sonoCOF-E11, (c) sonoCOF-F11, (d) sonoCOF-G11, (e) sonoCOF-J11 and (f) sonoCOF-K11.

3.6 Gas sorption analysis

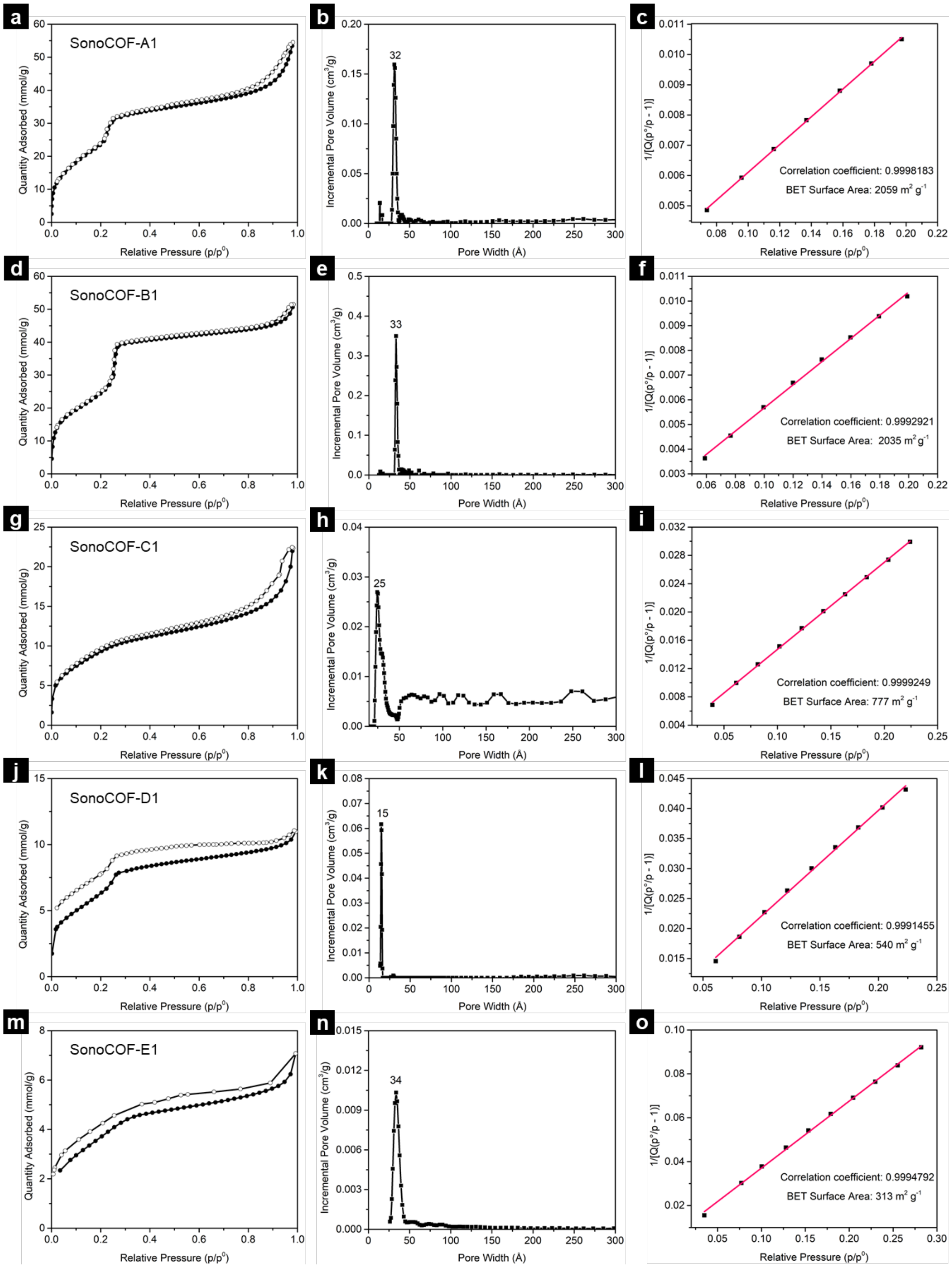

Figure S27. N_2 adsorption and desorption profiles (a, d, g, j and m), pore size distribution profile calculated by DFT (b, e, h, k and n) and BET surface area plot derived from N_2 sorption isotherm (c, f, I, l and o) of SonoCOF-A1, B1, C1, D1 and E1.

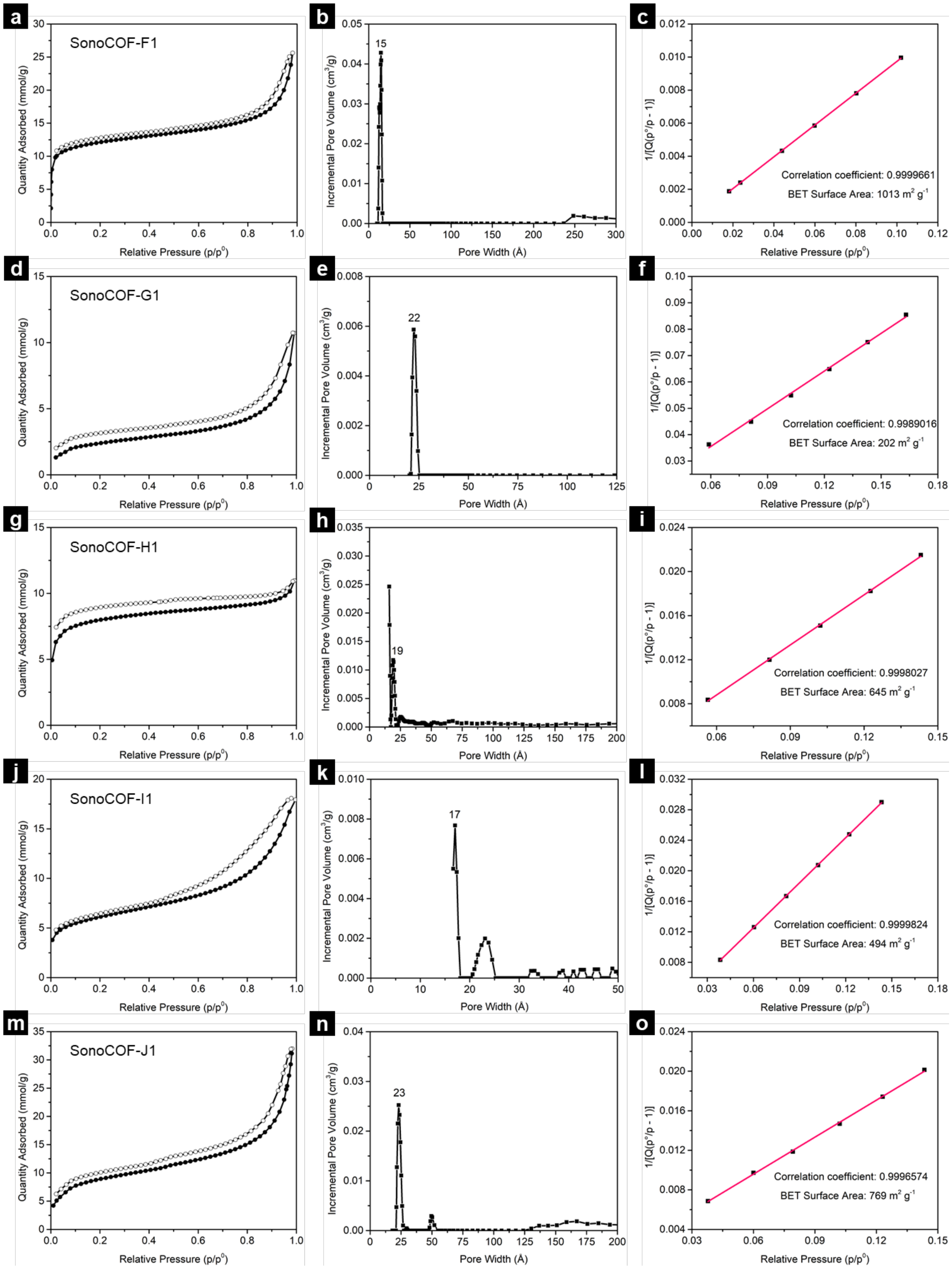

Figure S28. N_2 adsorption and desorption profiles (a, d, g, j and m), pore size distribution profile calculated by DFT (b, e, h, k and n) and BET surface area plot derived from N_2 sorption isotherm (c, f, I, l and o) of SonoCOF-F1, G1, H1, I1 and J1.

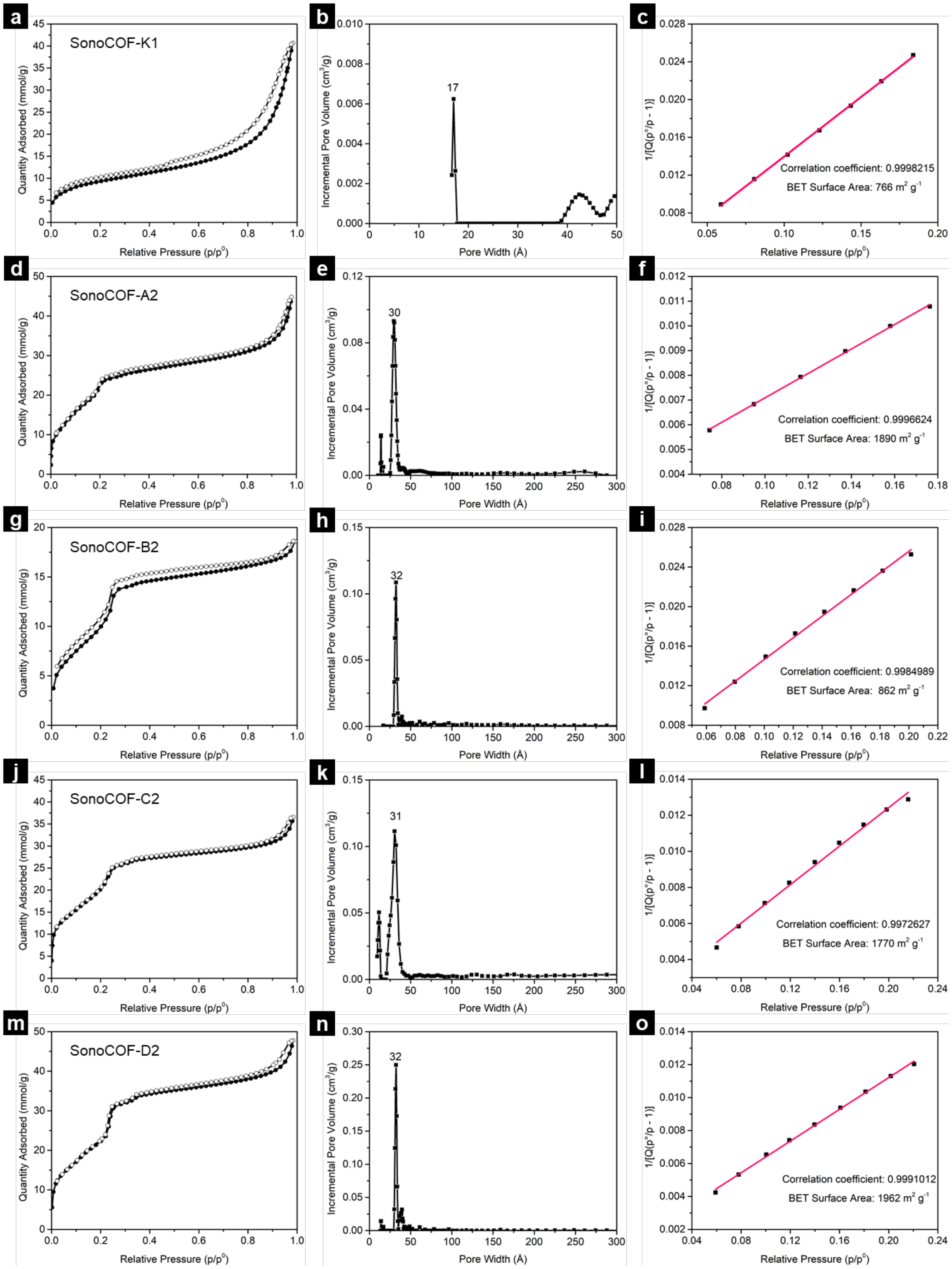

Figure S29. N_2 adsorption and desorption profiles (a, d, g, j and m), pore size distribution profile calculated by DFT (b, e, h, k and n) and BET surface area plot derived from N_2 sorption isotherm (c, f, I, l and o) of SonoCOF-K1, A2, B2, C2 and D2.

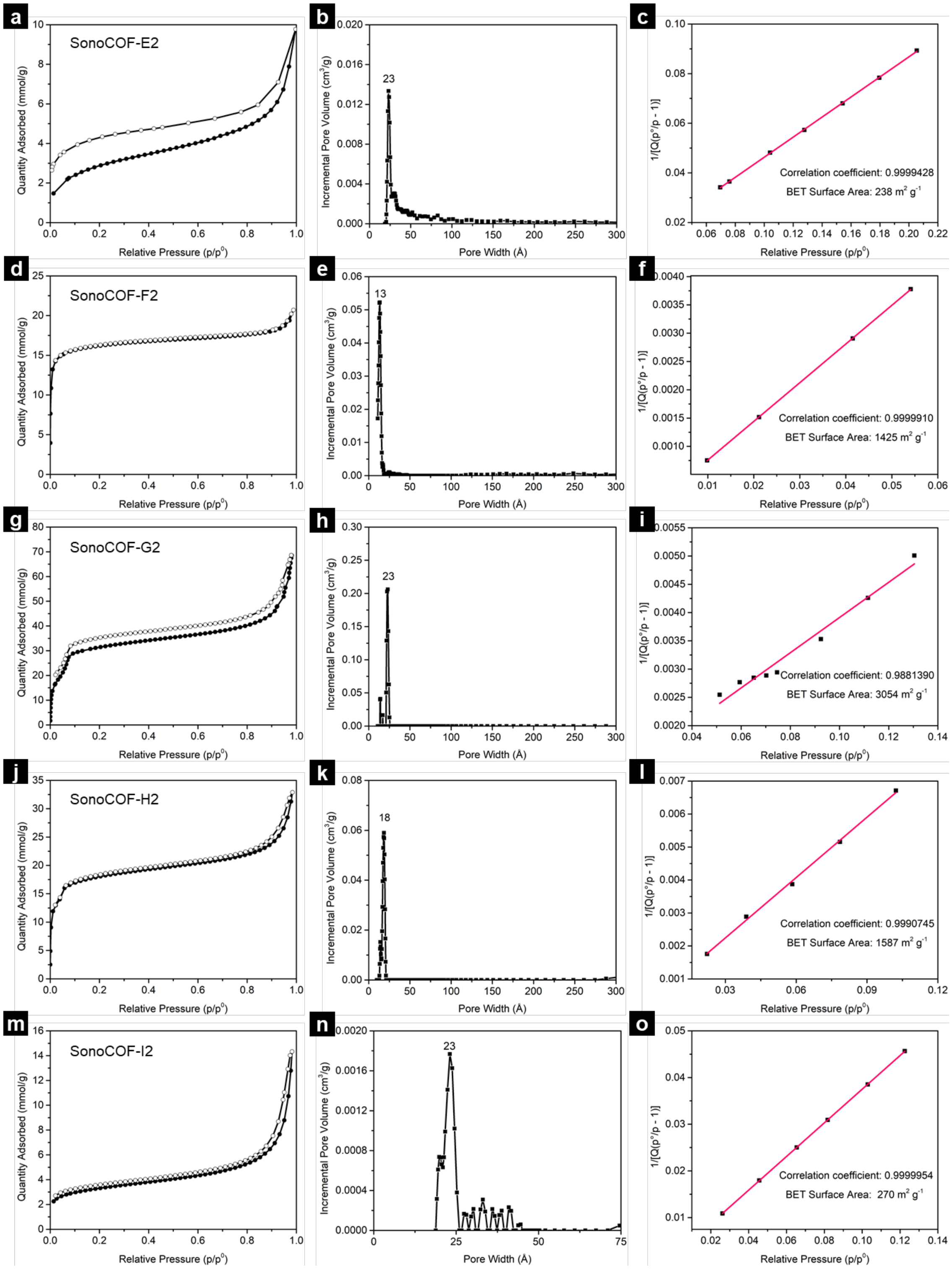

Figure S30. N_2 adsorption and desorption profiles (a, d, g, j and m), pore size distribution profile calculated by DFT (b, e, h, k and n) and BET surface area plot derived from N_2 sorption isotherm (c, f, I, l and o) of SonoCOF-E2, F2, G2, H2 and I2.

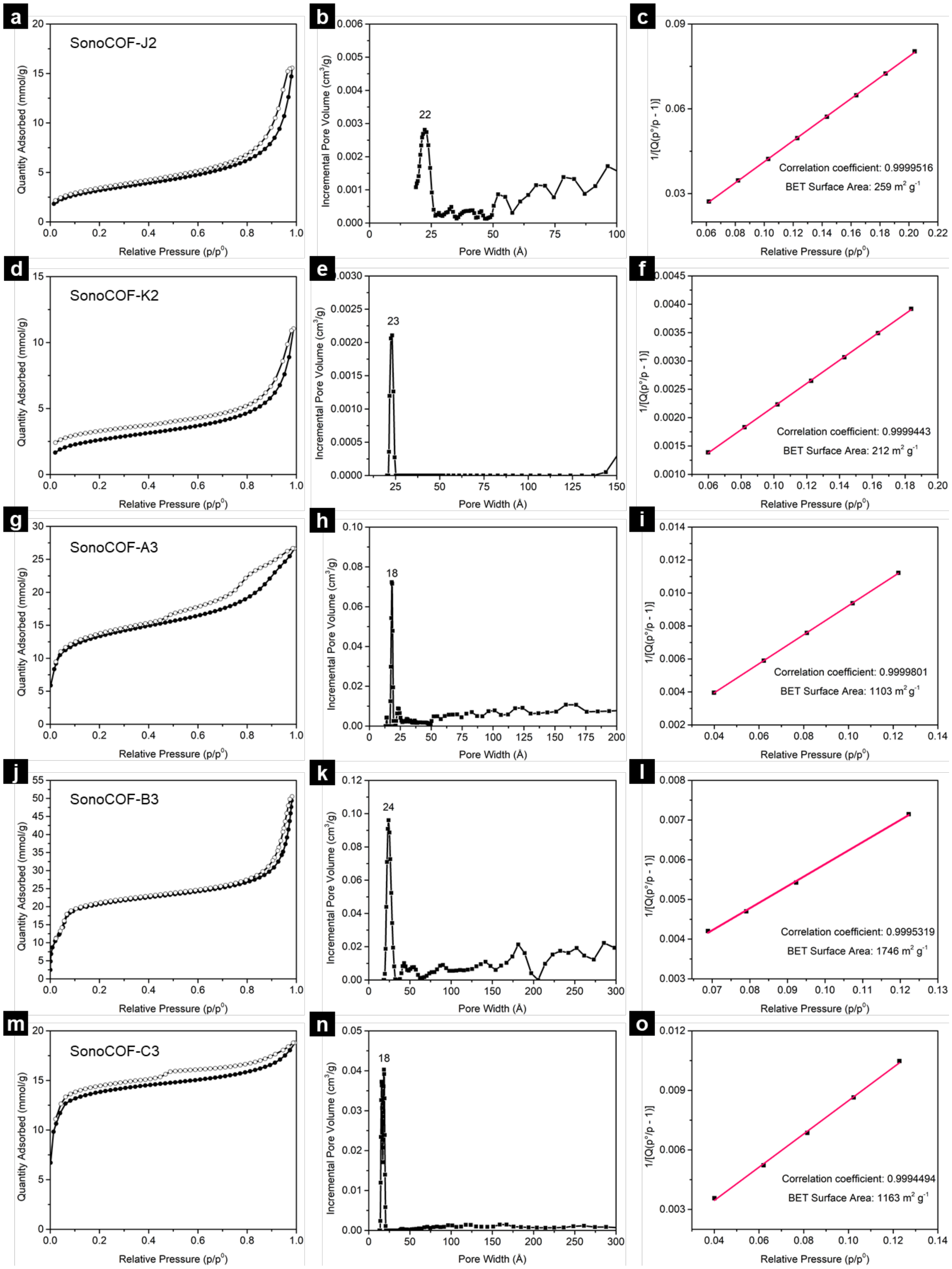

Figure S31. N_2 adsorption and desorption profiles (a, d, g, j and m), pore size distribution profile calculated by DFT (b, e, h, k and n) and BET surface area plot derived from N_2 sorption isotherm (c, f, I, l and o) of SonoCOF-J2, K2, A3, B3 and C3.

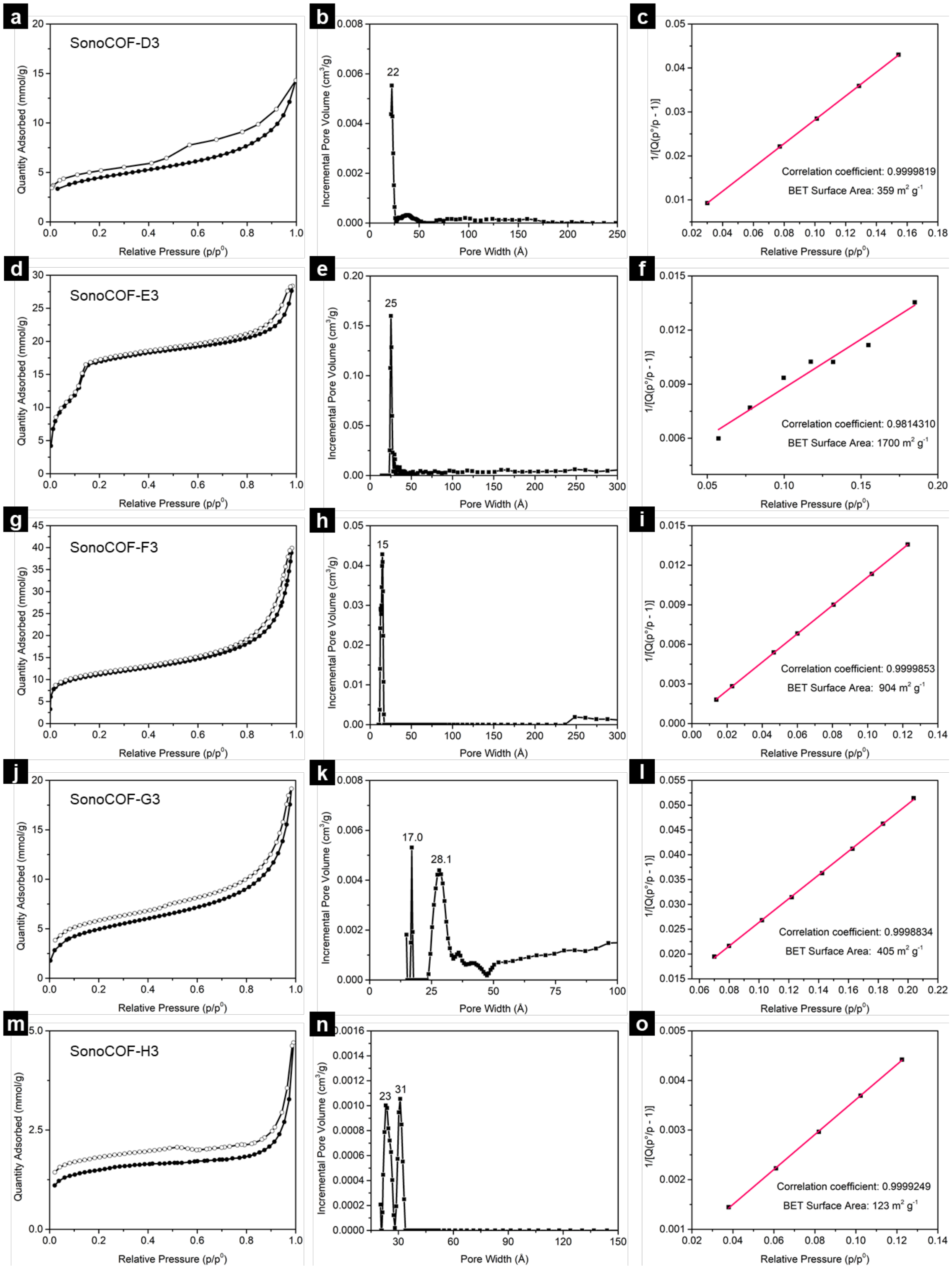

Figure S32. N_2 adsorption and desorption profiles (a, d, g, j and m), pore size distribution profile calculated by DFT (b, e, h, k and n) and BET surface area plot derived from N_2 sorption isotherm (c, f, I, l and o) of SonoCOF-D3, E3, F3, G3 and H3.

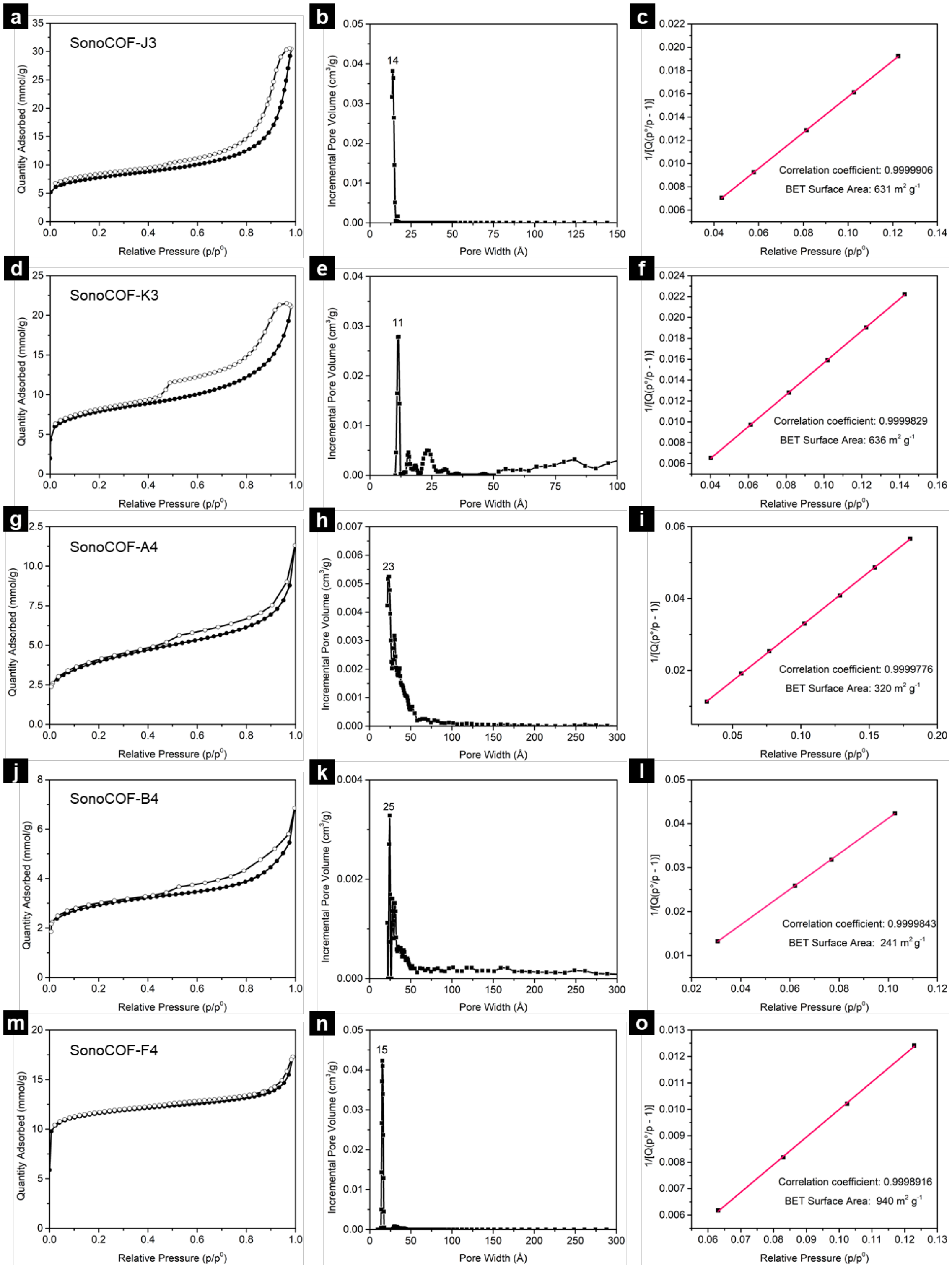

Figure S33. N_2 adsorption and desorption profiles (a, d, g, j and m), pore size distribution profile calculated by DFT (b, e, h, k and n) and BET surface area plot derived from N_2 sorption isotherm (c, f, I, l and o) of SonoCOF-J3, K3, A4, B4 and F4.

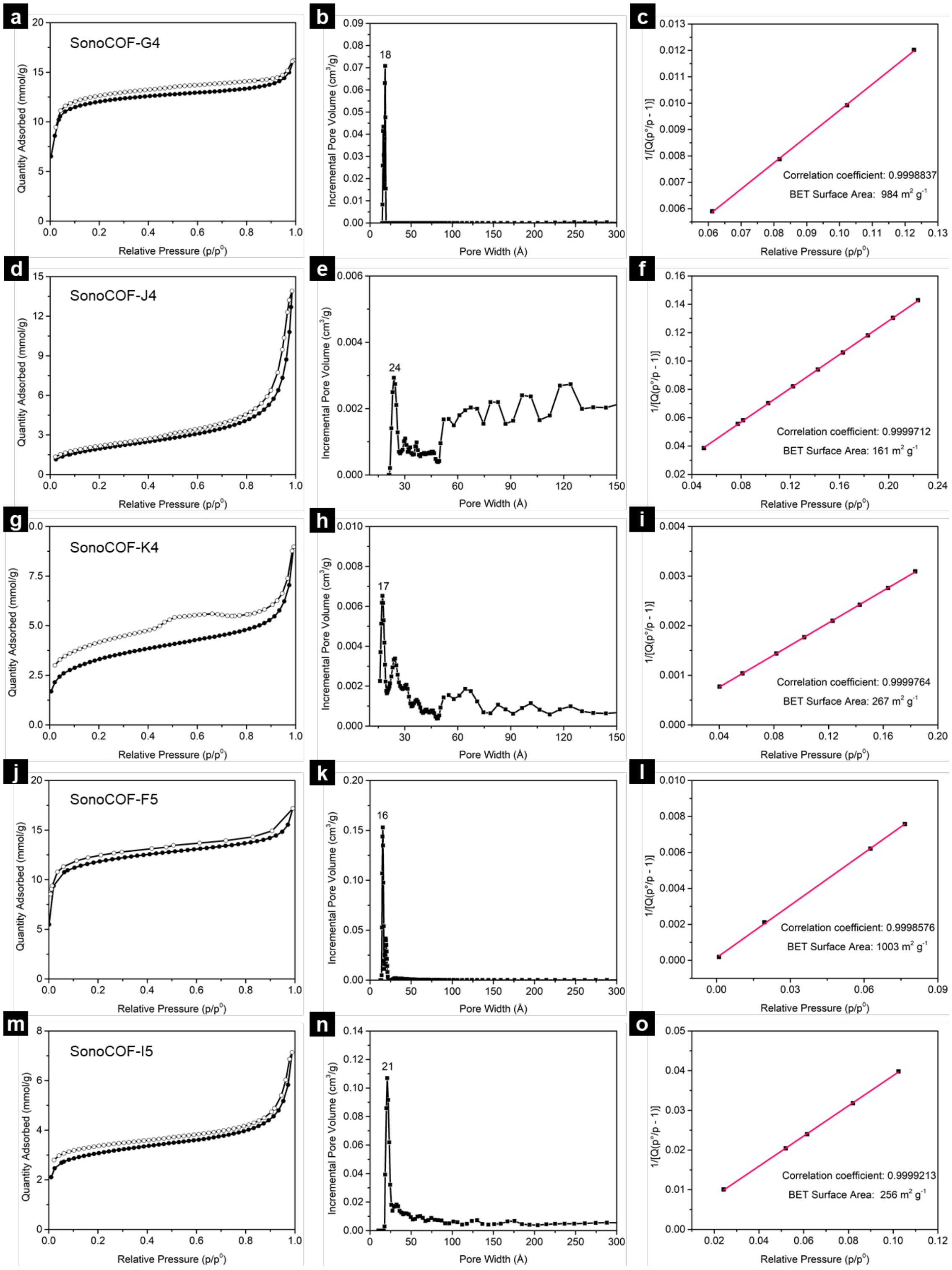

Figure S34. N_2 adsorption and desorption profiles (a, d, g, j and m), pore size distribution profile calculated by DFT (b, e, h, k and n) and BET surface area plot derived from N_2 sorption isotherm (c, f, I, l and o) of SonoCOF-G4, J4, K4, F5 and I5.

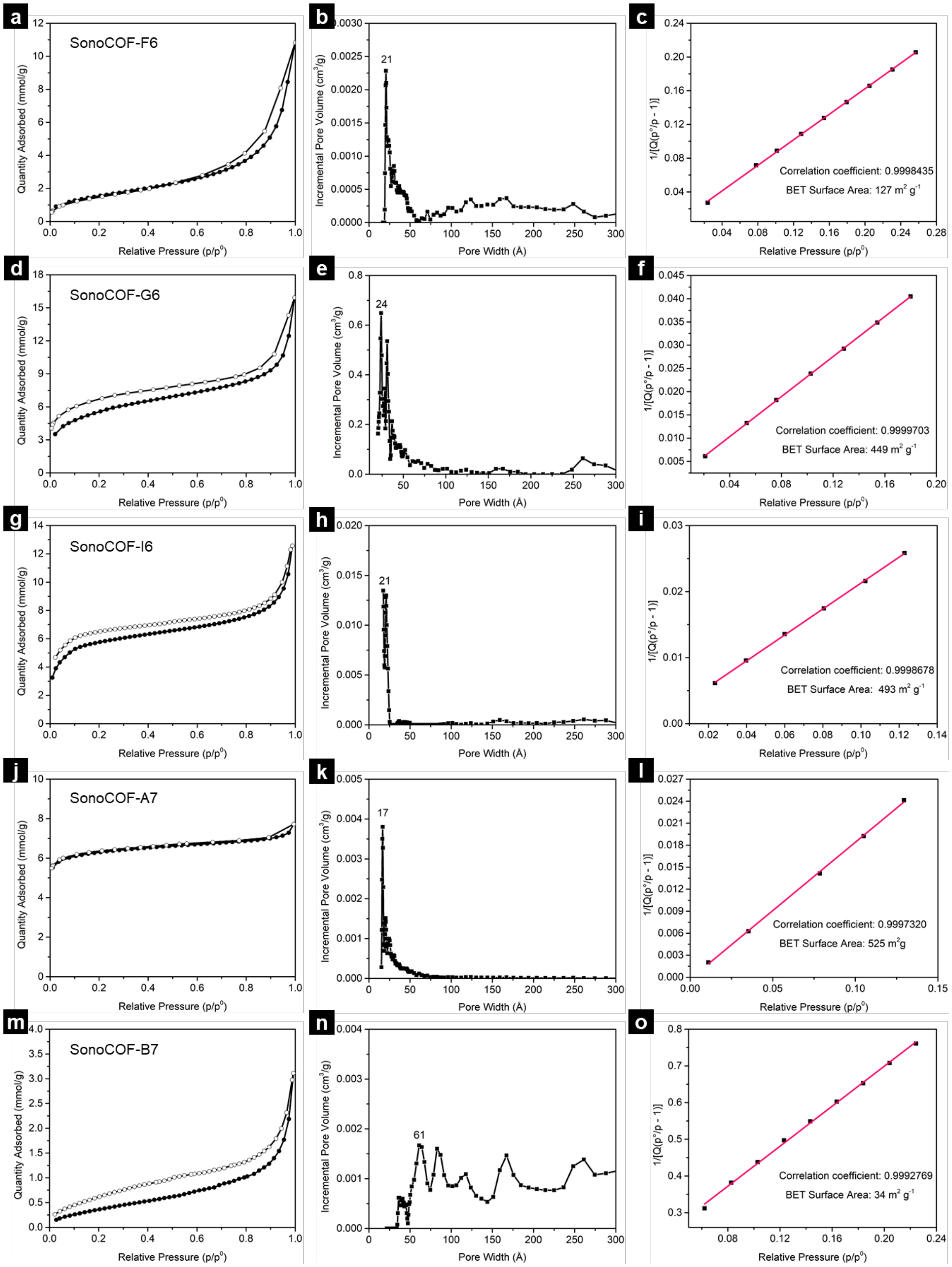

Figure S35. N_2 adsorption and desorption profiles (a, d, g, j and m), pore size distribution profile calculated by DFT (b, e, h, k and n) and BET surface area plot derived from N_2 sorption isotherm (c, f, I, l and o) of SonoCOF-F6, G6, I6, A7 and B7.

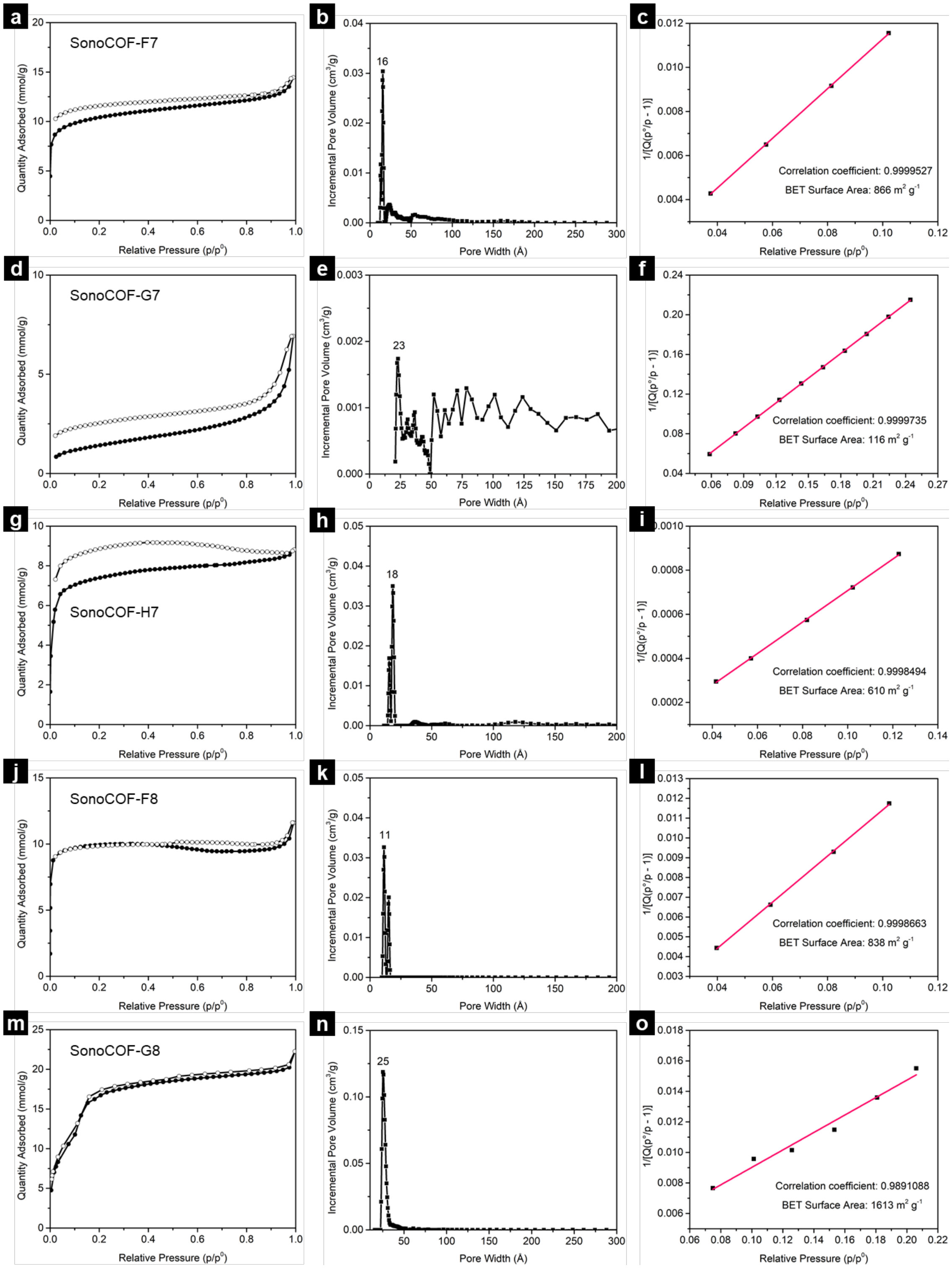

Figure S36. N_2 adsorption and desorption profiles (a, d, g, j and m), pore size distribution profile calculated by DFT (b, e, h, k and n) and BET surface area plot derived from N_2 sorption isotherm (c, f, I, l and o) of SonoCOF-F7, G7, H7, F8 and G8.

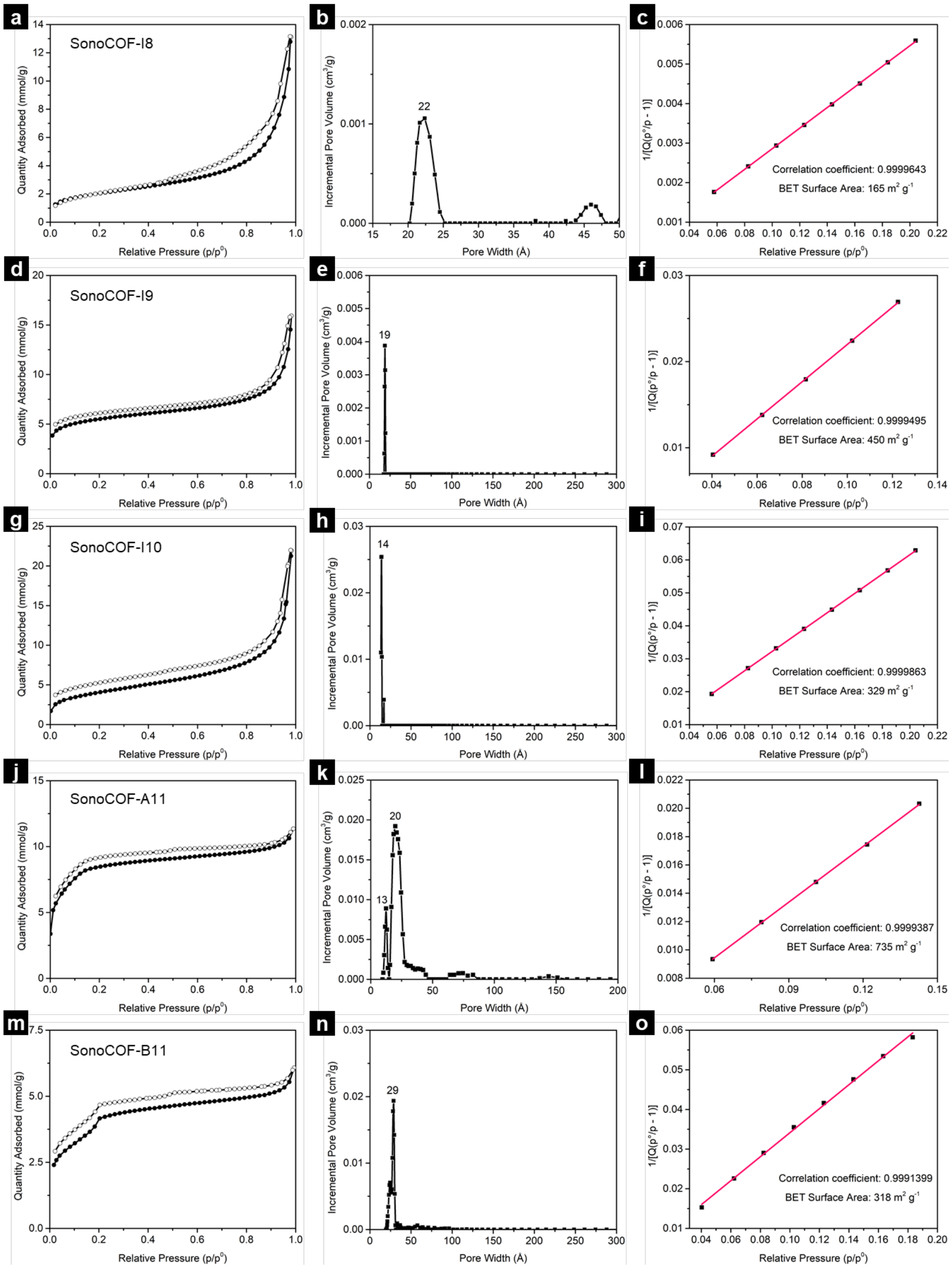

Figure S37. N_2 adsorption and desorption profiles (a, d, g, j and m), pore size distribution profile calculated by DFT (b, e, h, k and n) and BET surface area plot derived from N_2 sorption isotherm (c, f, I, l and o) of SonoCOF-I8, I9, I10, A11 and B11.

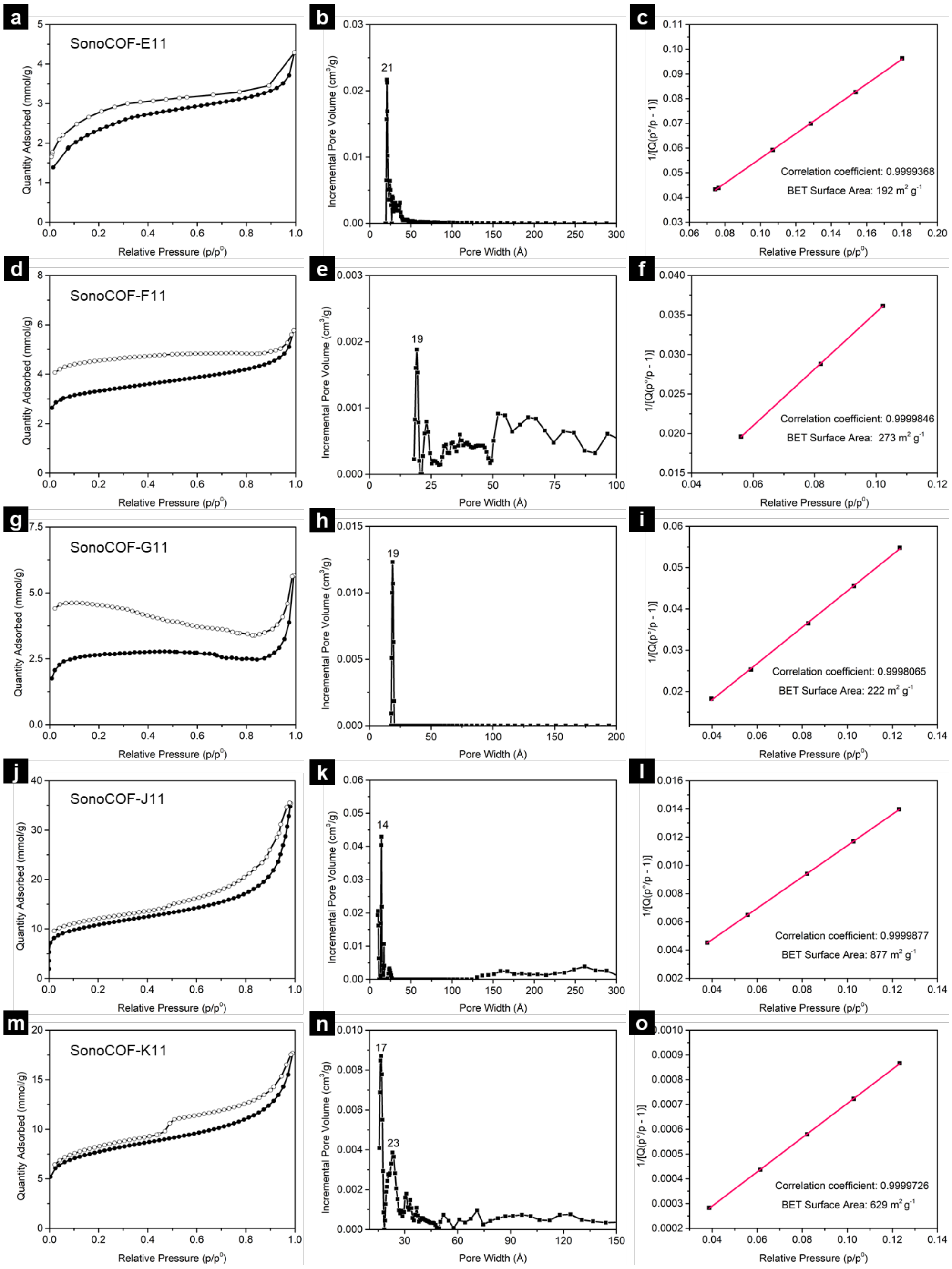

Figure S38. N_2 adsorption and desorption profiles (a, d, g, j and m), pore size distribution profile calculated by DFT (b, e, h, k and n) and BET surface area plot derived from N_2 sorption isotherm (c, f, I, l and o) of SonoCOF-E11, F11, G11, J11 and K11. Note that the hysteresis and increase in gas uptake observed on the desorption isotherm for SonoCOF-G11 suggests that this sample did not equilibrate properly under the measurement conditions for this particular material.

3.7 ^{13}C cross-polarization magic-angle spinning (CP/MAS) NMR analysis

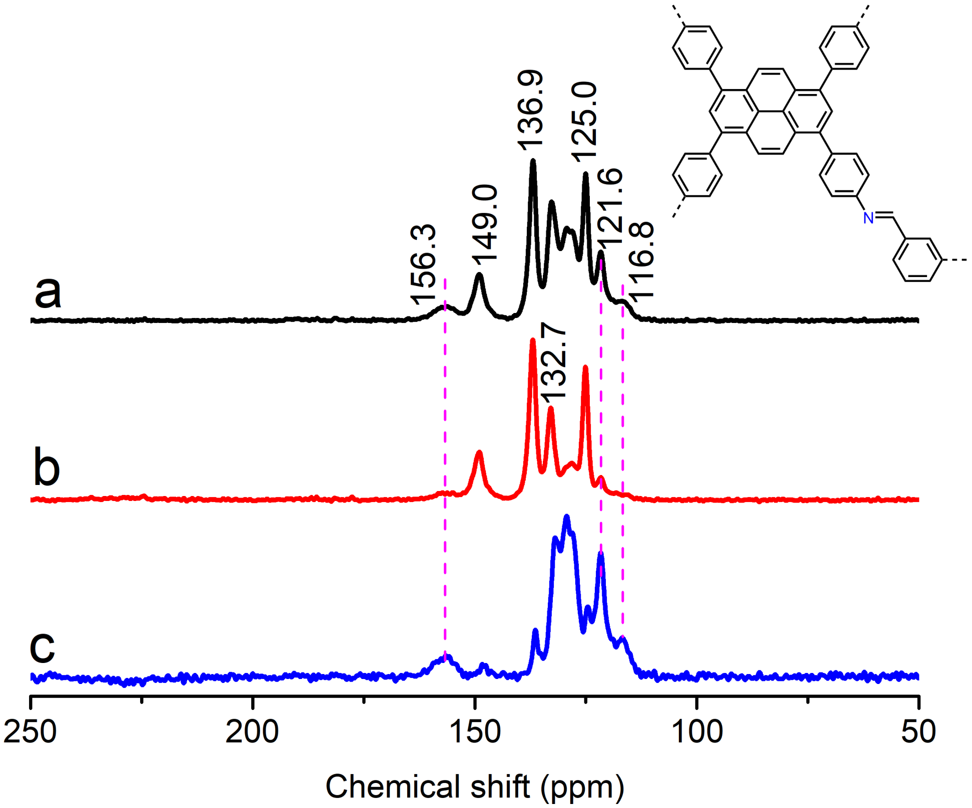

Figure S39. Full ^{13}C cross-polarization magic-angle-spinning (CP-MAS) NMR spectroscopy (a) and one with "interrupted decoupling" (b, often also called non-quaternary suppression) of sonoCOF-J3. In the latter, signals from CH and CH_2 species are largely suppressed leaving signals from quaternary and methyl carbons. The difference (c) between two spectra shows the protonated carbon signals. The resonance associated with the imine functionality was displayed at 156.3 ppm.

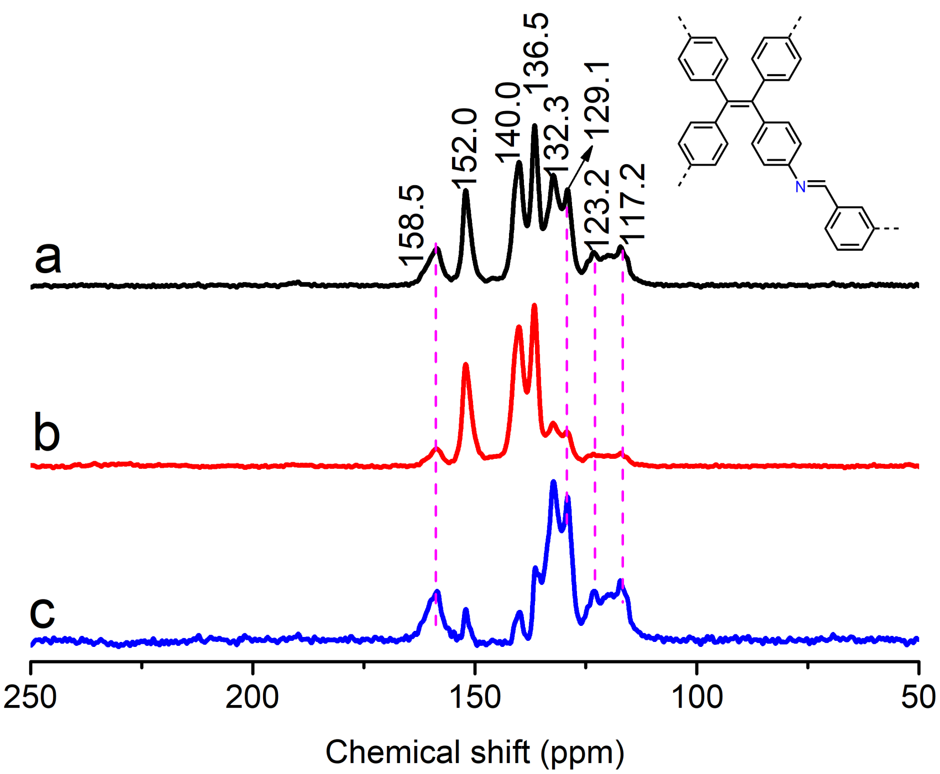

Figure S40. Full ^{13}C cross-polarization magic-angle-spinning (CP-MAS) NMR spectroscopy (a) and one with "interrupted decoupling" (b, often also called non-quaternary suppression) of sonoCOF-J4. In the latter, signals from CH and CH_2 species are largely suppressed leaving signals from quaternary and methyl carbons. The difference (c) between two spectra shows the protonated carbon signals. The resonance associated with the imine functionality was displayed at 158.5 ppm.

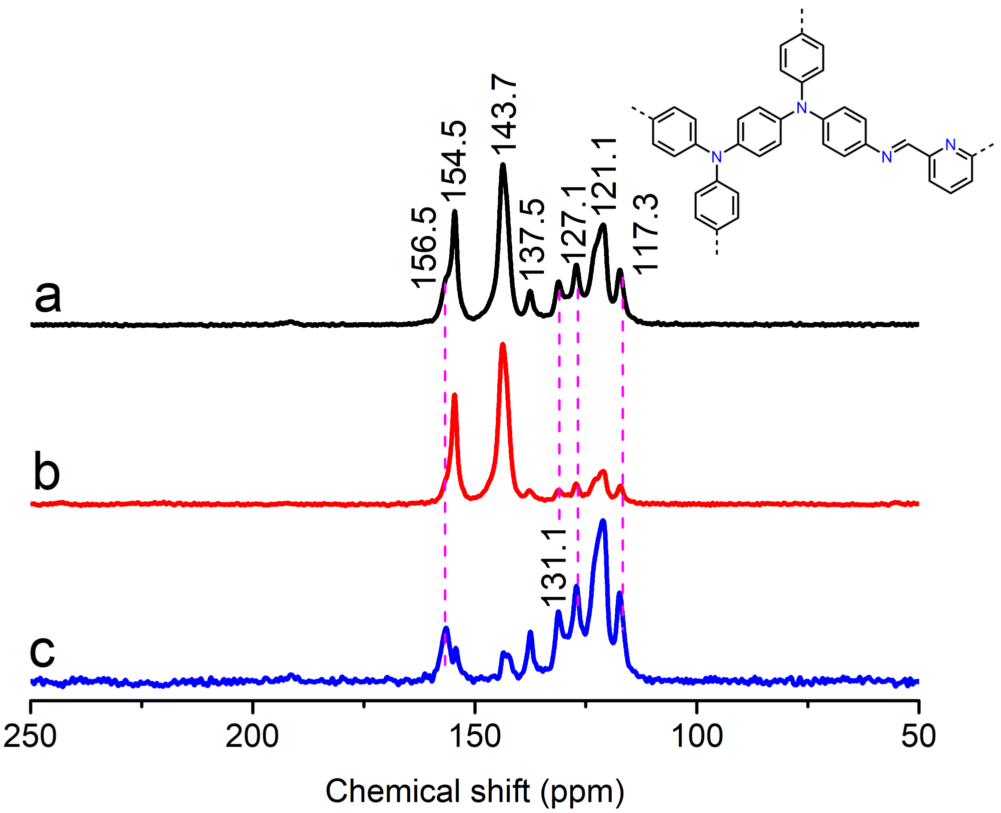

Figure S41. Full ^{13}C cross-polarization magic-angle-spinning (CP-MAS) NMR spectroscopy (a) and one with "interrupted decoupling" (b, often also called non-quaternary suppression) of sonoCOF-K11. In the latter, signals from CH and CH_2 species are largely suppressed leaving signals from quaternary and methyl carbons. The difference (c) between two spectra shows the protonated carbon signals. The resonance associated with the imine functionality was displayed at 156.5 ppm.

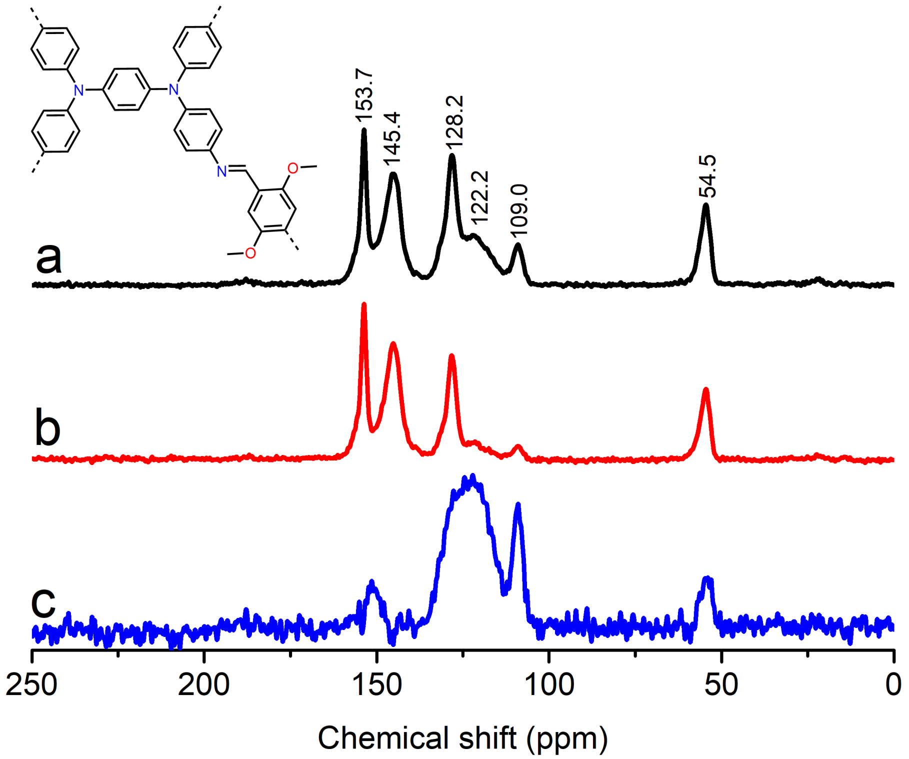

Figure S42. Full ^{13}C cross-polarization magic-angle-spinning (CP-MAS) NMR spectroscopy (a) and one with "interrupted decoupling" (b, often also called non-quaternary suppression) of sonoCOF-A11. In the latter, signals from CH and CH_2 species are largely suppressed leaving signals from quaternary and methyl carbons. The difference (c) between two spectra shows the protonated carbon signals. The resonance associated with the imine functionality was displayed at 153.7 ppm.

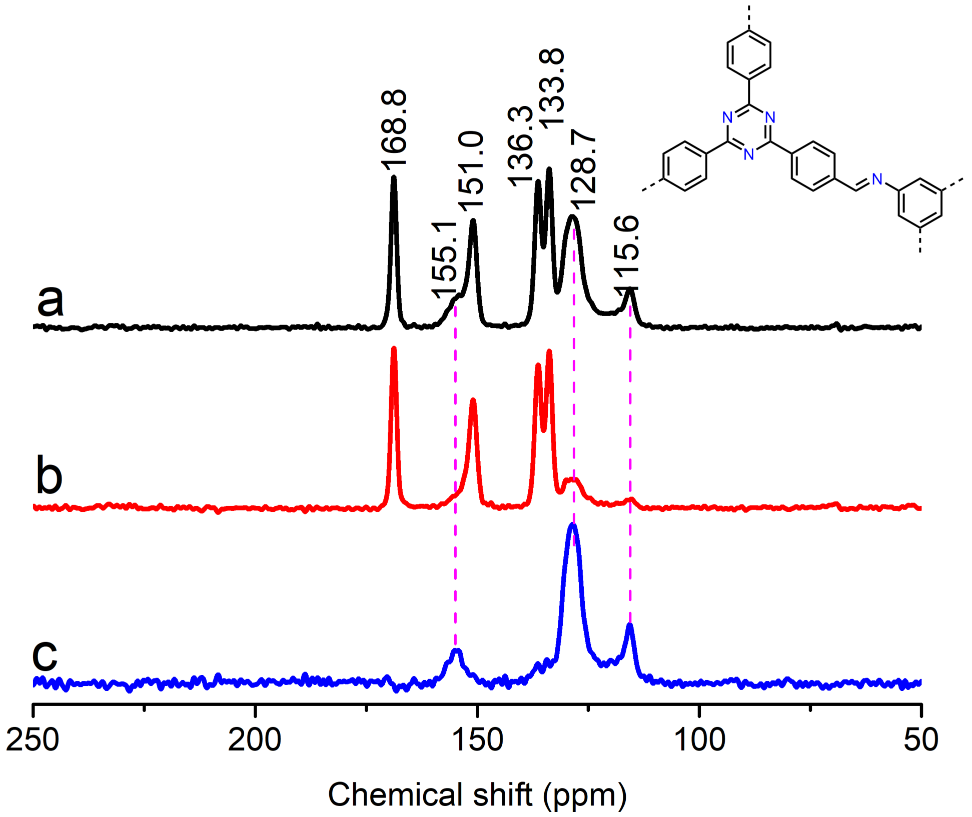

Figure S43. Full ^{13}C cross-polarization magic-angle-spinning (CP-MAS) NMR spectroscopy (a) and one with "interrupted decoupling" (b, often also called non-quaternary suppression) of sonoCOF-F2 before photocatalysis. In the latter, signals from CH and CH_2 species are largely suppressed leaving signals from quaternary and methyl carbons. The difference (c) between two spectra shows the protonated carbon signals. The resonance associated with the imine functionality was displayed at 155.1 ppm.

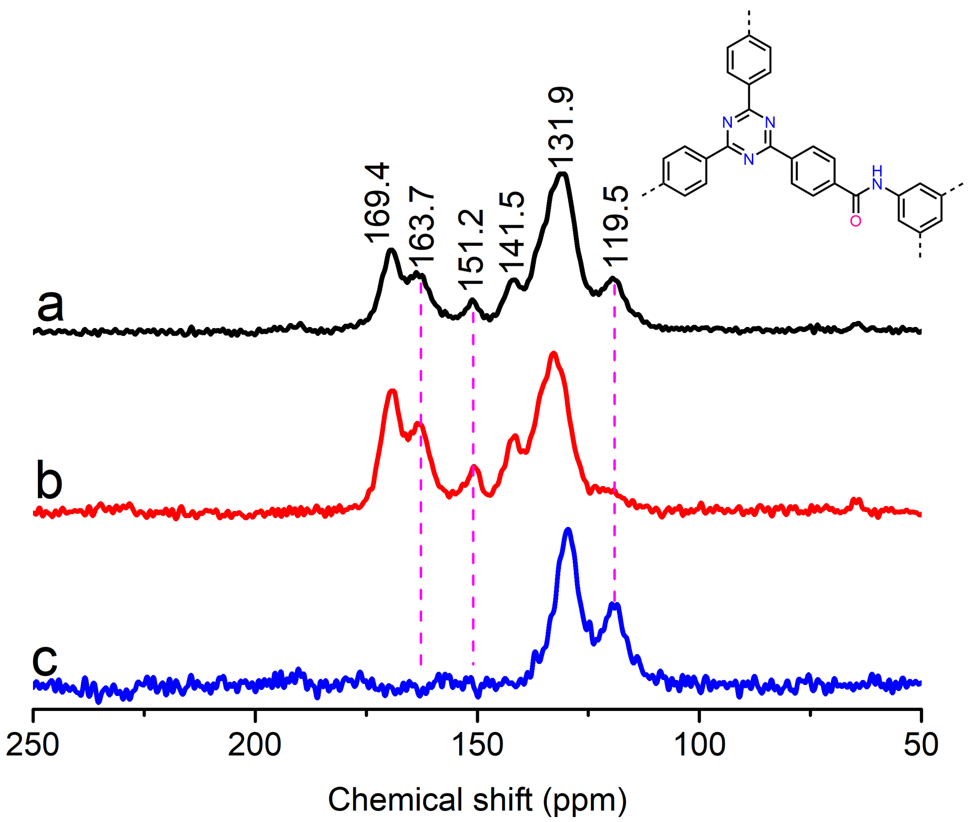

Figure S44. Full ^{13}C cross-polarization magic-angle-spinning (CP-MAS) NMR spectroscopy (a) and one with “interrupted decoupling” (b, often also called non-quaternary suppression) of sonoCOF-F2 after photocatalysis (96h). In the latter, signals from CH and CH_2 species are largely suppressed leaving signals from quaternary and methyl carbons. The difference (c) between two spectra shows the protonated carbon signals. The resonance associated with the amide functionality was displayed at 163.7 ppm. Reaction condition: 50 mg sonoCOF-F2, 60 ml water, O_2, 300 W Xe lamp ($\lambda > 420$ nm) for 96 h

3.8 UV-visible absorption spectra

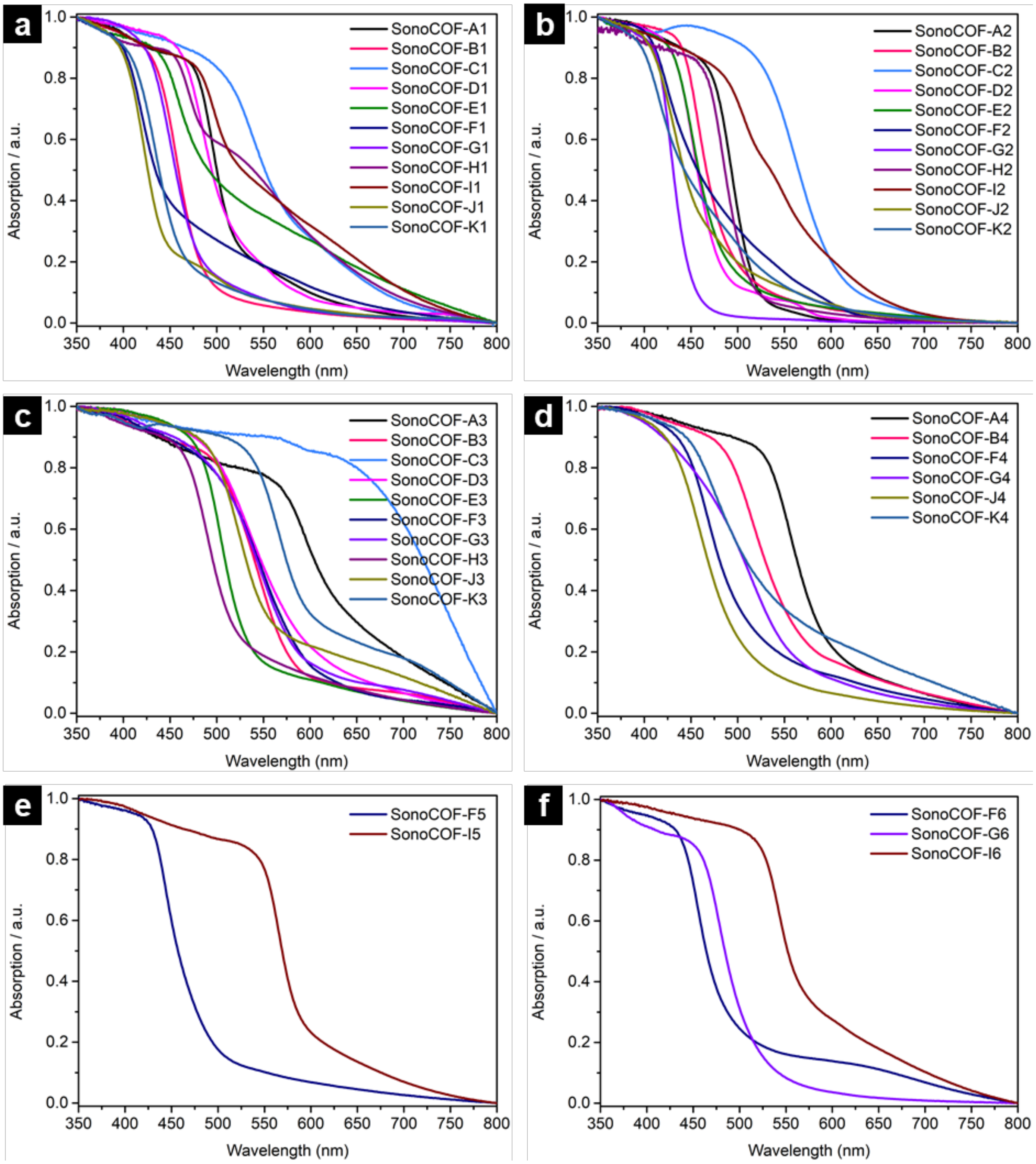

Figure S45. UV spectra of (a) sonoCOF-A1, -B1, -C1, -D1, -E1, -F1, -G1, -H1, -I1, -J1 and -K1; (b) sonoCOF-A2, -B2, -C2, -D2, E2, -F2, -G2, -H2, -I2, -J2 and -K2; (c) sonoCOF-A3, -B3, -C3, -D3, -E3, -F3, -G3, -H3, -J3 and -K3; (d) sonoCOF-A4, -B4, -F4, -G4, -J4 and -K4; (e) sonoCOF-F5 and -I5; (f) sonoCOF-F6, -G6 and I6.

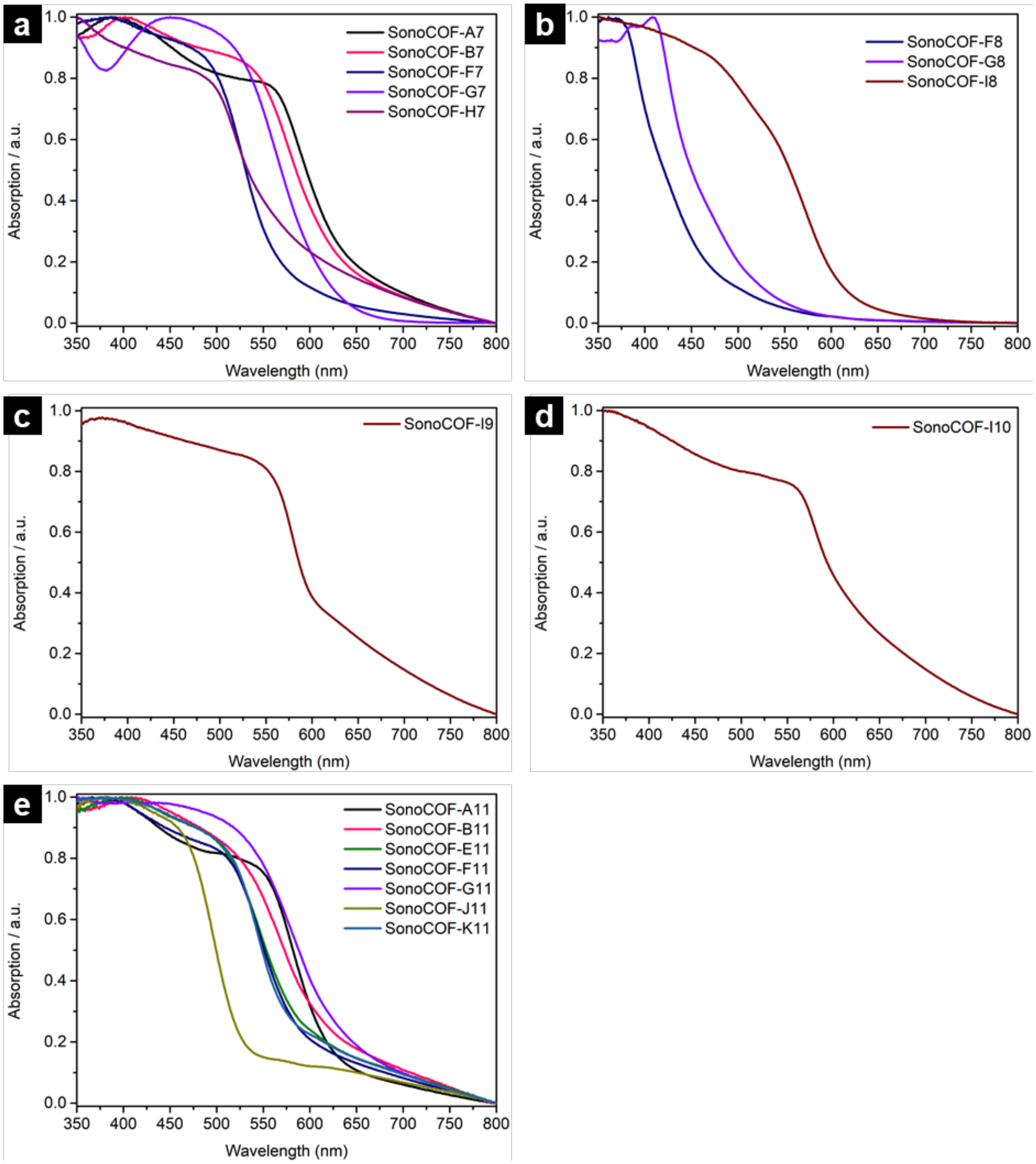

Figure S46. UV spectra of (a) sonoCOF-A7, -B7, -F7, -G7 and -H7; (b) sonoCOF-F8, -G8 and -I8; (c) sonoCOF-I9; (d) sonoCOF-I10; (e) sonoCOF-A11, B11, E11, F11, G11, J11 and K11.

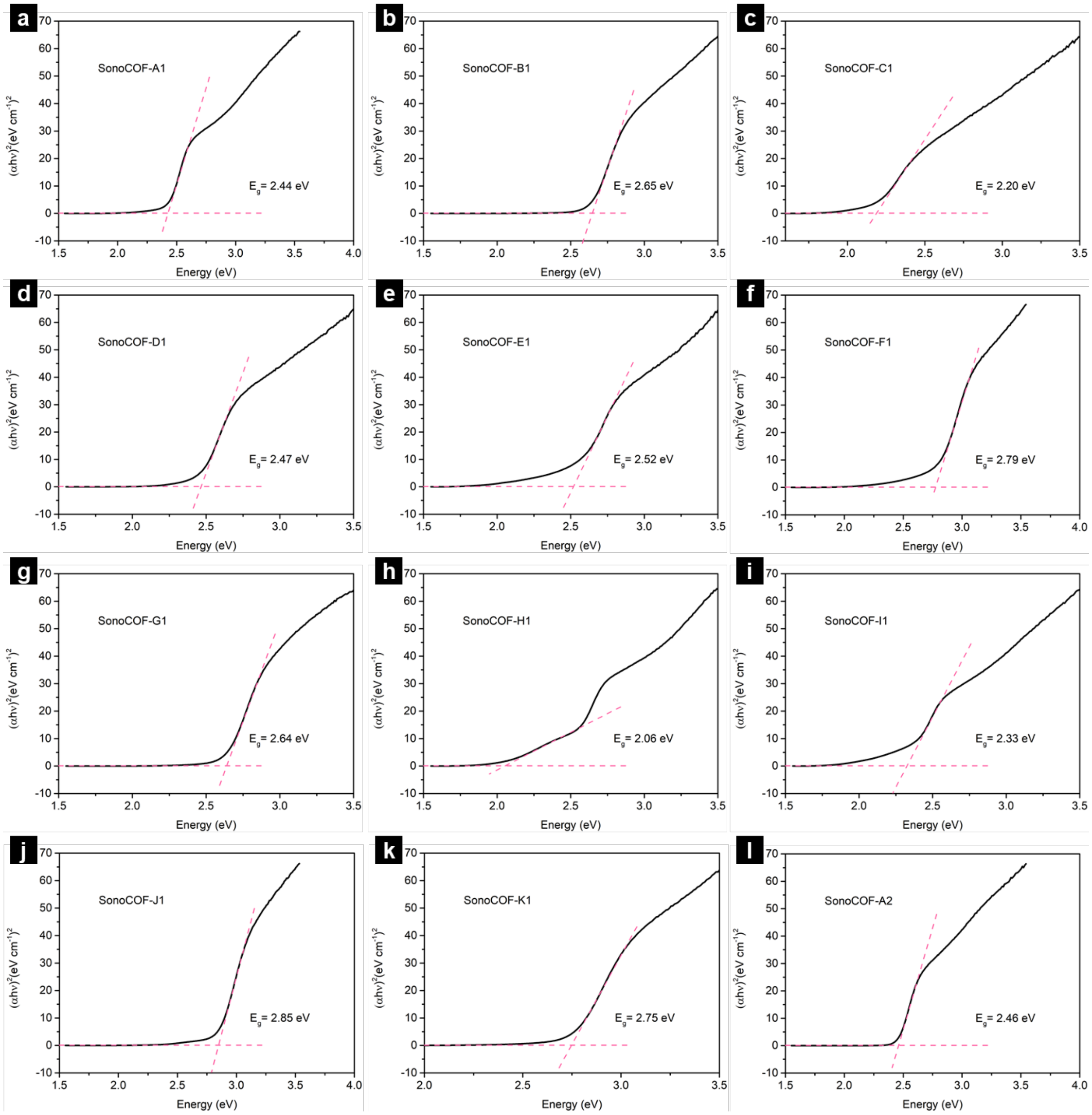

Figure S47. Kubelka-Munk-transformed reflectance spectra of sonoCOFs A1-K1 and sonoCOF-A2.

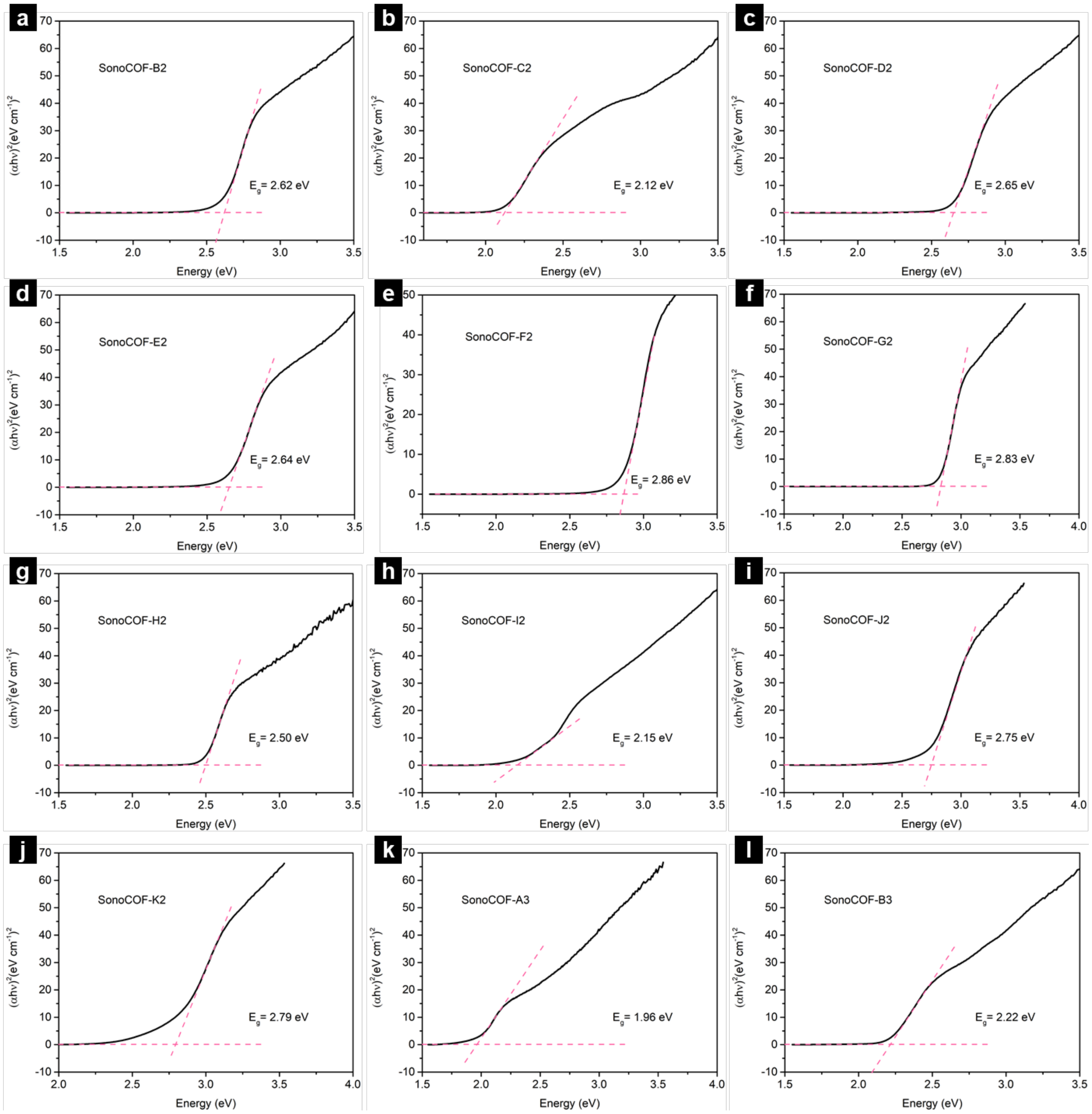

Figure S48. Kubelka-Munk-transformed reflectance spectra of sonoCOFs B2-K2 and sonoCOF-A3 and -B3.

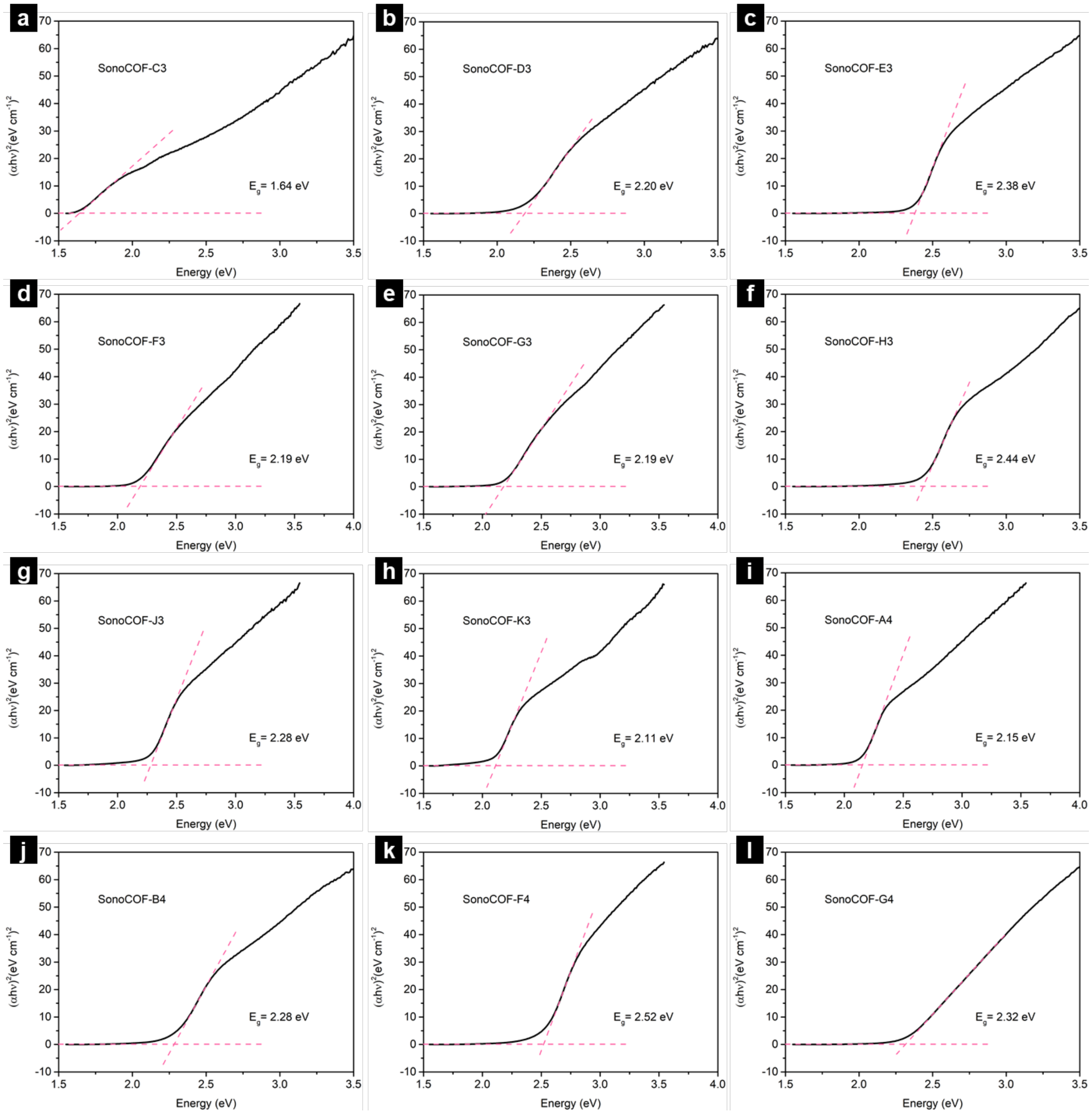

Figure S49. Kubelka-Munk-transformed reflectance spectra of sonoCOF-C3, -D3, -E3, -F3, -G3, -H3, -J3, -K3, -A4, -B4, -F4 and -G4.

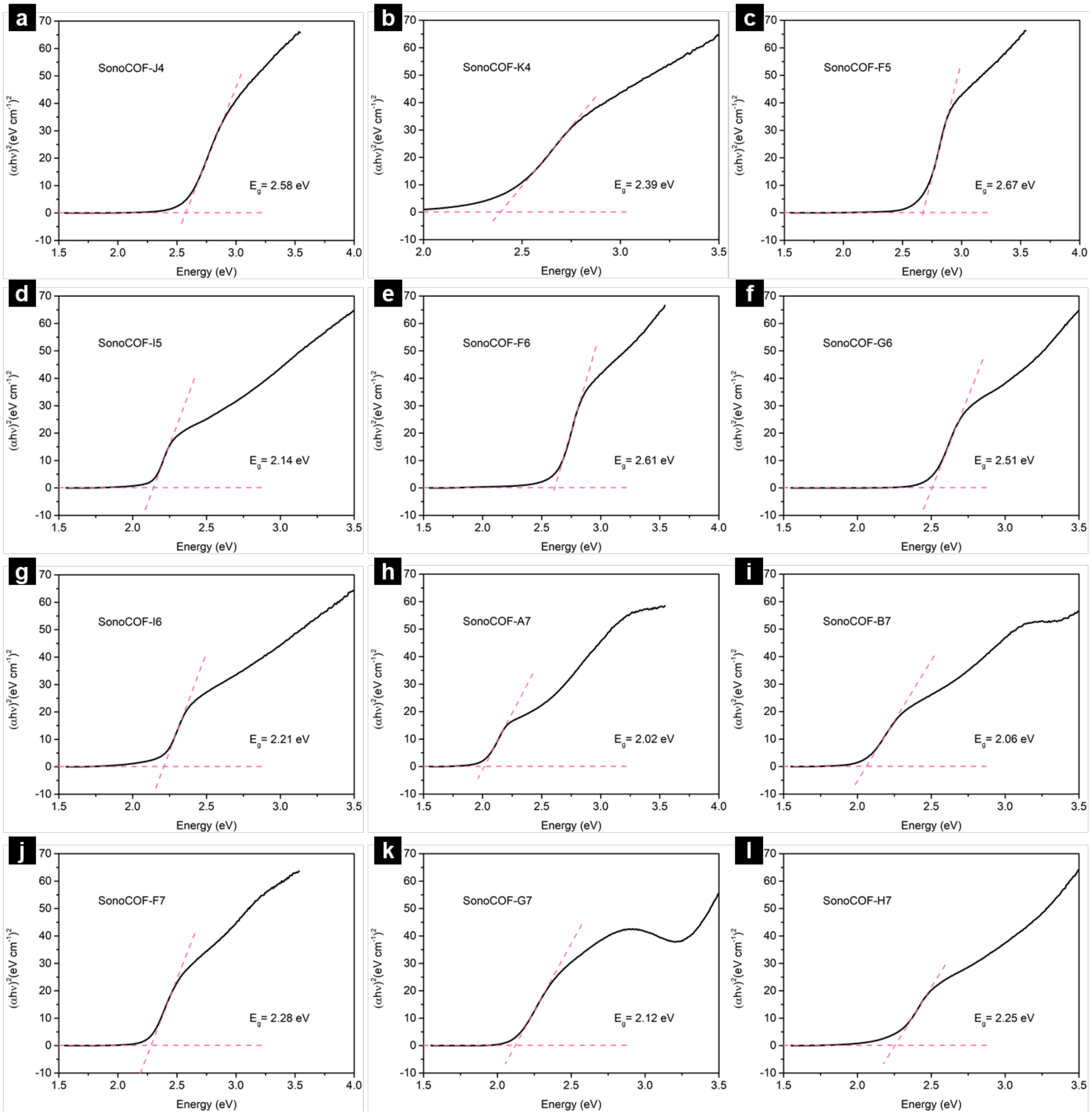

Figure S50. Kubelka-Munk-transformed reflectance spectra of sonoCOF-J4, -K4, -F5, -I5, -F6, -G6, -I6, -A7, -B7, -F7, -G7 and -H7.

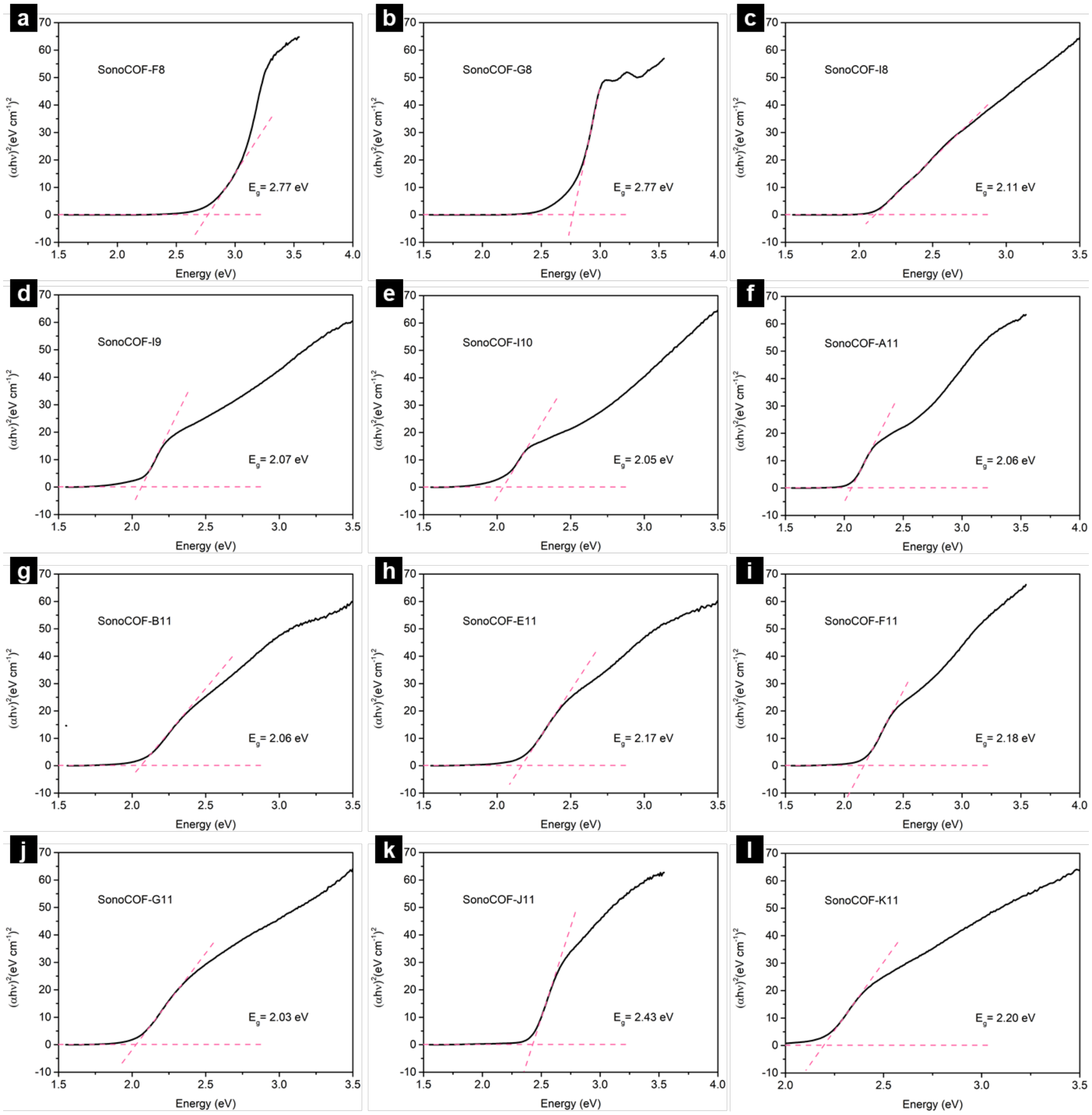

Figure S51. Kubelka-Munk-transformed reflectance spectra of sonoCOF-F8, -G8, -I8, -I9, -I10, -A11, -B11, -E11, -F11, -G11, -J11 and -K11.

3.9 Cyclic voltammetry (CV) spectra

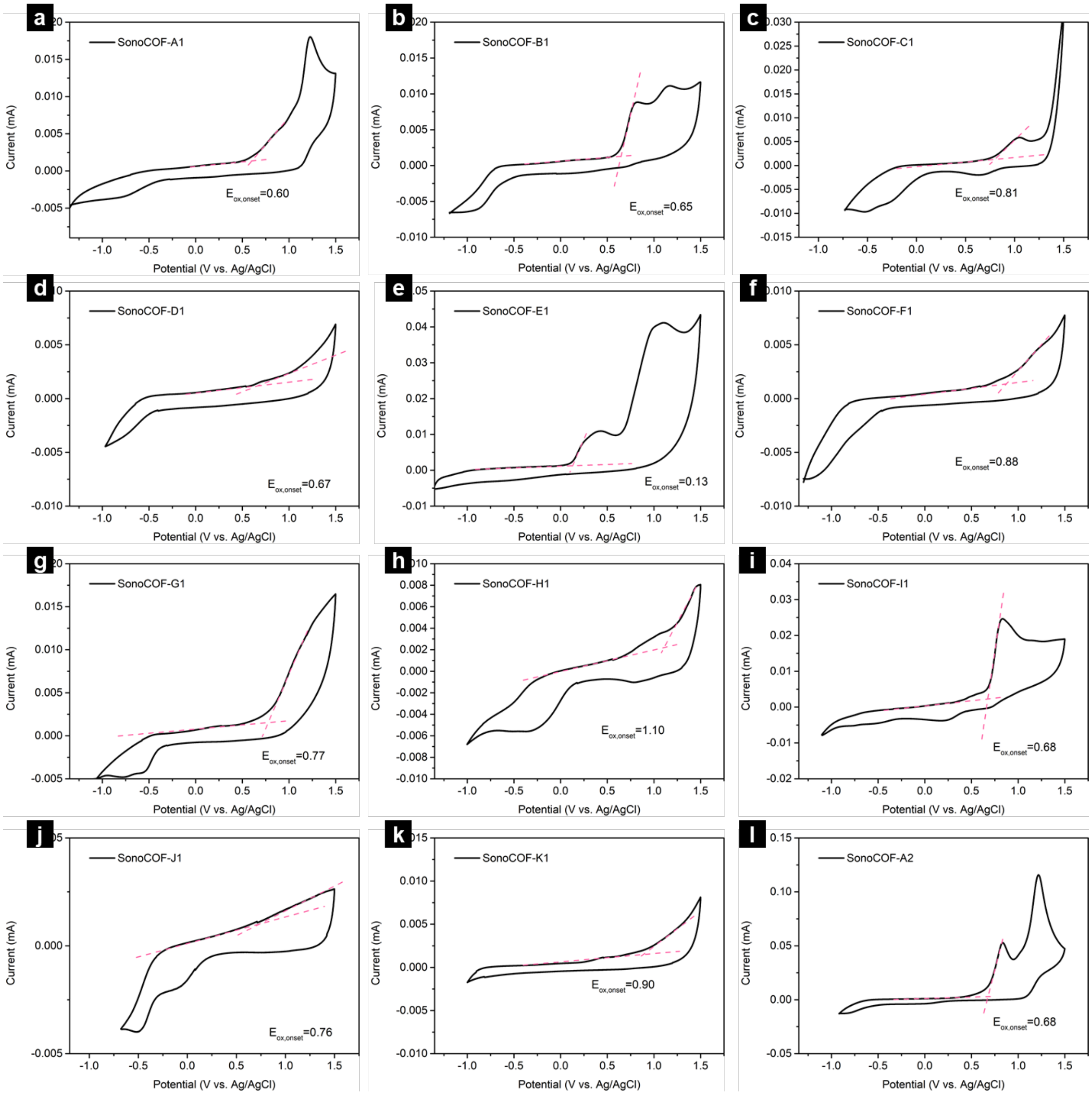

Figure S52. Oxidation onset potential of sonoCOFs A1-K1 and sonoCOF-A2 measured by cyclic voltammetry.

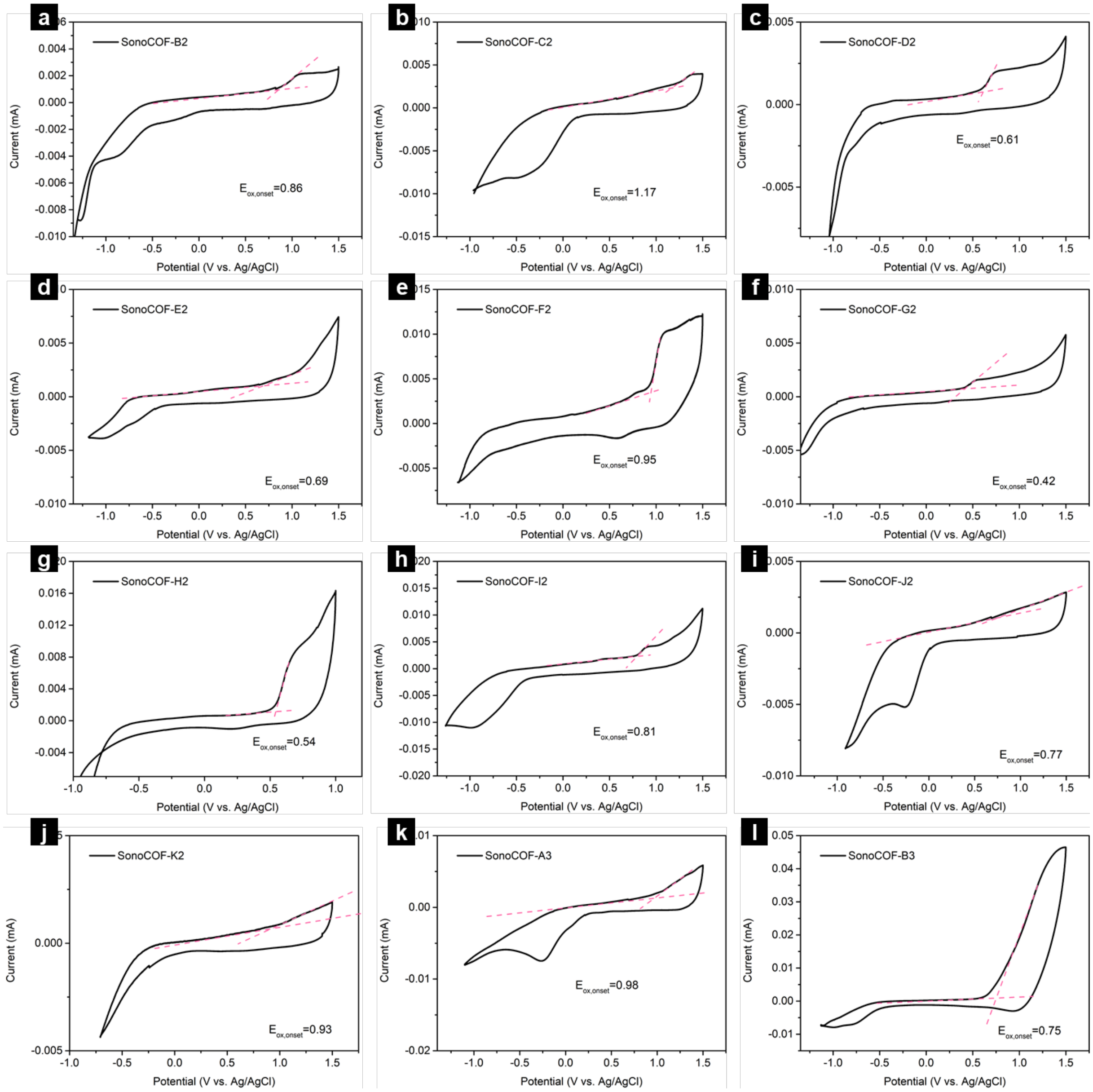

Figure S53. Oxidation onset potential of sonoCOFs B2-K2 and sonoCOF-A3 and -B3 measured by cyclic voltammetry.

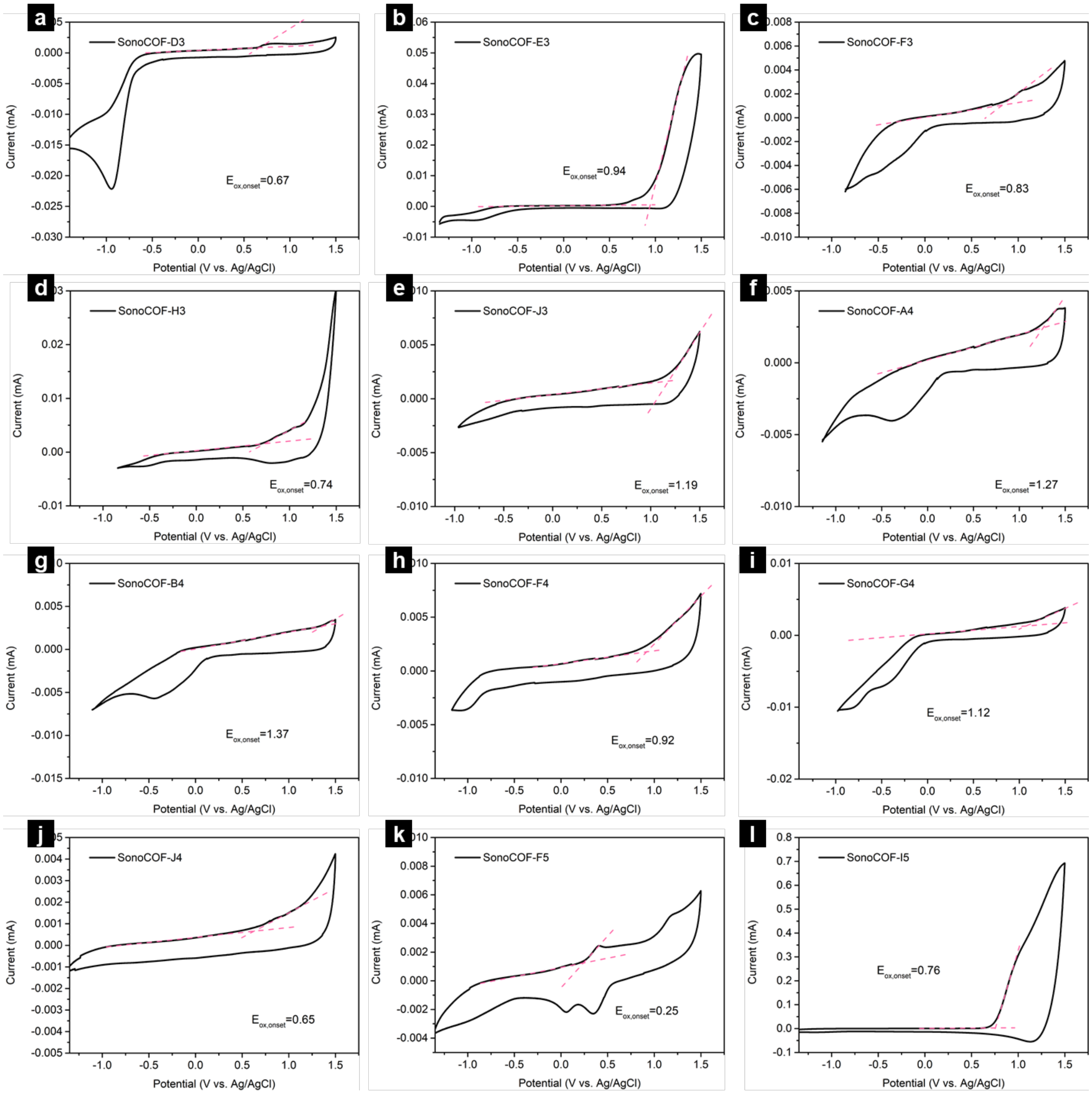

Figure S54. Oxidation onset potential of sonoCOF-D3, -E3, -F3, -H3, -J3, -A4, -B4, -F4, -G4, J4, F5 and I5 measured by cyclic voltammetry.

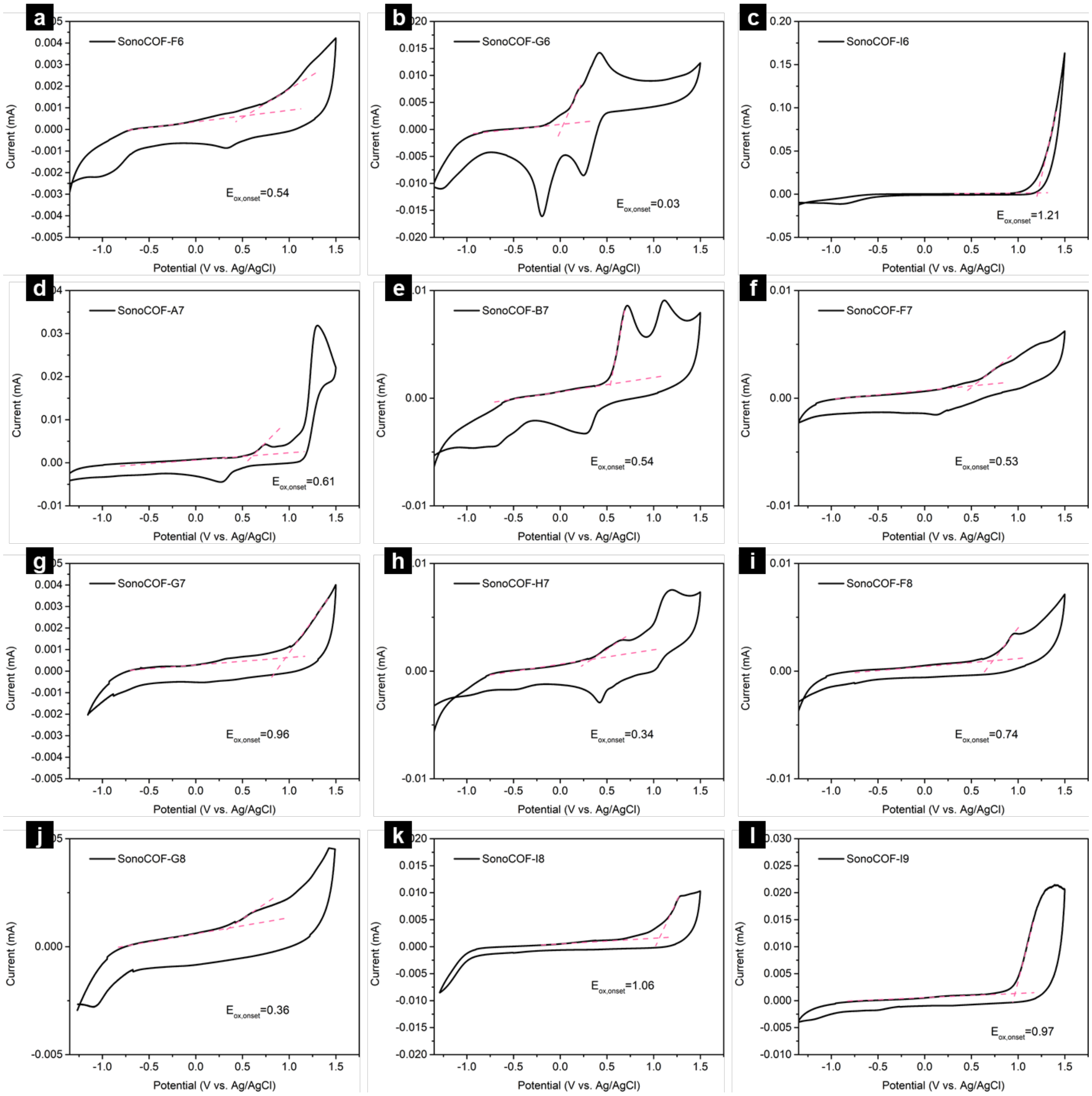

Figure S55. Oxidation onset potential of sonoCOF-F6, -G6, -I6, -A7, -B7, -F7, -G7, -H7, F8, G8, I8 and I9 measured by cyclic voltammetry.

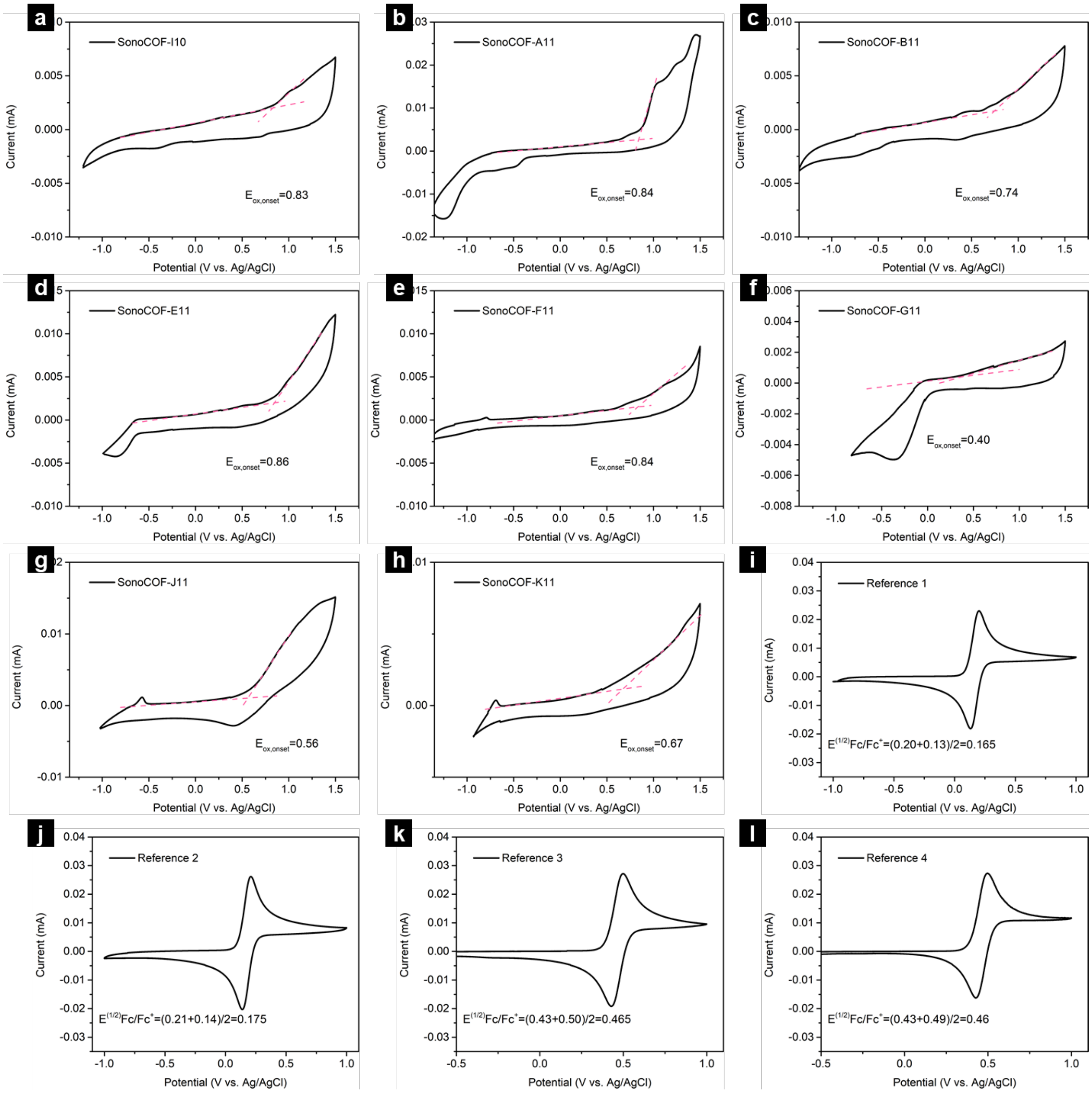

Figure S56. Oxidation onset potential of sonoCOF-I10, -A11, -B11, -E11, -F11, -G11, -J11, and K11 measured by cyclic voltammetry. Ferrocenium/ferrocene (Fc/Fc^{+}) redox couple was used as an external potential reference (Reference 1 is for sonoCOF-A1, B1, D1, E1, I1, K1, A2, D2, F4, I5, I6, I8, J11 and K11. Reference 2 is for sonoCOF-F1, G1, E2, G2, I2, J4, F5, F6, G6, A7, B7, F7, G7, H7, F8, G8, I9, I10, A11, B11, E11and F11. Reference 3 is for sonoCOF-C1, H1, J1, B2, C2, J2, K2, B3, D3, E3, F3, H3, J3, A4 and B4. Reference 4 is for F2, A3, G4 and G11).

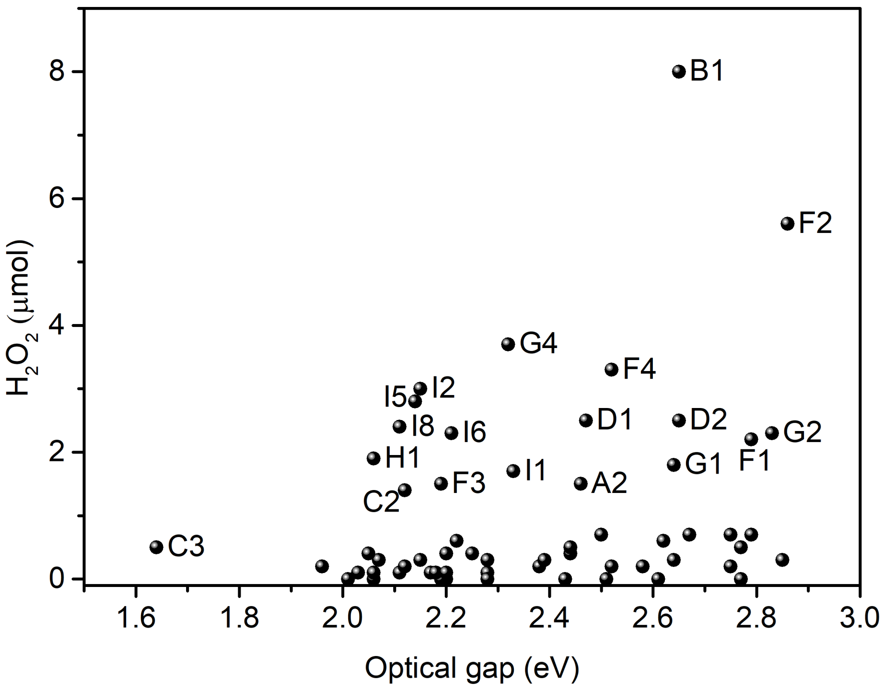

Figure S57. High-throughput test of H_2O_2 production of sonoCOFs in pure water under solar simulator plotted versus optical gaps. High-throughput test conditions: 3 mg COF, 5 ml water, air, simulated solar light for 1.5 h (Oriel Solar Simulator, 1.0 sun).

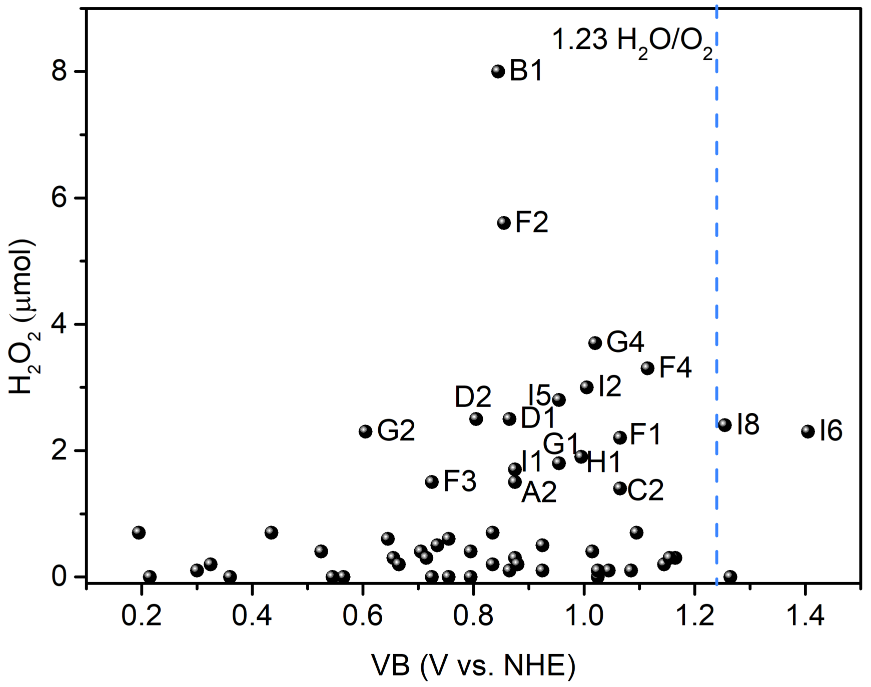

Figure S58. High-throughput test of H_2O_2 production of sonoCOFs in pure water under solar simulator plotted versus VB. High-throughput test conditions: 3 mg COF, 5 ml water, air, simulated solar light for 1.5 h (Oriel Solar Simulator, 1.0 sun).

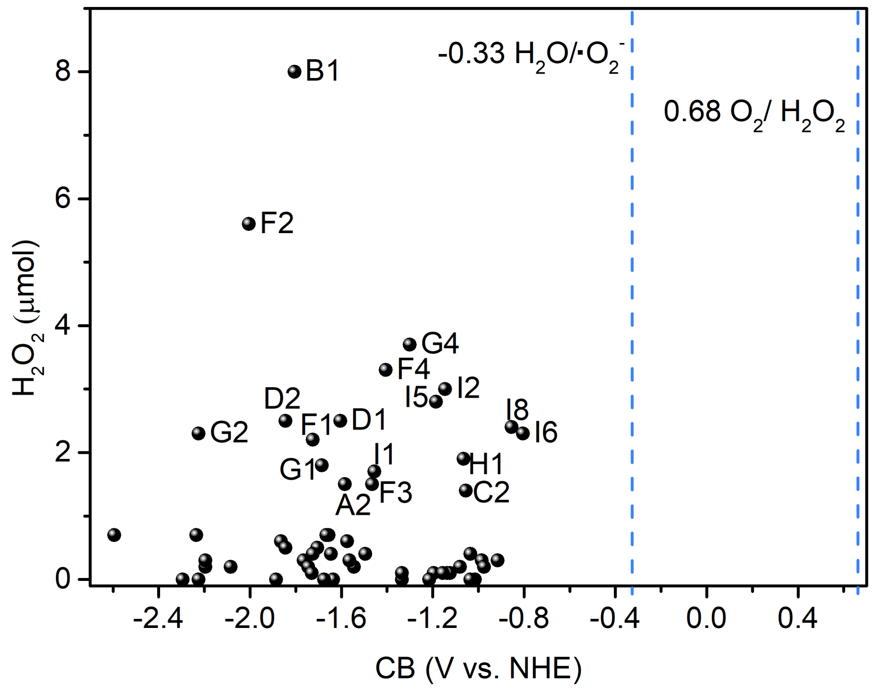

Figure S59. High-throughput test of H_2O_2 production of sonoCOFs in pure water under solar simulator plotted versus CB. High-throughput test conditions: 3 mg COF, 5 ml water, air, simulated solar light for 1.5 h (Oriel Solar Simulator, 1.0 sun).

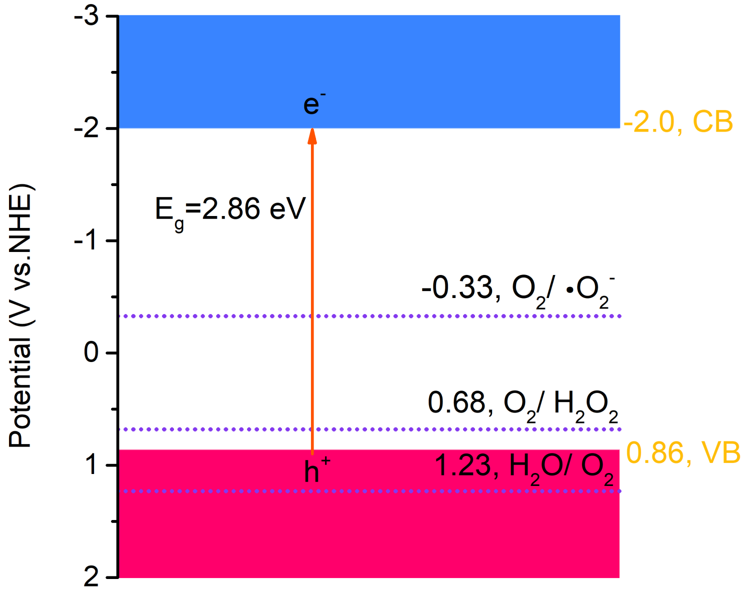

Figure S60. Band structure diagram for sonoCOF-F2 as determined from CV and UV measurements.

4. Photocatalytic hydrogen peroxide production

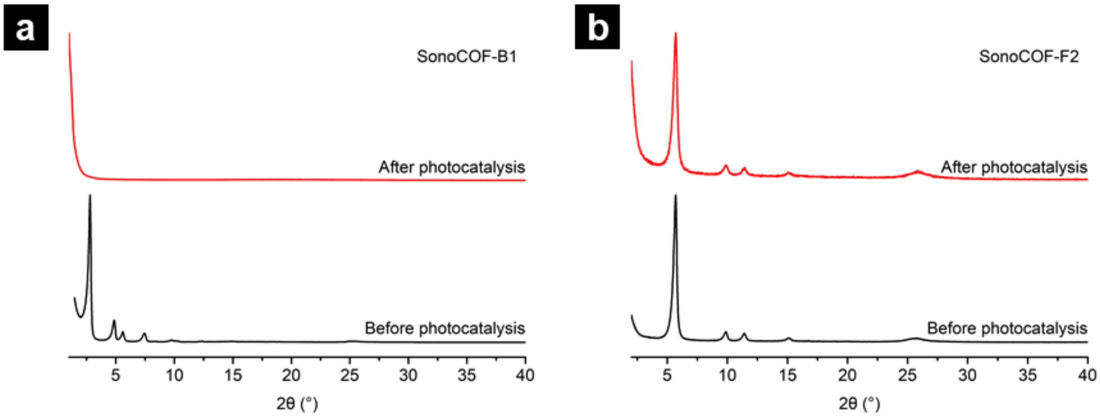

Figure S61. (a) PXRD patterns of sonoCOF-B1 and (b) sonoCOF-F2 before and after photocatalysis. Reaction conditions: 3 mg COF in 5 mL H_2O, air, 1.5 h illumination (Oriel Solar Simulator, 1.0 sun).

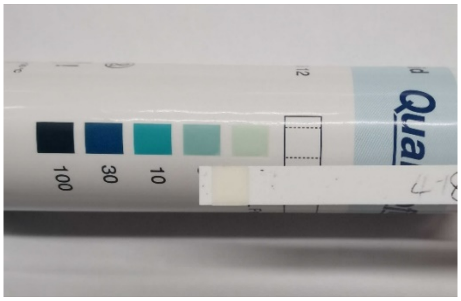

Figure S62. Image for photocatalytic H_2O_2 production for sonoCOF-F2 in neat water before illumination, as measured using Peroxide test sticks.

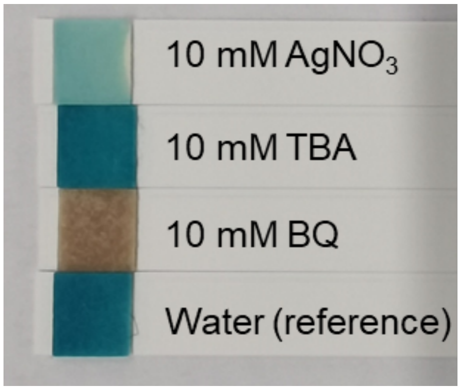

Figure S63. Images for photocatalytic H_2O_2 production for sonoCOF-F2 in neat water, with benzoquinone (BQ), tert-butyl alcohol (TBA) and $AgNO_3$ (5ml 10 mM aqueous solution, 3 mg COF), all with 1.5 h illumination (Oriel Solar Simulator, 1.0 sun), as measured using Peroxide test sticks. $AgNO_3$, tert-butyl alcohol (TBA), and benzoquinone (BQ) are as electron (e^-), hydroxyl radical (·OH), and superoxide radical (·O^{2-}) scavengers.

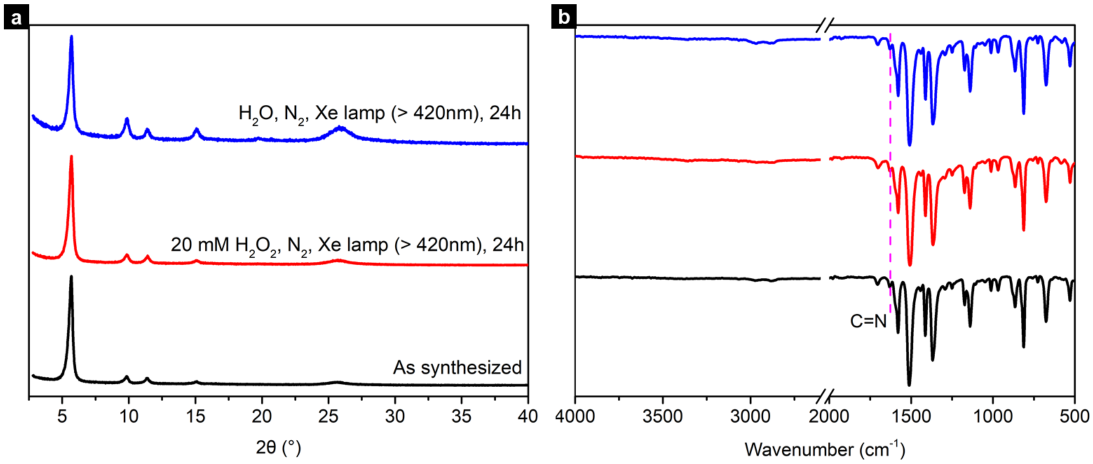

Figure S64. (a) PXRD and (b) FT-IR patterns of sonoCOF-F2 after irradiation in water or 20 mM H_2O_2 for 24 hours (N_2 atmosphere, 300 W Xe lamp, λ > 420 nm).

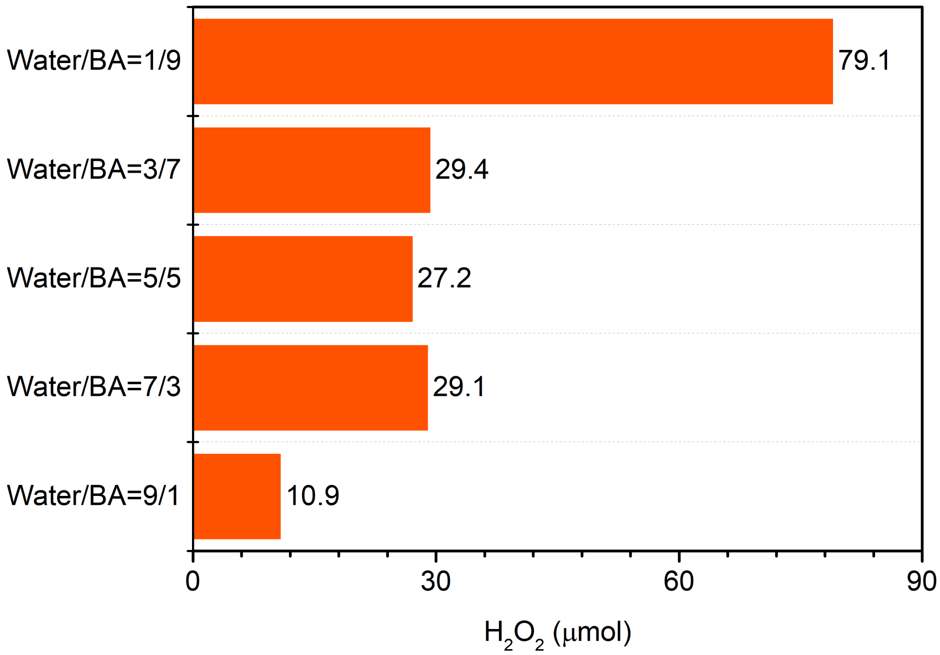

Figure S65. Photocatalytic H_2O_2 production for sonoCOF-F2 in mixtures of water and benzyl alcohol with different volume ratios (3 mg polymer, 5 mL solvents), all with 1.5 h illumination (Oriel Solar Simulator, 1.0 sun).

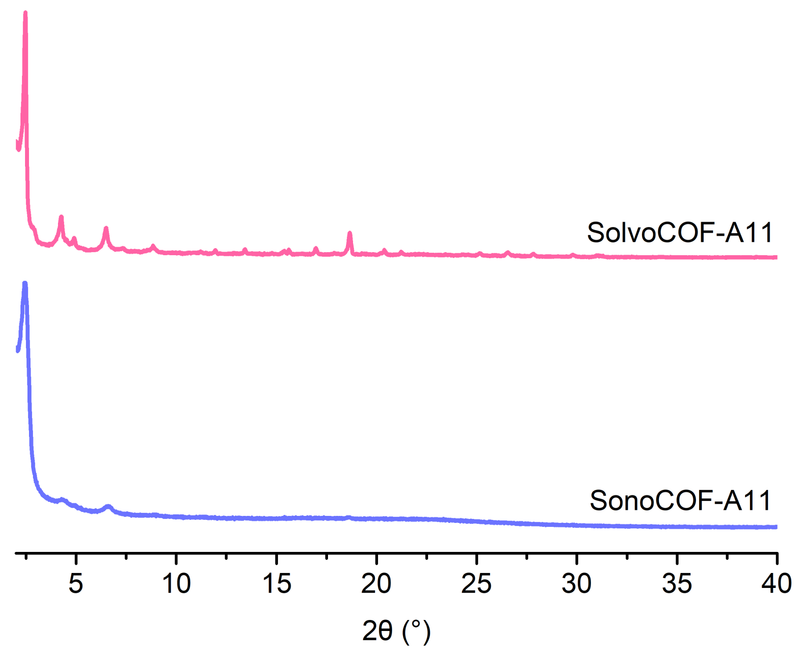

Figure S66. PXRD patterns of sonoCOF-A11 and solvoCOF-A11. Note that in this case, the solvothermal COF seems to have greater levels of crystallinity than the sonochemical COF, although its porosity level is somewhat lower (Fig. S57).

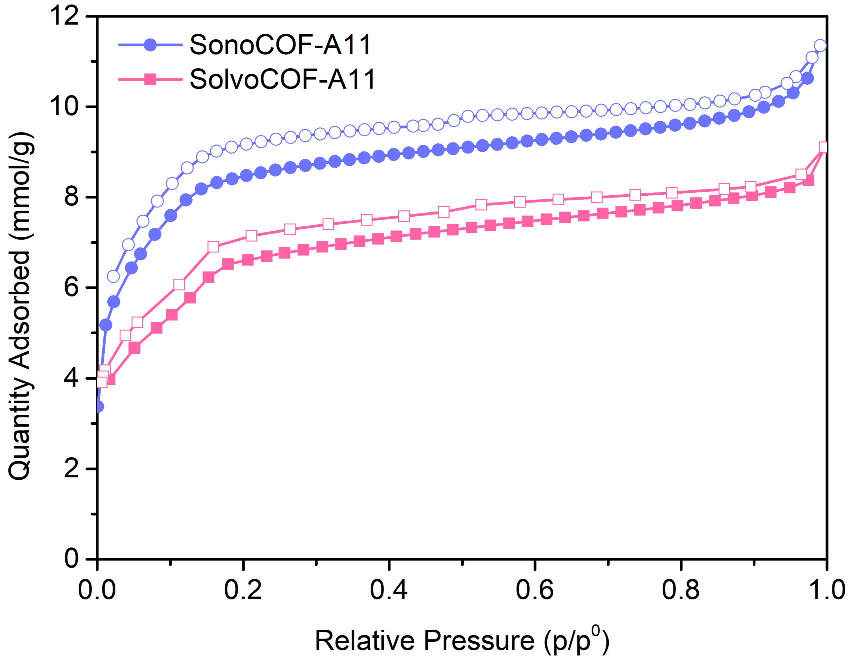

Figure S67. N_2 adsorption profiles of sonoCOF-A11 and solvoCOF-A11.

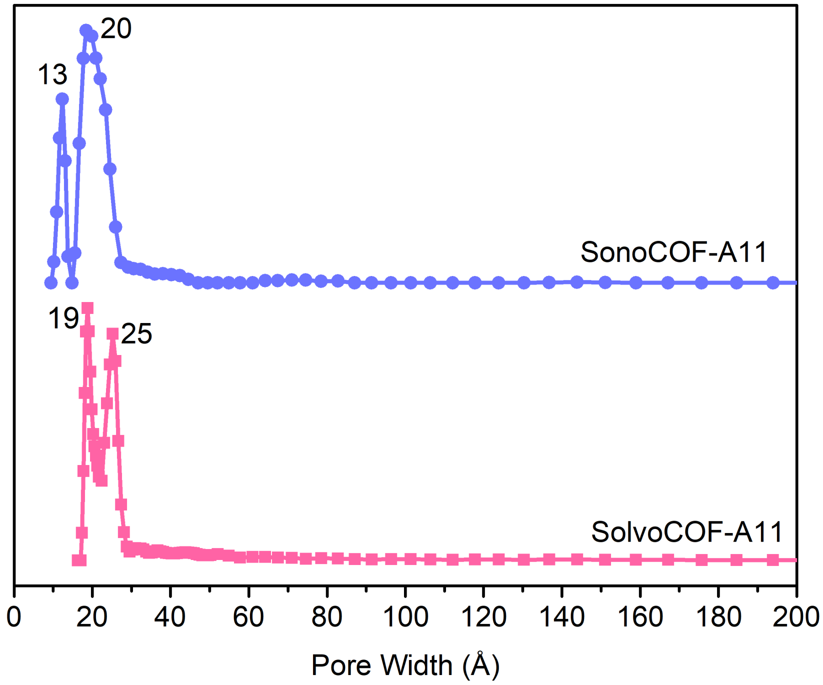

Figure S68. Pore size distribution profile of sonoCOF-A11 and solvoCOF-A11, as calculated by DFT.

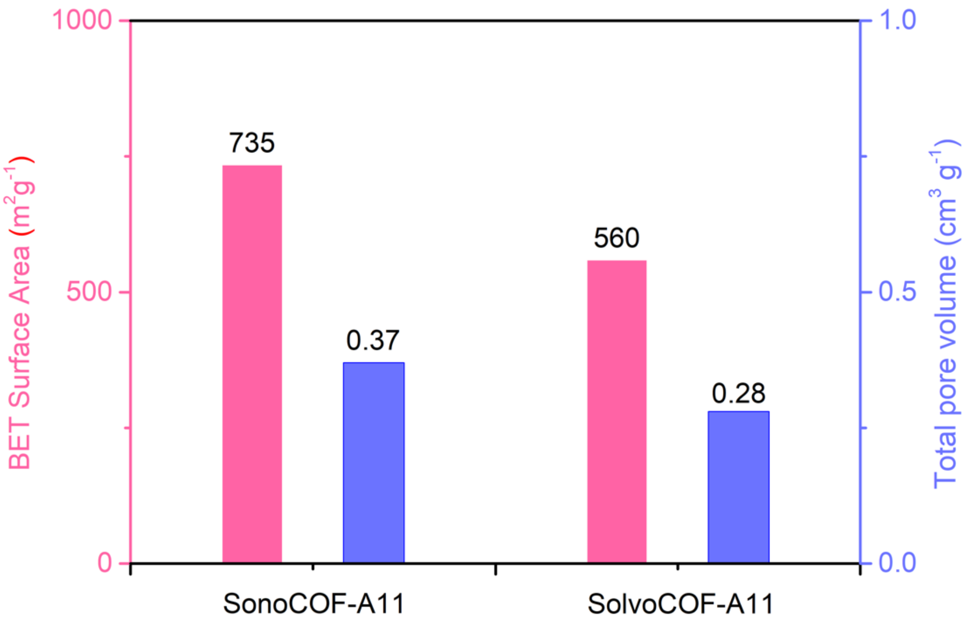

Figure S69. BET surface areas and total pore volumes of sonoCOF-A11 and solvoCOF-A11.

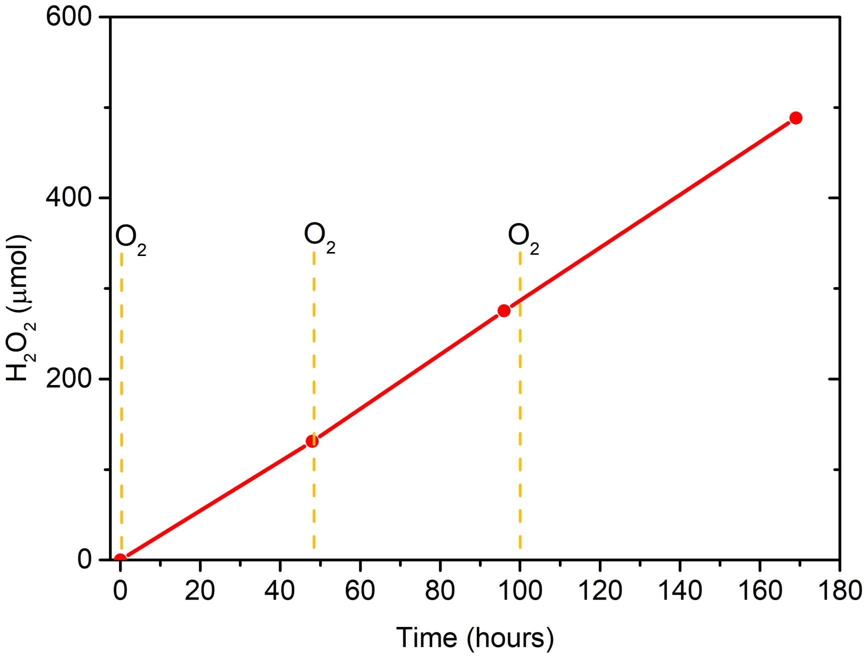

Figure S70. Long-term photocatalytic H_2O_2 production of sonoCOF-F2. Condition: 4.5 mL water, 0.5ml benzyl alcohol and 20 mg sonoCOF-F2; O_2; 300 W Xe lamp; λ > 420 nm.

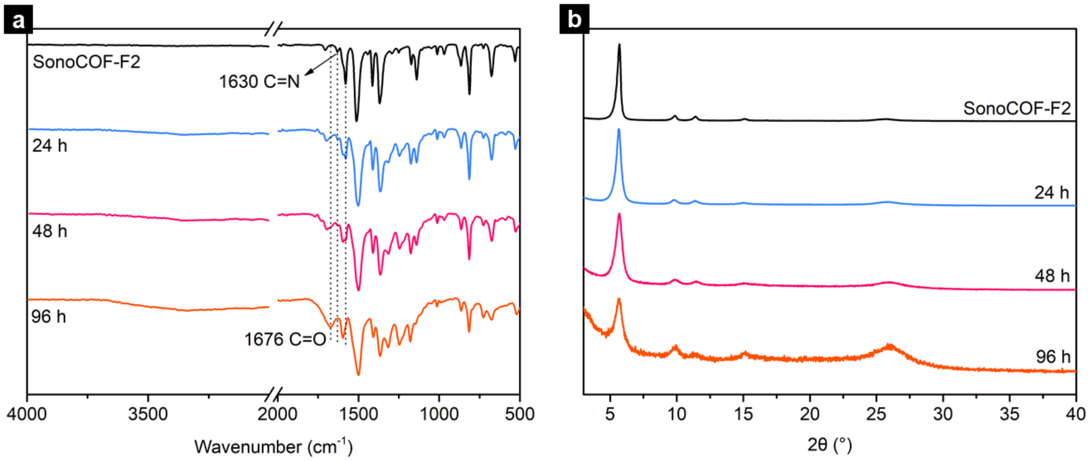

Figure S71. (a) FT-IR and (b) PXRD spectra of sonoCOF-F2 with different reaction time.

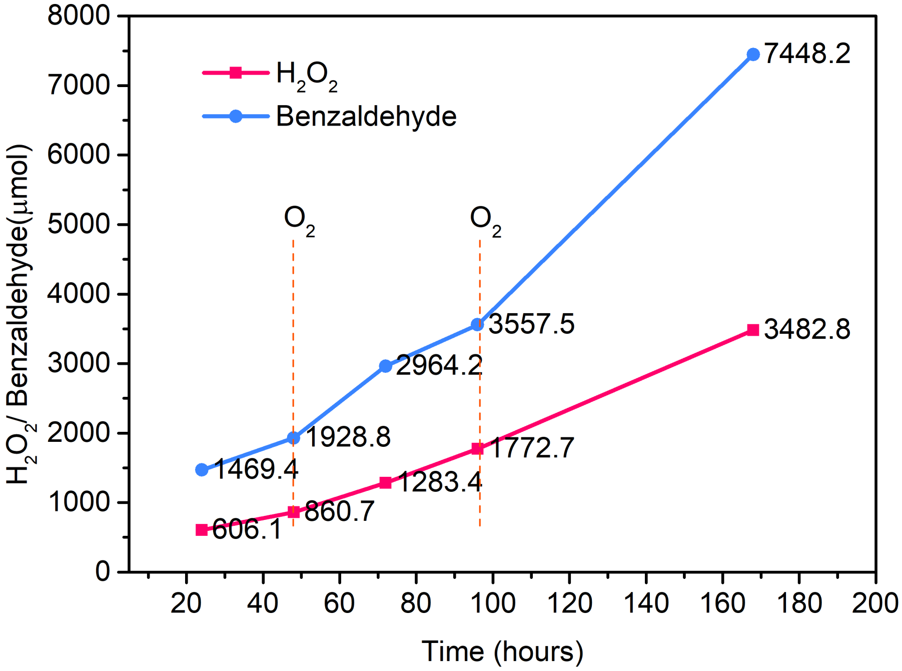

Figure S72. Long-term photocatalytic H_2O_2 production of sonoCOF-F2. Condition: 3 mL water, 27 ml benzyl alcohol and 50 mg sonoCOF-F2; O_2; 300 W Xe lamp; λ > 420 nm.

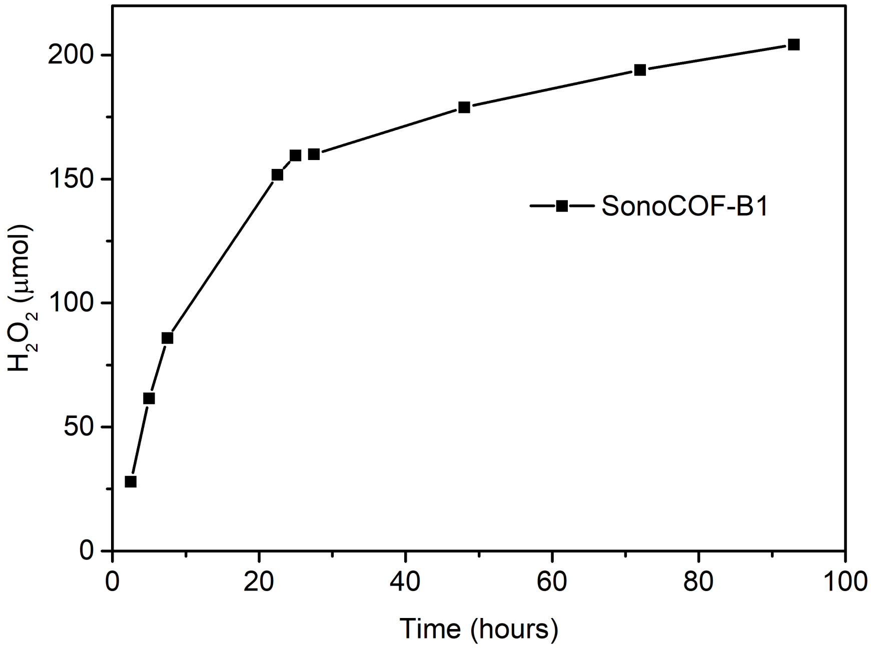

Figure S73. Long-term photocatalytic H_2O_2 production of sonoCOF-B1. Condition: 30 mL water, and 50 mg sonoCOF-B1; O_2; 300 W Xe lamp; λ > 420 nm.

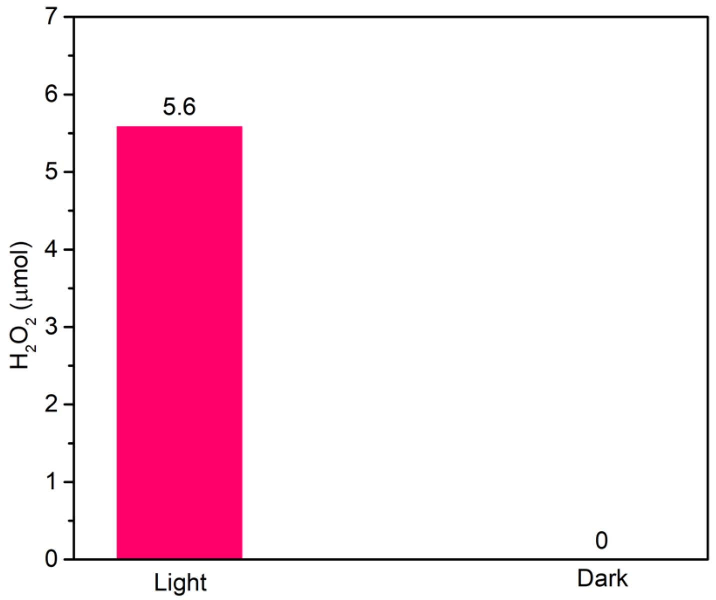

Figure S74. Reactions using sonoCOF-F2 under light and dark conditions. Light condition: 3 mg sonoCOF-F2 in 5 ml water, 1.5 h illumination (Oriel Solar Simulator, 1.0 sun). Dark condition: 3 mg COF in 5 ml water in dark for 48 h.

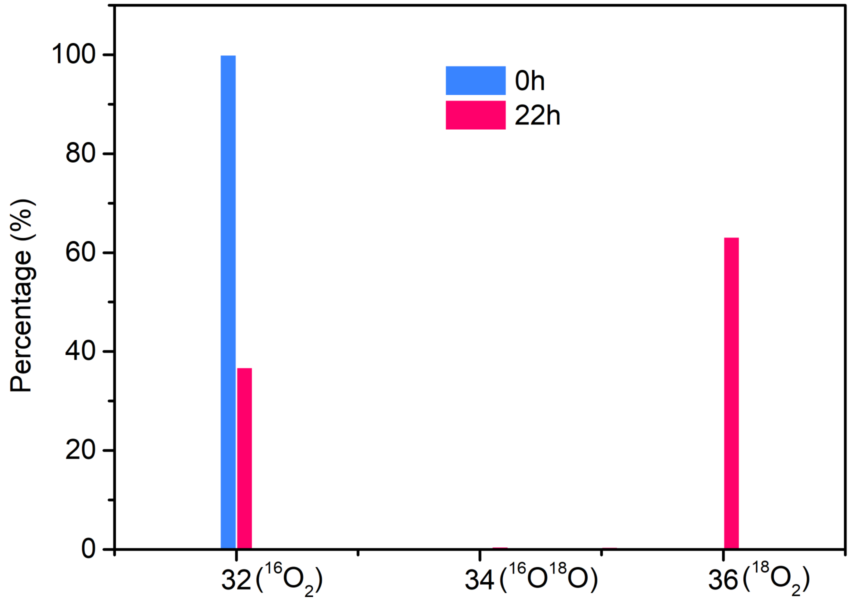

Figure S75. Isotopic $^{18}O_2$ labeling experiments with $^{18}O_2$. The signal of $^{16}O_2$ was from air during GC-MS injection.

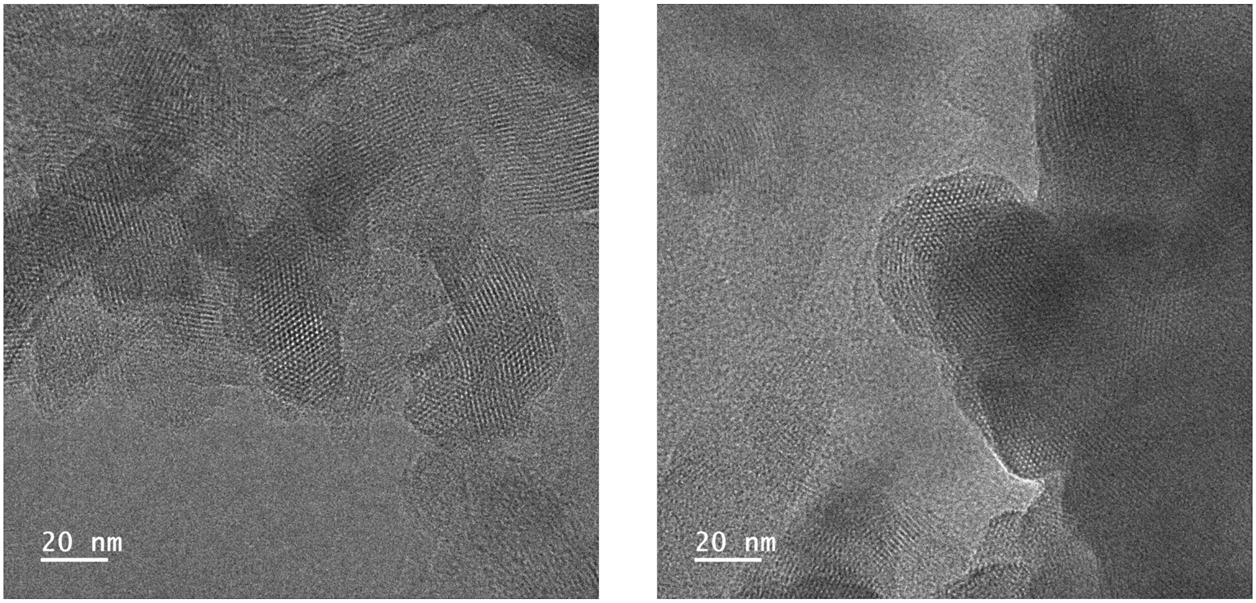

Figure S76. HRTEM image of sonoCOF-F2 before photocatalysis.

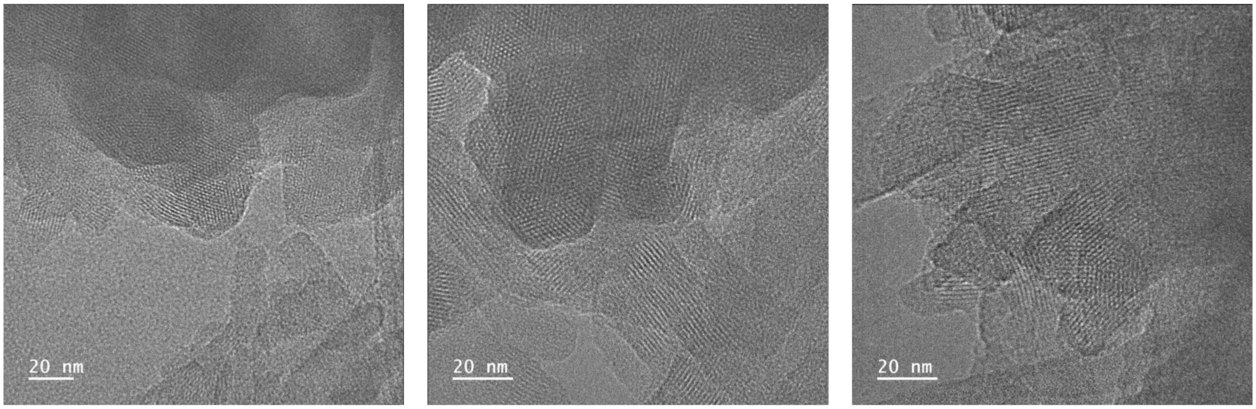

Figure S77. HRTEM image of sonoCOF-F2 before photocatalysis.

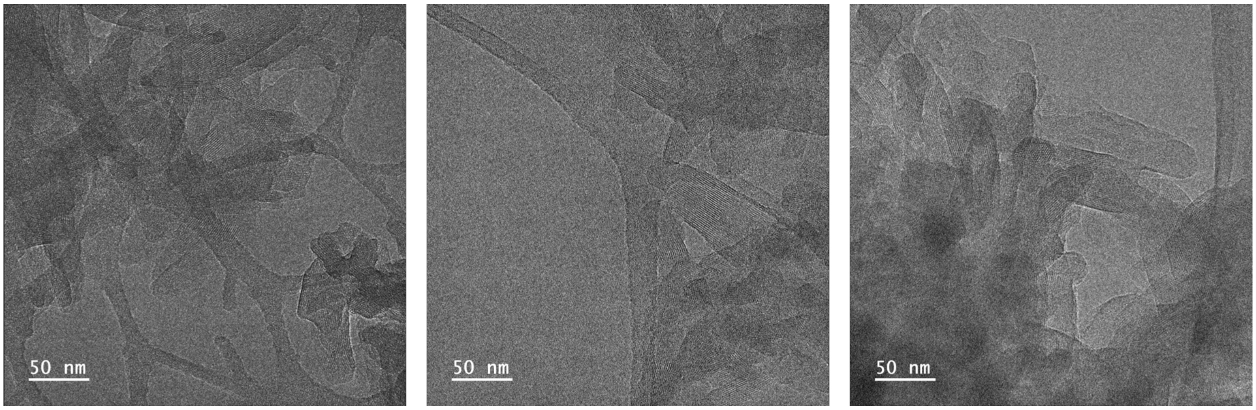

Figure S78. TEM image of sonoCOF-F2 after photocatalysis (96 h).

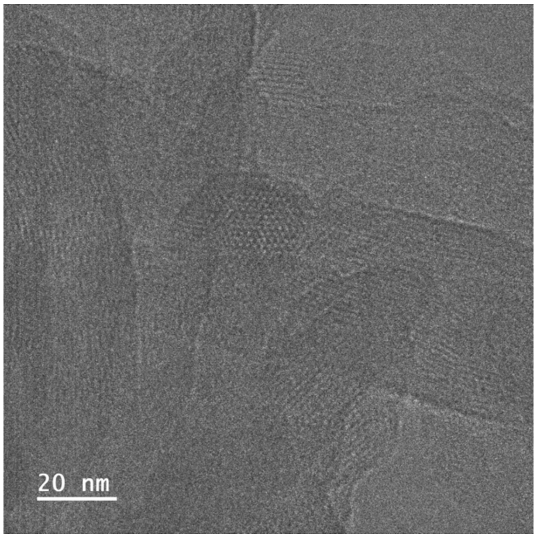

Figure S79. HRTEM image of sonoCOF-F2 after photocatalysis (96 h).

Table S3. Comparison of the photocatalytic H_2O_2 production activity of sonoCOF-F2 in pure water with other reported organic materials.

Photocatalysts	H_2O_2 yield / µmol	AQY / %[a]	Reaction conditions	Ref.
**SonoCOF-F2**	**197 (24 h)**	**4.8**	**λ > 420 nm**	**This work**
DE7-M	266 (24 h)	8.7	λ > 420 nm	1
RF523	61.6 (24 h)	~8.0	λ > 420 nm	2
RF-acid resins	91.0 (24 h)	8.5	λ > 420 nm	3
RF/P3HT resin	100 (18 h)	10.5	λ > 420 nm	4
CTF-BDDBN	70 (24 h)	–	λ > 420 nm	5
OCN-500	53 (10 h)	10.2	λ > 420 nm	6
Sb-SAPC15	470.5 (8 h)	17.6	λ > 420 nm	7
g-C_3N_4/PDI/rGO$_{0.05}$	29 (24 h)	6.1	λ > 420 nm	8
PEI/C_3N_4	4.2 (1 h)	2.21	λ > 420 nm	9
R_{370}-CN	17 (1 h)	~4.3	λ > 420 nm	10
PCNBA0.2Co5%	~35 (12 h)	8.0	λ > 420 nm	11

[a] All reported at 420 nm.

Table S4. Summary of reported sonoCOFs and their solvothermal analogs in the literature.

Number	SonoCOFs	COFs in literature	Ref.
1	SonoCOF-A1	TPB-DMTP-COF	12
2	SonoCOF-B1	SCOF-IC1	13
3	SonoCOF-C1	2,5-DhaTph	14
4	SonoCOF-D1	SCF-FCOF-2	15
5	SonoCOF-E1	sCOFs-IC2′	16
6	SonoCOF-F1	RT-COF-1	17
7	SonoCOF-G1	TBI-COF	18
8	SonoCOF-H1	TFPA-TAPB-COF	19
9	SonoCOF-I1	TAPB-TFP	20
10	SonoCOF-A2	COF-JLU5	21
11	SonoCOF-B2	TRITER-1	22
12	SonoCOF-C2	COF TzDa	23
13	SonoCOF-D2	SCF-FCOF-1	15
14	SonoCOF-E2	TzBA	24
15	SonoCOF-F2	N3-COF	25
16	SonoCOF-G2	TTI-COF	18
17	SonoCOF-H2	TPT-TPP-COF	26
18	SonoCOF-I2	TRIPTA	27
19	SonoCOF-J2	TaDA	28
20	SonoCOF-K2	TADAP	28
21	SonoCOF-B3	Py-1P COF	29
22	SonoCOF-C3	PyDHPh COF	30
23	SonoCOF-E3	0% BPy COF	31
24	SonoCOF-G3	PT-COF	32
25	SonoCOF-B4	ETTA-TPA COF	33
26	SonoCOF-F4	COF-432	34
27	SonoCOF-G4	SonoCOF-9	35
28	SonoCOF-F5	COF-LZU1	36
29	SonoCOF-I5	TpPa-1	37
30	SonoCOF-F6	COF-I	38
31	SonoCOF-G6	TFPB-BD	39
32	SonoCOF-I6	TpBD	40

33	SonoCOF-B7	TAPA-PDA COF	[41]
34	SonoCOF-F7	N-COF	[42]
35	SonoCOF-G7	TAPA-TFPT COF	[43]
36	SonoCOF-H7	TPA-COF	[44]
37	SonoCOF-F8	ACOF-1	[45]
38	SonoCOF-G8	N_3-COF (SSA)	[46]
39	SonoCOF-I8	COF-JLU2	[47]
40	SonoCOF-I9	TpPa-2	[37]
41	SonoCOF-I10	TpPa-Cl_2	[48]
42	SonoCOF-A11	TAPD-$(OMe)_2$ COF	[49]

5. Structural refinements against PXRD data

The Pawley refinement of the experimental PXRD was conducted to optimize the lattice parameters iteratively until the R_{WP} value converges.

Table S5. Fractional atomic coordinates for the unit cell of sonoCOF-B1.

SonoCOF-B1: Space group P6							
a= 36.1135 Å, b= 36.1135 Å, c= 3.4694 Å<br>α=β=90°, γ=120°, R_{wp}=9.56 %, R_p=7.76 %							
Atom	X (Å)	Y (Å)	Z (Å)	Atom	X (Å)	Y (Å)	Z (Å)
C1	0.62394	-0.68591	-0.01114	C12	0.54225	-0.48513	0.0147
C2	0.64281	-0.71007	-0.01103	C13	0.52739	-0.45772	0.01556
C3	0.62643	-0.6176	-0.01027	H14	0.45102	-0.57493	0.01609
C4	0.64574	-0.57787	-0.16324	H15	0.59095	-0.70076	-0.01101
C5	0.62654	-0.55427	-0.15747	H16	0.67514	-0.56519	-0.29831
C6	0.58725	-0.56999	-0.01143	H17	0.64173	-0.52393	-0.27755
C7	0.56764	-0.60951	0.14188	H18	0.5381	-0.62184	0.27326
C8	0.58715	-0.63284	0.14526	H19	0.57182	-0.66219	0.28279
N9	0.56898	-0.54443	-0.01349	H20	0.5078	-0.58948	0.03005
C10	0.53003	-0.55708	0.01548	H21	0.57505	-0.47321	0.0151
C11	0.51496	-0.52766	0.01476				

Table S6. Fractional atomic coordinates for the unit cell of sonoCOF-C1.

SonoCOF-C1: Space group P6							
a= 37.6239 Å, b= 37.6239 Å, c= 3.6515 Å<br>α=β=90°, γ=120°, R_{wp}=5.03 %, R_p=3.49 %							
Atom	X (Å)	Y (Å)	Z (Å)	Atom	X (Å)	Y (Å)	Z (Å)
C1	0.62393	-0.68588	-0.00299	C12	0.54225	-0.48518	0.01845
C2	0.64277	-0.71007	-0.0029	C13	0.52764	-0.4575	0.01918
C3	0.62651	-0.61752	-0.00233	O14	0.44499	-0.58474	0.01991
C4	0.64579	-0.57785	-0.15613	H15	0.59093	-0.7007	-0.0029
C5	0.6266	-0.55423	-0.15088	H16	0.67516	-0.56524	-0.29133
C6	0.58735	-0.56988	-0.00447	H17	0.64174	-0.52393	-0.27156
C7	0.56779	-0.60933	0.14991	H18	0.53827	-0.62158	0.28152
C8	0.58728	-0.63267	0.15374	H19	0.57198	-0.66198	0.29167

N9	0.56907	-0.54434	-0.00744	H20	0.50777	-0.58955	0.03347
C10	0.53005	-0.55709	0.0198	H21	0.57496	-0.4735	0.01892
C11	0.51484	-0.52773	0.01859	H22	0.41506	-0.59244	0.02004

Table S7. Fractional atomic coordinates for the unit cell of sonoCOF-D1.

SonoCOF-D1: Space group P6							
a= 40.3633 Å, b= 40.3633 Å, c= 4.1670 Å<br>α=β=90°, γ=120°, R_{wp}=6.06 %, R_p=4.66 %							
Atom	X (Å)	Y (Å)	Z (Å)	Atom	X (Å)	Y (Å)	Z (Å)
C1	0.62378	-0.68718	-0.08293	C12	0.54236	-0.48589	0.10199
C2	0.64399	-0.71014	-0.0818	C13	0.52783	-0.45774	0.10223
C3	0.62395	-0.62002	-0.07019	F14	0.44589	-0.58343	0.10587
C4	0.64115	-0.58082	-0.22393	H15	0.59063	-0.70302	-0.08103
C5	0.62123	-0.5579	-0.20061	H16	0.66965	-0.56797	-0.36787
C6	0.58338	-0.5738	-0.03294	H17	0.63488	-0.52758	-0.31671
C7	0.56566	-0.61304	0.11808	H18	0.53718	-0.62576	0.26106
C8	0.58589	-0.63571	0.10151	H19	0.57229	-0.66489	0.23569
N9	0.56523	-0.54821	-0.00958	H20	0.50598	-0.59077	0.14316
C10	0.5281	-0.55895	0.0862	F21	0.58325	-0.47232	0.11549
C11	0.51433	-0.52836	0.09638				

Table S8. Fractional atomic coordinates for the unit cell of sonoCOF-E1.

SonoCOF-E1: Space group P1							
a= 45.1442 Å, b= 44.6094 Å, c= 3.6209 Å<br>α=89.9715°, β=89.9931°, γ=120.0074°, R_{wp}=4.59 %, R_p=3.30 %							
Atom	X (Å)	Y (Å)	Z (Å)	Atom	X (Å)	Y (Å)	Z (Å)
H1	0.32132	0.20732	0.03704	C79	0.90321	0.73722	-0.43172
H2	0.63833	0.36796	0.16891	C80	0.89098	0.75961	-0.33484
H3	0.63217	0.26879	0.07398	C81	0.85615	0.74552	-0.24569
H4	0.89104	0.68472	-0.5214	C82	0.834	0.70963	-0.25008
H5	0.72655	0.36195	-0.16565	C83	0.91498	0.79735	-0.31783
H6	0.77096	0.13611	0.16436	N84	0.90372	0.8189	-0.2724
H7	0.03466	0.06398	0.23834	C85	0.92493	0.85577	-0.23264
H8	0.86012	0.63682	-0.11521	C86	0.95789	0.871	-0.07679

H9	0.93886	0.97238	0.00268	C87	0.97693	0.90686	-0.02089
H10	0.79135	0.10439	-0.16375	C88	0.96374	0.92847	-0.12509
H11	0.36609	0.22417	0.46893	C89	0.93084	0.91289	-0.28735
H12	0.03331	0.97112	-0.10426	C90	0.91156	0.87692	-0.33424
C13	0.68457	0.36931	-0.0055	C91	0.98349	0.96659	-0.04655
C14	0.64985	0.35247	0.09772	C92	0.01955	0.98437	-0.03013
C15	0.59233	0.29843	0.18573	H93	0.75199	0.41376	0.17231
C16	0.68225	0.25829	-0.20736	H94	0.78456	0.47702	0.0918
C17	0.69855	0.23852	-0.2168	H95	0.69398	0.47455	-0.33509
C18	0.73256	0.25269	-0.10037	H96	0.66205	0.41205	-0.27223
C19	0.75014	0.28696	0.03193	H97	0.7983	0.26953	-0.17978
C20	0.73378	0.30658	0.04791	H98	0.82119	0.57401	-0.10333
N21	0.48348	0.24686	0.29561	H99	0.77918	0.65089	-0.57072
C22	0.46263	0.21354	0.29302	H100	0.74016	0.58813	-0.54918
C23	0.79418	0.21842	-0.09751	H101	0.92993	0.74755	-0.5037
C24	0.77349	0.18401	0.02182	H102	0.8463	0.76233	-0.165
C25	0.78728	0.16214	0.06071	H103	0.80752	0.69948	-0.16912
C26	0.82199	0.17416	-0.0219	H104	0.94217	0.80679	-0.34917
C27	0.8425	0.20858	-0.14474	H105	0.96825	0.85509	0.01898
C28	0.82877	0.23049	-0.18016	H106	0.00134	0.91729	0.11956
C29	0.83686	0.15107	0.02172	H107	0.91997	0.92833	-0.38358
C30	0.81734	0.11546	-0.06145	H108	0.88623	0.86532	-0.45606
C31	0.83154	0.09394	-0.02532	C109	0.68177	0.31306	-0.03304
C32	0.86564	0.1076	0.09034	C110	0.70014	0.3493	-0.07379
C33	0.88513	0.14304	0.17659	C111	0.70441	0.40773	-0.04601
C34	0.87088	0.16452	0.14397	C112	0.57327	0.31423	0.076
C35	0.88066	0.08481	0.11265	C113	0.53773	0.29719	0.12327
N36	0.91355	0.0979	0.15313	C114	0.52014	0.26368	0.26832
C37	0.93098	0.07835	0.16455	C115	0.53878	0.24781	0.38514
C38	0.91503	0.04387	0.29022	C116	0.57451	0.26512	0.34704
C39	0.93275	0.02549	0.28454	N117	0.76282	0.51775	-0.16639
C40	0.96661	0.04106	0.14874	C118	0.75365	0.53863	-0.31168
C41	0.98261	0.07594	0.03298	C119	0.42519	0.19942	0.30448
C42	0.96504	0.0944	0.04617	C120	0.41139	0.22054	0.39957

C43	0.98471	0.02084	0.10965	C121	0.37596	0.20735	0.3903
C44	0.96645	0.98533	0.02078	C122	0.35358	0.17282	0.28279
H45	0.65625	0.2468	-0.31019	C123	0.36749	0.15155	0.1948
H46	0.6849	0.21215	-0.31912	C124	0.40293	0.16469	0.20776
H47	0.77593	0.29804	0.13927	C125	0.31605	0.15937	0.25208
H48	0.74753	0.33227	0.16816	C126	0.30372	0.18088	0.12082
H49	0.47224	0.19579	0.25433	C127	0.26853	0.16819	0.08382
H50	0.74685	0.17436	0.0927	C128	0.24487	0.1337	0.17547
H51	0.86909	0.21847	-0.21887	C129	0.25699	0.11215	0.30915
H52	0.84512	0.25686	-0.27526	C130	0.29223	0.12488	0.34779
H53	0.81613	0.06673	-0.09605	C131	0.20785	0.12073	0.1256
H54	0.91136	0.154	0.27159	N132	0.1853	0.08858	0.19708
H55	0.88637	0.19157	0.21934	C133	0.14866	0.07234	0.15854
H56	0.86408	0.05719	0.0707	C134	0.13208	0.08973	0.01963
H57	0.8892	0.0314	0.39897	C135	0.09632	0.07249	-0.00955
H58	0.92002	0.99922	0.39022	C136	0.07631	0.03763	0.10047
H59	0.00824	0.08866	-0.082	C137	0.09299	0.02034	0.2373
H60	0.97742	0.12094	-0.05096	C138	0.12877	0.0376	0.26487
C61	0.63015	0.31607	0.11572	C139	0.03825	0.01948	0.07238
C62	0.64663	0.2967	0.05399	C140	0.02052	0.03728	0.14657
C63	0.69955	0.29253	-0.07066	H141	0.58543	0.33919	-0.06073
C64	0.73925	0.42668	0.05396	H142	0.52345	0.30947	0.03201
C65	0.75783	0.46274	0.01134	H143	0.52592	0.22252	0.51434
C66	0.7422	0.48069	-0.12962	H144	0.58805	0.25252	0.45089
C67	0.70723	0.46191	-0.22616	H145	0.72832	0.52901	-0.42605
C68	0.68864	0.42584	-0.18592	H146	0.42812	0.24727	0.48095
N69	0.74781	0.23105	-0.09744	H147	0.35106	0.12508	0.10405
C70	0.7805	0.24232	-0.12743	H148	0.41306	0.14803	0.131
C71	0.77774	0.57639	-0.32462	H149	0.25979	0.18519	-0.02285
C72	0.81192	0.59065	-0.20719	H150	0.23912	0.08556	0.38604
C73	0.8342	0.62652	-0.21677	H151	0.30082	0.10789	0.45802
C74	0.82274	0.64888	-0.34369	H152	0.20022	0.13874	0.02594
C75	0.78856	0.63446	-0.46352	H153	0.14626	0.1163	-0.07258
C76	0.76631	0.59859	-0.4539	H154	0.08437	0.08625	-0.12625

C77	0.84618	0.68708	-0.34604	H155	0.07838	0.99373	0.33127
C78	0.88102	0.70133	-0.43902	H156	0.14124	0.02411	0.37478

Table S9. Fractional atomic coordinates for the unit cell of sonoCOF-G1.

SonoCOF-G1: Space group P-6							
a= 32.616 Å, b= 32.616 Å, c= 3.9033 Å<br>α=β=90°, γ=120°, R_{wp}=11.56 %, R_p=6.88 %							
Atom	X (Å)	Y (Å)	Z (Å)	Atom	X (Å)	Y (Å)	Z (Å)
C1	1.63478	-0.63607	-1	C15	1.34286	-0.4724	-1
C2	1.60114	-0.60378	-1	C16	1.36607	-0.51041	-1
C3	1.63084	-0.54151	-1	C17	1.35593	-0.37032	-1
C4	1.59913	-0.51141	-1	H18	1.67864	-0.51585	-1
C5	1.53738	-0.54226	-1	H19	1.62259	-0.46359	-1
C6	1.50727	-0.60447	-1	H20	1.45968	-0.63024	-1
C7	1.53883	-0.63476	-1	H21	1.51422	-0.6826	-1
N8	1.50773	-0.50837	-1	H22	1.4198	-0.57539	-1
C9	1.60521	-0.69803	-1	H23	1.51273	-0.40756	-1
C10	1.45101	-0.52853	-1	H24	1.47227	-0.34069	-1
C11	1.42741	-0.4876	-1	H25	1.29533	-0.49119	-1
C12	1.46525	-0.42604	-1	H26	1.33609	-0.55773	-1
C13	1.4421	-0.38797	-1	N27	0.29662	0.60753	0
C14	1.38059	-0.41069	-1				

Table S10. Fractional atomic coordinates for the unit cell of sonoCOF-H1.

SonoCOF-H1: Space group P3							
a= 23.3451 Å, b= 23.3451 Å, c= 4.1355 Å<br>α=β=90°, γ=120°, R_{wp}=19.44 %, R_p=14.23 %							
Atom	X (Å)	Y (Å)	Z (Å)	Atom	X (Å)	Y (Å)	Z (Å)
N1	0.49243	0.98423	1.05568	C14	0.58069	0.49399	1.25442
C2	0.50507	0.94559	0.88801	C15	0.63096	0.36647	1.11913
C3	0.4609	0.87429	0.89695	N16	0.59854	0.29799	1.12013
C4	0.40118	0.84647	1.06009	H17	0.54773	0.9644	0.7373
C5	0.3597	0.77886	1.06156	H18	0.38659	0.87716	1.18556
C6	0.37676	0.73713	0.90312	H19	0.31383	0.7593	1.18328

C7	0.43698	0.76567	0.7424	H20	0.45238	0.73566	0.62221
C8	0.47841	0.83327	0.73786	H21	0.52418	0.85354	0.6094
C9	0.524	0.46978	1.0704	H22	0.45814	0.3897	0.76111
C10	0.50144	0.41031	0.90715	H23	0.51879	0.33249	0.78662
C11	0.53602	0.37678	0.92465	H24	0.65806	0.47987	1.42422
C12	0.59341	0.40146	1.1074	H25	0.59784	0.53913	1.38492
C13	0.61513	0.46035	1.27399	N26	0.33333	0.66667	0.90431

Table S11. Fractional atomic coordinates for the unit cell of sonoCOF-I1.

SonoCOF-I1: Space group P-6							
a= 19.6666 Å, b= 19.6666 Å, c= 3.5851 Å<br>α=β=90°, γ=120°, R_{wp}=4.16 %, R_p=2.96 %							
Atom	X (Å)	Y (Å)	Z (Å)	Atom	X (Å)	Y (Å)	Z (Å)
C1	0.71558	0.41651	0	C11	0.37397	0.61685	0
C2	0.63138	0.38372	0	C12	0.42187	0.70652	0
C3	0.5625	0.15624	0	H13	0.70598	0.5543	0
C4	0.64137	0.52263	0	H14	0.64542	0.63618	0
C5	0.60659	0.5715	0	H15	0.41009	0.425	0
C6	0.52313	0.53849	0	H16	0.46688	0.34167	0
C7	0.47475	0.45442	0	H17	0.13964	0.63617	0
C8	0.50915	0.40512	0	O18	0.49502	0.74076	0
N9	0.49082	0.59217	0	H19	0.75283	0.47985	0
C10	0.16116	0.59392	0	H20	0.53381	0.65302	0

Table S12. Fractional atomic coordinates for the unit cell of sonoCOF-J1.

SonoCOF-J1: Space group R32							
a= 33.2557 Å, b= 33.2557 Å, c= 9.4303 Å<br>α=β=90°, γ=120°, R_{wp}=4.16 %, R_p=2.96 %							
Atom	X (Å)	Y (Å)	Z (Å)	Atom	X (Å)	Y (Å)	Z (Å)
C1	0.29187	0.30261	0.16985	H13	0.25782	0.30209	0.17291
C2	0.29251	0.26133	0.16924	H14	0.2527	0.18383	0.15765
C3	0.25049	0.21631	0.16941	H15	0.12716	0.20084	0.1319
N4	0.45669	0.33507	0.15067	H16	0.05203	0.1259	0.13114
C5	0.16792	0.16849	0.18582	H17	0.12493	0.05265	0.24348

C6	0.12691	0.16762	0.15613	H18	0.1995	0.12741	0.24806
C7	0.08524	0.12618	0.15576	H19	0.07409	0.00044	0.18423
C8	0.08399	0.08471	0.18566	C20	0.33333	0.26242	0.16667
C9	0.12515	0.08578	0.21804	C21	0.33333	0.34415	0.16667
C10	0.1666	0.1272	0.21943	H22	0.33333	0.37796	0.16667
C11	0.04014	0.04053	0.18398	H23	0.33333	0.22861	0.16667
C12	0.04024	0.00036	0.18407				

Table S13. Fractional atomic coordinates for the unit cell of sonoCOF-K1.

SonoCOF-K1: Space group R32							
a= 33.5568 Å, b= 33.5568 Å, c= 9.4430 Å<br>α=β=90°, γ=120°, R_{wp}=4.02 %, R_p=2.91 %							
Atom	X (Å)	Y (Å)	Z (Å)	Atom	X (Å)	Y (Å)	Z (Å)
C1	0.29187	0.30261	0.16985	N25	0.04024	1.00036	0.18407
C2	0.29251	0.26133	0.16924	H27	0.25782	0.30209	0.17291
C7	0.25049	0.21631	0.16941	H30	0.2527	0.18383	0.15765
N9	0.45669	0.33507	0.15067	H32	0.12716	0.20084	0.1319
C11	0.16792	0.16849	0.18582	H33	0.05203	0.1259	0.13114
C13	0.12691	0.16762	0.15613	H34	0.12493	0.05265	0.24348
C14	0.08524	0.12618	0.15576	H35	0.1995	0.12741	0.24806
C15	0.08399	0.08471	0.18566	H	0.07409	1.00044	0.18423
C16	0.12515	0.08578	0.21804	N3	0.33333	0.26242	0.16667
C17	0.1666	0.1272	0.21943	C6	0.33333	0.34415	0.16667
C23	0.04014	0.04053	0.18398	H29	0.33333	0.37629	0.16667

Table S14. Fractional atomic coordinates for the unit cell of sonoCOF-B2.

SonoCOF-B2: Space group P6							
a= 35.7527 Å, b= 35.7527 Å, c= 3.6700 Å<br>α=β=90°, γ=120°, R_{wp}=6.21 %, R_p=4.66 %							
Atom	X (Å)	Y (Å)	Z (Å)	Atom	X (Å)	Y (Å)	Z (Å)
N1	0.62504	-0.68581	0.00948	C11	0.5143	-0.52844	-0.01131
C2	0.64402	-0.70859	0.00952	C12	0.54236	-0.4858	-0.01106
C3	0.62635	-0.6193	0.00875	C13	0.52817	-0.4576	-0.01167
C4	0.64908	-0.5762	-0.03645	H14	0.44966	-0.57512	-0.01187

C5	0.62913	-0.55305	-0.03863	H15	0.68224	-0.56053	-0.07289
C6	0.58634	-0.57217	0.00339	H16	0.64687	-0.51995	-0.07568
C7	0.56346	-0.61525	0.05319	H17	0.53055	-0.63113	0.09711
C8	0.5833	-0.63853	0.05411	H18	0.56509	-0.67161	0.09439
N9	0.56786	-0.5465	-0.00002	H19	0.50576	-0.59104	-0.02666
C10	0.52868	-0.55869	-0.01162	H20	0.57524	-0.47443	-0.0111

Table S15. Fractional atomic coordinates for the unit cell of sonoCOF-C2.

SonoCOF-C2: Space group P6							
a= 38.8990 Å, b= 38.8990 Å, c= 3.7876 Å<br>α=β=90°, γ=120°, R_{wp}=5.27 %, R_p=4.02 %							
Atom	X (Å)	Y (Å)	Z (Å)	Atom	X (Å)	Y (Å)	Z (Å)
N1	0.62502	-0.68575	0.02213	C12	0.54238	-0.48576	-0.0091
C2	0.64395	-0.7086	0.0222	C13	0.52836	-0.45736	-0.00981
C3	0.62647	-0.61916	0.0209	O14	0.44356	-0.58502	-0.00991
C4	0.64922	-0.5761	-0.02813	H15	0.68237	-0.56049	-0.06693
C5	0.6293	-0.55289	-0.03146	H16	0.64704	-0.51981	-0.07166
C6	0.58653	-0.57194	0.01299	H17	0.53076	-0.63073	0.11481
C7	0.56364	-0.61496	0.06747	H18	0.56522	-0.67134	0.11316
C8	0.58345	-0.6383	0.06942	H19	0.50595	-0.59096	-0.03067
N9	0.56806	-0.54626	0.00802	H20	0.5752	-0.47454	-0.00916
C10	0.52884	-0.55857	-0.00989	H21	0.41359	-0.59226	-0.00974
C11	0.51426	-0.52845	-0.00946				

Table S16. Fractional atomic coordinates for the unit cell of sonoCOF-D2.

SonoCOF-D2: Space group P6							
a= 38.7536 Å, b= 38.7536 Å, c= 4.0084 Å<br>α=β=90°, γ=120°, R_{wp}=7.03 %, R_p=5.48 %							
Atom	X (Å)	Y (Å)	Z (Å)	Atom	X (Å)	Y (Å)	Z (Å)
N1	0.625	-0.68618	-0.05769	C11	0.51458	-0.52835	0.07412
C2	0.64435	-0.70863	-0.05792	C12	0.54262	-0.48562	0.07738
C3	0.62556	-0.62	-0.05116	C13	0.52782	-0.45752	0.07778
C4	0.64817	-0.57661	-0.06722	F14	0.44597	-0.58388	0.08028
C5	0.62788	-0.55377	-0.05363	H15	0.68152	-0.5604	-0.08957

C6	0.58499	-0.5735	-0.02164	H16	0.64529	-0.52031	-0.06501
C7	0.56208	-0.61689	-0.01086	H17	0.52881	-0.6333	0.00621
C8	0.58226	-0.63985	-0.0251	H18	0.56403	-0.67324	-0.01397
N9	0.56649	-0.54799	-0.00372	H19	0.50586	-0.59111	0.12571
C10	0.52838	-0.55914	0.06957	F20	0.58379	-0.47163	0.08755

Table S17. Fractional atomic coordinates for the unit cell of sonoCOF-E2.

SonoCOF-E2: Space group P1							
a= 44.9115 Å, b= 44.1431 Å, c= 3.9779 Å<br>α=83.3984°, β=96.9942°, γ=120.2114°, R_{wp}=6.37 %, R_p=5.41 %							
Atom	X (Å)	Y (Å)	Z (Å)	Atom	X (Å)	Y (Å)	Z (Å)
H1	0.31657	0.20663	-0.52282	C76	0.83219	0.70778	-0.17229
H2	0.89008	0.68462	-0.49119	C77	0.91248	0.79659	-0.27436
H3	0.7753	0.13875	0.34637	N78	0.90218	0.81734	-0.14571
H4	0.86095	0.63625	-0.07731	C79	0.92276	0.8545	-0.11647
H5	0.79472	0.10968	0.75381	C80	0.95917	0.87259	-0.14697
H6	0.36482	0.22477	-0.12041	C81	0.97794	0.90875	-0.11287
C7	0.68563	0.36729	-0.00611	C82	0.96086	0.92763	-0.04398
N8	0.65071	0.35012	-0.04921	C83	0.9245	0.90939	-0.00868
C9	0.59366	0.29675	-0.06787	C84	0.90577	0.87319	-0.04363
C10	0.68258	0.25753	0.20323	C85	0.98101	0.96605	-0.00806
C11	0.69968	0.23885	0.27015	N86	0.01592	0.9832	-0.0514
C12	0.73561	0.25602	0.32285	H87	0.75601	0.41021	0.0504
C13	0.75456	0.29252	0.30286	H88	0.78878	0.47394	-0.01172
C14	0.73755	0.31129	0.23544	H89	0.69321	0.47414	-0.194
N15	0.48385	0.24626	-0.19302	H90	0.66071	0.41128	-0.14052
C16	0.46273	0.21425	-0.2897	H91	0.79924	0.27277	0.59557
C17	0.79709	0.22241	0.52774	H92	0.82281	0.57312	-0.07717
C18	0.77697	0.18728	0.42607	H93	0.77655	0.64868	-0.49211
C19	0.79112	0.16542	0.43753	H94	0.73838	0.58562	-0.48282
C20	0.82571	0.17823	0.55119	H95	0.92827	0.74767	-0.48137
C21	0.84564	0.21333	0.65739	H96	0.8438	0.7602	-0.07726
C22	0.83147	0.23518	0.64532	H97	0.80564	0.69706	-0.07789
C23	0.8411	0.15525	0.55102	H98	0.93842	0.80658	-0.384

C24	0.82116	0.12014	0.6569	H99	0.97343	0.85915	-0.19182
C25	0.83531	0.09828	0.64429	H100	0.00593	0.92201	-0.1391
C26	0.86968	0.11104	0.5265	H101	0.91061	0.92316	0.04693
C27	0.88982	0.14618	0.42525	H102	0.87783	0.85942	-0.01444
C28	0.87568	0.16806	0.43726	C103	0.6834	0.31391	0.10267
C29	0.88374	0.08724	0.50211	N104	0.70182	0.34901	0.06988
N30	0.91489	0.09805	0.37432	C105	0.70577	0.4057	-0.04287
C31	0.9311	0.07739	0.31959	C106	0.57645	0.3157	-0.12364
C32	0.91213	0.04089	0.30007	C107	0.54041	0.29856	-0.16514
C33	0.92912	0.0221	0.23233	C108	0.52085	0.26234	-0.15736
C34	0.9653	0.03935	0.17947	C109	0.53779	0.24317	-0.09939
C35	0.98408	0.07585	0.19868	C110	0.57389	0.26026	-0.0559
C36	0.96701	0.09455	0.26592	N111	0.76444	0.51598	-0.14546
C37	0.98325	0.01945	0.09909	C112	0.75417	0.53671	-0.27533
N38	0.96482	0.98435	0.06721	C113	0.42495	0.20077	-0.30475
H39	0.65472	0.24346	0.16314	C114	0.41083	0.22167	-0.2031
H40	0.68501	0.21078	0.28045	C115	0.37496	0.20833	-0.21112
H41	0.78245	0.30666	0.33054	C116	0.35243	0.1738	-0.32232
H42	0.75274	0.33936	0.22	C117	0.36671	0.15303	-0.42711
H43	0.47244	0.19713	-0.36734	C118	0.40259	0.16637	-0.41727
H44	0.75048	0.17695	0.331	C119	0.31422	0.1594	-0.32298
H45	0.87207	0.22379	0.75428	C120	0.29999	0.18019	-0.42824
H46	0.84737	0.2621	0.72797	C121	0.2641	0.16687	-0.41936
H47	0.81939	0.07135	0.72657	C122	0.24171	0.13246	-0.30736
H48	0.9163	0.1565	0.33002	C123	0.25579	0.11153	-0.20537
H49	0.8915	0.19473	0.34632	C124	0.29166	0.12486	-0.21243
H50	0.8675	0.06066	0.59349	C125	0.20393	0.119	-0.293
H51	0.88425	0.02675	0.32848	N126	0.18278	0.08699	-0.1964
H52	0.91393	0.99404	0.21742	C127	0.14578	0.07093	-0.16103
H53	0.01194	0.08991	0.15795	C128	0.12885	0.09012	-0.10385
H54	0.98169	0.12262	0.27584	C129	0.09275	0.07306	-0.06024
C55	0.63193	0.31509	-0.01917	C130	0.07297	0.03656	-0.07132
N56	0.64846	0.29713	0.05662	C131	0.09017	0.01759	-0.12642
C57	0.70136	0.29403	0.18335	C132	0.12621	0.03471	-0.16805

C58	0.74213	0.42395	-0.00653	C133	0.03471	0.01824	-0.02234
C59	0.76084	0.46015	-0.04189	N134	0.01819	0.03622	0.05277
C60	0.74386	0.47881	-0.11621	H135	0.59089	0.34381	-0.13329
C61	0.70746	0.46071	-0.14793	H136	0.52746	0.31343	-0.20582
C62	0.68869	0.42455	-0.11339	H137	0.52329	0.2152	-0.07877
N63	0.75184	0.23537	0.3769	H138	0.58638	0.24499	-0.00985
C64	0.78301	0.2462	0.50411	H139	0.72827	0.5267	-0.38611
C65	0.77775	0.57467	-0.2798	H140	0.42757	0.24819	-0.11124
C66	0.81261	0.58948	-0.16938	H141	0.35015	0.12661	-0.52224
C67	0.83441	0.62553	-0.17205	H142	0.41293	0.14988	-0.49987
C68	0.82177	0.64749	-0.28477	H143	0.2538	0.18339	-0.5022
C69	0.78686	0.63253	-0.39612	H144	0.23901	0.08501	-0.11386
C70	0.7651	0.59649	-0.39367	H145	0.30176	0.1084	-0.12132
C71	0.84484	0.68582	-0.28471	H146	0.19426	0.13615	-0.37082
C72	0.87976	0.70077	-0.39551	H147	0.14335	0.1181	-0.08388
C73	0.90153	0.73681	-0.39275	H148	0.08027	0.08833	-0.01476
C74	0.88888	0.75863	-0.27916	H149	0.07573	0.98948	-0.13539
C75	0.854	0.74383	-0.16933	H150	0.13916	0.01983	-0.2081

Table S18. Fractional atomic coordinates for the unit cell of sonoCOF-G2.

SonoCOF-G2: Space group P-6							
a= 25.6211 Å, b= 25.6211 Å, c= 3.5291 Å<br>α=β=90°, γ=120°, R_{wp}=9.70 %, R_p=7.96 %							
Atom	X (Å)	Y (Å)	Z (Å)	Atom	X (Å)	Y (Å)	Z (Å)
C1	1.63585	-0.63708	-1	C15	1.34291	-0.47298	-1
C2	1.6022	-0.60477	-1	C16	1.36623	-0.51115	-1
C3	1.63216	-0.54234	-1	C17	1.35603	-0.37047	-1
C4	1.60032	-0.51209	-1	H18	1.68022	-0.51697	-1
C5	1.53826	-0.54311	-1	H19	1.62388	-0.46406	-1
C6	1.50799	-0.60563	-1	H20	1.4602	-0.63151	-1
C7	1.53973	-0.63603	-1	H21	1.51543	-0.68412	-1
N8	1.50849	-0.50908	-1	H22	1.42019	-0.57639	-1
N9	1.60671	-0.69725	-1	H23	1.51351	-0.40785	-1
C10	1.45153	-0.52933	-1	H24	1.47286	-0.34071	-1
C11	1.42783	-0.48823	-1	H25	1.29518	-0.49186	-1

C12	1.46582	-0.42642	-1	H26	1.33612	-0.55866	-1
C13	1.44257	-0.38819	-1	N27	0.29647	0.60728	0
C14	1.3808	-0.41101	-1				

Table S19. Fractional atomic coordinates for the unit cell of sonoCOF-I2.

SonoCOF-I2: Space group P-6							
a= 18.4996 Å, b= 18.4996 Å, c= 3.5434 Å<br>$\alpha=\beta=90°$, $\gamma=120°$, R_{wp}=4.33 %, R_p=3.14 %							
Atom	X (Å)	Y (Å)	Z (Å)	Atom	X (Å)	Y (Å)	Z (Å)
N1	0.71494	0.41521	0	C11	0.37441	0.6161	0
C2	0.63281	0.38196	0	C12	0.42304	0.70695	0
C3	0.56495	0.1608	0	H13	0.71105	0.54847	0
C4	0.64525	0.52033	0	H14	0.64977	0.63566	0
C5	0.61029	0.57015	0	H15	0.41027	0.42105	0
C6	0.52531	0.53645	0	H16	0.47107	0.33545	0
C7	0.47578	0.45088	0	H17	0.13714	0.636	0
C8	0.51074	0.40099	0	O18	0.49716	0.74157	0
N9	0.4927	0.59092	0	H19	0.53634	0.65254	0
C10	0.15889	0.59314	0				

Table S20. Fractional atomic coordinates for the unit cell of sonoCOF-J2.

SonoCOF-J2: Space group P-3221							
a= 36.8758 Å, b= 36.8758 Å, c= 6.9793 Å<br>$\alpha=\beta=90°$, $\gamma=120°$, R_{wp}=7.08 %, R_p=5.36 %							
Atom	X (Å)	Y (Å)	Z (Å)	Atom	X (Å)	Y (Å)	Z (Å)
C1	0.661	0.59455	0.07358	H32	0.78061	0.56148	0.12911
C2	0.66077	0.55545	0.13125	H33	0.50878	0.51703	0.23022
C3	0.70173	0.55687	0.13414	H34	0.43302	0.45461	0.2605
C4	0.74198	0.59478	0.08413	H35	0.48481	0.36278	0.2296
C5	0.74023	0.63291	0.0276	H36	0.55964	0.42584	0.19589
C6	0.70023	0.63274	0.02172	H37	0.84448	0.56585	0.06748
C7	0.62063	0.51469	0.18589	H38	0.91995	0.5787	0.07687
C8	0.78303	0.59349	0.09223	H39	0.97082	0.7223	0.0767
N9	0.82044	0.62977	0.06179	H40	0.89419	0.70777	0.06694

N10	0.58271	0.51349	0.18299	N41	0.37642	0.37668	0.25279
C11	0.54002	0.47642	0.21031	C42	1.33952	1.03983	0.18208
C12	0.86363	0.63598	0.06743	C43	1.29952	1.04083	0.1822
C13	0.50348	0.48305	0.23119	C44	1.29754	1.08149	0.1972
C14	0.46075	0.44793	0.24928	N45	1.21478	0.88068	0.12086
C15	0.45268	0.40346	0.24646	C46	1.33992	1.1624	0.22388
C16	0.48977	0.39663	0.23224	C47	0.38328	1.19907	0.2313
C17	0.53204	0.43247	0.21259	C48	0.39127	1.24206	0.2384
C18	0.87205	0.60032	0.07028	C49	1.35496	1.2503	0.24019
C19	0.91471	0.60741	0.07517	C50	1.31088	1.21316	0.23609
C20	0.95159	0.65162	0.07772	C51	1.3041	1.1706	0.22732
C21	0.94317	0.68778	0.07517	C52	1.36229	1.29338	0.24468
C22	0.90009	0.6795	0.0694	H53	0.37174	1.07071	0.19324
C23	0.40967	0.36762	0.24904	H54	1.26443	1.07832	0.1929
C24	0.99471	0.65918	0.08145	H55	0.41168	1.19335	0.22986
N25	0.40466	1.32673	0.24449	H56	0.42574	1.26974	0.24229
N26	1.02795	0.70152	0.0854	H57	1.28208	1.21824	0.23763
H27	0.63008	0.59581	0.06877	H58	1.26973	1.14289	0.22252
H28	0.70237	0.52676	0.17902	C59	1.26006	1	0.16667
H29	0.77053	0.66388	-0.01341	C60	1.3396	1	0.16667
H30	0.69986	0.663	-0.02351	H61	1.22846	1	0.16667
H31	0.62414	0.4857	0.2223	H62	0.37125	1	0.16667

Table S21. Fractional atomic coordinates for the unit cell of sonoCOF-K2.

SonoCOF-K2: Space group R32							
a= 33.2774 Å, b= 33.2774 Å, c= 10.4587 Å<br>α=β=90°, γ=120°, R_{wp}=3.40 %, R_p=2.45 %							
Atom	X (Å)	Y (Å)	Z (Å)	Atom	X (Å)	Y (Å)	Z (Å)
C1	0.29236	0.28879	0.16669	N25	0.03897	0.99695	0.16685
C2	0.29398	0.24801	0.16669	H27	0.25847	0.28608	0.16671
C7	0.25188	0.20361	0.16672	H30	0.25756	0.17379	0.16671
N9	0.45419	0.32178	0.1666	H32	0.13507	0.196	0.16678
C11	0.17161	0.15912	0.16676	H33	0.05668	0.12367	0.16683
C13	0.13125	0.16165	0.16679	H34	0.12177	0.04202	0.16679

C14	0.08859	0.12231	0.16681	H35	0.19912	0.11272	0.16675
C15	0.08537	0.07926	0.16681	N3	0.33333	0.24709	0.16667
C16	0.12549	0.07635	0.16679	C6	0.33333	0.33026	0.16667
C17	0.16806	0.11566	0.16677	H29	0.33333	0.36289	0.16667
C23	0.04045	0.03752	0.16684				

Table S22. Fractional atomic coordinates for the unit cell of sonoCOF-A3.

SonoCOF-A3: Space group P-1							
a= 23.8649 Å, b= 3.8186 Å, c= 25.637 Å<br>α=86.9149°, β=88.4825°, γ=89.9846°, R_{wp}=5.93 %, R_p=4.18 %							
Atom	X (Å)	Y (Å)	Z (Å)	Atom	X (Å)	Y (Å)	Z (Å)
C1	0.55501	0.13061	0.41414	C56	0.47809	-0.17902	0.69833
C2	0.49866	0.05076	0.4237	C57	0.46831	-0.19368	0.75383
C3	0.48074	0.02204	0.47858	C58	0.50479	-0.04097	0.79183
C4	0.57657	0.0367	0.50981	C59	0.55151	0.13669	0.77139
C5	0.59277	0.12365	0.45587	C60	0.56171	0.16052	0.71613
C6	0.45975	0.01968	0.37963	C61	0.28932	0.19586	0.4162
C7	0.40366	-0.00884	0.39342	C62	0.23265	0.18099	0.4213
C8	0.38359	0.02164	0.44697	C63	0.20708	0.00778	0.4653
C9	0.47437	0.02143	0.3231	C64	0.24141	-0.15735	0.50414
C10	0.32517	0.02758	0.4551	C65	0.29824	-0.15175	0.49945
C11	0.52175	-0.16102	0.30174	N66	0.14882	0.00327	0.46824
C12	0.53166	-0.17905	0.24629	N67	0.50651	-0.05558	0.15154
C13	0.49556	-0.02571	0.20801	C68	0.118	-0.09269	0.50915
C14	0.44914	0.15639	0.22811	C69	0.05795	-0.07955	0.50409
C15	0.43892	0.18427	0.28333	C70	0.46897	-0.04979	0.11339
C16	0.71047	0.15845	0.58509	C71	0.48513	-0.05346	0.056
C17	0.76716	0.13685	0.58109	C72	0.03187	0.01683	0.45536
C18	0.79294	-0.03247	0.53688	C73	0.97477	0.03127	0.44986
C19	0.759	-0.18719	0.49672	C74	0.54045	-0.05646	0.03958
C20	0.70212	-0.17638	0.50043	C75	0.55632	-0.05221	0.98504
N21	0.85111	-0.04683	0.53472	H76	0.43057	0.17844	0.62775
N22	0.49388	-0.06666	0.84835	H77	0.36452	0.19377	0.55463
C23	0.88118	-0.03623	0.49069	H78	0.62612	-0.08957	0.63945

C24	0.94122	-0.05348	0.49529	H79	0.44999	-0.31842	0.67037
C25	0.53142	-0.05972	0.88648	H80	0.43164	-0.33647	0.76861
C26	0.51527	-0.05831	0.94388	H81	0.58046	0.26617	0.79972
C27	0.96727	-0.15171	0.54392	H82	0.5986	0.30474	0.70215
C28	0.02435	-0.16824	0.54932	H83	0.30731	0.34047	0.38148
C29	0.45994	-0.05846	0.96031	H84	0.20693	0.31399	0.39043
C30	0.44409	-0.04953	0.01483	H85	0.22315	-0.30211	0.53864
H31	0.57014	0.18978	0.37276	H86	0.32323	-0.29814	0.52935
H32	0.63628	0.17759	0.44583	H87	0.13512	-0.18306	0.54851
H33	0.37343	-0.04445	0.3601	H88	0.42427	-0.03642	0.12231
H34	0.54943	-0.30247	0.32985	H89	0.05794	0.08245	0.41993
H35	0.56806	-0.32568	0.23178	H90	0.57292	-0.06192	0.07115
H36	0.42047	0.28637	0.19956	O91	0.04976	-0.26405	0.59841
H37	0.4023	0.33251	0.29699	C92	0.01656	-0.42249	0.6398
H38	0.69222	0.29927	0.62006	O93	0.61206	-0.049	0.97009
H39	0.79271	0.26161	0.61292	C94	0.64836	0.13453	1.0061
H40	0.77794	-0.3283	0.46217	O95	0.9493	0.12563	0.40074
H41	0.67727	-0.31582	0.46961	C96	0.98233	0.28674	0.35948
H42	0.86335	-0.00511	0.44942	O97	0.38835	-0.0438	0.02977
H43	0.57614	-0.04888	0.87754	C98	0.3522	0.13844	-0.00655
H44	0.94114	-0.21841	0.57927	H99	-0.0104	-0.62591	0.62223
H45	0.42747	-0.06552	0.92874	H100	-0.00924	-0.22381	0.66018
C46	0.44547	0.12476	0.58611	H101	0.04378	-0.54561	0.67042
C47	0.50145	0.03699	0.57616	H102	0.66452	-0.04467	1.03757
C48	0.5193	0.01461	0.52119	H103	0.62699	0.35405	1.02633
C49	0.4236	0.0525	0.49007	H104	0.68325	0.24122	0.98235
C50	0.40776	0.13325	0.54436	H105	0.95499	0.4138	0.32936
C51	0.54023	-0.00672	0.62012	H106	1.00777	0.08896	0.33847
C52	0.59612	-0.04547	0.60617	H107	1.00961	0.48762	0.37727
C53	0.61641	-0.00693	0.55284	H108	0.31734	0.2474	0.01701
C54	0.52582	-0.00169	0.67665	H109	0.33595	-0.04226	-0.03776
C55	0.67489	-0.00165	0.54509	H110	0.37372	0.35623	-0.02708

Table S23. Fractional atomic coordinates for the unit cell of sonoCOF-C3.

SonoCOF-C3: Space group P2							
a= 24.3068 Å, b= 3.7917 Å, c= 24.5584 Å<br>α=90°, β=93.1405°, γ=90°, R_{wp}=4.72 %, R_p=3.67 %							
Atom	X (Å)	Y (Å)	Z (Å)	Atom	X (Å)	Y (Å)	Z (Å)
C1	0.55103	0.13116	0.41427	C26	0.51749	0.00865	0.94607
C2	0.49539	0.06652	0.42333	C27	0.97017	-0.08484	0.54681
C3	0.4795	0.06138	0.47807	C28	0.02772	-0.07904	0.55194
C4	0.57717	0.06662	0.51083	C29	0.4611	0.00758	0.95604
C5	0.59091	0.13092	0.45693	C30	0.44342	0.00863	0.0095
C6	0.45485	0.02157	0.37988	H31	0.56504	0.18408	0.37428
C7	0.39969	-0.01863	0.39267	H32	0.63253	0.18308	0.44645
C8	0.38222	0.02216	0.44578	H33	0.36927	-0.07948	0.36015
C9	0.46703	0.01199	0.32063	H34	0.53998	-0.31563	0.32967
C10	0.32131	0.01358	0.4529	H35	0.55919	-0.31138	0.23328
C11	0.51263	-0.1709	0.30184	H36	0.41196	0.29863	0.19789
C12	0.52326	-0.17364	0.24653	H37	0.39548	0.32398	0.2943
C13	0.48678	-0.01348	0.20813	H38	0.69951	0.32389	0.61968
C14	0.44009	0.16102	0.22613	H39	0.799	0.2942	0.61126
C15	0.43071	0.17607	0.28169	H40	0.77342	-0.32069	0.46425
C16	0.71508	0.1779	0.5859	H41	0.67549	-0.31815	0.47528
C17	0.77201	0.16387	0.58102	H42	0.85971	-0.2074	0.45585
C18	0.79418	-0.00542	0.53666	H43	0.5792	0.04255	0.88313
C19	0.75828	-0.17451	0.49805	H44	0.94764	-0.0873	0.58367
C20	0.70145	-0.17104	0.50406	H45	0.43057	0.00871	0.92223
N21	0.85304	-0.00243	0.53323	O46	0.05227	-0.05831	0.60475
N22	0.50051	-0.02362	0.84827	O47	0.61285	0.00924	0.98116
C23	0.88073	-0.1066	0.49237	H48	0.09232	0.01393	0.60709
C24	0.94167	-0.08679	0.49545	H49	0.63763	0.00899	1.01572
C25	0.53575	0.01445	0.88957				

Table S24. Fractional atomic coordinates for the unit cell of sonoCOF-D3.

SonoCOF-D3: Space group P2

a= 24.0650 Å, b= 4.0888 Å, c= 24.5156 Å<br>α=90°, β=89.8491°, γ=90°, R_{wp}=5.89 %, R_p=3.94 %							
Atom	X (Å)	Y (Å)	Z (Å)	Atom	X (Å)	Y (Å)	Z (Å)
C1	0.55224	0.16891	0.41148	C25	0.53463	0.10028	0.88715
C2	0.4959	0.11776	0.4236	C26	0.51602	0.09216	0.94454
C3	0.47952	0.11265	0.47911	C27	0.96624	-0.32568	0.54653
C4	0.57765	0.11176	0.50695	C28	0.02465	-0.3264	0.5516
C5	0.59232	0.16259	0.45223	C29	0.45952	0.09262	0.9588
C6	0.45519	0.08219	0.38225	C30	0.44385	0.09303	0.01424
C7	0.3995	0.04668	0.39742	H31	0.56622	0.21416	0.37041
C8	0.38186	0.07136	0.4516	H32	0.63493	0.19963	0.44011
C9	0.46788	0.07484	0.32271	H33	0.36901	-0.00308	0.36605
C10	0.32098	0.03923	0.46214	H34	0.53602	-0.26883	0.32787
C11	0.51093	-0.11836	0.30133	H35	0.55516	-0.26794	0.22895
C12	0.5213	-0.12331	0.24523	H36	0.41728	0.38145	0.20376
C13	0.48705	0.04809	0.2093	H37	0.40092	0.40465	0.30154
C14	0.44317	0.23572	0.23024	H38	0.70311	0.35717	0.60445
C15	0.43401	0.2503	0.28634	H39	0.80345	0.25369	0.59466
C16	0.71729	0.19381	0.57266	H40	0.77057	-0.38277	0.45911
C17	0.77423	0.13867	0.56699	H41	0.67117	-0.3021	0.47116
C18	0.79434	-0.06503	0.52598	H42	0.862	-0.36387	0.45063
C19	0.75663	-0.21533	0.4902	H43	0.57804	0.15006	0.87873
C20	0.69949	-0.16777	0.49685	F44	0.93321	-0.3428	0.5912
N21	0.85283	-0.12689	0.52489	F45	0.42037	0.1021	0.91914
N22	0.50044	0.03685	0.84771	F46	0.04982	-0.33044	0.60104
C23	0.88119	-0.28071	0.48806	F47	0.61039	0.09622	0.97077
C24	0.94173	-0.3142	0.49469				

Table S25. Fractional atomic coordinates for the unit cell of sonoCOF-E3.

SonoCOF-E3: Space group P2M							
a= 28.0496 Å, b= 2.6879 Å, c= 30.4041 Å<br>α=90°, β=81.7481°, γ=90°, R_{wp}=7.63 %, R_p=6.13 %							
Atom	X (Å)	Y (Å)	Z (Å)	Atom	X (Å)	Y (Å)	Z (Å)
C1	-0.50286	-1	0.43531	C30	-0.45746	-1	0.83047

C2	-0.51701	-1	0.48274	C31	-0.47031	-1	0.87959
C3	-0.43534	-1	0.50383	C32	-0.51585	-1	0.89594
C4	-0.42482	-1	0.45754	C33	-0.52745	-1	0.94254
C5	-0.45641	-1	0.42549	C34	-0.49377	-1	0.97468
C6	-0.53539	-1	0.40051	C35	-0.4479	-1	0.95728
C7	-0.58103	-1	0.41776	C36	-0.43652	-1	0.91064
C8	-0.59897	-1	0.46311	H37	-0.3924	-1	0.44262
C9	-0.52753	-1	0.34655	H38	-0.44104	-1	0.3933
C10	-0.65307	-1	0.47012	H39	-0.6043	-1	0.39414
C11	-0.48482	-1	0.32043	H40	-0.45191	-1	0.33169
C12	-0.48086	-1	0.27296	H41	-0.44754	-1	0.25533
C13	-0.51855	-1	0.2476	H42	-0.59171	-1	0.25234
C14	-0.56113	-1	0.27052	H43	-0.60072	-1	0.32898
C15	-0.56552	-1	0.31794	H44	-0.66956	-1	0.39665
C16	-0.68106	-1	0.43169	H45	-0.74731	-1	0.40515
C17	-0.72849	-1	0.43546	H46	-0.74452	-1	0.54915
C18	-0.75218	-1	0.47719	H47	-0.66899	-1	0.5454
C19	-0.72749	-1	0.51575	H48	-0.18288	-1	0.4521
C20	-0.67989	-1	0.5124	H49	-0.12551	-1	0.56362
N21	-0.19902	-1	0.52159	H50	-0.04514	-1	0.57085
C22	-0.1709	-1	0.48588	H51	-0.02446	-1	0.42374
C23	-0.12136	-1	0.49049	H52	-0.10505	-1	0.41834
C24	-0.10319	-1	0.53309	H53	-0.42177	-1	0.81992
C25	-0.05622	-1	0.53689	H54	-0.5426	-1	0.87248
C26	-0.02552	-1	0.49801	H55	-0.56318	-1	0.95217
C27	-0.04475	-1	0.45525	H56	-0.41983	-1	0.97877
C28	-0.09176	-1	0.45169	H57	-0.40104	-1	0.89878
N29	-0.48841	-1	0.80102				

Table S26. Fractional atomic coordinates for the unit cell of sonoCOF-F3.

SonoCOF-F3: Space group P-1							
a= 41.0625 Å, b= 17.05 Å, c= 3.9461 Å<br>α=89.9887°, β=89.9935°, γ=90.1667°, R_{wp}=6.07 %, R_p=4.42 %							
Atom	X (Å)	Y (Å)	Z (Å)	Atom	X (Å)	Y (Å)	Z (Å)

C1	0.08725	0.85062	0.95734	C43	0.47563	0.20263	0.50338
C2	0.07649	0.7839	1.13117	N44	0.29762	0.43495	1.20779
C3	0.09552	0.71643	1.1488	N45	0.46525	0.96016	1.62081
C4	0.12618	0.71448	1.00295	C46	1.06894	1.06597	1.10137
C5	0.13699	0.78006	0.82102	C47	1.03461	1.06663	1.10635
C6	0.11764	0.8474	0.79792	C48	1.01722	0.99884	1.0097
C7	0.17726	0.49653	1.14699	C49	1.0341	0.9287	0.9336
C8	0.19408	0.42669	1.21444	C50	1.06827	0.92543	0.97105
C9	0.22791	0.42807	1.23711	C51	1.08504	0.99485	1.04363
C10	0.24505	0.49789	1.18661	C52	1.01712	1.13283	1.21066
C11	0.22797	0.56719	1.11951	C53	0.98364	1.13549	1.18633
C12	0.19408	0.56719	1.09986	H54	0.05365	0.78451	1.26108
C13	0.17684	0.64139	1.03038	H55	0.08713	0.66628	1.29007
C14	0.28076	0.49921	1.1875	H56	0.15996	0.77892	0.69204
C15	0.17701	0.35125	1.24858	H57	0.12632	0.89691	0.65413
N16	0.14548	0.64532	1.05002	H58	0.19102	0.69341	0.98162
C17	0.08878	0.1388	1.13488	H59	0.29257	0.55538	1.16138
C18	0.11767	0.13905	1.32185	H60	0.19082	0.30033	1.32262
C19	0.13711	0.20619	1.33553	H61	0.12486	0.08779	1.46324
C20	0.1278	0.27467	1.16417	H62	0.15866	0.20483	1.48841
C21	0.09897	0.27478	0.98276	H63	0.09196	0.32686	0.84612
C22	0.08006	0.20729	0.96104	H64	0.0589	0.20817	0.80196
N23	0.14679	0.34504	1.16352	H65	0.43185	0.28875	0.88195
C24	0.43563	0.40853	1.04202	H66	0.42414	0.54216	1.39105
C25	0.45724	0.47162	1.0995	H67	0.46036	0.64844	1.48259
C26	0.48944	0.46742	0.98084	H68	0.60388	0.47277	1.15718
C27	0.50054	0.39861	0.82164	H69	0.6618	0.46197	1.10677
C28	0.4804	0.33152	0.80977	H70	0.66502	0.68069	0.57231
C29	0.44787	0.33847	0.90849	H71	0.60573	0.69398	0.63851
C30	0.44773	0.53821	1.27974	H72	0.53687	0.26306	1.01321
C31	0.46861	0.60079	1.32601	H73	0.56065	0.13984	0.8151
C32	0.49332	0.25317	0.71229	H74	0.4757	0.09418	0.22953
C33	0.40001	0.41598	1.09825	H75	0.45208	0.21943	0.40992
C34	0.61665	0.51875	1.02874	H76	0.44232	0.9775	1.54046

C35	0.65001	0.51165	0.99247	H77	0.47706	0.99399	1.80095
C36	0.66777	0.56954	0.82372	H78	1.11117	0.99347	1.05581
C37	0.65148	0.63548	0.70034	H79	1.0291	1.18347	1.31433
C38	0.61784	0.64291	0.73878	H80	0.97185	1.18857	1.26549
C39	0.52359	0.22797	0.83388	H81	0.15112	0.4958	1.12907
C40	0.53707	0.15751	0.72532	H82	0.241	0.37439	1.28684
C41	0.52006	0.10951	0.50454	H83	0.24113	0.62121	1.07908
C42	0.48899	0.13129	0.40022				

Table S27. Fractional atomic coordinates for the unit cell of sonoCOF-G3.

SonoCOF-G3: Space group P1											
a= 52.2448 Å, b= 23.5896 Å, c= 3.6684 Å<br>α=90.0233°, β=90.0104°, γ=91.3388°, R_{wp}=2.50 %, R_p=1.90 %											
Atom	X (Å)	Y (Å)	Z (Å)	Atom	X (Å)	Y (Å)	Z (Å)	Atom	X (Å)	Y (Å)	Z (Å)
C1	0.09356	0.88981	1.21407	C74	0.38708	0.47739	1.28318	H147	0.2626	0.39067	1.38748
C2	0.08402	0.8488	1.39487	C75	0.39925	0.43561	1.439	H148	0.30831	0.38139	1.41227
C3	0.09621	0.80181	1.3917	C76	0.42563	0.42905	1.42814	H149	0.31492	0.53696	1.19307
C4	0.11941	0.79346	1.22313	C77	0.52229	0.66778	1.22943	H150	0.26854	0.5467	1.16817
C5	0.13031	0.83474	1.05871	C78	0.51866	0.71879	1.27894	H151	0.2159	0.60575	0.9533
C6	0.11753	0.88205	1.05394	C79	0.53651	0.74555	1.46314	H152	0.19825	0.68989	0.91205
N7	0.18133	0.47972	1.23924	C80	0.55782	0.72066	1.60338	H153	0.1279	0.64215	1.33974
C8	0.19371	0.43431	1.27757	C81	0.56104	0.66936	1.56453	H154	0.14568	0.5564	1.37821
N9	0.2194	0.4326	1.28769	N82	0.35971	0.48436	1.27158	H155	0.14468	0.40842	1.07747
C10	0.23316	0.47389	1.25919	N83	0.53304	0.79794	1.50695	H156	0.12625	0.32541	1.10629
N11	0.22069	0.51847	1.21969	N84	0.5156	0.17733	0.77802	H157	0.19469	0.26699	1.58403
C12	0.19487	0.52229	1.21038	C85	1.0778	1.03213	1.24526	H158	0.21285	0.34801	1.55447
C13	0.1824	0.57347	1.17	C86	1.05076	1.03197	1.27328	H159	0.16432	0.75275	0.96929
C14	0.18041	0.38564	1.31278	C87	1.03751	0.98762	1.2232	H160	0.35016	0.41192	1.36753
C15	0.26154	0.46949	1.27479	C88	1.05134	0.9421	1.17249	H161	0.16094	0.20988	1.51117
C16	0.27357	0.42318	1.34366	C89	1.07841	0.93957	1.19878	H162	0.12515	0.0545	1.52888
C17	0.29994	0.41778	1.3585	C90	1.091	0.98528	1.22126	H163	0.14672	0.13326	1.51944
C18	0.31541	0.45832	1.3048	C91	1.03636	1.07481	1.36097	H164	0.08522	0.19995	0.92746
C19	0.30362	0.50491	1.23632	C92	1.01018	1.07596	1.36094	H165	0.06489	0.12111	0.91426
C20	0.277	0.51042	1.22203	C93	0.99631	1.0343	1.27452	H166	0.4603	0.37628	1.07266
C21	0.19662	0.61265	1.03903	C94	1.01014	0.98877	1.22422	H167	0.46632	0.54302	1.49345
C22	0.18645	0.66133	1.01492	C95	0.9969	0.9444	1.17452	H168	0.49982	0.59891	1.56061
C23	0.16166	0.67352	1.12425	C96	1.0113	0.90156	1.08687	H169	0.58157	0.43175	0.81486
C24	0.14709	0.63451	1.25293	C97	1.03747	0.90044	1.08628	H170	0.54801	0.3762	0.74204
C25	0.15727	0.58514	1.27332	C98	0.96922	1.03677	1.24758	H171	0.58769	0.5982	1.23721
C26	0.15564	0.37793	1.19205	C99	0.95665	0.99102	1.22555	H172	0.6079	0.44068	1.33254
C27	0.14511	0.33014	1.20674	C100	0.96986	0.94417	1.20258	H173	0.65396	0.4282	1.31329
C28	0.159	0.28894	1.3465	C101	0.95532	0.89571	1.2158	H174	0.65972	0.56611	0.75279

C29	0.1834	0.2973	1.47248	C102	0.93138	0.8906	1.05682	H175	0.6143	0.57797	0.76631
C30	0.19387	0.34426	1.45491	C103	0.91905	0.84461	1.05827	H176	0.53915	0.32583	1.23383
C31	0.15216	0.72694	1.09727	C104	0.9304	0.80184	1.21737	H177	0.54616	0.23733	1.12602
C32	0.34347	0.44964	1.32141	C105	0.95358	0.80755	1.38574	H178	0.47642	0.2342	0.54932
C33	0.14933	0.23695	1.37029	C106	0.96533	0.85339	1.39263	H179	0.47039	0.32368	0.62332
N34	0.13052	0.74305	1.22734	N107	0.86638	0.49626	1.18719	H180	0.43964	0.5335	0.98035
C35	0.09237	0.08052	1.23213	C108	0.85407	0.54159	1.14105	H181	0.3936	0.54593	1.01217
C36	0.1163	0.08557	1.39123	N109	0.82839	0.54314	1.11816	H182	0.38865	0.4078	1.57238
C37	0.1287	0.13149	1.38855	C110	0.81463	0.50177	1.14166	H183	0.43404	0.39605	1.54711
C38	0.11741	0.1742	1.22795	N111	0.82704	0.45732	1.1893	H184	0.50894	0.64887	1.06707
C39	0.09422	0.16853	1.05984	C112	0.85281	0.45365	1.21136	H185	0.50228	0.73769	1.16441
C40	0.08239	0.1228	1.05462	C113	0.86519	0.40256	1.25944	H186	0.57178	0.7409	1.74685
N41	0.12825	0.22334	1.22559	C114	0.86739	0.59036	1.11257	H187	0.57748	0.65122	1.68246
C42	0.46875	0.4512	1.19746	C115	0.78631	0.50588	1.11135	H188	0.51718	0.81677	1.40303
C43	0.48874	0.48483	1.23857	C116	0.77435	0.55189	1.03146	H189	0.54624	0.81825	1.64438
C44	0.51392	0.4704	1.13754	C117	0.74804	0.55691	1.00179	H190	0.50241	0.15706	0.63989
C45	0.51923	0.42208	1.00556	C118	0.73254	0.51631	1.05164	H191	0.53167	0.15862	0.87636
C46	0.49986	0.387	0.99942	C119	0.74426	0.47002	1.13134	H192	1.11141	0.98436	1.22051
C47	0.4751	0.40276	1.08692	C120	0.77083	0.46487	1.16039	H193	1.04492	1.10878	1.43292
C48	0.48461	0.53161	1.39137	C121	0.8508	0.36361	1.39033	H194	1.0012	1.11077	1.4315
C49	0.50416	0.56442	1.42682	C122	0.86089	0.31489	1.41849	H195	1.00275	0.86757	1.01514
C50	0.52863	0.55278	1.30019	C123	0.88575	0.3025	1.31335	H196	1.04644	0.86564	1.01554
C51	0.53394	0.50441	1.16916	C124	0.90049	0.34132	1.18604	H197	0.93625	0.99189	1.22587
C52	0.55914	0.4899	1.06946	C125	0.8904	0.39069	1.16171	H198	0.92246	0.92168	0.92005
C53	0.56326	0.4432	0.91574	C126	0.89217	0.5978	1.23498	H199	0.90105	0.84282	0.92673
C54	0.54369	0.41054	0.87785	C127	0.90269	0.64565	1.22686	H200	0.96264	0.77609	1.51693
C55	0.54806	0.58777	1.3068	C128	0.8888	0.6872	1.09236	H201	0.98282	0.85509	1.53335
C56	0.57285	0.57184	1.2219	C129	0.86446	0.67908	0.96323	H202	0.78535	0.58443	0.99051
C57	0.57917	0.52335	1.11255	C130	0.85401	0.63206	0.97382	H203	0.73972	0.59308	0.93968
C58	0.5047	0.33289	0.92576	C131	0.8952	0.2491	1.34279	H204	0.73294	0.43792	1.17211
C59	0.44098	0.46368	1.26119	C132	0.70453	0.52468	1.0201	H205	0.77924	0.42883	1.22303
C60	0.60697	0.5106	1.05306	C133	0.89832	0.73937	1.07742	H206	0.83145	0.37072	1.47252
C61	0.54327	0.64195	1.37764	N134	0.9197	0.75255	1.21555	H207	0.84897	0.28648	1.52084
C62	0.6189	0.46784	1.19885	N135	0.68823	0.48999	1.06775	H208	0.91976	0.33353	1.10341
C63	0.6454	0.46085	1.19099	C136	0.95404	0.08648	1.23101	H209	0.90212	0.41928	1.05786
C64	0.66088	0.49675	1.04494	C137	0.96361	0.12754	1.05116	H210	0.90316	0.56703	1.34528
C65	0.64894	0.53842	0.88437	C138	0.95134	0.17444	1.05282	H211	0.92155	0.65016	1.32854
C66	0.62255	0.54505	0.88845	C139	0.92804	0.18264	1.21898	H212	0.8532	0.70962	0.85428
C67	0.52582	0.30705	1.07048	C140	0.9171	0.1413	1.3819	H213	0.83509	0.62857	0.87161
C68	0.52965	0.2562	1.01505	C141	0.92996	0.09408	1.38815	H214	0.88303	0.22337	1.47136
C69	0.51185	0.22954	0.82907	N142	0.91686	0.23296	1.2135	H215	0.6979	0.56221	0.96579
C70	0.49036	0.25437	0.69366	H143	0.06657	0.85252	1.53606	H216	0.88631	0.76699	0.94853
C71	0.48694	0.30556	0.73804	H144	0.08747	0.77152	1.52655	H217	0.98115	0.12396	0.91182
C72	0.42883	0.50636	1.11758	H145	0.14823	0.83067	0.9247	H218	0.96008	0.2048	0.9189
C73	0.40234	0.5133	1.13248	H146	0.12605	0.91202	0.91331	H219	0.89904	0.14523	1.51268

Table S28. Fractional atomic coordinates for the unit cell of sonoCOF-H3.

SonoCOF-H3: Space group P1											
a= 52.2448 Å, b= 23.5896 Å, c= 3.6684 Å<br>α=90.0233°, β=90.0104°, γ=91.3388°, R_{wp}=2.50 %, R_p=1.90 %											
Atom	X (Å)	Y (Å)	Z (Å)	Atom	X (Å)	Y (Å)	Z (Å)	Atom	X (Å)	Y (Å)	Z (Å)
C1	0.09705	0.86961	1.12721	C76	0.42695	0.40823	1.58183	H151	0.23795	0.55536	0.89224
C2	0.09125	0.81341	1.32337	C77	0.51302	0.71657	1.03079	H152	0.21331	0.65748	0.75437
C3	0.10752	0.7597	1.32762	C78	0.50619	0.77843	1.0333	H153	0.15264	0.5899	1.40703
C4	0.13006	0.76092	1.14119	C79	0.52246	0.80995	1.21572	H154	0.17698	0.48892	1.53366
C5	0.13633	0.81717	0.94846	C80	0.54573	0.77942	1.39399	H155	0.1767	0.4369	1.02001
C6	0.11993	0.87103	0.9421	C81	0.55263	0.71759	1.39188	H156	0.15189	0.34523	1.10159
C12	0.22454	0.45331	1.31752	N82	0.35528	0.47285	1.38665	H157	0.212	0.25388	1.85719
C13	0.20995	0.51394	1.23217	N83	0.51537	0.87293	1.21993	H158	0.23717	0.34597	1.76199
C14	0.2094	0.39881	1.378	N84	0.53307	0.11252	1.15321	H159	0.17323	0.72825	0.80526
C15	0.25395	0.44708	1.33961	C85	1.08097	1.03011	1.26846	H160	0.3502	0.38091	1.40225
C16	0.26816	0.39445	1.27511	C86	1.05259	1.03301	1.27966	H161	0.17193	0.19919	1.74093
C17	0.29626	0.3879	1.29788	C87	1.03817	0.9848	1.19857	H162	0.1262	0.02889	1.67173
C18	0.31107	0.43432	1.3808	C88	1.05225	0.93267	1.12129	H163	0.15407	0.11365	1.70264
C19	0.29734	0.48714	1.44512	C89	1.08082	0.9274	1.14402	H164	0.10117	0.23406	0.9577
C20	0.26914	0.4932	1.42691	C90	1.09458	0.97683	1.20919	H165	0.07312	0.14834	0.92346
C21	0.21952	0.56259	1.00684	C91	1.03832	1.08204	1.3781	H166	0.46343	0.34445	1.27833
C22	0.20542	0.62089	0.92844	C92	1.01037	1.08584	1.37495	H167	0.46312	0.54782	1.49612
C23	0.18125	0.63158	1.07321	C93	0.99552	1.04092	1.27147	H168	0.49487	0.62513	1.45027
C24	0.17131	0.58296	1.29257	C94	1.00958	0.98872	1.19524	H169	0.58488	0.43738	0.88129
C25	0.18543	0.52516	1.36928	C95	0.99512	0.94046	1.11567	H170	0.55312	0.36024	0.9252
C26	0.18487	0.39732	1.19846	C96	1.00941	0.89147	1.01669	H171	0.58466	0.64055	1.10035
C27	0.17049	0.34497	1.24903	C97	1.03738	0.88782	1.01759	H172	0.61255	0.45059	1.39841
C28	0.18013	0.29247	1.48495	C98	0.967	1.04634	1.24848	H173	0.66157	0.43431	1.35846
C29	0.20428	0.29365	1.67106	C99	0.95309	0.99691	1.1863	H174	0.65942	0.59995	0.61274
C30	0.21865	0.3463	1.6181	C100	0.96671	0.94329	1.12922	H175	0.61052	0.61615	0.64477
C31	0.16664	0.69323	0.99897	C101	0.95075	0.89027	1.10046	H176	0.54759	0.29204	1.49358
C32	0.34071	0.4263	1.39945	C102	0.9274	0.89817	0.88702	H177	0.56001	0.18355	1.48115
C33	0.16538	0.2364	1.53502	C103	0.91185	0.84858	0.87185	H178	0.48986	0.18145	0.84047
N34	0.14632	0.70429	1.16815	C104	0.91942	0.79019	1.0722	H179	0.47725	0.28994	0.84804
C35	0.09705	0.0826	1.30291	C105	0.94233	0.78284	1.28911	H180	0.43501	0.53416	0.98302
C36	0.12034	0.07366	1.51804	C106	0.95786	0.83224	1.30423	H181	0.3859	0.54884	1.02457
C37	0.13628	0.1224	1.53561	C108	0.82398	0.51766	1.07972	H182	0.38912	0.38326	1.77183
C38	0.1292	0.18097	1.33573	C113	0.83764	0.45604	1.16992	H183	0.438	0.36846	1.73719
C39	0.10626	0.18953	1.11848	C114	0.83992	0.57085	1.01659	H184	0.50065	0.69347	0.8797
C40	0.09033	0.14092	1.10072	C115	0.79456	0.52627	1.05332	H185	0.48851	0.80224	0.889
N41	0.14513	0.23237	1.33215	C116	0.78107	0.58037	1.11072	H186	0.55829	0.80354	1.53683
C42	0.46937	0.43766	1.31119	C117	0.75301	0.58925	1.08307	H187	0.57071	0.6949	1.53109
C43	0.48839	0.48229	1.30123	C118	0.73748	0.54382	1.00206	H188	0.49872	0.89695	1.07623
C44	0.51462	0.46972	1.20246	C119	0.75047	0.48958	0.94479	H189	0.52661	0.89588	1.3693
C45	0.52218	0.41153	1.13159	C120	0.77864	0.48116	0.96788	H190	0.52184	0.08947	1.00439

C46	0.50396	0.36491	1.17809	C121	0.82702	0.40873	1.39313	H191	0.54983	0.0887	1.29539
C47	0.4777	0.37938	1.25723	C122	0.83967	0.34896	1.46904	H192	1.11637	0.97359	1.2169
C48	0.48213	0.53848	1.39498	C123	0.86355	0.33509	1.32637	H193	1.04869	1.11742	1.46447
C49	0.5005	0.58327	1.36781	C124	0.87479	0.38271	1.11152	H194	1.00049	1.12382	1.46085
C50	0.52581	0.57372	1.24486	C125	0.86199	0.44209	1.03583	H195	0.99901	0.85602	0.93153
C51	0.53336	0.51551	1.17428	C126	0.86432	0.57166	1.20028	H196	1.04726	0.84994	0.93091
C52	0.5596	0.5029	1.07598	C127	0.87904	0.62367	1.15097	H197	0.9313	1.00045	1.18011
C53	0.56585	0.44675	0.98193	C128	0.8698	0.67631	0.91306	H198	0.92125	0.94273	0.73186
C54	0.54748	0.40203	1.00828	C129	0.8459	0.67541	0.72183	H199	0.89399	0.85649	0.70605
C55	0.54406	0.62028	1.19863	C130	0.83128	0.62322	0.77272	H200	0.94779	0.73855	1.45071
C56	0.57036	0.60571	1.1206	C131	0.87601	0.26943	1.4035	H201	0.97515	0.82558	1.47981
C57	0.57866	0.54742	1.06698	C132	0.7079	0.55411	0.97996	H202	0.79212	0.61671	1.17158
C58	0.51193	0.29996	1.16237	C133	0.8845	0.73252	0.8656	H203	0.7434	0.6315	1.12906
C59	0.44035	0.45054	1.36041	N134	0.90401	0.73785	1.07588	H204	0.73874	0.4541	0.87938
C60	0.6077	0.53418	1.02079	N135	0.6927	0.50859	0.99647	H205	0.78782	0.4389	0.92231
C61	0.53615	0.68524	1.21392	C136	0.95131	0.10466	1.26227	H206	0.80872	0.41805	1.50576
C62	0.62244	0.48255	1.21944	C137	0.95628	0.16241	1.06938	H207	0.83083	0.3134	1.63965
C63	0.65026	0.47329	1.19879	C138	0.93856	0.21556	1.06863	H208	0.89322	0.37392	0.99634
C64	0.6639	0.51636	0.98886	C139	0.91527	0.21158	1.25642	H209	0.87113	0.47716	0.87069
C65	0.64924	0.56729	0.7844	C140	0.90955	0.15377	1.44594	H210	0.8719	0.53217	1.38401
C66	0.62138	0.57615	0.80074	C141	0.92839	0.10903	1.41845	H211	0.89747	0.62326	1.3021
C67	0.53519	0.26884	1.34342	N142	0.89934	0.26389	1.24295	H212	0.83849	0.71519	0.53385
C68	0.54216	0.20711	1.33936	H143	0.0746	0.81153	1.4816	H213	0.81288	0.62394	0.62601
C69	0.52586	0.17543	1.15828	H144	0.10311	0.71681	1.48364	H214	0.86116	0.23935	1.32475
C70	0.50245	0.2057	0.98233	H145	0.15379	0.82005	0.80468	H215	0.69901	0.60018	0.97346
C71	0.49543	0.26745	0.98551	H146	0.12516	0.91387	0.79375	H216	0.87841	0.76904	0.65445
C72	0.42532	0.50189	1.16269	H147	0.25763	0.35757	1.2116	H217	0.97308	0.16628	0.91322
C73	0.39745	0.51025	1.18433	H148	0.30647	0.34672	1.24668	H218	0.9424	0.2594	0.91752
C74	0.38411	0.46662	1.3948	H149	0.30852	0.52327	1.51278	H219	0.8929	0.14636	1.60201
C75	0.39909	0.41626	1.59959	H150	0.25933	0.53438	1.47809	H151	0.23795	0.55536	0.89224

Table S29. Fractional atomic coordinates for the unit cell of sonoCOF-J3.

SonoCOF-J3: Space group CMMM							
a= 16.4705 Å, b= 30.7826 Å, c= 3.0169 Å<br>α=β=γ=90°, R_{wp}=8.76 %, R_p=6.18 %							
Atom	X (Å)	Y (Å)	Z (Å)	Atom	X (Å)	Y (Å)	Z (Å)
C1	-0.06682	0.07953	-0.5	H15	-0.22498	-0.0672	-0.5
C2	-0.0645	0.04013	-0.5	H16	-0.16638	-0.19792	-0.5
C3	-0.12551	0.01853	-0.5	H17	-0.07024	-0.15828	-0.5
C4	-0.12898	-0.10453	-0.5	H18	0.31129	-0.10624	-0.5
C5	-0.20249	-0.09443	-0.5	H19	-0.38724	-0.14108	-0.5
C6	-0.25813	-0.11871	-0.5	H20	0.38524	-0.24831	-0.5

C7	-0.249	-0.1568	-0.5	C21	0	0.01975	-0.5
C8	-0.17791	-0.16838	-0.5	C22	0	0.09587	-0.5
C9	-0.12162	-0.14405	-0.5	H23	0	0.12542	-0.5
N10	-0.30717	-0.18173	-0.5	C24	0	0.32338	-0.5
C11	-0.37281	-0.17024	-0.5	C25	0	0.24805	-0.5
C12	-0.43419	-0.19442	-0.5	H26	0	0.35351	-0.5
C13	0.43546	-0.23296	-0.5	H27	0	0.21794	-0.5
H14	-0.1793	0.02829	-0.5				

Table S30. Fractional atomic coordinates for the unit cell of sonoCOF-K3.

SonoCOF-K3: Space group CMMM							
a= 18.6595 Å, b= 38.3636 Å, c= 3.4543 Å<br>α=β=γ=90°, R_{wp}=4.53 %, R_p=2.77 %							
Atom	X (Å)	Y (Å)	Z (Å)	Atom	X (Å)	Y (Å)	Z (Å)
C1	-0.06829	0.07841	-0.5	H14	-0.18322	0.02821	-0.5
C2	-0.06597	0.03954	-0.5	H15	-0.23018	-0.06684	-0.5
C3	-0.12843	0.01832	-0.5	H16	-0.16693	-0.19572	-0.5
C4	-0.13148	-0.10327	-0.5	H17	-0.07028	-0.1561	-0.5
C5	-0.20685	-0.09371	-0.5	H18	0.3181	-0.10663	-0.5
C6	-0.26304	-0.11814	-0.5	H19	-0.39637	-0.14307	-0.5
C7	-0.25222	-0.15565	-0.5	H20	0.38263	-0.24964	-0.5
C8	-0.17951	-0.16663	-0.5	C21	0	0.01948	-0.5
C9	-0.12287	-0.14224	-0.5	C22	0	0.0944	-0.5
N10	-0.30998	-0.18102	-0.5	H23	0	0.12351	-0.5
C11	-0.37805	-0.17141	-0.5	N24	0	0.32038	-0.5
C12	-0.43674	-0.19754	-0.5	C25	0	0.24536	-0.5
C13	0.43501	-0.2355	-0.5	H26	0	0.21561	-0.5

Table S31. Fractional atomic coordinates for the unit cell of sonoCOF-A4.

SonoCOF-A4: Space group P2							
a= 7.4664 Å, b= 16.6879 Å, c= 9.5758 Å<br>α=90°, β=93.4826, γ=90°, R_{wp}=7.19 %, R_p=5.50 %							
Atom	X (Å)	Y (Å)	Z (Å)	Atom	X (Å)	Y (Å)	Z (Å)
N1	0.23832	2.71397	-1.83666	C20	-0.24941	2.21144	-1.2454

C2	0.37099	2.75914	-1.78872	C21	-0.35252	2.26737	-1.32912
C3	-0.37295	2.84235	-1.16395	C22	-0.27154	2.32953	-1.39976
C4	-0.47519	2.89682	-1.25352	H23	0.46513	2.74127	-1.69586
C5	-0.45128	2.97768	-1.2296	H24	-0.56547	2.87698	-1.34776
C6	-0.32599	3.00515	-1.11469	H25	-0.14015	2.97052	-0.92635
C7	-0.23533	2.9506	-1.01709	H26	-0.17888	2.82856	-0.97076
C8	-0.25754	2.86976	-1.04211	H27	0.15794	3.10434	-1.97031
C9	0.26863	3.08826	-1.89064	H28	0.07116	2.64382	-1.11848
N10	-0.32975	3.13751	-1.21325	H29	0.17621	2.53041	-1.25644
C11	-0.1969	2.63846	-1.22352	H30	-0.343	2.50684	-1.49569
C12	-0.02244	2.61108	-1.19405	H31	-0.4529	2.61477	-1.34847
C13	0.03718	2.54683	-1.27282	H32	0.16052	2.28704	-1.28638
C14	-0.07727	2.50953	-1.38378	H33	0.02012	2.1782	-1.16533
C15	-0.25533	2.53336	-1.4068	H34	-0.49469	2.25884	-1.34854
C16	-0.31612	2.59661	-1.32542	H35	-0.35108	2.36796	-1.4753
C17	0.08615	2.33626	-1.61247	H36	-0.52321	3.01884	-1.30576
C18	0.01679	2.28386	-1.29415	C37	0	2.46243	-1.5
C19	-0.06401	2.22186	-1.22371	C38	0	2.38302	-1.5

Table S32. Fractional atomic coordinates for the unit cell of sonoCOF-B4.

SonoCOF-B4: Space group P2							
a= 19.8859 Å, b= 6.2542 Å, c= 19.6534 Å<br>α=90°, β=89.3677, γ=90°, R_{wp}=8.50 %, R_p=6.22 %							
Atom	X (Å)	Y (Å)	Z (Å)	Atom	X (Å)	Y (Å)	Z (Å)
C1	0.47748	0.00053	0.47353	C20	0.95553	-0.18579	0.55601
C2	0.50069	0.00957	0.40144	C21	0.02664	-0.18975	0.56641
C3	0.40209	-0.01044	0.48426	C22	0.44183	0.06896	0.95804
C4	0.52378	-0.17559	0.36806	C23	0.43438	0.07195	0.02907
C5	0.53729	-0.17231	0.29785	H24	0.52935	-0.3235	0.39583
C6	0.52421	0.01209	0.25971	H25	0.55475	-0.31555	0.27223
C7	0.50252	0.19857	0.29303	H26	0.49313	0.34277	0.26402
C8	0.49194	0.19812	0.36358	H27	0.47487	0.34305	0.38816
C9	0.63981	0.16596	0.53095	H28	0.61751	0.31799	0.54446
C10	0.71129	0.14498	0.53138	H29	0.74325	0.27904	0.54591

C11	0.74205	-0.04914	0.51395	H30	0.72368	-0.3771	0.4846
C12	0.70057	-0.22446	0.49658	H31	0.59765	-0.34607	0.48994
C13	0.62892	-0.20614	0.49889	H32	0.83222	-0.2477	0.43251
N14	0.81509	-0.06865	0.51987	H33	0.56497	0.13247	0.83327
N15	0.46879	0.00617	0.81304	H34	0.92137	-0.1834	0.59967
C16	0.85373	-0.17641	0.47823	H35	0.39648	0.06918	0.92597
C17	0.92846	-0.18543	0.48953	H36	0.04665	-0.18949	0.61801
C18	0.51639	0.07667	0.8538	H37	0.38327	0.07356	0.05096
C19	0.50753	0.07075	0.92849				

Table S33. Fractional atomic coordinates for the unit cell of sonoCOF-J4.

SonoCOF-J4: Space group P2							
a= 6.3319 Å, b= 15.0036 Å, c= 8.5603 Å<br>α=90°, β=92.6688, γ=90°, R_{wp}=8.96 %, R_p=7.07 %							
Atom	X (Å)	Y (Å)	Z (Å)	Atom	X (Å)	Y (Å)	Z (Å)
N1	0.18402	2.71033	-1.88476	C20	-0.25202	2.21229	-1.22613
C2	0.28798	2.77107	-1.84485	C21	-0.30126	2.2133	-1.3658
C3	-0.28084	2.84934	-1.08252	C22	-0.23298	2.27462	-1.45135
C4	-0.31818	2.92016	-1.15702	H23	0.35809	2.76942	-1.74584
C5	-0.29384	2.9968	-1.09746	H24	-0.35849	2.91561	-1.26405
C6	-0.23791	3.00225	-0.95943	H25	-0.21647	3.06099	-0.91211
C7	-0.20456	2.93164	-0.88323	H26	-0.15876	2.9361	-0.77747
C8	-0.22392	2.85536	-0.94487	H27	-0.19126	2.80114	-0.88669
C9	0.31176	3.06893	-1.81394	H28	0.33755	3.05814	-1.70642
N10	-0.27221	3.14125	-1.14151	H29	0.12955	2.62255	-1.11944
C11	-0.15662	2.63781	-1.19418	H30	0.21267	2.51571	-1.28576
C12	0.02214	2.60041	-1.18972	H31	-0.35172	2.52848	-1.45574
C13	0.069	2.53991	-1.28414	H32	-0.43642	2.63513	-1.2891
C14	-0.06518	2.51436	-1.3817	H33	0.01461	2.38384	-1.21461
C15	-0.24894	2.54738	-1.37989	H34	-0.12673	2.28025	-1.06348
C16	-0.29536	2.60865	-1.28625	H35	-0.38467	2.16548	-1.41098
C17	0.11223	2.33533	-1.60017	H36	-0.2632	2.27156	-1.56022
C18	-0.07917	2.33872	-1.25795	C37	0	2.466	-1.5
C19	-0.15473	2.27876	-1.17184	C38	0	2.38397	-1.5

Table S34. Fractional atomic coordinates for the unit cell of sonoCOF-K4.

SonoCOF-K4: Space group P2							
a= 6.5547 Å, b= 14.8273 Å, c= 9.2284 Å<br>α=90°, β=72.6399, γ=90°, R_{wp}=8.59 %, R_p=5.81 %							
Atom	X (Å)	Y (Å)	Z (Å)	Atom	X (Å)	Y (Å)	Z (Å)
N1	0.18402	2.71033	-1.88476	C20	-0.25202	2.21229	-1.22613
C2	0.28798	2.77107	-1.84485	C21	-0.30126	2.2133	-1.3658
C3	-0.28084	2.84934	-1.08252	C22	-0.23298	2.27462	-1.45135
N4	-0.31818	2.92016	-1.15702	H23	0.35809	2.76942	-1.74584
C5	-0.29384	2.9968	-1.09746	H24	-0.21647	3.06099	-0.91211
C6	-0.23791	3.00225	-0.95943	H25	-0.15876	2.9361	-0.77747
C7	-0.20456	2.93164	-0.88323	H26	-0.19126	2.80114	-0.88669
C8	-0.22392	2.85536	-0.94487	H27	0.33755	3.05814	-1.70642
C9	0.31176	3.06893	-1.81394	H28	0.12955	2.62255	-1.11944
N10	-0.27221	3.14125	-1.14151	H29	0.21267	2.51571	-1.28576
C11	-0.15662	2.63781	-1.19418	H30	-0.35172	2.52848	-1.45574
C12	0.02214	2.60041	-1.18972	H31	-0.43642	2.63513	-1.2891
C13	0.069	2.53991	-1.28414	H32	0.01461	2.38384	-1.21461
C14	-0.06518	2.51436	-1.3817	H33	-0.12673	2.28025	-1.06348
C15	-0.24894	2.54738	-1.37989	H34	-0.38467	2.16548	-1.41098
C16	-0.29536	2.60865	-1.28625	H35	-0.2632	2.27156	-1.56022
C17	0.11223	2.33533	-1.60017	C36	0	2.466	-1.5
C18	-0.07917	2.33872	-1.25795	C37	0	2.38397	-1.5
C19	-0.15473	2.27876	-1.17184				

Table S35. Fractional atomic coordinates for the unit cell of sonoCOF-F5.

SonoCOF-F5: Space group P6/M							
a= 21.8748 Å, b= 21.8748 Å, c= 3.7288 Å<br>α=β=90°, γ=120°, R_{wp}=6.10 %, R_p=4.72 %							
Atom	X (Å)	Y (Å)	Z (Å)	Atom	X (Å)	Y (Å)	Z (Å)
N1	2.14429	-1.44006	0	C7	1.71486	-1.59725	0
C2	2.07169	-1.46872	0	H8	2.06883	-1.3742	0
C3	2.0399	-1.429	0	H9	1.94481	-1.42953	0
C4	1.96885	-1.46039	0	H10	1.82337	-1.66325	0

C5	1.80961	-1.62408	0	H11	1.75219	-1.54349	0
C6	1.73656	-1.64537	0				

Table S36. Fractional atomic coordinates for the unit cell of sonoCOF-I5.

SonoCOF-I5: Space group P6/M							
a= 23.1134 Å, b= 23.1134 Å, c= 3.4583 Å<br>α=β=90°, γ=120°, R_{wp}=10.10 %, R_p=7.55 %							
Atom	X (Å)	Y (Å)	Z (Å)	Atom	X (Å)	Y (Å)	Z (Å)
N1	0.13963	0.54663	0	C7	0.71081	0.40596	0
C2	0.06951	0.52448	0	H8	0.07624	0.62149	0
C3	0.04436	0.56841	0	H9	0.95789	0.57891	0
C4	0.97583	0.544	0	H10	0.82213	0.34729	0
C5	0.80766	0.38471	0	O11	0.74626	0.46604	0
C6	0.74089	0.36239	0	H12	0.15088	0.50864	0

Table S37. Fractional atomic coordinates for the unit cell of sonoCOF-F6.

SonoCOF-F6: Space group P6/M							
a= 31.9966 Å, b= 31.9966 Å, c= 3.6969 Å<br>α=β=90°, γ=120°, R_{wp}=9.66 %, R_p=7.42 %							
Atom	X (Å)	Y (Å)	Z (Å)	Atom	X (Å)	Y (Å)	Z (Å)
C1	1.61449	-1.68344	0	C9	1.48501	-1.56972	0
C2	1.63096	-1.71921	0	C10	1.5094	-1.59853	0
C3	1.63295	-1.59291	0	H11	1.57407	-1.69642	0
N4	1.58471	-1.60724	0	H12	1.66179	-1.55308	0
C5	1.56234	-1.57511	0	H13	1.63215	-1.50178	0
C6	1.59109	-1.5217	0	H14	1.59171	-1.45198	0
C7	1.56695	-1.49261	0	H15	1.44407	-1.59102	0
C8	1.51313	-1.51576	0	H16	1.48692	-1.6397	0

Table S38. Fractional atomic coordinates for the unit cell of sonoCOF-G6.

SonoCOF-G6: Space group P1											
a= 44.931 Å, b= 44.8445 Å, c= 3.5052 Å<br>α=90.273°, β=90.2346°, γ=119.456°, R_{wp}=8.15 %, R_p=6.69 %											
Atom	X (Å)	Y (Å)	Z (Å)	Atom	X (Å)	Y (Å)	Z (Å)	Atom	X (Å)	Y (Å)	Z (Å)
H1	0.32255	0.19415	0	H51	0.01808	0.09104	0	C101	0.69958	0.40682	0

H2	0.8716	0.67745	0	H52	0.98968	0.1279	0	C102	0.57278	0.30866	0
H3	0.79244	0.15439	0	C53	0.63153	0.31394	0	C103	0.53667	0.28863	0
H4	0.84561	0.63806	0	N54	0.65105	0.29846	0	C104	0.52012	0.25251	0
H5	0.80585	0.1284	0	C55	0.70724	0.30042	0	C105	0.5404	0.23644	0
H6	0.36194	0.20756	0	C56	0.73588	0.42722	0	C106	0.57653	0.25641	0
C7	0.6824	0.36847	0	C57	0.75196	0.46333	0	C107	0.75068	0.51708	0
N8	0.64741	0.34895	0	C58	0.73239	0.47988	0	N108	0.73737	0.53718	0
C9	0.59318	0.29276	0	C59	0.69604	0.4596	0	C109	0.42489	0.18481	0
C10	0.69134	0.26412	0	C60	0.67987	0.42347	0	C110	0.40943	0.20549	0
C11	0.71137	0.24804	0	C61	0.7664	0.24932	0	C111	0.3744	0.19063	0
C12	0.74749	0.26761	0	N62	0.79981	0.26263	0	C112	0.35281	0.15568	0
C13	0.76356	0.30396	0	C63	0.75992	0.57511	0	C113	0.36753	0.13413	0
C14	0.74359	0.32013	0	C64	0.79606	0.59057	0	C114	0.40347	0.14882	0
C15	0.48292	0.2336	0	C65	0.81623	0.6256	0	C115	0.31471	0.14206	0
N16	0.46282	0.20019	0	C66	0.80287	0.64719	0	C116	0.30321	0.16519	0
C17	0.81519	0.24008	0	C67	0.7666	0.63247	0	C117	0.26907	0.15536	0
C18	0.79451	0.20394	0	C68	0.74534	0.59653	0	C118	0.24391	0.12024	0
C19	0.80937	0.18377	0	C69	0.82735	0.68529	0	C119	0.25481	0.09575	0
C20	0.84432	0.19713	0	C70	0.86198	0.69679	0	C120	0.29006	0.10645	0
C21	0.86587	0.2334	0	C71	0.88628	0.73093	0	N121	0.20688	0.10981	0
C22	0.85118	0.25466	0	C72	0.87634	0.75609	0	C122	0.18323	0.07727	0
C23	0.85794	0.17265	0	C73	0.84094	0.74519	0	C123	0.14634	0.06299	0
C24	0.83481	0.13802	0	C74	0.81639	0.70994	0	C124	0.13046	0.08346	0
C25	0.84464	0.11372	0	N75	0.90292	0.79312	0	C125	0.09437	0.06788	0
C26	0.87976	0.12366	0	C76	0.89404	0.81677	0	C126	0.07339	0.03163	0
C27	0.90425	0.15906	0	C77	0.91666	0.85366	0	C127	0.0894	0.01134	0
C28	0.89355	0.18361	0	C78	0.953	0.86954	0	C128	0.12547	0.02698	0
N29	0.89019	0.09708	0	C79	0.97351	0.90563	0	C129	0.03508	0.01512	0
C30	0.92273	0.10596	0	C80	0.95824	0.92661	0	N130	0.01981	0.03482	0
C31	0.93701	0.08334	0	C81	0.92194	0.9106	0	H131	0.58566	0.33808	0
C32	0.91654	0.047	0	C82	0.90151	0.87453	0	H132	0.52034	0.30174	0
C33	0.93212	0.02649	0	C83	0.98004	0.96492	0	H133	0.52738	0.20701	0
C34	0.96837	0.04176	0	N84	0.01501	0.98019	0	H134	0.5928	0.24324	0
C35	0.98866	0.07806	0	H85	0.75242	0.41434	0	H135	0.42624	0.23488	0
C36	0.97302	0.09849	0	H86	0.7814	0.47966	0	H136	0.35011	0.10481	0
C37	0.98488	0.01996	0	H87	0.67962	0.47262	0	H137	0.41546	0.13147	0
N38	0.96518	0.98499	0	H88	0.65043	0.4072	0	H138	0.26082	0.17576	0
H39	0.66192	0.24758	0	H89	0.80864	0.57376	0	H139	0.23488	0.06695	0
H40	0.69826	0.2186	0	H90	0.75469	0.64989	0	H140	0.29879	0.08642	0
H41	0.79299	0.32038	0	H91	0.71601	0.58454	0	H141	0.14703	0.11288	0
H42	0.75676	0.34957	0	H92	0.91493	0.73918	0	H142	0.08168	0.08459	0
H43	0.76512	0.19136	0	H93	0.83207	0.76512	0	H143	0.07295	0.98192	0

H44	0.89519	0.24531	0	H94	0.78763	0.70121	0	H144	0.13823	0.01032	0
H45	0.86853	0.28399	0	H95	0.96585	0.85297	0	H145	0.46792	0.24809	0
H46	0.82424	0.08507	0	H96	0.00291	0.91832	0	H146	0.94347	0.13449	0
H47	0.93305	0.16793	0	H97	0.90896	0.92705	0	H147	0.75191	0.21983	0
H48	0.91358	0.21237	0	H98	0.8721	0.86177	0	H148	0.86551	0.80899	0
H49	0.88712	0.03415	0	C99	0.68606	0.3176	0	H149	0.78017	0.53208	0
H50	0.91541	0.99709	0	N100	0.70154	0.35259	0	H150	0.19101	0.05653	0

Table S39. Fractional atomic coordinates for the unit cell of sonoCOF-I6.

SonoCOF-I6: Space group P6/M							
a= 30.4728 Å, b= 30.4728 Å, c= 3.5061 Å α=β=90°, γ=120°, R_{wp}=5.33 %, R_p=3.60 %							
Atom	X (Å)	Y (Å)	Z (Å)	Atom	X (Å)	Y (Å)	Z (Å)
C1	0.61161	0.30972	0	C10	0.50051	0.39838	0
C2	0.63448	0.27704	0	O11	0.5661	0.2894	0
C3	0.6237	0.39742	0	H12	0.65067	0.43698	0
N4	0.57124	0.3832	0	H13	0.62392	0.48754	0
C5	0.5524	0.41776	0	H14	0.59012	0.54135	0
C6	0.58369	0.47008	0	H15	0.43983	0.41162	0
C7	0.56343	0.50185	0	H16	0.47543	0.35822	0
C8	0.51099	0.48276	0	H17	0.54362	0.3456	0
C9	0.48008	0.42995	0				

Table S40. Fractional atomic coordinates for the unit cell of sonoCOF-A7.

SonoCOF-A7: Space group P-3							
a= 31.6777 Å, b= 31.6777 Å, c= 4.5365 Å α=β=90°, γ=120°, R_{wp}=7.74 %, R_p=6.26 %							
Atom	X (Å)	Y (Å)	Z (Å)	Atom	X (Å)	Y (Å)	Z (Å)
C1	1.28222	-0.35696	0.04333	H13	1.467	-0.19079	0.31799
C2	1.4059	-0.25758	0.1942	H14	1.35541	-0.18557	-0.23126
C3	1.43218	-0.20825	0.19401	H15	1.30921	-0.27156	-0.23239
C4	1.41542	-0.1806	0.04416	H16	1.49024	-0.40012	-0.20139
C5	1.37059	-0.20505	-0.10682	H17	1.52199	-0.05755	0.21385
C6	1.34396	-0.25438	-0.10735	O18	1.58752	0.02869	0.29629
N7	1.44346	-0.13078	0.05141	C19	0.41038	0.38039	1.04954
C8	1.46672	-0.43346	-0.06972	H20	0.43056	0.37077	0.87716

C9	1.48333	-0.46721	-0.03273	H21	0.39303	0.39627	0.90481
C10	1.51154	-0.03251	0.11579	H22	0.38281	0.34798	1.17142
C11	1.5441	0.01536	0.1478	N23	1.33333	-0.33333	0.04318
H12	1.42085	-0.27728	0.31903				

Table S41. Fractional atomic coordinates for the unit cell of sonoCOF-B7.

SonoCOF-B7: Space group R-3							
a= 32.3991 Å, b= 32.3991 Å, c= 7.8008 Å<br>α=β=90°, γ=120°, R_{wp}=7.89 %, R_p=5.95 %							
Atom	X (Å)	Y (Å)	Z (Å)	Atom	X (Å)	Y (Å)	Z (Å)
C1	-0.38444	1.31113	0.07945	C11	-0.12592	0.67988	0.26175
C2	-0.25968	1.40716	0.16117	H12	-0.2459	1.38594	0.22309
C3	-0.23271	1.45651	0.17196	H13	-0.19841	1.47227	0.23999
C4	-0.24852	1.48602	0.1029	H14	-0.30701	1.48448	-0.04056
C5	-0.29256	1.46352	0.01791	H15	-0.35404	1.39839	-0.06307
C6	-0.31982	1.41415	0.00602	H16	-0.17709	1.26191	0.00208
N7	-0.22056	1.5357	0.12367	H17	-0.14557	1.60654	0.26274
C8	-0.20172	1.22978	0.07477	H18	-0.09385	0.689	0.33748
C9	-0.18486	1.19744	0.12221	N19	-0.33333	1.33333	0.07383
C10	-0.1556	1.63256	0.2187				

Table S42. Fractional atomic coordinates for the unit cell of sonoCOF-F7.

SonoCOF-F7: Space group P3							
a= 17.3329 Å, b= 17.3329 Å, c= 4.6876 Å<br>α=β=90°, γ=120°, R_{wp}=6.22 %, R_p=4.85 %							
Atom	X (Å)	Y (Å)	Z (Å)	Atom	X (Å)	Y (Å)	Z (Å)
C1	2.7007	-1.7303	0.02886	C10	2.38151	-1.53375	-0.16692
C2	2.76498	-1.6325	0.02852	H11	2.72704	-1.7796	0.02969
C3	2.59807	-1.53413	0.02995	H12	2.64928	-1.4595	0.05649
N4	2.50879	-1.56244	-0.01256	H13	2.57311	-1.39147	0.28324
C5	2.46748	-1.5026	-0.01136	H14	2.49808	-1.29441	0.27208
C6	2.5086	-1.41583	0.14994	H15	2.27169	-1.50461	-0.29022
C7	2.46472	-1.36075	0.14845	H16	2.349	-1.60088	-0.28976
C8	2.37885	-1.3908	-0.0117	N17	2.33333	-1.33333	-0.01245

C9	2.33793	-1.47839	-0.16881				

Table S43. Fractional atomic coordinates for the unit cell of sonoCOF-G7.

SonoCOF-G7: Space group P3							
a= 23.6 Å, b= 23.6 Å, c= 4.0224 Å<br>α=β=90°, γ=120°, R_{wp}=9.65 %, R_p=6.82 %							
Atom	X (Å)	Y (Å)	Z (Å)	Atom	X (Å)	Y (Å)	Z (Å)
N1	0.4545	0.98709	1.07352	C14	0.62295	0.52265	1.18594
C2	0.47168	0.94763	0.9414	C15	0.64113	0.37373	1.0461
C3	0.43454	0.87734	0.9355	N16	0.6011	0.30794	1.04622
C4	0.37304	0.84069	1.08777	H18	0.35003	0.86382	1.21762
C5	0.34024	0.77228	1.07869	H19	0.29329	0.7462	1.19545
C6	0.36728	0.73821	0.91859	H20	0.45202	0.75256	0.64396
C7	0.42864	0.77581	0.76503	H21	0.50893	0.87188	0.65475
C8	0.4617	0.8442	0.77405	H22	0.47518	0.42046	0.83031
C9	0.5599	0.50127	1.06153	H23	0.52144	0.34755	0.82214
C10	0.52377	0.43779	0.92969	H24	0.6982	0.49881	1.27768
C11	0.55006	0.39624	0.92569	H25	0.6515	0.57122	1.29065
C12	0.61331	0.41771	1.04923	H26	0.4077	0.9688	1.19449
C13	0.64962	0.48144	1.17784	N27	0.33333	0.66667	0.91534

Table S44. Fractional atomic coordinates for the unit cell of sonoCOF-H7.

SonoCOF-H7: Space group P3							
a= 21.1334 Å, b= 21.1334 Å, c= 4.5417 Å<br>α=β=90°, γ=120°, R_{wp}=11.92 %, R_p=8.83 %							
Atom	X (Å)	Y (Å)	Z (Å)	Atom	X (Å)	Y (Å)	Z (Å)
C1	3.6353	0.38066	-0.09157	C14	3.39059	0.52647	0.26524
C2	3.66713	0.4452	-0.26149	H15	3.71586	0.46039	-0.38922
C3	3.63613	0.49022	-0.26896	H16	3.66125	0.5391	-0.40586
C4	3.57332	0.4723	-0.10085	H17	3.49327	0.3935	0.20481
C5	3.54173	0.40831	0.07268	H18	3.5463	0.31321	0.20449
C6	3.57209	0.36269	0.07439	H19	3.56145	0.56293	-0.27943
C7	3.54124	0.52037	-0.11171	H20	3.52665	0.64229	-0.2034
N8	3.48939	0.51066	0.07228	H21	3.45864	0.70702	-0.1715

C9	3.4531	0.5528	0.08702	H22	3.30271	0.54168	0.41511
C10	3.47781	0.61903	-0.06823	H23	3.37187	0.47616	0.38913
C11	3.43907	0.65665	-0.0496	N24	3.66667	0.33333	-0.0894
C12	3.3751	0.62925	0.12261	N25	3.33333	0.66667	0.13065
C13	3.35162	0.56391	0.28145				

Table S45. Fractional atomic coordinates for the unit cell of sonoCOF-F8.

SonoCOF-F8: Space group P6/M							
a= 15.2777 Å, b= 15.2777 Å, c= 3.5181 Å<br>$\alpha=\beta=90°$, $\gamma=120°$, R_{wp}=4.46 %, R_p=3.38 %							
Atom	X (Å)	Y (Å)	Z (Å)	Atom	X (Å)	Y (Å)	Z (Å)
N1	3.46225	-2.05113	0	C4	3.30384	-2.2599	0
C2	3.51382	-1.87844	0	H5	3.4346	-1.89925	0
C3	3.40735	-2.22975	0	H6	3.28098	-2.20302	0

Table S46. Fractional atomic coordinates for the unit cell of sonoCOF-G8.

SonoCOF-G8: Space group P6/M							
a= 29.6412 Å, b= 29.6412 Å, c= 3.5308 Å<br>$\alpha=\beta=90°$, $\gamma=120°$, R_{wp}=7.35 %, R_p=6.17 %							
Atom	X (Å)	Y (Å)	Z (Å)	Atom	X (Å)	Y (Å)	Z (Å)
C1	1.49902	-0.94152	0	C9	1.36509	-1.28098	0
N2	1.51692	-0.97318	0	N10	1.3129	-1.30181	0
C3	1.46579	-1.11515	0	H11	1.45749	-0.95687	0
C4	1.4117	-1.13674	0	H12	1.39481	-1.11189	0
C5	1.37902	-1.19053	0	H13	1.33747	-1.20615	0
C6	1.39976	-1.22385	0	H14	1.47107	-1.22667	0
C7	1.45396	-1.20203	0	H15	1.52819	-1.13242	0
C8	1.4866	-1.14824	0				

Table S47. Fractional atomic coordinates for the unit cell of sonoCOF-I8.

SonoCOF-I8: Space group P6/M							
a= 14.9674 Å, b= 14.9674 Å, c= 3.2382 Å<br>$\alpha=\beta=90°$, $\gamma=120°$, R_{wp}=11.92 %, R_p=8.83 %							
Atom	X (Å)	Y (Å)	Z (Å)	Atom	X (Å)	Y (Å)	Z (Å)

N1	0.45967	0.95221	0	H5	0.44864	0.11157	0
C2	0.52445	0.12978	0	O6	0.26852	0.79416	0
C3	0.40548	0.77407	0	H7	0.38961	0.94301	0
C4	0.29871	0.73721	0				

Table S48. Fractional atomic coordinates for the unit cell of sonoCOF-I9.

SonoCOF-I9: Space group P6							
a= 23.091 Å, b= 23.091 Å, c= 3.8265 Å<br>α=β=90°, γ=120°, R_{wp}=4.61 %, R_p=3.29 %							
Atom	X (Å)	Y (Å)	Z (Å)	Atom	X (Å)	Y (Å)	Z (Å)
N1	0.14548	0.5592	-0.08409	C9	0.94197	0.59011	-0.02847
C2	0.0722	0.52956	-0.06251	H10	0.81865	0.33313	0.03756
C3	0.0421	0.57109	-0.05954	O11	0.75803	0.465	0.25665
C4	0.97075	0.54249	-0.05201	H12	0.16424	0.52935	-0.19805
C5	0.80537	0.37316	0.03837	H13	0.89529	0.56757	0.14075
C6	0.74043	0.3555	0.12187	H14	0.92759	0.59903	-0.29569
C7	0.71749	0.40624	0.1672	H15	0.97968	0.64034	0.08812
H8	0.07361	0.62608	-0.07016				

Table S49. Fractional atomic coordinates for the unit cell of sonoCOF-I10.

SonoCOF-I10: Space group P6/M							
a= 23.9116 Å, b= 23.9116 Å, c= 3.7333 Å<br>α=β=90°, γ=120°, R_{wp}=4.99 %, R_p=3.51 %							
Atom	X (Å)	Y (Å)	Z (Å)	Atom	X (Å)	Y (Å)	Z (Å)
N1	0.14548	0.5592	-0.08409	C9	0.94197	0.59011	-0.02847
C2	0.0722	0.52956	-0.06251	H10	0.81865	0.33313	0.03756
C3	0.0421	0.57109	-0.05954	O11	0.75803	0.465	0.25665
C4	0.97075	0.54249	-0.05201	H12	0.16424	0.52935	-0.19805
C5	0.80537	0.37316	0.03837	H13	0.89529	0.56757	0.14075
C6	0.74043	0.3555	0.12187	H14	0.92759	0.59903	-0.29569
C7	0.71749	0.40624	0.1672	H15	0.97968	0.64034	0.08812
H8	0.07361	0.62608	-0.07016				

Table S50. Fractional atomic coordinates for the unit cell of sonoCOF-A11.

SonoCOF-A11: Space group P6							
a= 41.3805 Å, b= 41.3805 Å, c= 4.7666 Å<br>α=β=90°, γ=120°, R_{wp}=3.89 %, R_p=3.13 %							
Atom	X (Å)	Y (Å)	Z (Å)	Atom	X (Å)	Y (Å)	Z (Å)
H1	0.15474	0.33408	1.22308	C26	0.75667	0.55238	0.72391
H2	0.26882	0.5203	0.53506	C27	0.78598	0.54613	0.69123
H3	0.17955	0.33016	1.51299	C28	0.81732	0.56646	0.85323
H4	0.25731	0.49031	0.23179	C29	0.84792	0.55943	0.83889
C5	0.03183	0.49868	1.24738	C30	0.72735	0.58379	0.95713
C6	0.03309	0.53191	1.24634	H31	0.05712	0.49886	1.26017
C7	0.00128	0.53383	1.22227	H32	0.05925	0.55604	1.25746
N8	0.99739	0.43189	1.17204	H33	0.97734	0.60369	1.48734
C9	0.96376	0.39975	1.10249	H34	0.92352	0.60774	1.40334
C10	0.96985	0.56944	1.12923	H35	0.89633	0.53511	0.70081
C11	0.96102	0.58996	1.30711	H36	0.95255	0.53416	0.76769
C12	0.92975	0.59168	1.263	H37	0.95167	0.42367	0.7495
C13	0.90596	0.57215	1.0463	H38	0.89364	0.36943	0.6396
C14	0.91406	0.55075	0.87024	H39	0.91007	0.31385	1.32894
C15	0.94617	0.55004	0.90864	H40	0.9655	0.36745	1.44558
C16	0.94268	0.39967	0.8757	H41	0.84361	0.60861	1.15842
C17	0.90971	0.36879	0.81272	H42	0.73222	0.53721	0.60329
C18	0.89814	0.33691	0.96553	H43	0.84758	0.54058	0.68609
C19	0.91909	0.3371	1.19383	H44	0.7322	0.6086	1.05511
C20	0.95138	0.36812	1.26094	O45	0.21466	0.47979	0.50397
N21	0.86436	0.30539	0.89516	O46	0.20889	0.37456	1.25694
N22	0.87427	0.57526	1.01708	C47	0.17408	0.82456	1.38568
C23	0.81934	0.59315	1.03688	C48	0.25658	0.75196	0.37359
C24	0.79031	0.59952	1.06834	H49	0.16609	0.36364	1.5289
C25	0.75856	0.57844	0.91339	H50	0.24388	0.5238	-0.75569

Table S51. Fractional atomic coordinates for the unit cell of sonoCOF-B11.

SonoCOF-B11: Space group P6

a= 44.9122 Å, b= 44.9122 Å, c= 4.7913 Å<br>α=β=90°, γ=120°, R_{wp}=6.21 %, R_p=4.64 %							
Atom	X (Å)	Y (Å)	Z (Å)	Atom	X (Å)	Y (Å)	Z (Å)
C1	0.03137	0.49863	0.11517	C22	0.75049	0.53689	0.05423
C2	0.03269	0.53147	0.08427	C23	0.77893	0.52985	0.06088
C3	0.00134	0.53394	0.09771	C24	0.81183	0.55427	-0.0779
N4	0.99747	0.43146	0.08958	C25	0.84305	0.5489	-0.06645
C5	0.96386	0.39768	0.11217	C26	0.72325	0.57373	-0.10203
C6	0.97015	0.57058	0.04451	H27	0.05698	0.49779	0.15525
C7	0.96509	0.59569	0.21043	H28	0.0594	0.55689	0.04747
C8	0.93419	0.59792	0.17276	H29	0.9864	0.61459	0.37855
C9	0.907	0.57424	-0.02253	H30	0.93108	0.61914	0.30177
C10	0.91214	0.54995	-0.19893	H31	0.89034	0.53085	-0.36257
C11	0.94407	0.54915	-0.17406	H32	0.94884	0.53096	-0.33256
C12	0.93679	0.3926	0.32106	H33	0.94191	0.41411	0.49771
C13	0.90339	0.36117	0.31408	H34	0.88107	0.35817	0.47256
C14	0.89708	0.33323	0.1134	H35	0.92197	0.31319	-0.21526
C15	0.9255	0.33662	-0.06919	H36	0.98022	0.37079	-0.22929
C16	0.95807	0.36856	-0.07268	H37	0.84189	0.60418	-0.33679
N17	0.86172	0.30232	0.10046	H38	0.72433	0.51738	0.17178
N18	0.87503	0.57637	-0.04415	H39	0.83899	0.52078	-0.07718
C19	0.81528	0.58474	-0.22536	H40	0.72171	0.59147	-0.28802
C20	0.78674	0.59147	-0.23711	H41	0.79005	0.61617	-0.36102
C21	0.75393	0.56737	-0.09408	H42	0.77534	0.50466	0.17735

Table S52. Fractional atomic coordinates for the unit cell of sonoCOF-E11.

SonoCOF-E11: Space group P6							
a= 50.7036 Å, b= 50.7036 Å, c= 5.7113 Å<br>α=β=90°, γ=120°, R_{wp}=3.55 %, R_p=2.49 %							
Atom	X (Å)	Y (Å)	Z (Å)	Atom	X (Å)	Y (Å)	Z (Å)
C1	0.02664	0.4985	0.22192	H27	0.04772	0.49818	0.22899
C2	0.02814	0.52675	0.21437	H28	0.05032	0.54695	0.20672
C3	0.00149	0.52878	0.2107	H29	0.98314	0.59013	0.42142
N4	0.9972	0.44207	0.18782	H30	0.93771	0.59458	0.32735

C5	0.96861	0.41401	0.16789	H31	0.92051	0.53727	-0.27478
C6	0.97615	0.55983	0.13512	H32	0.96582	0.53335	-0.18068
C7	0.96877	0.57795	0.27356	H33	0.95302	0.41562	0.51593
C8	0.94336	0.58066	0.21912	H34	0.90426	0.36808	0.47478
C9	0.92496	0.56509	0.02741	H35	0.93129	0.35858	-0.23149
C10	0.93328	0.54812	-0.1183	H36	0.97773	0.40405	-0.18044
C11	0.95892	0.54576	-0.06541	H37	0.77503	0.58371	-0.45306
C12	0.94772	0.40328	0.35305	H38	0.70277	0.5312	0.19837
C13	0.91988	0.37628	0.32924	H39	0.87204	0.52448	-0.16657
C14	0.9127	0.3595	0.12198	H40	0.67602	0.57073	-0.22868
C15	0.93426	0.37004	-0.06396	C41	0.79362	0.55371	0.84046
C16	0.96179	0.39682	-0.03763	C42	0.79176	0.52536	0.81398
N17	0.88353	0.33198	0.10004	C43	0.81809	0.52311	0.82017
N18	0.89804	0.56695	-0.01467	C44	0.84655	0.54908	0.85845
C19	0.75936	0.57211	-0.31084	C45	0.84849	0.57757	0.88048
C20	0.73258	0.57352	-0.29459	C46	0.82217	0.57991	0.86947
C21	0.7123	0.55899	-0.10983	H47	0.72782	0.58621	-0.42404
C22	0.71856	0.54251	0.05591	H48	0.74943	0.52775	0.16679
C23	0.74494	0.54056	0.03726	H49	0.76984	0.50494	0.79146
C24	0.76559	0.5555	-0.14531	H50	0.81617	0.50095	0.80257
C25	0.87368	0.54584	-0.11628	H51	0.8704	0.59782	0.90923
C26	0.68461	0.56106	-0.08817	H52	0.8239	0.60194	0.89184

Table S53. Fractional atomic coordinates for the unit cell of sonoCOF-F11.

SonoCOF-F11: Space group P-1							
a= 36.6493 Å, b= 15.7584 Å, c= 4.8096 Å<br>α=93.0775°, β=90.0088°, γ=93.1796°, R_{wp}=3.02 %, R_p=2.30 %							
Atom	X (Å)	Y (Å)	Z (Å)	Atom	X (Å)	Y (Å)	Z (Å)
C1	0.09123	0.91374	1.21725	H38	0.25293	0.6313	1.4025
C2	0.07368	0.83607	1.30379	C39	0.65079	1.59801	0.57234
C3	0.09274	0.75675	1.339	C40	0.6348	1.55377	0.79222
C4	0.12952	0.75301	1.28646	C41	0.59884	1.56826	0.85497
C5	0.14677	0.82933	1.18467	C42	0.57787	1.62686	0.69917
C6	0.12778	0.9088	1.15138	C43	0.59394	1.67107	0.47874

C7	0.17971	0.49663	1.4371	C44	0.63037	1.65803	0.41982
C8	0.19708	0.41483	1.48093	N45	0.54048	1.64024	0.76569
C9	0.23454	0.41246	1.50087	C46	0.52293	1.72528	0.7347
C10	0.25487	0.49	1.46812	C47	0.52065	1.56851	0.88699
C11	0.23727	0.57125	1.42859	C48	0.45841	1.19517	1.22091
C12	0.19976	0.57545	1.41371	C49	0.47641	1.11377	1.23265
C13	0.18261	0.66279	1.36732	C50	0.51334	1.11094	1.28153
C14	0.29431	0.48638	1.44782	C51	0.53205	1.18966	1.32677
C15	0.17722	0.3301	1.4887	C52	0.51411	1.2709	1.31893
N16	0.14817	0.67066	1.33476	C53	0.52132	1.48355	0.77142
C17	0.08949	0.08054	1.25372	C54	0.50029	1.41593	0.88195
C18	0.11361	0.08775	1.47163	N55	0.46813	1.97183	0.7151
C19	0.13188	0.16753	1.51992	H56	0.65043	1.51053	0.92188
C20	0.12587	0.2419	1.35233	H57	0.58746	1.53494	1.02971
C21	0.10063	0.23591	1.14352	H58	0.57822	1.71532	0.35268
C22	0.08281	0.15588	1.09262	H59	0.64264	1.69328	0.25208
N23	0.14552	0.32341	1.38142	H60	0.43018	1.19611	1.17141
N24	0.31178	0.41257	1.48554	H61	0.46176	1.05265	1.20159
H25	0.04529	0.83712	1.34765	H62	0.56054	1.1877	1.36595
H26	0.07909	0.69798	1.41173	H63	0.52928	1.33122	1.34991
H27	0.17468	0.82787	1.12747	H64	0.53738	1.47073	0.59251
H28	0.14159	0.96664	1.07332	H65	0.4999	1.35057	0.79168
H29	0.19968	0.72088	1.36689	H66	0.44059	1.97504	0.6807
H30	0.30842	0.5461	1.38686	H67	0.48231	2.03109	0.7388
H31	0.19134	0.27137	1.55728	N68	1.07195	1.99703	1.19443
H32	0.11822	0.03124	1.60327	C69	1.03543	1.99781	1.09856
H33	0.15021	0.1711	1.68974	C70	1.00977	2.05281	1.22169
H34	0.09597	0.29278	1.01337	C71	0.97459	2.05454	1.12409
H35	0.06449	0.15181	0.9225	H72	1.01701	2.0932	1.39534
H36	0.1508	0.49881	1.41983	H73	0.95468	2.09565	1.22148
H37	0.24805	0.34935	1.52773				

Table S54. Fractional atomic coordinates for the unit cell of sonoCOF-G11.

SonoCOF-G11: Space group P1

a= 51.3552 Å, b= 24.8977 Å, c= 4.7294 Å

α=63.8042°, β=105.4318°, γ=85.5203°, R_{wp}=4.18 %, R_p=3.17 %

Atom	X (Å)	Y (Å)	Z (Å)	Atom	X (Å)	Y (Å)	Z (Å)	Atom	X (Å)	Y (Å)	Z (Å)
C1	0.09648	0.86364	1.3035	C71	0.95697	0.85298	1.39904	H141	0.92253	0.96957	0.61246
C2	0.09541	0.80531	1.54019	N72	0.84707	0.51838	1.09614	H142	0.897	0.89167	0.59305
C3	0.11185	0.75216	1.58912	C73	0.83551	0.56978	1.06493	H143	0.94749	0.76391	1.6095
C4	0.12992	0.75642	1.4093	N74	0.8085	0.57847	1.09002	H144	0.97373	0.84201	1.62532
C5	0.13112	0.81472	1.17491	C75	0.79481	0.53544	1.15447	H145	0.77524	0.6137	1.34855
C6	0.11462	0.86749	1.12419	N76	0.80694	0.47689	1.20323	H146	0.72781	0.64107	1.3362
N7	0.2105	0.42894	1.50755	C77	0.83351	0.46827	1.16373	H147	0.70335	0.5146	0.97663
C8	0.22413	0.37609	1.54607	C78	0.84574	0.41559	1.18551	H148	0.75033	0.48792	0.98405
N9	0.25099	0.36968	1.56033	C79	0.85023	0.61991	1.00661	H149	0.81878	0.35434	1.41237
C10	0.26389	0.41632	1.52564	C80	0.76664	0.5483	1.17015	H150	0.83909	0.2672	1.40496
N11	0.25001	0.4694	1.48194	C81	0.75972	0.59165	1.26835	H151	0.89933	0.36494	0.98835
C12	0.22349	0.47544	1.48048	C82	0.73265	0.60736	1.26113	H152	0.8795	0.45353	0.99799
C13	0.20896	0.53235	1.45269	C83	0.71207	0.57966	1.15719	H153	0.88016	0.5741	1.50641
C14	0.20964	0.32655	1.55817	C84	0.71898	0.53601	1.06201	H154	0.90267	0.66108	1.42344
C15	0.2927	0.40961	1.5291	C85	0.746	0.5202	1.07009	H155	0.84604	0.76442	0.40951
C16	0.31323	0.35881	1.79302	C86	0.83518	0.35833	1.30659	H156	0.82298	0.67806	0.49871
C17	0.34073	0.35319	1.80154	C87	0.84688	0.30838	1.30813	H157	0.87258	0.21816	1.26687
C18	0.34797	0.39711	1.5413	C88	0.87037	0.30846	1.1835	H158	0.67811	0.63687	1.17778
C19	0.32719	0.44681	1.27105	C89	0.88163	0.36431	1.07248	H159	0.88199	0.81154	0.61012
C20	0.29977	0.45341	1.26718	C90	0.87062	0.41364	1.08142	H160	0.96773	0.18044	0.74698
C21	0.21931	0.58583	1.34297	C91	0.87288	0.61532	1.26584	H161	0.9382	0.268	0.70136
C22	0.20596	0.63904	1.32337	C92	0.88555	0.6649	1.21862	H162	0.90496	0.13177	1.4448
C23	0.18219	0.63974	1.41622	C93	0.87598	0.71928	0.91013	C163	0.53252	1.69628	-0.62034
C24	0.17189	0.58633	1.52795	C94	0.85362	0.72317	0.64955	C164	0.53167	1.59528	-0.1545
C25	0.18517	0.53307	1.54634	C95	0.8406	0.67404	0.69895	C165	0.54528	1.72478	-0.85942
C26	0.18083	0.34001	1.39881	C96	0.88117	0.25839	1.17705	C166	0.53326	1.78395	-1.12395
C27	0.16759	0.29366	1.38771	C97	0.68326	0.59891	1.13332	C167	0.50753	1.81484	-1.16351
C28	0.18289	0.23302	1.54134	C98	0.88858	0.77271	0.85855	C168	0.49377	1.78562	-0.93839
C29	0.21154	0.21921	1.70963	N99	0.90701	0.7726	1.10828	C169	0.50623	1.72698	-0.66682
C30	0.22481	0.26565	1.71569	N100	0.66445	0.57122	1.05459	C170	0.54201	1.5605	0.19209
C31	0.16825	0.69708	1.3854	C101	0.95295	0.10868	1.12223	C171	0.53026	1.51304	0.36159
C32	0.37745	0.39202	1.56657	C102	0.95416	0.17271	0.89501	C172	0.50704	1.49942	0.1912
C33	0.16947	0.18339	1.53326	C103	0.93756	0.22161	0.8668	C173	0.4969	1.53449	-0.16041
N34	0.14566	0.70086	1.45755	C104	0.91725	0.21145	1.05555	C174	0.50836	1.58335	-0.32975
C35	0.09841	0.06277	1.29009	C105	0.9155	0.14676	1.26364	N175	0.49592	1.44769	0.37317
C36	0.12042	0.04742	1.58088	C106	0.93374	0.11001	1.2511	C176	0.46694	1.45268	0.35139
C37	0.13551	0.09058	1.60056	N107	0.90253	0.25899	1.03328	C177	0.51468	1.38851	0.61477
C38	0.12931	0.14945	1.32516	H108	0.08232	0.8009	1.6919	C178	0.53341	1.37885	0.92031
C39	0.10738	0.1648	1.03438	H109	0.11092	0.70744	1.77107	C179	0.55253	1.32274	1.13854
C40	0.09199	0.12172	1.01561	H110	0.14399	0.81982	1.02152	C180	0.55185	1.27398	1.06763
N41	0.14405	0.19529	1.33909	H111	0.11592	0.91148	0.94272	C181	0.5312	1.28082	0.78596
N42	0.54686	0.63776	0.67428	H112	0.30798	0.32444	1.99597	C182	0.51251	1.33725	0.56445
C43	0.57696	0.62001	0.79916	H113	0.35647	0.31477	2.01364	C183	0.44455	1.47875	0.0507

C44	0.59072	0.56006	0.90386	H114	0.33235	0.48103	1.06666	C184	0.41797	1.46909	0.04729
C45	0.61954	0.54461	0.99379	H115	0.28395	0.4922	1.05882	C185	0.41285	1.4357	0.3437
C46	0.63531	0.58783	0.99295	H116	0.23756	0.58653	1.26872	C186	0.43461	1.41318	0.64482
C47	0.62184	0.64705	0.90486	H117	0.21413	0.67992	1.23368	C187	0.46096	1.42363	0.64626
C48	0.59297	0.66276	0.81097	H118	0.15363	0.586	1.60148	N188	0.4956	1.87598	-1.43084
N49	0.3849	0.43166	1.32912	H119	0.17707	0.49211	1.63717	N189	0.57186	1.21737	1.28355
C50	1.08187	1.01746	1.27394	H120	0.16877	0.38671	1.27509	H190	0.79601	0.44241	1.2607
C51	1.05387	1.02504	1.25774	H121	0.14543	0.30491	1.26	H191	0.56503	1.7015	-0.83407
C52	1.03852	0.98251	1.21805	H122	0.22368	0.17257	1.83278	H192	0.54364	1.80598	-1.3022
C53	1.05157	0.93104	1.21037	H123	0.24703	0.25418	1.8416	H193	0.47365	1.80871	-0.97067
C54	1.08036	0.92084	1.25911	H124	0.17734	0.73656	1.29717	H194	0.49607	1.70658	-0.48425
C55	1.09477	0.96529	1.28012	H125	0.39271	0.35576	1.79237	H195	0.55979	1.57012	0.32827
C56	1.04108	1.07301	1.28915	H126	0.18161	0.13664	1.68619	H196	0.53957	1.48879	0.62748
C57	1.01339	1.08087	1.26619	H127	0.12574	0.00261	1.79571	H197	0.48187	1.52181	-0.31056
C58	0.99709	1.04226	1.19991	H128	0.15148	0.07831	1.83388	H198	0.50056	1.6084	-0.60017
C59	1.01004	0.99141	1.18664	H129	0.10247	0.21019	0.8218	H199	0.53261	1.41341	0.99868
C60	0.99465	0.94911	1.14509	H130	0.07514	0.13418	0.78809	H200	0.56747	1.31663	1.36662
C61	1.00758	0.90047	1.11913	H131	0.5791	0.52552	0.90859	H201	0.52978	1.24254	0.73567
C62	1.03541	0.8919	1.14817	H132	0.62972	0.49879	1.06214	H202	0.49649	1.34049	0.35514
C63	0.96839	1.05222	1.15387	H133	0.63331	0.68196	0.89496	H203	0.44696	1.50653	-0.18427
C64	0.95366	1.00901	1.1212	H134	0.58295	0.70902	0.73657	H204	0.40159	1.48316	-0.1861
C65	0.96654	0.95708	1.12518	H135	1.11672	0.95808	1.31447	H205	0.43126	1.38752	0.87932
C66	0.95025	0.91104	1.11696	H136	1.05264	1.10421	1.33874	H206	0.47667	1.41429	0.87197
C67	0.92823	0.92459	0.82967	H137	1.00498	1.11717	1.3053	H207	0.47767	1.90208	-1.44754
C68	0.91369	0.87976	0.81998	H138	0.99606	0.86902	1.07204	H208	0.50468	1.89626	-1.61394
C69	0.92082	0.82094	1.10125	H139	1.04412	0.85472	1.11741	H209	0.58706	1.21105	1.49933
C70	0.9422	0.80859	1.39056	H140	0.93177	1.01617	1.08899	H210	0.57238	1.18169	1.22429

Table S55. Fractional atomic coordinates for the unit cell of sonoCOF-J11.

SonoCOF-J11: Space group C2/M							
a= 22.4674 Å, b= 16.3829 Å, c= 4.2789 Å α=90°, β=83.5325°, γ=90°, R_{wp}=7.52 %, R_p=5.49 %							
Atom	X (Å)	Y (Å)	Z (Å)	Atom	X (Å)	Y (Å)	Z (Å)
C1	-0.78807	-0.43086	0.45111	H13	-0.73034	-0.30653	0.21599
C2	-0.74801	-0.4303	0.20328	H14	-0.66823	-1.21548	0.07778
C3	-0.72174	-0.35809	0.09116	H15	-0.58681	-1.13235	-0.08272
N4	-0.68427	-0.35967	-0.13768	H16	-0.53226	-0.28768	-0.77316
C5	-0.64628	-0.30066	-0.25432	H17	-0.61344	-0.37196	-0.61017
C6	-0.63985	-1.23112	-0.11212	H18	-0.58258	-1.06995	-0.65077
C7	-0.59327	-1.18357	-0.2026	N19	-0.5	-1.16275	-0.5
C8	-0.55281	-1.20475	-0.43503	C20	-0.5	-1.08049	-0.5

C9	-0.56216	-0.27128	-0.58914	C21	-0.72871	-0.5	0.0797
C10	-0.6084	-0.31872	-0.49865	C22	-0.80784	-0.5	0.57445
C11	-0.54643	-1.03978	-0.58483	H23	-0.83657	-0.5	0.77419
H12	-0.80249	-0.37793	0.55406	H24	-0.69691	-0.5	-0.10859

Table S56. Fractional atomic coordinates for the unit cell of sonoCOF-K11.

SonoCOF-K11: Space group P21/M							
a= 4.5582 Å, b= 17.3644 Å, c= 24.2215 Å<br>α=90°, β=91.6359°, γ=90°, R_{wp}=7.52 %, R_p=5.49 %							
Atom	X (Å)	Y (Å)	Z (Å)	Atom	X (Å)	Y (Å)	Z (Å)
C1	0.08336	1.18026	0.54516	C23	0.85175	1.05417	0.10469
C2	0.29378	1.18115	0.50299	C24	1.00323	0.98399	0.10931
C3	0.38439	1.10938	0.47508	C25	0.95906	0.93606	0.1557
N4	0.57163	1.11314	0.43455	C26	0.76537	0.95775	0.19816
C5	0.65009	1.05413	0.39544	C27	0.60112	1.02527	0.19119
C6	0.49891	0.98391	0.3908	C28	0.64399	1.07283	0.1449
C7	0.54361	0.93598	0.34442	C29	0.61988	0.79036	0.20662
C8	0.73749	0.95772	0.30201	H30	1.50116	1.12633	-0.06084
C9	0.90151	1.02527	0.30902	H31	1.22578	1.05616	0.01443
C10	0.85815	1.07282	0.3553	H32	1.16657	0.96817	0.07926
C11	0.88336	0.79036	0.29352	H33	1.08623	0.88413	0.16011
H12	-0.00188	1.12632	0.56057	H34	0.44878	1.0427	0.22291
H13	0.27429	1.05618	0.48543	H35	0.52606	1.12674	0.14151
H14	0.33537	0.96806	0.4208	H36	0.51718	0.82094	0.17284
H15	0.41668	0.88403	0.33995	N37	0.75158	0.91517	0.25008
H16	1.054	1.04273	0.27734	C38	0.75164	0.8317	0.25007
H17	0.97589	1.12675	0.35873	N39	0.3914	0.25	0.48324
H18	0.98595	0.82093	0.32731	C40	-0.02457	0.25	0.56575
C19	1.41617	1.18025	-0.04536	H41	-0.19834	0.25	0.59625
C20	1.2063	1.18115	-0.00309	N42	1.10896	0.25	0.01672
C21	1.11608	1.10939	0.02488	C43	1.52387	0.25	-0.06599
N22	0.92964	1.11318	0.06554	H44	1.69721	0.25	-0.09658

6. ^{1}H NMR and ^{13}C NMR spectra

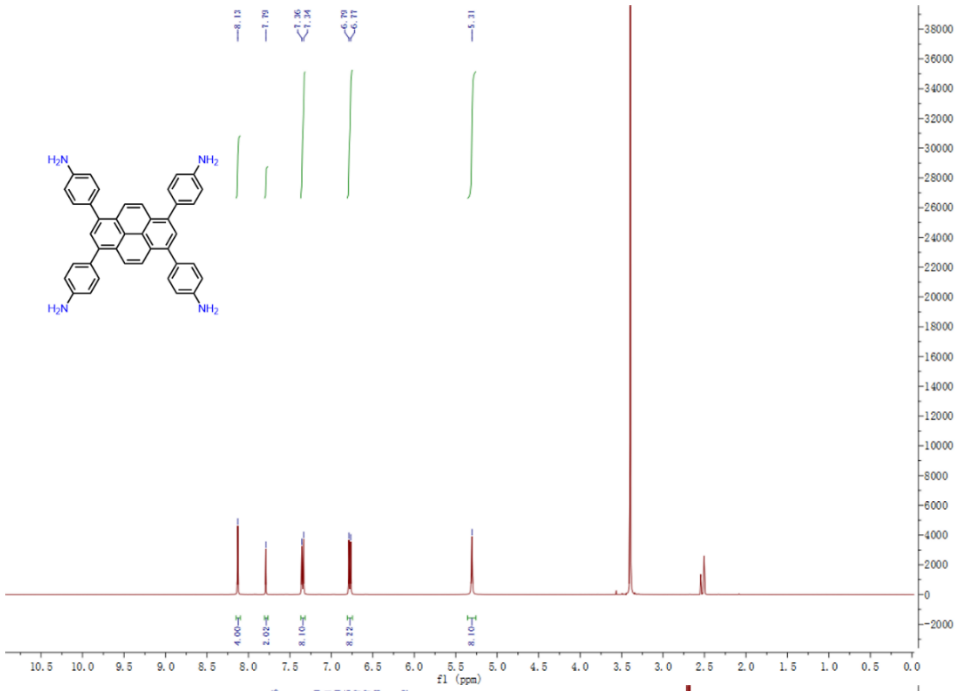

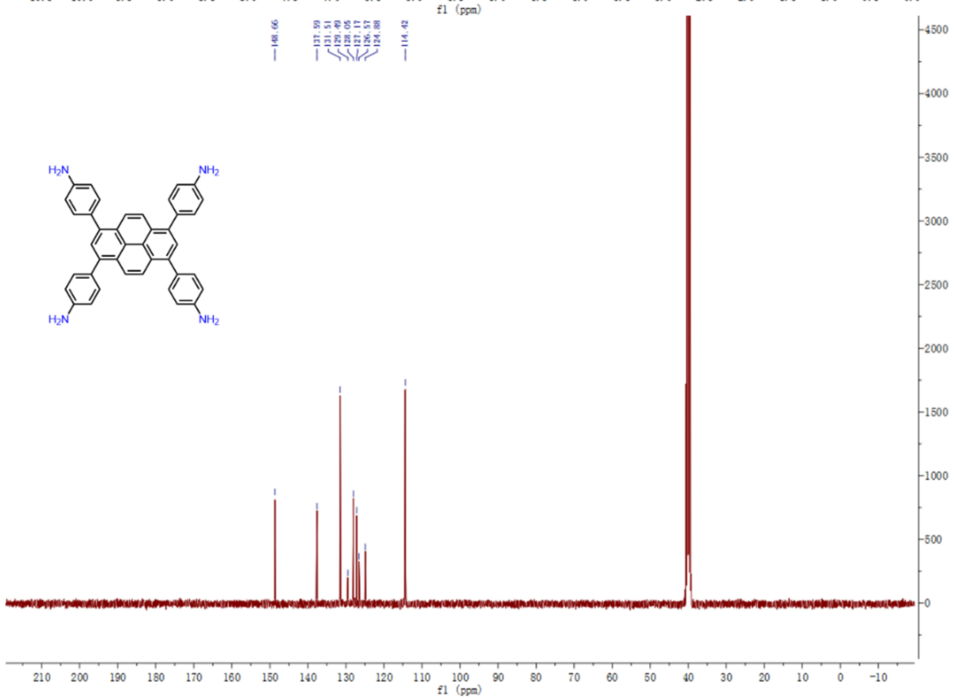

Figure S80 ^{1}H and ^{13}C NMR spectra of PTTA

7. References:

(1) Liu, L.; Gao, M.; Yang, H.; Wang, X.; Li, X.; Cooper, A. I. Linear Conjugated Polymers for Solar-Driven Hydrogen Peroxide Production: The Importance of Catalyst Stability. *J. Am. Chem. Soc.* **2021**, *143*, 19287-19293.

(2) Shiraishi, Y.; Takii, T.; Hagi, T.; Mori, S.; Kofuji, Y.; Kitagawa, Y.; Tanaka, S.; Ichikawa, S.; Hirai, T. Resorcinol-Formaldehyde Resins as Metal-Free Semiconductor Photocatalysts for Solar-to-Hydrogen Peroxide Energy Conversion. *Nat. Mater.* **2019**, *18*, 985-993.

(3) Shiraishi, Y.; Hagi, T.; Matsumoto, M.; Tanaka, S.; Ichikawa, S.; Hirai, T. Solar-to-Hydrogen Peroxide Energy Conversion on Resorcinol-Formaldehyde Resin Photocatalysts Prepared by Acid-Catalysed Polycondensation. *Commun. Chem.* **2020**, *3*, 169.

(4) Shiraishi, Y.; Matsumoto, M.; Ichikawa, S.; Tanaka, S.; Hirai, T. Polythiophene-Doped Resorcinol-Formaldehyde Resin Photocatalysts for Solar-to-Hydrogen Peroxide Energy Conversion. *J. Am. Chem. Soc.* **2021**, *143*, 12590-12599.

(5) Chen, L.; Wang, L.; Wan, Y.; Zhang, Y.; Qi, Z.; Wu, X.; Xu, H. Acetylene and Diacetylene Functionalized Covalent Triazine Frameworks as Metal-Free Photocatalysts for Hydrogen Peroxide Production: A New Two-Electron Water Oxidation Pathway. *Adv. Mater.* **2020**, *32*, 1904433.

(6) Wei, Z.; Liu, M.; Zhang, Z.; Yao, W.; Tan, H.; Zhu, Y. Efficient Visible-Light-Driven Selective Oxygen Reduction to Hydrogen Peroxide by Oxygen-Enriched Graphitic Carbon Nitride Polymers. *Energy Environ. Sci.* **2018**, *11*, 2581-2589.

(7) Teng, Z.; Zhang, Q.; Yang, H.; Kato, K.; Yang, W.; Lu, Y.; Liu, S.; Wang, C.; Yamakata, A.; Su, C.et al. Atomically Dispersed Antimony on Carbon Nitride for the Artificial Photosynthesis of Hydrogen Peroxide. *Nat. Catal.* **2021**, *4*, 374-384.

(8) Kofuji, Y.; Isobe, Y.; Shiraishi, Y.; Sakamoto, H.; Tanaka, S.; Ichikawa, S.; Hirai, T. Carbon Nitride-Aromatic Diimide-Graphene Nanohybrids: Metal-Free Photocatalysts for Solar-to-Hydrogen Peroxide Energy Conversion with 0.2% Efficiency. *J. Am. Chem. Soc.* **2016**, *138*, 10019-10025.

(9) Zeng, X.; Liu, Y.; Kang, Y.; Li, Q.; Xia, Y.; Zhu, Y.; Hou, H.; Uddin, M. H.; Gengenbach, T. R.; Xia, D.et al. Simultaneously Tuning Charge Separation and Oxygen Reduction Pathway on Graphitic Carbon Nitride by Polyethylenimine for Boosted Photocatalytic Hydrogen Peroxide Production. *ACS Catal.* **2020**, *10*, 3697-3706.

(10) Zhu, Z.; Pan, H.; Murugananthan, M.; Gong, J.; Zhang, Y. Visible Light-Driven

Photocatalytically Active g-C3N4 Material for Enhanced Generation of H_2O_2. *Appl. Catal. B-Environ.* **2018**, *232*, 19-25.

(11) Teng, Z.; Cai, W.; Liu, S.; Wang, C.; Zhang, Q.; Chenliang, S.; Ohno, T. Bandgap Engineering of Polymetric Carbon Nitride Copolymerized by 2,5,8-Triamino-Tri-S-Triazine (Melem) and Barbituric Acid for Efficient Nonsacrificial Photocatalytic H_2O_2 Production. *Appl. Catal. B-Environ.* **2020**, *271*, 118917.

(12) Xu, H.; Gao, J.; Jiang, D. Stable, Crystalline, Porous, Covalent Organic Frameworks as a Platform for Chiral Organocatalysts. *Nat. Chem.* **2015**, *7*, 905-912.

(13) Liu, X.; Guan, C.; Ding, S.; Wang, W.; Yan, H.; Wang, D.; Wan, L. On-Surface Synthesis of Single-Layered Two-Dimensional Covalent Organic Frameworks via Solid-Vapor Interface Reactions. *J. Am. Chem. Soc.* **2013**, *135*, 10470-10474.

(14) Biswal, B. P.; Kandambeth, S.; Chandra, S.; Shinde, D. B.; Bera, S.; Karak, S.; Garai, B.; Kharul, U. K.; Banerjee, R. Pore Surface Engineering in Porous, Chemically Stable Covalent Organic Frameworks for Water Adsorption. *J. Mater. Chem. A* **2015**, *3*, 23664-23669.

(15) Liao, Q.; Ke, C.; Huang, X.; Zhang, G.; Zhang, Q.; Zhang, Z.; Zhang, Y.; Liu, Y.; Ning, F.; Xi, K. Catalyst-Free and Efficient Fabrication of Highly Crystalline Fluorinated Covalent Organic Frameworks for Selective Guest Adsorption. *J. Mater. Chem. A* **2019**, *7*, 18959-18970.

(16) Liu, X.; Mo, Y.; Yue, J.; Zheng, Q.; Yan, H.; Wang, D.; Wan, L. Isomeric Routes to Schiff-Base Single-layered Covalent Organic Frameworks. *Small* **2014**, *10*, 4934-4939.

(17) de la Peña Ruigómez, A.; Rodríguez-San-Miguel, D.; Stylianou, K. C.; Cavallini, M.; Gentili, D.; Liscio, F.; Milita, S.; Roscioni, O. M.; Ruiz-González, M. L.; Carbonell, C.et al. Direct On-Surface Patterning of a Crystalline Laminar Covalent Organic Framework Synthesized at Room Temperature. *Chem. Eur. J.* **2015**, *21*, 10666-10670.

(18) Haase, F.; Gottschling, K.; Stegbauer, L.; Germann, L. S.; Gutzler, R.; Duppel, V.; Vyas, V. S.; Kern, K.; Dinnebier, R. E.; Lotsch, B. V. Tuning the Stacking Behaviour of a 2D Covalent Organic Framework Through Non-Covalent Interactions. *Mater. Chem. Front.* **2017**, *1*, 1354-1361.

(19) Zhai, L.; Huang, N.; Xu, H.; Chen, Q.; Jiang, D. A Backbone Design Principle for Covalent Organic Frameworks: the Impact of Weakly Interacting Units on CO_2 Adsorption. *Chem. Commun.* **2017**, *53*, 4242-4245.

(20) Kaleeswaran, D.; Vishnoi, P.; Murugavel, R. [3+3] Imine and β-Ketoenamine Tethered Fluorescent Covalent-Organic Frameworks for CO_2 Uptake and Nitroaromatic Sensing. *J. Mater. Chem. C* **2015**, *3*, 7159-7171.

(21) Zhi, Y.; Li, Z.; Feng, X.; Xi, H.; Zhang, Y.; Shi, Z.; Mu, Y.; Liu, X. Covalent Organic Frameworks as Metal-Free Heterogeneous Photocatalysts for Organic Transformations. *J. Mater. Chem. A* **2017**, *5*, 22933-22938.

(22) Gomes, R.; Bhanja, P.; Bhaumik, A. A Triazine-Based Covalent Organic Polymer for Efficient CO_2 Adsorption. *Chem. Commun.* **2015**, *51*, 10050-10053.

(23) Qian, H.; Dai, C.; Yang, C.; Yan, X. High-Crystallinity Covalent Organic Framework with Dual Fluorescence Emissions and Its Ratiometric Sensing Application. *ACS Appl. Mater. Interfaces* **2017**, *9*, 24999-25005.

(24) Qian, H. L.; Meng, F. L.; Yang, C. X.; Yan, X. P. Irreversible Amide-Linked Covalent Organic Framework for Selective and Ultrafast Gold Recovery. *Angew. Chem. Int. Ed.* **2020**, *59*, 17607-17613.

(25) Bai, L.; Gao, Q.; Zhao, Y. Two Fully Conjugated Covalent Organic Frameworks as Anode Materials for Lithium Ion Batteries. *J. Mater. Chem. A* **2016**, *4*, 14106-14110.

(26) EL-Mahdy, A. F. M.; Kuo, C.; Alshehri, A.; Young, C.; Yamauchi, Y.; Kim, J.; Kuo, S. Strategic Design of Triphenylamine- and Triphenyltriazine-Based Two-Dimensional Covalent Organic Frameworks for CO_2 Uptake and Energy Storage. *J. Mater. Chem. A* **2018**, *6*, 19532-19541.

(27) Gomesa, R.; Bhaumik, A. A New Triazine Functionalized Luminescent Covalent Organic Framework for Nitroaromatic Sensing and CO_2 Storage. *RSC Adv.* **2016**, *6*, 28047-28054.

(28) Wu, X.; Wang, B.; Yang, Z.; Chen, L. Novel Imine-Linked Covalent Organic Frameworks: Preparation, Characterization and Application. *J. Mater. Chem. A* **2019**, *7*, 5650-5655.

(29) Auras, F.; Ascherl, L.; Hakimioun, A. H.; Margraf, J. T.; Hanusch, F. C.; Reuter, S.; Bessinger, D.; Döblinger, M.; Hettstedt, C.; Karaghiosoff, K.et al. Synchronized Offset Stacking: A Concept for Growing Large-Domain and Highly Crystalline 2D Covalent Organic Frameworks. *J. Am. Chem. Soc.* **2016**, *138*, 16703-16710.

(30) Chen, X.; Huang, N.; Gao, J.; Xu, H.; Xu, F.; Jiang, D. Towards Covalent Organic Frameworks with Predesignable and Aligned Open Docking Sites. *Chem. Commun.* **2014**, *50*, 6161-6163.

(31) Leng, W.; Peng, Y.; Zhang, J.; Lu, H.; Feng, X.; Ge, R.; Dong, B.; Wang, B.; Hu, X.; Gao, Y. Sophisticated Design of Covalent Organic Frameworks with Controllable Bimetallic Docking for a Cascade Reaction. *Chem. Eur. J.* **2016**, *22*, 9087-9091.

(32) Banerjee, T.; Haase, F.; Trenker, S.; Biswal, B. P.; Savasci, G.; Duppel, V.; Moudrakovski, I.; Ochsenfeld, C.; Lotsch, B. V. Sub-Stoichiometric 2D Covalent Organic Frameworks from Tri- and Tetratopic Linkers. *Nat. Commun.* **2019**, *10*, 2689.

(33) Zhou, T.; Xu, S.; Wen, Q.; Pang, Z.; Zhao, X. One-Step Construction of Two Different Kinds of

Pores in a 2D Covalent Organic Framework. *J. Am. Chem. Soc.* **2014**, *136*, 15885-15888.

(34) Nguyen, H. L.; Hanikel, N.; Lyle, S. J.; Zhu, C.; Proserpio, D. M.; Yaghi, O. M. A Porous Covalent Organic Framework with Voided Square Grid Topology for Atmospheric Water Harvesting. *J. Am. Chem. Soc.* **2020**, *142*, 2218-2221.

(35) Zhao, W.; Yan, P.; Yang, H.; Bahri, M.; James, A. M.; Chen, H.; Liu, L.; Li, B.; Pang, Z.; Clowes, R.et al. Using Sound to Synthesize Covalent Organic Frameworks in Water. *Nat. Synth.* **2022**, *1*, 87-95.

(36) Ding, S.; Gao, J.; Wang, Q.; Zhang, Y.; Song, W.; Su, C.; Wang, W. Construction of Covalent Organic Framework for Catalysis: Pd/COF-LZU1 in Suzuki-Miyaura Coupling Reaction. *J. Am. Chem. Soc.* **2011**, *133*, 19816-19822.

(37) Kandambeth, S.; Mallick, A.; Lukose, B.; Mane, M. V.; Heine, T.; Banerjee, R. Construction of Crystalline 2D Covalent Organic Frameworks with Remarkable Chemical (Acid/Base) Stability via a Combined Reversible and Irreversible Route. *J. Am. Chem. Soc.* **2012**, *134*, 19524-19527.

(38) Gao, Q.; Bai, L.; Zeng, Y.; Wang, P.; Zhang, X.; Zou, R.; Zhao, Y. Reconstruction of Covalent Organic Frameworks by Dynamic Equilibrium. *Chem. Eur. J.* **2015**, *21*, 16818-16822.

(39) Guo, J.; Qian, H.; Zhao, X.; Yang, C.; Yan, X. In Situ Room-Temperature Fabrication of a Covalent Organic Framework and Its Bonded Fiber for Solid-Phase Microextraction of Polychlorinated Biphenyls in Aquatic Products. *J. Mater. Chem. A* **2019**, *7*, 13249-13255.

(40) Biswal, B. P.; Chandra, S.; Kandambeth, S.; Lukose, B.; Heine, T.; Banerjee, R. Mechanochemical Synthesis of Chemically Stable Isoreticular Covalent Organic Frameworks. *J. Am. Chem. Soc.* **2013**, *135*, 5328-5331.

(41) Chen, R.; Hu, T.; Zhang, W.; He, C.; Li, Y. Synthesis of Nitrogen-Containing Covalent Organic Framework with Reversible Iodine Capture Capability. *Microporous Mesoporous Mater.* **2021**, *312*, 110739.

(42) Dong, J.; Wang, Y.; Liu, G.; Cheng, Y.; Zhao, D. Isoreticular Covalent Organic Frameworks for Hydrocarbon Uptake and Separation: the Important Role of Monomer Planarity. *CrystEngComm* **2017**, *4899–4904*.

(43) Wang, S.; Yang, Y.; Zhang, H.; Zhang, Z.; Zhang, C.; Huang, X.; Kozawa, D.; Liu, P.; Li, B.; Wang, W. Toward Covalent Organic Framework Metastructures. *J. Am. Chem. Soc.* **2021**, *143*, 5003-5010.

(44) Peng, Y.; Huang, Y.; Zhu, Y.; Chen, B.; Wang, L.; Lai, Z.; Zhang, Z.; Zhao, M.; Tan, C.; Yang, N.et al. Ultrathin Two-Dimensional Covalent Organic Framework Nanosheets: Preparation and Application in Highly Sensitive and Selective DNA Detection. *J. Am. Chem. Soc.* **2017**, *139*,

8698-8704.

(45) Li, Z.; Feng, X.; Zou, Y.; Zhang, Y.; Xia, H.; Liu, X.; Mu, Y. A 2D Azine-Linked Covalent Organic Framework for Gas Storage Applications. *Chem. Commun.* **2014**, *50*, 13825-13828.

(46) Peng, Y.; Wong, W. K.; Hu, Z.; Cheng, Y.; Yuan, D.; Khan, S. A.; Zhao, D. Room Temperature Batch and Continuous Flow Synthesis of Water-Stable Covalent Organic Frameworks (COFs). *Chem. Mater.* **2016**, *28*, 5095-5101.

(47) Li, Z.; Zhi, Y.; Feng, X.; Ding, X.; Zou, Y.; Liu, X.; Mu, Y. An Azine-Linked Covalent Organic Framework: Synthesis, Characterization and Efficient Gas Storage. *Chem. Eur. J.* **2015**, *21*, 12079-12084.

(48) Karak, S.; Kumar, S.; Pachfule, P.; Banerjee, R. Porosity Prediction through Hydrogen Bonding in Covalent Organic Frameworks. *J. Am. Chem. Soc.* **2018**, *140*, 5138-5145.

(49) Krishnaraj, C.; Sekhar Jena, H.; Bourda, L.; Laemont, A.; Pachfule, P.; Roeser, J.; Chandran, C. V.; Borgmans, S.; Rogge, S. M. J.; Leus, K.et al. Strongly Reducing (Diarylamino)benzene-Based Covalent Organic Framework for Metal-Free Visible Light Photocatalytic H_2O_2 Generation. *J. Am. Chem. Soc.* **2020**, *142*, 20107-20116.
